# Data sets of eQTL loci, correlation analysis, and overlapped genes among gene sets that their expression levels are closely related to genes of Vegf family

**DOI:** 10.1016/j.dib.2018.09.004

**Published:** 2018-09-08

**Authors:** Jinglin Cui, Jing Li, Hang Lu, Hong Yu, Dongmei Wei, Yan Jiao, Monica M. Jablonski, Weikuan Gu, Hong Chen

**Affiliations:** a Center of Integrative Research, The First Hospital of Qiqihar City, Qiqihar, Heilongjiang 161005, PR China; b Department of Orthopedic Surgery and BME-Campbell Clinic, University of Tennessee Health Science Center, Memphis, TN 38163, USA; c Department of Ophthalmology, University of Tennessee Health Science Center, Memphis, TN 38163, USA; d Research Service, Veterans Affairs Medical Center, 1030 Jefferson Avenue, Memphis, TN 38104, USA

**Keywords:** Vegf family, eQTL, Mouse retina, Gene relationship, Expression levels

## Abstract

This data is generated from the analysis of similarities and differences in gene expression levels and pathways among genes (VegfA, VegfB, VegfC, and Pgf) in Vegf family using whole genome expression data generated from normal retina of eighty strains of mice. The results have been published in doi:10.1016/j.exer.2018.06.024 (Cui et al., 2018) [1]. Fig. 1 shows the expression quantitative trait loci (eQTL) that regulate the expression level of each gene in the Vegf family. The other three figure show the overlapped genes among the top 500 genes that their expression levels are most closely correlated to each of the Vegf genes. The four tables contain the information of top 50 genes that their expression levels are most closely correlated to each of the Vegf genes, and the correlation of the top 50 genes from one gene to the other genes in the Vegf family.

**Specifications table**

**Table:** 

Subject area	*Molecular Biology*
More specific subject area	*Gene expression and association of Vegf family genes*
Type of data	*Table and Figure*
How data was acquired	*GeneNetwork platform, excel organization and statistical analysis*
Data format	*Raw and analyzed data*
Experimental factors	*The top 50 genes that their expression levels are most closely correlated to each of the Vegf family genes were extracted from whole genome expression retina data at GeneNetwork database for the correlation comparison among the Vegf genes. Top 500 genes then were extracted for the comparison of overlapped genes among these correlated genes in Vegf family. Expression QTL (eQTL) for each of the Vegf genes were obtained for the comparison of their regulation mechanism among Vegf genes.*
Experimental features	*Top 50 genes from one gene was analyzed for their correlation to the other gene or genes in Vegf family. Top 500 genes from one gene was compared to the top 500 genes from the other gene or genes to obtain the name list of overlapped genes between two sets of 500 genes or among different genes in Vegf family. The loci of eQTL from different genes were used to compare their similarities and differences with others among genes in Vegf family.*
Data source location	*Data were obtained from Genenetwork database.*
Data accessibility	*All the data are presented in this article. Additional information can be found from GeneNetwork (*http://genenetwork.org/webqtl/main.py*) and**[1]*.
Related research article	*Jinglin Cui, Lidi Liu, Hang Lu, Dongmei Wei, Yan Jiao, Monica M. Jablonski, Robert W. Williams, Weikuan Gu, P Hong Chen. Potential Effect on Molecular Pathways in Different Targeted genes in the VEGF Family in Retina - From the Genomic Point of View. Exp Eye Res. 2018 Jun 23;176:78-87.**[1]*

**Value of the Data**

• This data can be useful in the construction of new pathways among Vegf genes.
• This data could be used as a reference for the new anti-Vegf drug development.
• The methodology in this data can be used for comparison of pathways of other genes.

## 1 Data

This data is generated from the analysis of similarities and differences in regulation of gene expression levels, most correlated genes to their expression levels, and overlapped genes among the top 500 genes that are most correlated to each of genes (VegfA, VegfB, VegfC, and pgf) in Vegf family. Data is obtained from the whole genome expression data generated from normal retina of eighty strains of mice, including 75 BXD RI strains, the parental strains (C57BL/6J and DBA/2J), the reciprocal crosses, and the BALB/cByJ mice (http://www.genenetwork.org/webqtl/main.py) [1], [2], [3]. Overlapped genes from top 500 genes among VegfA, VegfB and VegfC are shown in Fig. 2, Fig. 3, Fig. 4. Tables 1–4 shows the correlations to genes in Vegf family. Rs values of genes is the accumulation of all *R* values, which is equal to ∑*R*, for all the closely related genes to a Vegf family gene. Re is the average of the all closely related genes to a probe of Vegf family gene. Rn values are the summary of absolute values for all *R* values of top correlated genes. Ra is the average of Rn [1].

**Fig. 1:**
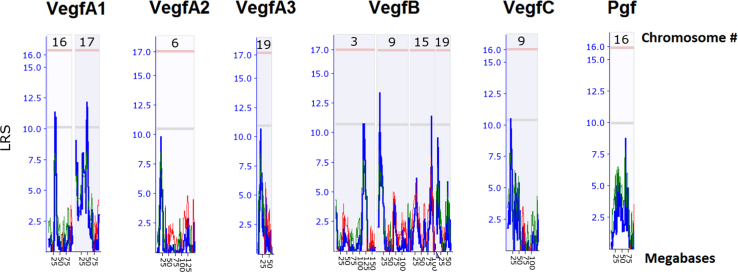
eQTL locations of Vegf genes on different chromosomes.

**Fig. 2:**
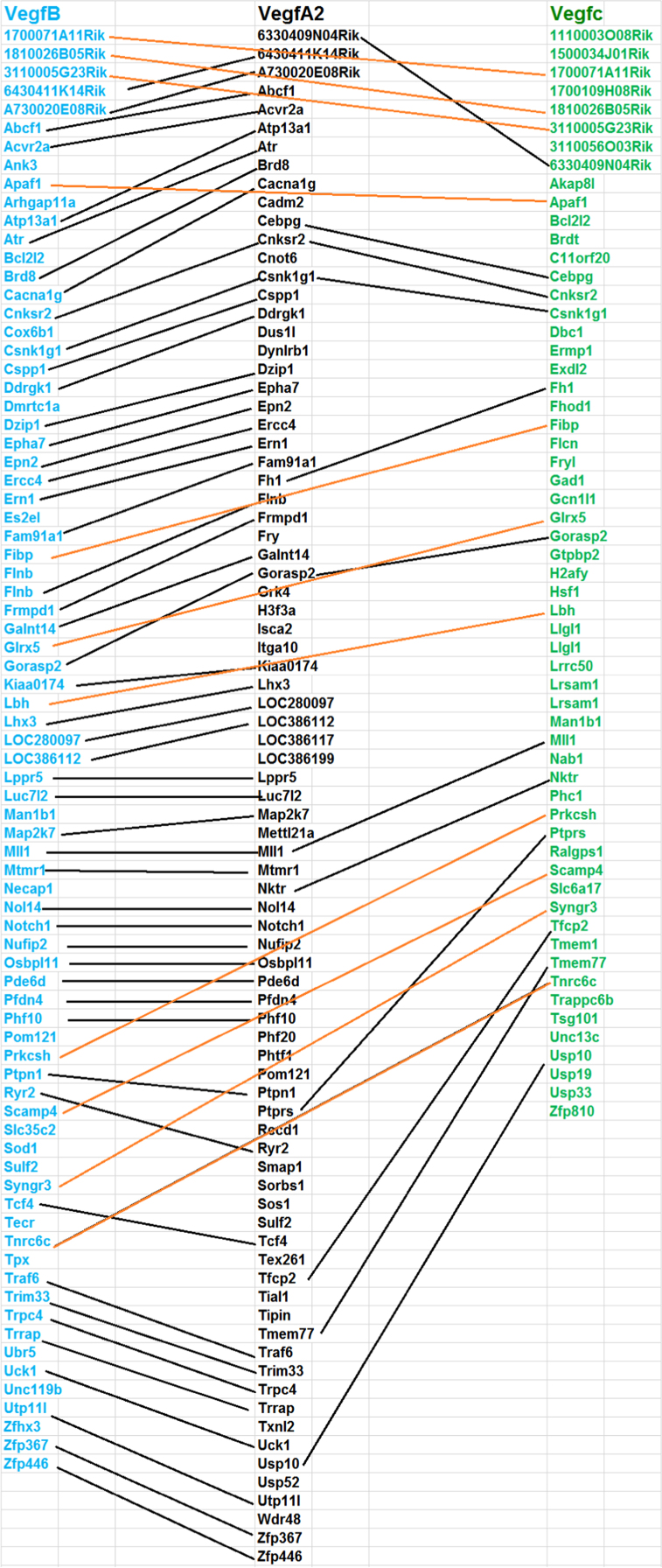
Overlapped genes from top 500 most correlated genes among VegfA, VegfB and VegfC. A total of 49 overlapped genes between the top correlates of VegfA2 and VegfB, the most among these probes. VegfC had a similar number of overlapped genes with VegfA2 and VegfB, with 11 and 12, respectively. The top 500 from each gene are colored in Blue (their expression levels were most correlated to that of VegfB), black (most correlated to that VegfA2) and green (VegfC). The overlapped genes among these gene sets are linked with dashed lines. (Most of unoverlapped genes from each set has been deleted so that the name of hte overlapped genes can be standed out and figure can be presented appropriately).

**Fig. 3:**
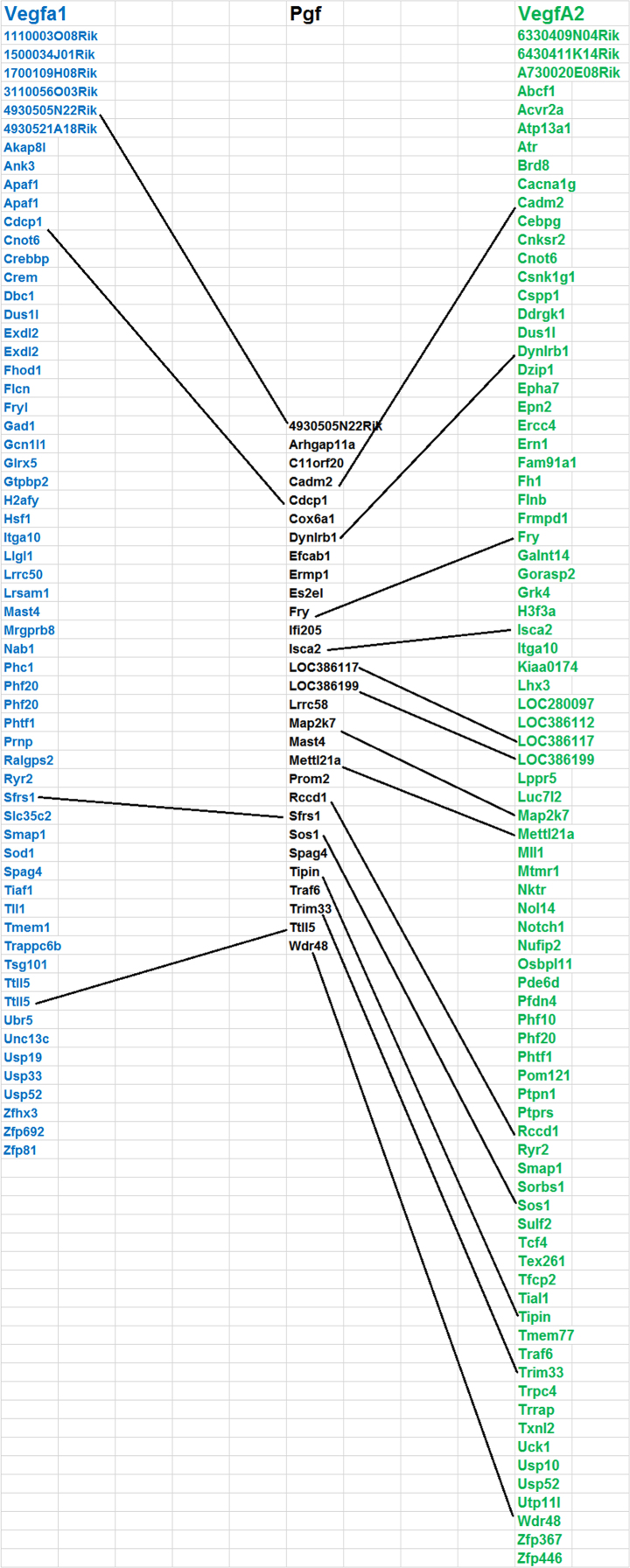
Overlapped genes from top 500 genes among VegfA1 and VegfA2 and Pgf. Pgf had 13 overlapped genes with VegfA2, 4 with VegfA1. The top 500 from each gene are colored in Blue (their expression levels were most correlated to that of VegfA1), black (most correlated to that Pgf) and green (VegfA2). The overlapped genes among these gene sets are linked wtih dashed lines. (Most of unoverlapped genes from each set have has been deleted so that the name of the overlapped genes can be standed out and figure can be physically presented appropriately).

**Fig. 4:**
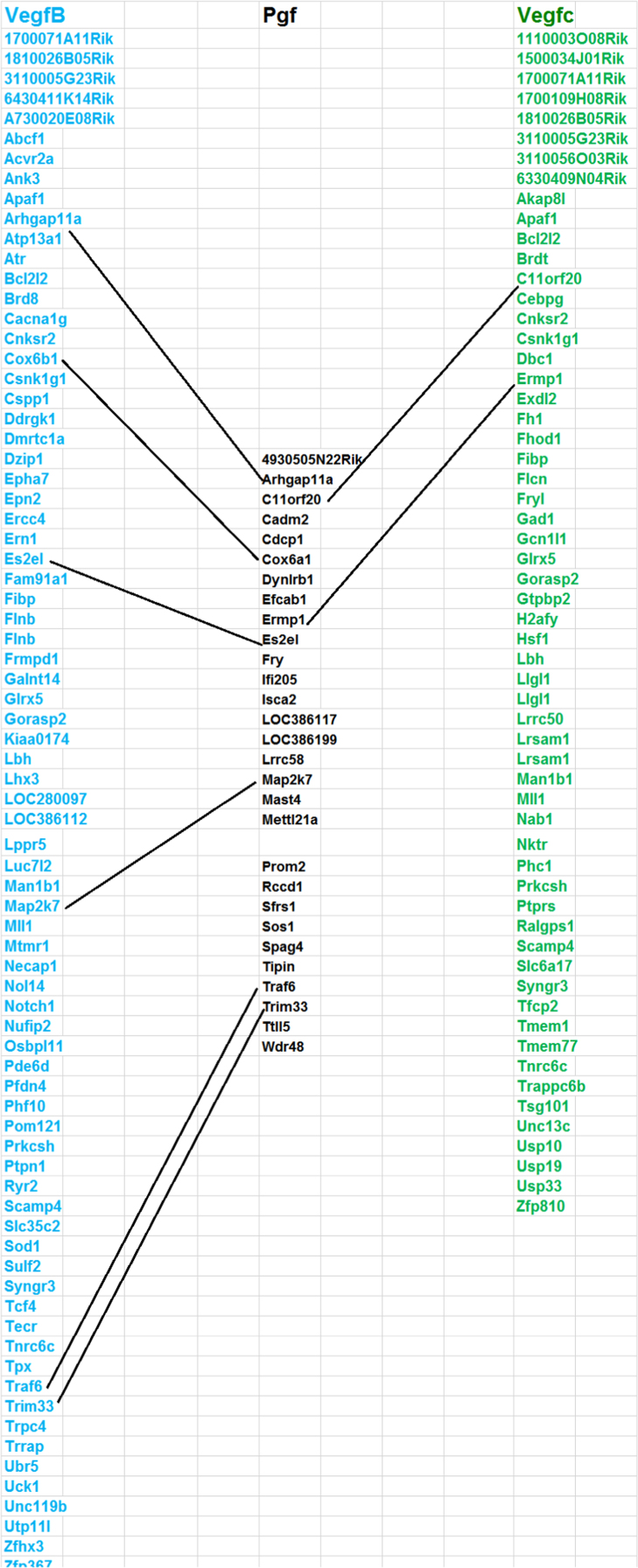
Overlapped genes among VegfB, VegfC and Pgf. Pgf. had 6 and 2 overlaps with VegfB and VegfC. The top 500 from each gene are colored with Blue (their expression levels were most correlated to that of VegfB), black (most correlated to that Pgf) and green (VegfC). The overlapped genes among these gene sets are linked wtih dashed lines. (Most of unoverlapped genes from each set has been deleted so that the name of hte overlapped genes can be standed out and figure can be presented appropriately).

## 2 Experimental designs, materials, and methods

### 2.1 Mapping expression quantitative trait loci of Vegf family genes

The gene expression data and analytic tools of the data were from GeneNetwork. For each probe of the gene the genomic region(s) or expression quantitative trait loci (eQTL) that regulate its expression level was mapped with the GeneNetwork tool. Interval mapping with 1000 permutation tests was conducted to locate the eQTL. A 2000 Bootstrap test was conducted to estimate the suggestive and significant linkage scores. The genomic region of each eQTL was compared to that of other probes [2].

#### 2.1.1 Obtaining the top genes which are closely related to the expression levels of Vegf genes

In the GeneNetwork platform, firstly identify the data of retina and data sets of Normal HEI Retina from the mouse species and then by typing the gene names in the “Get Any” column and click the “Search”, the Vegf genes were obtained. After that, click the names of each gene to get into a new link page of the gene clicked. In the new link page of the gene of interest, click the “calculation correlation”, and choose either 50 or 500 and then “pearson” as the analytic method. By clicking the “Compute” command, the top related genes were obtained [3].

#### 2.1.2 Comparing the correlation of top 50 genes of one gene with another different gene within the VEgf family

After the top 50 genes which were closely related to one of the Vegf genes were “selected” and collected by clicking the “Add” command, and the other genes from Vegf family were also obtained and “added” to the collection list, all above genes would appear in the page of “BXD Trait collection”. The correlations between the top 50 genes from one Vegf gene and other genes then were obtained by “selecting” all of them and clicking the “Metrix” command (Supplementary Table 1).

#### 2.1.3 Identifying the overlapped genes among top 500 genes that are closely related to different genes in Vegf family

After the top 500 genes for each of the gene in the Vegf family were obtained, uploading them into the Excel work sheet and then comparing the names of each gene in this top 500 genes which were most closely related to one Vegf gene with the 500 genes in another different Vegf gene. The gene symbols that matched exactly with each other would be marked as same between these two 500 sets. These genes have been linked together in the figure of the gene list to show their identity.

### 2.2 Data characterization and analysis

#### 2.2.1 The eQTLs that regulate the expression levels of VegfA, VegfB, VegfC and Pgf in mouse retina

The eQTL of each probe of gene in the Vegf family was mapped and examined. Overall, there was no eQTL with a score that reaches to the significant level. A total of 10 eQTL exactly at or closed to a suggestive level were identified (Fig. 1) in which four were identified from the VegfB probe, two were from the VegfA1 probe and one was identified from each of the other four probes. The genomic regions that contain genes for the regulation of expression levels of these probes are different from each other, except for VegfB and VegfC, which may be regulated by the same locus on chromosome 9 (Fig. 1). This information indicated a complex regulatory mechanism of expression levels among these genes in the Vegf family.
