# Supplementary material for: Data sets of eQTL loci, correlation analysis, and overlapped genes among gene sets that their expression levels are closely related to genes of Vegf family
Source: Data Brief. 2018 Sep 8;20:1854–60. doi: 10.1016/j.dib.2018.09.004 (PMC6169370; doi:10.1016/j.dib.2018.09.004)
Supplement: Supplementary file 2 — Supplementary material [file mmc2.docx]

Supplementary Table 1. Top 50 genes from Vegfb and correlations with probes of other genes.

Lower left cells list Pearson product-moment correlations; upper right cells list Spearman rank order correlations. Each cell also contains the n of cases. Values higher than 0.7 are displayed in red; those between 0.5 and 0.7 in orange; Values lower than -0.7 are in blue; between -0.5 and -0.7 in green. Select any cell to generate a scatter plot. Select trait labels for more information.

|  | Spearman Rank Correlation (rho) | | | | | | | | | | | | | | | | | | | | | | | | | | | | | | | | | | | | | | | | | | | | | | | | | | | | | | | | |
| --- | --- | --- | --- | --- | --- | --- | --- | --- | --- | --- | --- | --- | --- | --- | --- | --- | --- | --- | --- | --- | --- | --- | --- | --- | --- | --- | --- | --- | --- | --- | --- | --- | --- | --- | --- | --- | --- | --- | --- | --- | --- | --- | --- | --- | --- | --- | --- | --- | --- | --- | --- | --- | --- | --- | --- | --- | --- |
| P e a r s o n     r |  | [**Trait1**](javascript:showDatabase2('Illum_Retina_BXD_RankInv0410','ILMN_2450384','');) | [**Trait2**](javascript:showDatabase2('Illum_Retina_BXD_RankInv0410','ILMN_3068754','');) | [**Trait3**](javascript:showDatabase2('Illum_Retina_BXD_RankInv0410','ILMN_2677092','');) | [**Trait4**](javascript:showDatabase2('Illum_Retina_BXD_RankInv0410','ILMN_2624938','');) | [**Trait5**](javascript:showDatabase2('Illum_Retina_BXD_RankInv0410','ILMN_2847269','');) | [**Trait6**](javascript:showDatabase2('Illum_Retina_BXD_RankInv0410','ILMN_2839682','');) | [**Trait7**](javascript:showDatabase2('Illum_Retina_BXD_RankInv0410','ILMN_3147135','');) | [**Trait8**](javascript:showDatabase2('Illum_Retina_BXD_RankInv0410','ILMN_1255823','');) | [**Trait9**](javascript:showDatabase2('Illum_Retina_BXD_RankInv0410','ILMN_2626143','');) | [**Trait10**](javascript:showDatabase2('Illum_Retina_BXD_RankInv0410','ILMN_1234930','');) | [**Trait11**](javascript:showDatabase2('Illum_Retina_BXD_RankInv0410','ILMN_1249408','');) | [**Trait12**](javascript:showDatabase2('Illum_Retina_BXD_RankInv0410','ILMN_2971559','');) | [**Trait13**](javascript:showDatabase2('Illum_Retina_BXD_RankInv0410','ILMN_1258455','');) | [**Trait14**](javascript:showDatabase2('Illum_Retina_BXD_RankInv0410','ILMN_2691613','');) | [**Trait15**](javascript:showDatabase2('Illum_Retina_BXD_RankInv0410','ILMN_1253600','');) | [**Trait16**](javascript:showDatabase2('Illum_Retina_BXD_RankInv0410','ILMN_2991545','');) | [**Trait17**](javascript:showDatabase2('Illum_Retina_BXD_RankInv0410','ILMN_2623591','');) | [**Trait18**](javascript:showDatabase2('Illum_Retina_BXD_RankInv0410','ILMN_2603568','');) | [**Trait19**](javascript:showDatabase2('Illum_Retina_BXD_RankInv0410','ILMN_2992541','');) | [**Trait20**](javascript:showDatabase2('Illum_Retina_BXD_RankInv0410','ILMN_2792868','');) | [**Trait21**](javascript:showDatabase2('Illum_Retina_BXD_RankInv0410','ILMN_3129497','');) | [**Trait22**](javascript:showDatabase2('Illum_Retina_BXD_RankInv0410','ILMN_1224034','');) | [**Trait23**](javascript:showDatabase2('Illum_Retina_BXD_RankInv0410','ILMN_2639819','');) | [**Trait24**](javascript:showDatabase2('Illum_Retina_BXD_RankInv0410','ILMN_2994779','');) | [**Trait25**](javascript:showDatabase2('Illum_Retina_BXD_RankInv0410','ILMN_2625047','');) | [**Trait26**](javascript:showDatabase2('Illum_Retina_BXD_RankInv0410','ILMN_2808186','');) | [**Trait27**](javascript:showDatabase2('Illum_Retina_BXD_RankInv0410','ILMN_2689056','');) | [**Trait28**](javascript:showDatabase2('Illum_Retina_BXD_RankInv0410','ILMN_2648386','');) | [**Trait29**](javascript:showDatabase2('Illum_Retina_BXD_RankInv0410','ILMN_2869225','');) | [**Trait30**](javascript:showDatabase2('Illum_Retina_BXD_RankInv0410','ILMN_2728431','');) | [**Trait31**](javascript:showDatabase2('Illum_Retina_BXD_RankInv0410','ILMN_3094608','');) | [**Trait32**](javascript:showDatabase2('Illum_Retina_BXD_RankInv0410','ILMN_2896528','');) | [**Trait33**](javascript:showDatabase2('Illum_Retina_BXD_RankInv0410','ILMN_2701750','');) | [**Trait34**](javascript:showDatabase2('Illum_Retina_BXD_RankInv0410','ILMN_1239448','');) | [**Trait35**](javascript:showDatabase2('Illum_Retina_BXD_RankInv0410','ILMN_2583882','');) | [**Trait36**](javascript:showDatabase2('Illum_Retina_BXD_RankInv0410','ILMN_2915166','');) | [**Trait37**](javascript:showDatabase2('Illum_Retina_BXD_RankInv0410','ILMN_2702997','');) | [**Trait38**](javascript:showDatabase2('Illum_Retina_BXD_RankInv0410','ILMN_2435206','');) | [**Trait39**](javascript:showDatabase2('Illum_Retina_BXD_RankInv0410','ILMN_2789888','');) | [**Trait40**](javascript:showDatabase2('Illum_Retina_BXD_RankInv0410','ILMN_1218901','');) | [**Trait41**](javascript:showDatabase2('Illum_Retina_BXD_RankInv0410','ILMN_1254734','');) | [**Trait42**](javascript:showDatabase2('Illum_Retina_BXD_RankInv0410','ILMN_3008924','');) | [**Trait43**](javascript:showDatabase2('Illum_Retina_BXD_RankInv0410','ILMN_2628175','');) | [**Trait44**](javascript:showDatabase2('Illum_Retina_BXD_RankInv0410','ILMN_2891573','');) | [**Trait45**](javascript:showDatabase2('Illum_Retina_BXD_RankInv0410','ILMN_1220846','');) | [**Trait46**](javascript:showDatabase2('Illum_Retina_BXD_RankInv0410','ILMN_1241962','');) | [**Trait47**](javascript:showDatabase2('Illum_Retina_BXD_RankInv0410','ILMN_3163027','');) | [**Trait48**](javascript:showDatabase2('Illum_Retina_BXD_RankInv0410','ILMN_2775098','');) | [**Trait49**](javascript:showDatabase2('Illum_Retina_BXD_RankInv0410','ILMN_2752552','');) | [**Trait50**](javascript:showDatabase2('Illum_Retina_BXD_RankInv0410','ILMN_2730293','');) | [**Trait51**](javascript:showDatabase2('Illum_Retina_BXD_RankInv0410','ILMN_2680872','');) | [**Trait52**](javascript:showDatabase2('Illum_Retina_BXD_RankInv0410','ILMN_2768533','');) | [**Trait53**](javascript:showDatabase2('Illum_Retina_BXD_RankInv0410','ILMN_3121255','');) | [**Trait54**](javascript:showDatabase2('Illum_Retina_BXD_RankInv0410','ILMN_2484527','');) | [**Trait55**](javascript:showDatabase2('Illum_Retina_BXD_RankInv0410','ILMN_3045723','');) | [**Trait56**](javascript:showDatabase2('Illum_Retina_BXD_RankInv0410','ILMN_2486573','');) |
|  | [**Trait 1::ILMN_2450384**](javascript:showDatabase2('Illum_Retina_BXD_RankInv0410','ILMN_2450384','');)  Symbol: Vegfb  Vegfb | [***n* 79**](javascript:showDatabase2('Illum_Retina_BXD_RankInv0410','ILMN_2450384','')) | [**0.756 79**](javascript:showCorrelationPlot2(db='Illum_Retina_BXD_RankInv0410',ProbeSetID='ILMN_2450384',CellID='',db2='Illum_Retina_BXD_RankInv0410',ProbeSetID2='ILMN_3068754',CellID2='',rank='1')) | [**0.744 79**](javascript:showCorrelationPlot2(db='Illum_Retina_BXD_RankInv0410',ProbeSetID='ILMN_2450384',CellID='',db2='Illum_Retina_BXD_RankInv0410',ProbeSetID2='ILMN_2677092',CellID2='',rank='1')) | [**0.718 79**](javascript:showCorrelationPlot2(db='Illum_Retina_BXD_RankInv0410',ProbeSetID='ILMN_2450384',CellID='',db2='Illum_Retina_BXD_RankInv0410',ProbeSetID2='ILMN_2624938',CellID2='',rank='1')) | [**0.719 79**](javascript:showCorrelationPlot2(db='Illum_Retina_BXD_RankInv0410',ProbeSetID='ILMN_2450384',CellID='',db2='Illum_Retina_BXD_RankInv0410',ProbeSetID2='ILMN_2847269',CellID2='',rank='1')) | [**0.670 79**](javascript:showCorrelationPlot2(db='Illum_Retina_BXD_RankInv0410',ProbeSetID='ILMN_2450384',CellID='',db2='Illum_Retina_BXD_RankInv0410',ProbeSetID2='ILMN_2839682',CellID2='',rank='1')) | [**0.701 79**](javascript:showCorrelationPlot2(db='Illum_Retina_BXD_RankInv0410',ProbeSetID='ILMN_2450384',CellID='',db2='Illum_Retina_BXD_RankInv0410',ProbeSetID2='ILMN_3147135',CellID2='',rank='1')) | [**0.702 79**](javascript:showCorrelationPlot2(db='Illum_Retina_BXD_RankInv0410',ProbeSetID='ILMN_2450384',CellID='',db2='Illum_Retina_BXD_RankInv0410',ProbeSetID2='ILMN_1255823',CellID2='',rank='1')) | [**0.677 79**](javascript:showCorrelationPlot2(db='Illum_Retina_BXD_RankInv0410',ProbeSetID='ILMN_2450384',CellID='',db2='Illum_Retina_BXD_RankInv0410',ProbeSetID2='ILMN_2626143',CellID2='',rank='1')) | [**0.666 79**](javascript:showCorrelationPlot2(db='Illum_Retina_BXD_RankInv0410',ProbeSetID='ILMN_2450384',CellID='',db2='Illum_Retina_BXD_RankInv0410',ProbeSetID2='ILMN_1234930',CellID2='',rank='1')) | [**0.711 79**](javascript:showCorrelationPlot2(db='Illum_Retina_BXD_RankInv0410',ProbeSetID='ILMN_2450384',CellID='',db2='Illum_Retina_BXD_RankInv0410',ProbeSetID2='ILMN_1249408',CellID2='',rank='1')) | [**0.676 79**](javascript:showCorrelationPlot2(db='Illum_Retina_BXD_RankInv0410',ProbeSetID='ILMN_2450384',CellID='',db2='Illum_Retina_BXD_RankInv0410',ProbeSetID2='ILMN_2971559',CellID2='',rank='1')) | [**0.699 79**](javascript:showCorrelationPlot2(db='Illum_Retina_BXD_RankInv0410',ProbeSetID='ILMN_2450384',CellID='',db2='Illum_Retina_BXD_RankInv0410',ProbeSetID2='ILMN_1258455',CellID2='',rank='1')) | [**0.671 79**](javascript:showCorrelationPlot2(db='Illum_Retina_BXD_RankInv0410',ProbeSetID='ILMN_2450384',CellID='',db2='Illum_Retina_BXD_RankInv0410',ProbeSetID2='ILMN_2691613',CellID2='',rank='1')) | [**0.700 79**](javascript:showCorrelationPlot2(db='Illum_Retina_BXD_RankInv0410',ProbeSetID='ILMN_2450384',CellID='',db2='Illum_Retina_BXD_RankInv0410',ProbeSetID2='ILMN_1253600',CellID2='',rank='1')) | [**0.627 79**](javascript:showCorrelationPlot2(db='Illum_Retina_BXD_RankInv0410',ProbeSetID='ILMN_2450384',CellID='',db2='Illum_Retina_BXD_RankInv0410',ProbeSetID2='ILMN_2991545',CellID2='',rank='1')) | [**0.686 79**](javascript:showCorrelationPlot2(db='Illum_Retina_BXD_RankInv0410',ProbeSetID='ILMN_2450384',CellID='',db2='Illum_Retina_BXD_RankInv0410',ProbeSetID2='ILMN_2623591',CellID2='',rank='1')) | [**0.662 79**](javascript:showCorrelationPlot2(db='Illum_Retina_BXD_RankInv0410',ProbeSetID='ILMN_2450384',CellID='',db2='Illum_Retina_BXD_RankInv0410',ProbeSetID2='ILMN_2603568',CellID2='',rank='1')) | [**0.642 79**](javascript:showCorrelationPlot2(db='Illum_Retina_BXD_RankInv0410',ProbeSetID='ILMN_2450384',CellID='',db2='Illum_Retina_BXD_RankInv0410',ProbeSetID2='ILMN_2992541',CellID2='',rank='1')) | [**0.664 79**](javascript:showCorrelationPlot2(db='Illum_Retina_BXD_RankInv0410',ProbeSetID='ILMN_2450384',CellID='',db2='Illum_Retina_BXD_RankInv0410',ProbeSetID2='ILMN_2792868',CellID2='',rank='1')) | [**0.710 79**](javascript:showCorrelationPlot2(db='Illum_Retina_BXD_RankInv0410',ProbeSetID='ILMN_2450384',CellID='',db2='Illum_Retina_BXD_RankInv0410',ProbeSetID2='ILMN_3129497',CellID2='',rank='1')) | [**0.710 79**](javascript:showCorrelationPlot2(db='Illum_Retina_BXD_RankInv0410',ProbeSetID='ILMN_2450384',CellID='',db2='Illum_Retina_BXD_RankInv0410',ProbeSetID2='ILMN_1224034',CellID2='',rank='1')) | [**0.651 79**](javascript:showCorrelationPlot2(db='Illum_Retina_BXD_RankInv0410',ProbeSetID='ILMN_2450384',CellID='',db2='Illum_Retina_BXD_RankInv0410',ProbeSetID2='ILMN_2639819',CellID2='',rank='1')) | [**0.624 79**](javascript:showCorrelationPlot2(db='Illum_Retina_BXD_RankInv0410',ProbeSetID='ILMN_2450384',CellID='',db2='Illum_Retina_BXD_RankInv0410',ProbeSetID2='ILMN_2994779',CellID2='',rank='1')) | [**0.675 79**](javascript:showCorrelationPlot2(db='Illum_Retina_BXD_RankInv0410',ProbeSetID='ILMN_2450384',CellID='',db2='Illum_Retina_BXD_RankInv0410',ProbeSetID2='ILMN_2625047',CellID2='',rank='1')) | [**0.654 79**](javascript:showCorrelationPlot2(db='Illum_Retina_BXD_RankInv0410',ProbeSetID='ILMN_2450384',CellID='',db2='Illum_Retina_BXD_RankInv0410',ProbeSetID2='ILMN_2808186',CellID2='',rank='1')) | [**0.614 79**](javascript:showCorrelationPlot2(db='Illum_Retina_BXD_RankInv0410',ProbeSetID='ILMN_2450384',CellID='',db2='Illum_Retina_BXD_RankInv0410',ProbeSetID2='ILMN_2689056',CellID2='',rank='1')) | [**0.651 79**](javascript:showCorrelationPlot2(db='Illum_Retina_BXD_RankInv0410',ProbeSetID='ILMN_2450384',CellID='',db2='Illum_Retina_BXD_RankInv0410',ProbeSetID2='ILMN_2648386',CellID2='',rank='1')) | [**0.654 79**](javascript:showCorrelationPlot2(db='Illum_Retina_BXD_RankInv0410',ProbeSetID='ILMN_2450384',CellID='',db2='Illum_Retina_BXD_RankInv0410',ProbeSetID2='ILMN_2869225',CellID2='',rank='1')) | [**0.659 79**](javascript:showCorrelationPlot2(db='Illum_Retina_BXD_RankInv0410',ProbeSetID='ILMN_2450384',CellID='',db2='Illum_Retina_BXD_RankInv0410',ProbeSetID2='ILMN_2728431',CellID2='',rank='1')) | [**0.625 79**](javascript:showCorrelationPlot2(db='Illum_Retina_BXD_RankInv0410',ProbeSetID='ILMN_2450384',CellID='',db2='Illum_Retina_BXD_RankInv0410',ProbeSetID2='ILMN_3094608',CellID2='',rank='1')) | [**0.663 79**](javascript:showCorrelationPlot2(db='Illum_Retina_BXD_RankInv0410',ProbeSetID='ILMN_2450384',CellID='',db2='Illum_Retina_BXD_RankInv0410',ProbeSetID2='ILMN_2896528',CellID2='',rank='1')) | [**0.663 79**](javascript:showCorrelationPlot2(db='Illum_Retina_BXD_RankInv0410',ProbeSetID='ILMN_2450384',CellID='',db2='Illum_Retina_BXD_RankInv0410',ProbeSetID2='ILMN_2701750',CellID2='',rank='1')) | [**0.631 79**](javascript:showCorrelationPlot2(db='Illum_Retina_BXD_RankInv0410',ProbeSetID='ILMN_2450384',CellID='',db2='Illum_Retina_BXD_RankInv0410',ProbeSetID2='ILMN_1239448',CellID2='',rank='1')) | [**-0.614 79**](javascript:showCorrelationPlot2(db='Illum_Retina_BXD_RankInv0410',ProbeSetID='ILMN_2450384',CellID='',db2='Illum_Retina_BXD_RankInv0410',ProbeSetID2='ILMN_2583882',CellID2='',rank='1')) | [**0.637 79**](javascript:showCorrelationPlot2(db='Illum_Retina_BXD_RankInv0410',ProbeSetID='ILMN_2450384',CellID='',db2='Illum_Retina_BXD_RankInv0410',ProbeSetID2='ILMN_2915166',CellID2='',rank='1')) | [**0.651 79**](javascript:showCorrelationPlot2(db='Illum_Retina_BXD_RankInv0410',ProbeSetID='ILMN_2450384',CellID='',db2='Illum_Retina_BXD_RankInv0410',ProbeSetID2='ILMN_2702997',CellID2='',rank='1')) | [**0.637 79**](javascript:showCorrelationPlot2(db='Illum_Retina_BXD_RankInv0410',ProbeSetID='ILMN_2450384',CellID='',db2='Illum_Retina_BXD_RankInv0410',ProbeSetID2='ILMN_2435206',CellID2='',rank='1')) | [**0.647 79**](javascript:showCorrelationPlot2(db='Illum_Retina_BXD_RankInv0410',ProbeSetID='ILMN_2450384',CellID='',db2='Illum_Retina_BXD_RankInv0410',ProbeSetID2='ILMN_2789888',CellID2='',rank='1')) | [**0.604 79**](javascript:showCorrelationPlot2(db='Illum_Retina_BXD_RankInv0410',ProbeSetID='ILMN_2450384',CellID='',db2='Illum_Retina_BXD_RankInv0410',ProbeSetID2='ILMN_1218901',CellID2='',rank='1')) | [**0.622 79**](javascript:showCorrelationPlot2(db='Illum_Retina_BXD_RankInv0410',ProbeSetID='ILMN_2450384',CellID='',db2='Illum_Retina_BXD_RankInv0410',ProbeSetID2='ILMN_1254734',CellID2='',rank='1')) | [**0.569 79**](javascript:showCorrelationPlot2(db='Illum_Retina_BXD_RankInv0410',ProbeSetID='ILMN_2450384',CellID='',db2='Illum_Retina_BXD_RankInv0410',ProbeSetID2='ILMN_3008924',CellID2='',rank='1')) | [**0.634 79**](javascript:showCorrelationPlot2(db='Illum_Retina_BXD_RankInv0410',ProbeSetID='ILMN_2450384',CellID='',db2='Illum_Retina_BXD_RankInv0410',ProbeSetID2='ILMN_2628175',CellID2='',rank='1')) | [**0.589 79**](javascript:showCorrelationPlot2(db='Illum_Retina_BXD_RankInv0410',ProbeSetID='ILMN_2450384',CellID='',db2='Illum_Retina_BXD_RankInv0410',ProbeSetID2='ILMN_2891573',CellID2='',rank='1')) | [**0.634 79**](javascript:showCorrelationPlot2(db='Illum_Retina_BXD_RankInv0410',ProbeSetID='ILMN_2450384',CellID='',db2='Illum_Retina_BXD_RankInv0410',ProbeSetID2='ILMN_1220846',CellID2='',rank='1')) | [**0.633 79**](javascript:showCorrelationPlot2(db='Illum_Retina_BXD_RankInv0410',ProbeSetID='ILMN_2450384',CellID='',db2='Illum_Retina_BXD_RankInv0410',ProbeSetID2='ILMN_1241962',CellID2='',rank='1')) | [**0.628 79**](javascript:showCorrelationPlot2(db='Illum_Retina_BXD_RankInv0410',ProbeSetID='ILMN_2450384',CellID='',db2='Illum_Retina_BXD_RankInv0410',ProbeSetID2='ILMN_3163027',CellID2='',rank='1')) | [**0.612 79**](javascript:showCorrelationPlot2(db='Illum_Retina_BXD_RankInv0410',ProbeSetID='ILMN_2450384',CellID='',db2='Illum_Retina_BXD_RankInv0410',ProbeSetID2='ILMN_2775098',CellID2='',rank='1')) | [**0.643 79**](javascript:showCorrelationPlot2(db='Illum_Retina_BXD_RankInv0410',ProbeSetID='ILMN_2450384',CellID='',db2='Illum_Retina_BXD_RankInv0410',ProbeSetID2='ILMN_2752552',CellID2='',rank='1')) | [**0.696 79**](javascript:showCorrelationPlot2(db='Illum_Retina_BXD_RankInv0410',ProbeSetID='ILMN_2450384',CellID='',db2='Illum_Retina_BXD_RankInv0410',ProbeSetID2='ILMN_2730293',CellID2='',rank='1')) | [**0.610 79**](javascript:showCorrelationPlot2(db='Illum_Retina_BXD_RankInv0410',ProbeSetID='ILMN_2450384',CellID='',db2='Illum_Retina_BXD_RankInv0410',ProbeSetID2='ILMN_2680872',CellID2='',rank='1')) | [**0.228 79**](javascript:showCorrelationPlot2(db='Illum_Retina_BXD_RankInv0410',ProbeSetID='ILMN_2450384',CellID='',db2='Illum_Retina_BXD_RankInv0410',ProbeSetID2='ILMN_2768533',CellID2='',rank='1')) | [**-0.149 79**](javascript:showCorrelationPlot2(db='Illum_Retina_BXD_RankInv0410',ProbeSetID='ILMN_2450384',CellID='',db2='Illum_Retina_BXD_RankInv0410',ProbeSetID2='ILMN_3121255',CellID2='',rank='1')) | [**-0.275 79**](javascript:showCorrelationPlot2(db='Illum_Retina_BXD_RankInv0410',ProbeSetID='ILMN_2450384',CellID='',db2='Illum_Retina_BXD_RankInv0410',ProbeSetID2='ILMN_2484527',CellID2='',rank='1')) | [**0.194 79**](javascript:showCorrelationPlot2(db='Illum_Retina_BXD_RankInv0410',ProbeSetID='ILMN_2450384',CellID='',db2='Illum_Retina_BXD_RankInv0410',ProbeSetID2='ILMN_3045723',CellID2='',rank='1')) | [**0.360 79**](javascript:showCorrelationPlot2(db='Illum_Retina_BXD_RankInv0410',ProbeSetID='ILMN_2450384',CellID='',db2='Illum_Retina_BXD_RankInv0410',ProbeSetID2='ILMN_2486573',CellID2='',rank='1')) |
|  | [**Trait 2: ILMN_3068754**](javascript:showDatabase2('Illum_Retina_BXD_RankInv0410','ILMN_3068754','');)  Symbol: Akr1a4  Akr1a4 | [**0.784 79**](javascript:showCorrelationPlot2(db='Illum_Retina_BXD_RankInv0410',ProbeSetID='ILMN_3068754',CellID='',db2='Illum_Retina_BXD_RankInv0410',ProbeSetID2='ILMN_2450384',CellID2='',rank='0')) | [***n* 79**](javascript:showDatabase2('Illum_Retina_BXD_RankInv0410','ILMN_3068754','')) | [**0.685 79**](javascript:showCorrelationPlot2(db='Illum_Retina_BXD_RankInv0410',ProbeSetID='ILMN_3068754',CellID='',db2='Illum_Retina_BXD_RankInv0410',ProbeSetID2='ILMN_2677092',CellID2='',rank='1')) | [**0.667 79**](javascript:showCorrelationPlot2(db='Illum_Retina_BXD_RankInv0410',ProbeSetID='ILMN_3068754',CellID='',db2='Illum_Retina_BXD_RankInv0410',ProbeSetID2='ILMN_2624938',CellID2='',rank='1')) | [**0.548 79**](javascript:showCorrelationPlot2(db='Illum_Retina_BXD_RankInv0410',ProbeSetID='ILMN_3068754',CellID='',db2='Illum_Retina_BXD_RankInv0410',ProbeSetID2='ILMN_2847269',CellID2='',rank='1')) | [**0.740 79**](javascript:showCorrelationPlot2(db='Illum_Retina_BXD_RankInv0410',ProbeSetID='ILMN_3068754',CellID='',db2='Illum_Retina_BXD_RankInv0410',ProbeSetID2='ILMN_2839682',CellID2='',rank='1')) | [**0.664 79**](javascript:showCorrelationPlot2(db='Illum_Retina_BXD_RankInv0410',ProbeSetID='ILMN_3068754',CellID='',db2='Illum_Retina_BXD_RankInv0410',ProbeSetID2='ILMN_3147135',CellID2='',rank='1')) | [**0.562 79**](javascript:showCorrelationPlot2(db='Illum_Retina_BXD_RankInv0410',ProbeSetID='ILMN_3068754',CellID='',db2='Illum_Retina_BXD_RankInv0410',ProbeSetID2='ILMN_1255823',CellID2='',rank='1')) | [**0.667 79**](javascript:showCorrelationPlot2(db='Illum_Retina_BXD_RankInv0410',ProbeSetID='ILMN_3068754',CellID='',db2='Illum_Retina_BXD_RankInv0410',ProbeSetID2='ILMN_2626143',CellID2='',rank='1')) | [**0.646 79**](javascript:showCorrelationPlot2(db='Illum_Retina_BXD_RankInv0410',ProbeSetID='ILMN_3068754',CellID='',db2='Illum_Retina_BXD_RankInv0410',ProbeSetID2='ILMN_1234930',CellID2='',rank='1')) | [**0.579 79**](javascript:showCorrelationPlot2(db='Illum_Retina_BXD_RankInv0410',ProbeSetID='ILMN_3068754',CellID='',db2='Illum_Retina_BXD_RankInv0410',ProbeSetID2='ILMN_1249408',CellID2='',rank='1')) | [**0.568 79**](javascript:showCorrelationPlot2(db='Illum_Retina_BXD_RankInv0410',ProbeSetID='ILMN_3068754',CellID='',db2='Illum_Retina_BXD_RankInv0410',ProbeSetID2='ILMN_2971559',CellID2='',rank='1')) | [**0.547 79**](javascript:showCorrelationPlot2(db='Illum_Retina_BXD_RankInv0410',ProbeSetID='ILMN_3068754',CellID='',db2='Illum_Retina_BXD_RankInv0410',ProbeSetID2='ILMN_1258455',CellID2='',rank='1')) | [**0.601 79**](javascript:showCorrelationPlot2(db='Illum_Retina_BXD_RankInv0410',ProbeSetID='ILMN_3068754',CellID='',db2='Illum_Retina_BXD_RankInv0410',ProbeSetID2='ILMN_2691613',CellID2='',rank='1')) | [**0.674 79**](javascript:showCorrelationPlot2(db='Illum_Retina_BXD_RankInv0410',ProbeSetID='ILMN_3068754',CellID='',db2='Illum_Retina_BXD_RankInv0410',ProbeSetID2='ILMN_1253600',CellID2='',rank='1')) | [**0.602 79**](javascript:showCorrelationPlot2(db='Illum_Retina_BXD_RankInv0410',ProbeSetID='ILMN_3068754',CellID='',db2='Illum_Retina_BXD_RankInv0410',ProbeSetID2='ILMN_2991545',CellID2='',rank='1')) | [**0.740 79**](javascript:showCorrelationPlot2(db='Illum_Retina_BXD_RankInv0410',ProbeSetID='ILMN_3068754',CellID='',db2='Illum_Retina_BXD_RankInv0410',ProbeSetID2='ILMN_2623591',CellID2='',rank='1')) | [**0.590 79**](javascript:showCorrelationPlot2(db='Illum_Retina_BXD_RankInv0410',ProbeSetID='ILMN_3068754',CellID='',db2='Illum_Retina_BXD_RankInv0410',ProbeSetID2='ILMN_2603568',CellID2='',rank='1')) | [**0.546 79**](javascript:showCorrelationPlot2(db='Illum_Retina_BXD_RankInv0410',ProbeSetID='ILMN_3068754',CellID='',db2='Illum_Retina_BXD_RankInv0410',ProbeSetID2='ILMN_2992541',CellID2='',rank='1')) | [**0.412 79**](javascript:showCorrelationPlot2(db='Illum_Retina_BXD_RankInv0410',ProbeSetID='ILMN_3068754',CellID='',db2='Illum_Retina_BXD_RankInv0410',ProbeSetID2='ILMN_2792868',CellID2='',rank='1')) | [**0.579 79**](javascript:showCorrelationPlot2(db='Illum_Retina_BXD_RankInv0410',ProbeSetID='ILMN_3068754',CellID='',db2='Illum_Retina_BXD_RankInv0410',ProbeSetID2='ILMN_3129497',CellID2='',rank='1')) | [**0.550 79**](javascript:showCorrelationPlot2(db='Illum_Retina_BXD_RankInv0410',ProbeSetID='ILMN_3068754',CellID='',db2='Illum_Retina_BXD_RankInv0410',ProbeSetID2='ILMN_1224034',CellID2='',rank='1')) | [**0.677 79**](javascript:showCorrelationPlot2(db='Illum_Retina_BXD_RankInv0410',ProbeSetID='ILMN_3068754',CellID='',db2='Illum_Retina_BXD_RankInv0410',ProbeSetID2='ILMN_2639819',CellID2='',rank='1')) | [**0.449 79**](javascript:showCorrelationPlot2(db='Illum_Retina_BXD_RankInv0410',ProbeSetID='ILMN_3068754',CellID='',db2='Illum_Retina_BXD_RankInv0410',ProbeSetID2='ILMN_2994779',CellID2='',rank='1')) | [**0.568 79**](javascript:showCorrelationPlot2(db='Illum_Retina_BXD_RankInv0410',ProbeSetID='ILMN_3068754',CellID='',db2='Illum_Retina_BXD_RankInv0410',ProbeSetID2='ILMN_2625047',CellID2='',rank='1')) | [**0.585 79**](javascript:showCorrelationPlot2(db='Illum_Retina_BXD_RankInv0410',ProbeSetID='ILMN_3068754',CellID='',db2='Illum_Retina_BXD_RankInv0410',ProbeSetID2='ILMN_2808186',CellID2='',rank='1')) | [**0.762 79**](javascript:showCorrelationPlot2(db='Illum_Retina_BXD_RankInv0410',ProbeSetID='ILMN_3068754',CellID='',db2='Illum_Retina_BXD_RankInv0410',ProbeSetID2='ILMN_2689056',CellID2='',rank='1')) | [**0.624 79**](javascript:showCorrelationPlot2(db='Illum_Retina_BXD_RankInv0410',ProbeSetID='ILMN_3068754',CellID='',db2='Illum_Retina_BXD_RankInv0410',ProbeSetID2='ILMN_2648386',CellID2='',rank='1')) | [**0.528 79**](javascript:showCorrelationPlot2(db='Illum_Retina_BXD_RankInv0410',ProbeSetID='ILMN_3068754',CellID='',db2='Illum_Retina_BXD_RankInv0410',ProbeSetID2='ILMN_2869225',CellID2='',rank='1')) | [**0.524 79**](javascript:showCorrelationPlot2(db='Illum_Retina_BXD_RankInv0410',ProbeSetID='ILMN_3068754',CellID='',db2='Illum_Retina_BXD_RankInv0410',ProbeSetID2='ILMN_2728431',CellID2='',rank='1')) | [**0.757 79**](javascript:showCorrelationPlot2(db='Illum_Retina_BXD_RankInv0410',ProbeSetID='ILMN_3068754',CellID='',db2='Illum_Retina_BXD_RankInv0410',ProbeSetID2='ILMN_3094608',CellID2='',rank='1')) | [**0.397 79**](javascript:showCorrelationPlot2(db='Illum_Retina_BXD_RankInv0410',ProbeSetID='ILMN_3068754',CellID='',db2='Illum_Retina_BXD_RankInv0410',ProbeSetID2='ILMN_2896528',CellID2='',rank='1')) | [**0.502 79**](javascript:showCorrelationPlot2(db='Illum_Retina_BXD_RankInv0410',ProbeSetID='ILMN_3068754',CellID='',db2='Illum_Retina_BXD_RankInv0410',ProbeSetID2='ILMN_2701750',CellID2='',rank='1')) | [**0.681 79**](javascript:showCorrelationPlot2(db='Illum_Retina_BXD_RankInv0410',ProbeSetID='ILMN_3068754',CellID='',db2='Illum_Retina_BXD_RankInv0410',ProbeSetID2='ILMN_1239448',CellID2='',rank='1')) | [**-0.520 79**](javascript:showCorrelationPlot2(db='Illum_Retina_BXD_RankInv0410',ProbeSetID='ILMN_3068754',CellID='',db2='Illum_Retina_BXD_RankInv0410',ProbeSetID2='ILMN_2583882',CellID2='',rank='1')) | [**0.519 79**](javascript:showCorrelationPlot2(db='Illum_Retina_BXD_RankInv0410',ProbeSetID='ILMN_3068754',CellID='',db2='Illum_Retina_BXD_RankInv0410',ProbeSetID2='ILMN_2915166',CellID2='',rank='1')) | [**0.523 79**](javascript:showCorrelationPlot2(db='Illum_Retina_BXD_RankInv0410',ProbeSetID='ILMN_3068754',CellID='',db2='Illum_Retina_BXD_RankInv0410',ProbeSetID2='ILMN_2702997',CellID2='',rank='1')) | [**0.537 79**](javascript:showCorrelationPlot2(db='Illum_Retina_BXD_RankInv0410',ProbeSetID='ILMN_3068754',CellID='',db2='Illum_Retina_BXD_RankInv0410',ProbeSetID2='ILMN_2435206',CellID2='',rank='1')) | [**0.750 79**](javascript:showCorrelationPlot2(db='Illum_Retina_BXD_RankInv0410',ProbeSetID='ILMN_3068754',CellID='',db2='Illum_Retina_BXD_RankInv0410',ProbeSetID2='ILMN_2789888',CellID2='',rank='1')) | [**0.695 79**](javascript:showCorrelationPlot2(db='Illum_Retina_BXD_RankInv0410',ProbeSetID='ILMN_3068754',CellID='',db2='Illum_Retina_BXD_RankInv0410',ProbeSetID2='ILMN_1218901',CellID2='',rank='1')) | [**0.566 79**](javascript:showCorrelationPlot2(db='Illum_Retina_BXD_RankInv0410',ProbeSetID='ILMN_3068754',CellID='',db2='Illum_Retina_BXD_RankInv0410',ProbeSetID2='ILMN_1254734',CellID2='',rank='1')) | [**0.611 79**](javascript:showCorrelationPlot2(db='Illum_Retina_BXD_RankInv0410',ProbeSetID='ILMN_3068754',CellID='',db2='Illum_Retina_BXD_RankInv0410',ProbeSetID2='ILMN_3008924',CellID2='',rank='1')) | [**0.778 79**](javascript:showCorrelationPlot2(db='Illum_Retina_BXD_RankInv0410',ProbeSetID='ILMN_3068754',CellID='',db2='Illum_Retina_BXD_RankInv0410',ProbeSetID2='ILMN_2628175',CellID2='',rank='1')) | [**0.693 79**](javascript:showCorrelationPlot2(db='Illum_Retina_BXD_RankInv0410',ProbeSetID='ILMN_3068754',CellID='',db2='Illum_Retina_BXD_RankInv0410',ProbeSetID2='ILMN_2891573',CellID2='',rank='1')) | [**0.680 79**](javascript:showCorrelationPlot2(db='Illum_Retina_BXD_RankInv0410',ProbeSetID='ILMN_3068754',CellID='',db2='Illum_Retina_BXD_RankInv0410',ProbeSetID2='ILMN_1220846',CellID2='',rank='1')) | [**0.437 79**](javascript:showCorrelationPlot2(db='Illum_Retina_BXD_RankInv0410',ProbeSetID='ILMN_3068754',CellID='',db2='Illum_Retina_BXD_RankInv0410',ProbeSetID2='ILMN_1241962',CellID2='',rank='1')) | [**0.473 79**](javascript:showCorrelationPlot2(db='Illum_Retina_BXD_RankInv0410',ProbeSetID='ILMN_3068754',CellID='',db2='Illum_Retina_BXD_RankInv0410',ProbeSetID2='ILMN_3163027',CellID2='',rank='1')) | [**0.443 79**](javascript:showCorrelationPlot2(db='Illum_Retina_BXD_RankInv0410',ProbeSetID='ILMN_3068754',CellID='',db2='Illum_Retina_BXD_RankInv0410',ProbeSetID2='ILMN_2775098',CellID2='',rank='1')) | [**0.673 79**](javascript:showCorrelationPlot2(db='Illum_Retina_BXD_RankInv0410',ProbeSetID='ILMN_3068754',CellID='',db2='Illum_Retina_BXD_RankInv0410',ProbeSetID2='ILMN_2752552',CellID2='',rank='1')) | [**0.573 79**](javascript:showCorrelationPlot2(db='Illum_Retina_BXD_RankInv0410',ProbeSetID='ILMN_3068754',CellID='',db2='Illum_Retina_BXD_RankInv0410',ProbeSetID2='ILMN_2730293',CellID2='',rank='1')) | [**0.473 79**](javascript:showCorrelationPlot2(db='Illum_Retina_BXD_RankInv0410',ProbeSetID='ILMN_3068754',CellID='',db2='Illum_Retina_BXD_RankInv0410',ProbeSetID2='ILMN_2680872',CellID2='',rank='1')) | [**0.220 79**](javascript:showCorrelationPlot2(db='Illum_Retina_BXD_RankInv0410',ProbeSetID='ILMN_3068754',CellID='',db2='Illum_Retina_BXD_RankInv0410',ProbeSetID2='ILMN_2768533',CellID2='',rank='1')) | [**-0.345 79**](javascript:showCorrelationPlot2(db='Illum_Retina_BXD_RankInv0410',ProbeSetID='ILMN_3068754',CellID='',db2='Illum_Retina_BXD_RankInv0410',ProbeSetID2='ILMN_3121255',CellID2='',rank='1')) | [**-0.478 79**](javascript:showCorrelationPlot2(db='Illum_Retina_BXD_RankInv0410',ProbeSetID='ILMN_3068754',CellID='',db2='Illum_Retina_BXD_RankInv0410',ProbeSetID2='ILMN_2484527',CellID2='',rank='1')) | [**0.183 79**](javascript:showCorrelationPlot2(db='Illum_Retina_BXD_RankInv0410',ProbeSetID='ILMN_3068754',CellID='',db2='Illum_Retina_BXD_RankInv0410',ProbeSetID2='ILMN_3045723',CellID2='',rank='1')) | [**0.195 79**](javascript:showCorrelationPlot2(db='Illum_Retina_BXD_RankInv0410',ProbeSetID='ILMN_3068754',CellID='',db2='Illum_Retina_BXD_RankInv0410',ProbeSetID2='ILMN_2486573',CellID2='',rank='1')) |
|  | [**Trait 3: ILMN_2677092**](javascript:showDatabase2('Illum_Retina_BXD_RankInv0410','ILMN_2677092','');)  Symbol: Nutf2  Nutf2 | [**0.749 79**](javascript:showCorrelationPlot2(db='Illum_Retina_BXD_RankInv0410',ProbeSetID='ILMN_2677092',CellID='',db2='Illum_Retina_BXD_RankInv0410',ProbeSetID2='ILMN_2450384',CellID2='',rank='0')) | [**0.745 79**](javascript:showCorrelationPlot2(db='Illum_Retina_BXD_RankInv0410',ProbeSetID='ILMN_2677092',CellID='',db2='Illum_Retina_BXD_RankInv0410',ProbeSetID2='ILMN_3068754',CellID2='',rank='0')) | [***n* 79**](javascript:showDatabase2('Illum_Retina_BXD_RankInv0410','ILMN_2677092','')) | [**0.644 79**](javascript:showCorrelationPlot2(db='Illum_Retina_BXD_RankInv0410',ProbeSetID='ILMN_2677092',CellID='',db2='Illum_Retina_BXD_RankInv0410',ProbeSetID2='ILMN_2624938',CellID2='',rank='1')) | [**0.700 79**](javascript:showCorrelationPlot2(db='Illum_Retina_BXD_RankInv0410',ProbeSetID='ILMN_2677092',CellID='',db2='Illum_Retina_BXD_RankInv0410',ProbeSetID2='ILMN_2847269',CellID2='',rank='1')) | [**0.611 79**](javascript:showCorrelationPlot2(db='Illum_Retina_BXD_RankInv0410',ProbeSetID='ILMN_2677092',CellID='',db2='Illum_Retina_BXD_RankInv0410',ProbeSetID2='ILMN_2839682',CellID2='',rank='1')) | [**0.640 79**](javascript:showCorrelationPlot2(db='Illum_Retina_BXD_RankInv0410',ProbeSetID='ILMN_2677092',CellID='',db2='Illum_Retina_BXD_RankInv0410',ProbeSetID2='ILMN_3147135',CellID2='',rank='1')) | [**0.649 79**](javascript:showCorrelationPlot2(db='Illum_Retina_BXD_RankInv0410',ProbeSetID='ILMN_2677092',CellID='',db2='Illum_Retina_BXD_RankInv0410',ProbeSetID2='ILMN_1255823',CellID2='',rank='1')) | [**0.560 79**](javascript:showCorrelationPlot2(db='Illum_Retina_BXD_RankInv0410',ProbeSetID='ILMN_2677092',CellID='',db2='Illum_Retina_BXD_RankInv0410',ProbeSetID2='ILMN_2626143',CellID2='',rank='1')) | [**0.618 79**](javascript:showCorrelationPlot2(db='Illum_Retina_BXD_RankInv0410',ProbeSetID='ILMN_2677092',CellID='',db2='Illum_Retina_BXD_RankInv0410',ProbeSetID2='ILMN_1234930',CellID2='',rank='1')) | [**0.627 79**](javascript:showCorrelationPlot2(db='Illum_Retina_BXD_RankInv0410',ProbeSetID='ILMN_2677092',CellID='',db2='Illum_Retina_BXD_RankInv0410',ProbeSetID2='ILMN_1249408',CellID2='',rank='1')) | [**0.558 79**](javascript:showCorrelationPlot2(db='Illum_Retina_BXD_RankInv0410',ProbeSetID='ILMN_2677092',CellID='',db2='Illum_Retina_BXD_RankInv0410',ProbeSetID2='ILMN_2971559',CellID2='',rank='1')) | [**0.623 79**](javascript:showCorrelationPlot2(db='Illum_Retina_BXD_RankInv0410',ProbeSetID='ILMN_2677092',CellID='',db2='Illum_Retina_BXD_RankInv0410',ProbeSetID2='ILMN_1258455',CellID2='',rank='1')) | [**0.643 79**](javascript:showCorrelationPlot2(db='Illum_Retina_BXD_RankInv0410',ProbeSetID='ILMN_2677092',CellID='',db2='Illum_Retina_BXD_RankInv0410',ProbeSetID2='ILMN_2691613',CellID2='',rank='1')) | [**0.683 79**](javascript:showCorrelationPlot2(db='Illum_Retina_BXD_RankInv0410',ProbeSetID='ILMN_2677092',CellID='',db2='Illum_Retina_BXD_RankInv0410',ProbeSetID2='ILMN_1253600',CellID2='',rank='1')) | [**0.595 79**](javascript:showCorrelationPlot2(db='Illum_Retina_BXD_RankInv0410',ProbeSetID='ILMN_2677092',CellID='',db2='Illum_Retina_BXD_RankInv0410',ProbeSetID2='ILMN_2991545',CellID2='',rank='1')) | [**0.675 79**](javascript:showCorrelationPlot2(db='Illum_Retina_BXD_RankInv0410',ProbeSetID='ILMN_2677092',CellID='',db2='Illum_Retina_BXD_RankInv0410',ProbeSetID2='ILMN_2623591',CellID2='',rank='1')) | [**0.676 79**](javascript:showCorrelationPlot2(db='Illum_Retina_BXD_RankInv0410',ProbeSetID='ILMN_2677092',CellID='',db2='Illum_Retina_BXD_RankInv0410',ProbeSetID2='ILMN_2603568',CellID2='',rank='1')) | [**0.609 79**](javascript:showCorrelationPlot2(db='Illum_Retina_BXD_RankInv0410',ProbeSetID='ILMN_2677092',CellID='',db2='Illum_Retina_BXD_RankInv0410',ProbeSetID2='ILMN_2992541',CellID2='',rank='1')) | [**0.526 79**](javascript:showCorrelationPlot2(db='Illum_Retina_BXD_RankInv0410',ProbeSetID='ILMN_2677092',CellID='',db2='Illum_Retina_BXD_RankInv0410',ProbeSetID2='ILMN_2792868',CellID2='',rank='1')) | [**0.642 79**](javascript:showCorrelationPlot2(db='Illum_Retina_BXD_RankInv0410',ProbeSetID='ILMN_2677092',CellID='',db2='Illum_Retina_BXD_RankInv0410',ProbeSetID2='ILMN_3129497',CellID2='',rank='1')) | [**0.682 79**](javascript:showCorrelationPlot2(db='Illum_Retina_BXD_RankInv0410',ProbeSetID='ILMN_2677092',CellID='',db2='Illum_Retina_BXD_RankInv0410',ProbeSetID2='ILMN_1224034',CellID2='',rank='1')) | [**0.687 79**](javascript:showCorrelationPlot2(db='Illum_Retina_BXD_RankInv0410',ProbeSetID='ILMN_2677092',CellID='',db2='Illum_Retina_BXD_RankInv0410',ProbeSetID2='ILMN_2639819',CellID2='',rank='1')) | [**0.451 79**](javascript:showCorrelationPlot2(db='Illum_Retina_BXD_RankInv0410',ProbeSetID='ILMN_2677092',CellID='',db2='Illum_Retina_BXD_RankInv0410',ProbeSetID2='ILMN_2994779',CellID2='',rank='1')) | [**0.524 79**](javascript:showCorrelationPlot2(db='Illum_Retina_BXD_RankInv0410',ProbeSetID='ILMN_2677092',CellID='',db2='Illum_Retina_BXD_RankInv0410',ProbeSetID2='ILMN_2625047',CellID2='',rank='1')) | [**0.704 79**](javascript:showCorrelationPlot2(db='Illum_Retina_BXD_RankInv0410',ProbeSetID='ILMN_2677092',CellID='',db2='Illum_Retina_BXD_RankInv0410',ProbeSetID2='ILMN_2808186',CellID2='',rank='1')) | [**0.647 79**](javascript:showCorrelationPlot2(db='Illum_Retina_BXD_RankInv0410',ProbeSetID='ILMN_2677092',CellID='',db2='Illum_Retina_BXD_RankInv0410',ProbeSetID2='ILMN_2689056',CellID2='',rank='1')) | [**0.637 79**](javascript:showCorrelationPlot2(db='Illum_Retina_BXD_RankInv0410',ProbeSetID='ILMN_2677092',CellID='',db2='Illum_Retina_BXD_RankInv0410',ProbeSetID2='ILMN_2648386',CellID2='',rank='1')) | [**0.538 79**](javascript:showCorrelationPlot2(db='Illum_Retina_BXD_RankInv0410',ProbeSetID='ILMN_2677092',CellID='',db2='Illum_Retina_BXD_RankInv0410',ProbeSetID2='ILMN_2869225',CellID2='',rank='1')) | [**0.553 79**](javascript:showCorrelationPlot2(db='Illum_Retina_BXD_RankInv0410',ProbeSetID='ILMN_2677092',CellID='',db2='Illum_Retina_BXD_RankInv0410',ProbeSetID2='ILMN_2728431',CellID2='',rank='1')) | [**0.608 79**](javascript:showCorrelationPlot2(db='Illum_Retina_BXD_RankInv0410',ProbeSetID='ILMN_2677092',CellID='',db2='Illum_Retina_BXD_RankInv0410',ProbeSetID2='ILMN_3094608',CellID2='',rank='1')) | [**0.417 79**](javascript:showCorrelationPlot2(db='Illum_Retina_BXD_RankInv0410',ProbeSetID='ILMN_2677092',CellID='',db2='Illum_Retina_BXD_RankInv0410',ProbeSetID2='ILMN_2896528',CellID2='',rank='1')) | [**0.591 79**](javascript:showCorrelationPlot2(db='Illum_Retina_BXD_RankInv0410',ProbeSetID='ILMN_2677092',CellID='',db2='Illum_Retina_BXD_RankInv0410',ProbeSetID2='ILMN_2701750',CellID2='',rank='1')) | [**0.454 79**](javascript:showCorrelationPlot2(db='Illum_Retina_BXD_RankInv0410',ProbeSetID='ILMN_2677092',CellID='',db2='Illum_Retina_BXD_RankInv0410',ProbeSetID2='ILMN_1239448',CellID2='',rank='1')) | [**-0.615 79**](javascript:showCorrelationPlot2(db='Illum_Retina_BXD_RankInv0410',ProbeSetID='ILMN_2677092',CellID='',db2='Illum_Retina_BXD_RankInv0410',ProbeSetID2='ILMN_2583882',CellID2='',rank='1')) | [**0.557 79**](javascript:showCorrelationPlot2(db='Illum_Retina_BXD_RankInv0410',ProbeSetID='ILMN_2677092',CellID='',db2='Illum_Retina_BXD_RankInv0410',ProbeSetID2='ILMN_2915166',CellID2='',rank='1')) | [**0.570 79**](javascript:showCorrelationPlot2(db='Illum_Retina_BXD_RankInv0410',ProbeSetID='ILMN_2677092',CellID='',db2='Illum_Retina_BXD_RankInv0410',ProbeSetID2='ILMN_2702997',CellID2='',rank='1')) | [**0.527 79**](javascript:showCorrelationPlot2(db='Illum_Retina_BXD_RankInv0410',ProbeSetID='ILMN_2677092',CellID='',db2='Illum_Retina_BXD_RankInv0410',ProbeSetID2='ILMN_2435206',CellID2='',rank='1')) | [**0.798 79**](javascript:showCorrelationPlot2(db='Illum_Retina_BXD_RankInv0410',ProbeSetID='ILMN_2677092',CellID='',db2='Illum_Retina_BXD_RankInv0410',ProbeSetID2='ILMN_2789888',CellID2='',rank='1')) | [**0.683 79**](javascript:showCorrelationPlot2(db='Illum_Retina_BXD_RankInv0410',ProbeSetID='ILMN_2677092',CellID='',db2='Illum_Retina_BXD_RankInv0410',ProbeSetID2='ILMN_1218901',CellID2='',rank='1')) | [**0.712 79**](javascript:showCorrelationPlot2(db='Illum_Retina_BXD_RankInv0410',ProbeSetID='ILMN_2677092',CellID='',db2='Illum_Retina_BXD_RankInv0410',ProbeSetID2='ILMN_1254734',CellID2='',rank='1')) | [**0.599 79**](javascript:showCorrelationPlot2(db='Illum_Retina_BXD_RankInv0410',ProbeSetID='ILMN_2677092',CellID='',db2='Illum_Retina_BXD_RankInv0410',ProbeSetID2='ILMN_3008924',CellID2='',rank='1')) | [**0.668 79**](javascript:showCorrelationPlot2(db='Illum_Retina_BXD_RankInv0410',ProbeSetID='ILMN_2677092',CellID='',db2='Illum_Retina_BXD_RankInv0410',ProbeSetID2='ILMN_2628175',CellID2='',rank='1')) | [**0.557 79**](javascript:showCorrelationPlot2(db='Illum_Retina_BXD_RankInv0410',ProbeSetID='ILMN_2677092',CellID='',db2='Illum_Retina_BXD_RankInv0410',ProbeSetID2='ILMN_2891573',CellID2='',rank='1')) | [**0.677 79**](javascript:showCorrelationPlot2(db='Illum_Retina_BXD_RankInv0410',ProbeSetID='ILMN_2677092',CellID='',db2='Illum_Retina_BXD_RankInv0410',ProbeSetID2='ILMN_1220846',CellID2='',rank='1')) | [**0.479 79**](javascript:showCorrelationPlot2(db='Illum_Retina_BXD_RankInv0410',ProbeSetID='ILMN_2677092',CellID='',db2='Illum_Retina_BXD_RankInv0410',ProbeSetID2='ILMN_1241962',CellID2='',rank='1')) | [**0.556 79**](javascript:showCorrelationPlot2(db='Illum_Retina_BXD_RankInv0410',ProbeSetID='ILMN_2677092',CellID='',db2='Illum_Retina_BXD_RankInv0410',ProbeSetID2='ILMN_3163027',CellID2='',rank='1')) | [**0.517 79**](javascript:showCorrelationPlot2(db='Illum_Retina_BXD_RankInv0410',ProbeSetID='ILMN_2677092',CellID='',db2='Illum_Retina_BXD_RankInv0410',ProbeSetID2='ILMN_2775098',CellID2='',rank='1')) | [**0.459 79**](javascript:showCorrelationPlot2(db='Illum_Retina_BXD_RankInv0410',ProbeSetID='ILMN_2677092',CellID='',db2='Illum_Retina_BXD_RankInv0410',ProbeSetID2='ILMN_2752552',CellID2='',rank='1')) | [**0.677 79**](javascript:showCorrelationPlot2(db='Illum_Retina_BXD_RankInv0410',ProbeSetID='ILMN_2677092',CellID='',db2='Illum_Retina_BXD_RankInv0410',ProbeSetID2='ILMN_2730293',CellID2='',rank='1')) | [**0.386 79**](javascript:showCorrelationPlot2(db='Illum_Retina_BXD_RankInv0410',ProbeSetID='ILMN_2677092',CellID='',db2='Illum_Retina_BXD_RankInv0410',ProbeSetID2='ILMN_2680872',CellID2='',rank='1')) | [**0.178 79**](javascript:showCorrelationPlot2(db='Illum_Retina_BXD_RankInv0410',ProbeSetID='ILMN_2677092',CellID='',db2='Illum_Retina_BXD_RankInv0410',ProbeSetID2='ILMN_2768533',CellID2='',rank='1')) | [**-0.220 79**](javascript:showCorrelationPlot2(db='Illum_Retina_BXD_RankInv0410',ProbeSetID='ILMN_2677092',CellID='',db2='Illum_Retina_BXD_RankInv0410',ProbeSetID2='ILMN_3121255',CellID2='',rank='1')) | [**-0.455 79**](javascript:showCorrelationPlot2(db='Illum_Retina_BXD_RankInv0410',ProbeSetID='ILMN_2677092',CellID='',db2='Illum_Retina_BXD_RankInv0410',ProbeSetID2='ILMN_2484527',CellID2='',rank='1')) | [**0.202 79**](javascript:showCorrelationPlot2(db='Illum_Retina_BXD_RankInv0410',ProbeSetID='ILMN_2677092',CellID='',db2='Illum_Retina_BXD_RankInv0410',ProbeSetID2='ILMN_3045723',CellID2='',rank='1')) | [**0.371 79**](javascript:showCorrelationPlot2(db='Illum_Retina_BXD_RankInv0410',ProbeSetID='ILMN_2677092',CellID='',db2='Illum_Retina_BXD_RankInv0410',ProbeSetID2='ILMN_2486573',CellID2='',rank='1')) |
|  | [**Trait 4: ILMN_2624938**](javascript:showDatabase2('Illum_Retina_BXD_RankInv0410','ILMN_2624938','');)  Symbol: Pea15a  Pea15a | [**0.727 79**](javascript:showCorrelationPlot2(db='Illum_Retina_BXD_RankInv0410',ProbeSetID='ILMN_2624938',CellID='',db2='Illum_Retina_BXD_RankInv0410',ProbeSetID2='ILMN_2450384',CellID2='',rank='0')) | [**0.702 79**](javascript:showCorrelationPlot2(db='Illum_Retina_BXD_RankInv0410',ProbeSetID='ILMN_2624938',CellID='',db2='Illum_Retina_BXD_RankInv0410',ProbeSetID2='ILMN_3068754',CellID2='',rank='0')) | [**0.670 79**](javascript:showCorrelationPlot2(db='Illum_Retina_BXD_RankInv0410',ProbeSetID='ILMN_2624938',CellID='',db2='Illum_Retina_BXD_RankInv0410',ProbeSetID2='ILMN_2677092',CellID2='',rank='0')) | [***n* 79**](javascript:showDatabase2('Illum_Retina_BXD_RankInv0410','ILMN_2624938','')) | [**0.740 79**](javascript:showCorrelationPlot2(db='Illum_Retina_BXD_RankInv0410',ProbeSetID='ILMN_2624938',CellID='',db2='Illum_Retina_BXD_RankInv0410',ProbeSetID2='ILMN_2847269',CellID2='',rank='1')) | [**0.592 79**](javascript:showCorrelationPlot2(db='Illum_Retina_BXD_RankInv0410',ProbeSetID='ILMN_2624938',CellID='',db2='Illum_Retina_BXD_RankInv0410',ProbeSetID2='ILMN_2839682',CellID2='',rank='1')) | [**0.564 79**](javascript:showCorrelationPlot2(db='Illum_Retina_BXD_RankInv0410',ProbeSetID='ILMN_2624938',CellID='',db2='Illum_Retina_BXD_RankInv0410',ProbeSetID2='ILMN_3147135',CellID2='',rank='1')) | [**0.611 79**](javascript:showCorrelationPlot2(db='Illum_Retina_BXD_RankInv0410',ProbeSetID='ILMN_2624938',CellID='',db2='Illum_Retina_BXD_RankInv0410',ProbeSetID2='ILMN_1255823',CellID2='',rank='1')) | [**0.766 79**](javascript:showCorrelationPlot2(db='Illum_Retina_BXD_RankInv0410',ProbeSetID='ILMN_2624938',CellID='',db2='Illum_Retina_BXD_RankInv0410',ProbeSetID2='ILMN_2626143',CellID2='',rank='1')) | [**0.694 79**](javascript:showCorrelationPlot2(db='Illum_Retina_BXD_RankInv0410',ProbeSetID='ILMN_2624938',CellID='',db2='Illum_Retina_BXD_RankInv0410',ProbeSetID2='ILMN_1234930',CellID2='',rank='1')) | [**0.630 79**](javascript:showCorrelationPlot2(db='Illum_Retina_BXD_RankInv0410',ProbeSetID='ILMN_2624938',CellID='',db2='Illum_Retina_BXD_RankInv0410',ProbeSetID2='ILMN_1249408',CellID2='',rank='1')) | [**0.721 79**](javascript:showCorrelationPlot2(db='Illum_Retina_BXD_RankInv0410',ProbeSetID='ILMN_2624938',CellID='',db2='Illum_Retina_BXD_RankInv0410',ProbeSetID2='ILMN_2971559',CellID2='',rank='1')) | [**0.568 79**](javascript:showCorrelationPlot2(db='Illum_Retina_BXD_RankInv0410',ProbeSetID='ILMN_2624938',CellID='',db2='Illum_Retina_BXD_RankInv0410',ProbeSetID2='ILMN_1258455',CellID2='',rank='1')) | [**0.652 79**](javascript:showCorrelationPlot2(db='Illum_Retina_BXD_RankInv0410',ProbeSetID='ILMN_2624938',CellID='',db2='Illum_Retina_BXD_RankInv0410',ProbeSetID2='ILMN_2691613',CellID2='',rank='1')) | [**0.576 79**](javascript:showCorrelationPlot2(db='Illum_Retina_BXD_RankInv0410',ProbeSetID='ILMN_2624938',CellID='',db2='Illum_Retina_BXD_RankInv0410',ProbeSetID2='ILMN_1253600',CellID2='',rank='1')) | [**0.557 79**](javascript:showCorrelationPlot2(db='Illum_Retina_BXD_RankInv0410',ProbeSetID='ILMN_2624938',CellID='',db2='Illum_Retina_BXD_RankInv0410',ProbeSetID2='ILMN_2991545',CellID2='',rank='1')) | [**0.601 79**](javascript:showCorrelationPlot2(db='Illum_Retina_BXD_RankInv0410',ProbeSetID='ILMN_2624938',CellID='',db2='Illum_Retina_BXD_RankInv0410',ProbeSetID2='ILMN_2623591',CellID2='',rank='1')) | [**0.448 79**](javascript:showCorrelationPlot2(db='Illum_Retina_BXD_RankInv0410',ProbeSetID='ILMN_2624938',CellID='',db2='Illum_Retina_BXD_RankInv0410',ProbeSetID2='ILMN_2603568',CellID2='',rank='1')) | [**0.685 79**](javascript:showCorrelationPlot2(db='Illum_Retina_BXD_RankInv0410',ProbeSetID='ILMN_2624938',CellID='',db2='Illum_Retina_BXD_RankInv0410',ProbeSetID2='ILMN_2992541',CellID2='',rank='1')) | [**0.503 79**](javascript:showCorrelationPlot2(db='Illum_Retina_BXD_RankInv0410',ProbeSetID='ILMN_2624938',CellID='',db2='Illum_Retina_BXD_RankInv0410',ProbeSetID2='ILMN_2792868',CellID2='',rank='1')) | [**0.685 79**](javascript:showCorrelationPlot2(db='Illum_Retina_BXD_RankInv0410',ProbeSetID='ILMN_2624938',CellID='',db2='Illum_Retina_BXD_RankInv0410',ProbeSetID2='ILMN_3129497',CellID2='',rank='1')) | [**0.542 79**](javascript:showCorrelationPlot2(db='Illum_Retina_BXD_RankInv0410',ProbeSetID='ILMN_2624938',CellID='',db2='Illum_Retina_BXD_RankInv0410',ProbeSetID2='ILMN_1224034',CellID2='',rank='1')) | [**0.614 79**](javascript:showCorrelationPlot2(db='Illum_Retina_BXD_RankInv0410',ProbeSetID='ILMN_2624938',CellID='',db2='Illum_Retina_BXD_RankInv0410',ProbeSetID2='ILMN_2639819',CellID2='',rank='1')) | [**0.553 79**](javascript:showCorrelationPlot2(db='Illum_Retina_BXD_RankInv0410',ProbeSetID='ILMN_2624938',CellID='',db2='Illum_Retina_BXD_RankInv0410',ProbeSetID2='ILMN_2994779',CellID2='',rank='1')) | [**0.640 79**](javascript:showCorrelationPlot2(db='Illum_Retina_BXD_RankInv0410',ProbeSetID='ILMN_2624938',CellID='',db2='Illum_Retina_BXD_RankInv0410',ProbeSetID2='ILMN_2625047',CellID2='',rank='1')) | [**0.449 79**](javascript:showCorrelationPlot2(db='Illum_Retina_BXD_RankInv0410',ProbeSetID='ILMN_2624938',CellID='',db2='Illum_Retina_BXD_RankInv0410',ProbeSetID2='ILMN_2808186',CellID2='',rank='1')) | [**0.652 79**](javascript:showCorrelationPlot2(db='Illum_Retina_BXD_RankInv0410',ProbeSetID='ILMN_2624938',CellID='',db2='Illum_Retina_BXD_RankInv0410',ProbeSetID2='ILMN_2689056',CellID2='',rank='1')) | [**0.595 79**](javascript:showCorrelationPlot2(db='Illum_Retina_BXD_RankInv0410',ProbeSetID='ILMN_2624938',CellID='',db2='Illum_Retina_BXD_RankInv0410',ProbeSetID2='ILMN_2648386',CellID2='',rank='1')) | [**0.662 79**](javascript:showCorrelationPlot2(db='Illum_Retina_BXD_RankInv0410',ProbeSetID='ILMN_2624938',CellID='',db2='Illum_Retina_BXD_RankInv0410',ProbeSetID2='ILMN_2869225',CellID2='',rank='1')) | [**0.549 79**](javascript:showCorrelationPlot2(db='Illum_Retina_BXD_RankInv0410',ProbeSetID='ILMN_2624938',CellID='',db2='Illum_Retina_BXD_RankInv0410',ProbeSetID2='ILMN_2728431',CellID2='',rank='1')) | [**0.600 79**](javascript:showCorrelationPlot2(db='Illum_Retina_BXD_RankInv0410',ProbeSetID='ILMN_2624938',CellID='',db2='Illum_Retina_BXD_RankInv0410',ProbeSetID2='ILMN_3094608',CellID2='',rank='1')) | [**0.592 79**](javascript:showCorrelationPlot2(db='Illum_Retina_BXD_RankInv0410',ProbeSetID='ILMN_2624938',CellID='',db2='Illum_Retina_BXD_RankInv0410',ProbeSetID2='ILMN_2896528',CellID2='',rank='1')) | [**0.405 79**](javascript:showCorrelationPlot2(db='Illum_Retina_BXD_RankInv0410',ProbeSetID='ILMN_2624938',CellID='',db2='Illum_Retina_BXD_RankInv0410',ProbeSetID2='ILMN_2701750',CellID2='',rank='1')) | [**0.623 79**](javascript:showCorrelationPlot2(db='Illum_Retina_BXD_RankInv0410',ProbeSetID='ILMN_2624938',CellID='',db2='Illum_Retina_BXD_RankInv0410',ProbeSetID2='ILMN_1239448',CellID2='',rank='1')) | [**-0.388 79**](javascript:showCorrelationPlot2(db='Illum_Retina_BXD_RankInv0410',ProbeSetID='ILMN_2624938',CellID='',db2='Illum_Retina_BXD_RankInv0410',ProbeSetID2='ILMN_2583882',CellID2='',rank='1')) | [**0.528 79**](javascript:showCorrelationPlot2(db='Illum_Retina_BXD_RankInv0410',ProbeSetID='ILMN_2624938',CellID='',db2='Illum_Retina_BXD_RankInv0410',ProbeSetID2='ILMN_2915166',CellID2='',rank='1')) | [**0.513 79**](javascript:showCorrelationPlot2(db='Illum_Retina_BXD_RankInv0410',ProbeSetID='ILMN_2624938',CellID='',db2='Illum_Retina_BXD_RankInv0410',ProbeSetID2='ILMN_2702997',CellID2='',rank='1')) | [**0.561 79**](javascript:showCorrelationPlot2(db='Illum_Retina_BXD_RankInv0410',ProbeSetID='ILMN_2624938',CellID='',db2='Illum_Retina_BXD_RankInv0410',ProbeSetID2='ILMN_2435206',CellID2='',rank='1')) | [**0.588 79**](javascript:showCorrelationPlot2(db='Illum_Retina_BXD_RankInv0410',ProbeSetID='ILMN_2624938',CellID='',db2='Illum_Retina_BXD_RankInv0410',ProbeSetID2='ILMN_2789888',CellID2='',rank='1')) | [**0.616 79**](javascript:showCorrelationPlot2(db='Illum_Retina_BXD_RankInv0410',ProbeSetID='ILMN_2624938',CellID='',db2='Illum_Retina_BXD_RankInv0410',ProbeSetID2='ILMN_1218901',CellID2='',rank='1')) | [**0.504 79**](javascript:showCorrelationPlot2(db='Illum_Retina_BXD_RankInv0410',ProbeSetID='ILMN_2624938',CellID='',db2='Illum_Retina_BXD_RankInv0410',ProbeSetID2='ILMN_1254734',CellID2='',rank='1')) | [**0.518 79**](javascript:showCorrelationPlot2(db='Illum_Retina_BXD_RankInv0410',ProbeSetID='ILMN_2624938',CellID='',db2='Illum_Retina_BXD_RankInv0410',ProbeSetID2='ILMN_3008924',CellID2='',rank='1')) | [**0.560 79**](javascript:showCorrelationPlot2(db='Illum_Retina_BXD_RankInv0410',ProbeSetID='ILMN_2624938',CellID='',db2='Illum_Retina_BXD_RankInv0410',ProbeSetID2='ILMN_2628175',CellID2='',rank='1')) | [**0.654 79**](javascript:showCorrelationPlot2(db='Illum_Retina_BXD_RankInv0410',ProbeSetID='ILMN_2624938',CellID='',db2='Illum_Retina_BXD_RankInv0410',ProbeSetID2='ILMN_2891573',CellID2='',rank='1')) | [**0.715 79**](javascript:showCorrelationPlot2(db='Illum_Retina_BXD_RankInv0410',ProbeSetID='ILMN_2624938',CellID='',db2='Illum_Retina_BXD_RankInv0410',ProbeSetID2='ILMN_1220846',CellID2='',rank='1')) | [**0.516 79**](javascript:showCorrelationPlot2(db='Illum_Retina_BXD_RankInv0410',ProbeSetID='ILMN_2624938',CellID='',db2='Illum_Retina_BXD_RankInv0410',ProbeSetID2='ILMN_1241962',CellID2='',rank='1')) | [**0.539 79**](javascript:showCorrelationPlot2(db='Illum_Retina_BXD_RankInv0410',ProbeSetID='ILMN_2624938',CellID='',db2='Illum_Retina_BXD_RankInv0410',ProbeSetID2='ILMN_3163027',CellID2='',rank='1')) | [**0.282 79**](javascript:showCorrelationPlot2(db='Illum_Retina_BXD_RankInv0410',ProbeSetID='ILMN_2624938',CellID='',db2='Illum_Retina_BXD_RankInv0410',ProbeSetID2='ILMN_2775098',CellID2='',rank='1')) | [**0.768 79**](javascript:showCorrelationPlot2(db='Illum_Retina_BXD_RankInv0410',ProbeSetID='ILMN_2624938',CellID='',db2='Illum_Retina_BXD_RankInv0410',ProbeSetID2='ILMN_2752552',CellID2='',rank='1')) | [**0.540 79**](javascript:showCorrelationPlot2(db='Illum_Retina_BXD_RankInv0410',ProbeSetID='ILMN_2624938',CellID='',db2='Illum_Retina_BXD_RankInv0410',ProbeSetID2='ILMN_2730293',CellID2='',rank='1')) | [**0.593 79**](javascript:showCorrelationPlot2(db='Illum_Retina_BXD_RankInv0410',ProbeSetID='ILMN_2624938',CellID='',db2='Illum_Retina_BXD_RankInv0410',ProbeSetID2='ILMN_2680872',CellID2='',rank='1')) | [**0.202 79**](javascript:showCorrelationPlot2(db='Illum_Retina_BXD_RankInv0410',ProbeSetID='ILMN_2624938',CellID='',db2='Illum_Retina_BXD_RankInv0410',ProbeSetID2='ILMN_2768533',CellID2='',rank='1')) | [**-0.244 79**](javascript:showCorrelationPlot2(db='Illum_Retina_BXD_RankInv0410',ProbeSetID='ILMN_2624938',CellID='',db2='Illum_Retina_BXD_RankInv0410',ProbeSetID2='ILMN_3121255',CellID2='',rank='1')) | [**-0.351 79**](javascript:showCorrelationPlot2(db='Illum_Retina_BXD_RankInv0410',ProbeSetID='ILMN_2624938',CellID='',db2='Illum_Retina_BXD_RankInv0410',ProbeSetID2='ILMN_2484527',CellID2='',rank='1')) | [**0.151 79**](javascript:showCorrelationPlot2(db='Illum_Retina_BXD_RankInv0410',ProbeSetID='ILMN_2624938',CellID='',db2='Illum_Retina_BXD_RankInv0410',ProbeSetID2='ILMN_3045723',CellID2='',rank='1')) | [**0.308 79**](javascript:showCorrelationPlot2(db='Illum_Retina_BXD_RankInv0410',ProbeSetID='ILMN_2624938',CellID='',db2='Illum_Retina_BXD_RankInv0410',ProbeSetID2='ILMN_2486573',CellID2='',rank='1')) |
|  | [**Trait 5: ILMN_2847269**](javascript:showDatabase2('Illum_Retina_BXD_RankInv0410','ILMN_2847269','');)  Symbol: Tprgl  Tprgl | [**0.722 79**](javascript:showCorrelationPlot2(db='Illum_Retina_BXD_RankInv0410',ProbeSetID='ILMN_2847269',CellID='',db2='Illum_Retina_BXD_RankInv0410',ProbeSetID2='ILMN_2450384',CellID2='',rank='0')) | [**0.556 79**](javascript:showCorrelationPlot2(db='Illum_Retina_BXD_RankInv0410',ProbeSetID='ILMN_2847269',CellID='',db2='Illum_Retina_BXD_RankInv0410',ProbeSetID2='ILMN_3068754',CellID2='',rank='0')) | [**0.682 79**](javascript:showCorrelationPlot2(db='Illum_Retina_BXD_RankInv0410',ProbeSetID='ILMN_2847269',CellID='',db2='Illum_Retina_BXD_RankInv0410',ProbeSetID2='ILMN_2677092',CellID2='',rank='0')) | [**0.738 79**](javascript:showCorrelationPlot2(db='Illum_Retina_BXD_RankInv0410',ProbeSetID='ILMN_2847269',CellID='',db2='Illum_Retina_BXD_RankInv0410',ProbeSetID2='ILMN_2624938',CellID2='',rank='0')) | [***n* 79**](javascript:showDatabase2('Illum_Retina_BXD_RankInv0410','ILMN_2847269','')) | [**0.513 79**](javascript:showCorrelationPlot2(db='Illum_Retina_BXD_RankInv0410',ProbeSetID='ILMN_2847269',CellID='',db2='Illum_Retina_BXD_RankInv0410',ProbeSetID2='ILMN_2839682',CellID2='',rank='1')) | [**0.719 79**](javascript:showCorrelationPlot2(db='Illum_Retina_BXD_RankInv0410',ProbeSetID='ILMN_2847269',CellID='',db2='Illum_Retina_BXD_RankInv0410',ProbeSetID2='ILMN_3147135',CellID2='',rank='1')) | [**0.698 79**](javascript:showCorrelationPlot2(db='Illum_Retina_BXD_RankInv0410',ProbeSetID='ILMN_2847269',CellID='',db2='Illum_Retina_BXD_RankInv0410',ProbeSetID2='ILMN_1255823',CellID2='',rank='1')) | [**0.547 79**](javascript:showCorrelationPlot2(db='Illum_Retina_BXD_RankInv0410',ProbeSetID='ILMN_2847269',CellID='',db2='Illum_Retina_BXD_RankInv0410',ProbeSetID2='ILMN_2626143',CellID2='',rank='1')) | [**0.568 79**](javascript:showCorrelationPlot2(db='Illum_Retina_BXD_RankInv0410',ProbeSetID='ILMN_2847269',CellID='',db2='Illum_Retina_BXD_RankInv0410',ProbeSetID2='ILMN_1234930',CellID2='',rank='1')) | [**0.659 79**](javascript:showCorrelationPlot2(db='Illum_Retina_BXD_RankInv0410',ProbeSetID='ILMN_2847269',CellID='',db2='Illum_Retina_BXD_RankInv0410',ProbeSetID2='ILMN_1249408',CellID2='',rank='1')) | [**0.708 79**](javascript:showCorrelationPlot2(db='Illum_Retina_BXD_RankInv0410',ProbeSetID='ILMN_2847269',CellID='',db2='Illum_Retina_BXD_RankInv0410',ProbeSetID2='ILMN_2971559',CellID2='',rank='1')) | [**0.423 79**](javascript:showCorrelationPlot2(db='Illum_Retina_BXD_RankInv0410',ProbeSetID='ILMN_2847269',CellID='',db2='Illum_Retina_BXD_RankInv0410',ProbeSetID2='ILMN_1258455',CellID2='',rank='1')) | [**0.786 79**](javascript:showCorrelationPlot2(db='Illum_Retina_BXD_RankInv0410',ProbeSetID='ILMN_2847269',CellID='',db2='Illum_Retina_BXD_RankInv0410',ProbeSetID2='ILMN_2691613',CellID2='',rank='1')) | [**0.478 79**](javascript:showCorrelationPlot2(db='Illum_Retina_BXD_RankInv0410',ProbeSetID='ILMN_2847269',CellID='',db2='Illum_Retina_BXD_RankInv0410',ProbeSetID2='ILMN_1253600',CellID2='',rank='1')) | [**0.537 79**](javascript:showCorrelationPlot2(db='Illum_Retina_BXD_RankInv0410',ProbeSetID='ILMN_2847269',CellID='',db2='Illum_Retina_BXD_RankInv0410',ProbeSetID2='ILMN_2991545',CellID2='',rank='1')) | [**0.505 79**](javascript:showCorrelationPlot2(db='Illum_Retina_BXD_RankInv0410',ProbeSetID='ILMN_2847269',CellID='',db2='Illum_Retina_BXD_RankInv0410',ProbeSetID2='ILMN_2623591',CellID2='',rank='1')) | [**0.465 79**](javascript:showCorrelationPlot2(db='Illum_Retina_BXD_RankInv0410',ProbeSetID='ILMN_2847269',CellID='',db2='Illum_Retina_BXD_RankInv0410',ProbeSetID2='ILMN_2603568',CellID2='',rank='1')) | [**0.771 79**](javascript:showCorrelationPlot2(db='Illum_Retina_BXD_RankInv0410',ProbeSetID='ILMN_2847269',CellID='',db2='Illum_Retina_BXD_RankInv0410',ProbeSetID2='ILMN_2992541',CellID2='',rank='1')) | [**0.543 79**](javascript:showCorrelationPlot2(db='Illum_Retina_BXD_RankInv0410',ProbeSetID='ILMN_2847269',CellID='',db2='Illum_Retina_BXD_RankInv0410',ProbeSetID2='ILMN_2792868',CellID2='',rank='1')) | [**0.617 79**](javascript:showCorrelationPlot2(db='Illum_Retina_BXD_RankInv0410',ProbeSetID='ILMN_2847269',CellID='',db2='Illum_Retina_BXD_RankInv0410',ProbeSetID2='ILMN_3129497',CellID2='',rank='1')) | [**0.597 79**](javascript:showCorrelationPlot2(db='Illum_Retina_BXD_RankInv0410',ProbeSetID='ILMN_2847269',CellID='',db2='Illum_Retina_BXD_RankInv0410',ProbeSetID2='ILMN_1224034',CellID2='',rank='1')) | [**0.565 79**](javascript:showCorrelationPlot2(db='Illum_Retina_BXD_RankInv0410',ProbeSetID='ILMN_2847269',CellID='',db2='Illum_Retina_BXD_RankInv0410',ProbeSetID2='ILMN_2639819',CellID2='',rank='1')) | [**0.637 79**](javascript:showCorrelationPlot2(db='Illum_Retina_BXD_RankInv0410',ProbeSetID='ILMN_2847269',CellID='',db2='Illum_Retina_BXD_RankInv0410',ProbeSetID2='ILMN_2994779',CellID2='',rank='1')) | [**0.555 79**](javascript:showCorrelationPlot2(db='Illum_Retina_BXD_RankInv0410',ProbeSetID='ILMN_2847269',CellID='',db2='Illum_Retina_BXD_RankInv0410',ProbeSetID2='ILMN_2625047',CellID2='',rank='1')) | [**0.425 79**](javascript:showCorrelationPlot2(db='Illum_Retina_BXD_RankInv0410',ProbeSetID='ILMN_2847269',CellID='',db2='Illum_Retina_BXD_RankInv0410',ProbeSetID2='ILMN_2808186',CellID2='',rank='1')) | [**0.517 79**](javascript:showCorrelationPlot2(db='Illum_Retina_BXD_RankInv0410',ProbeSetID='ILMN_2847269',CellID='',db2='Illum_Retina_BXD_RankInv0410',ProbeSetID2='ILMN_2689056',CellID2='',rank='1')) | [**0.621 79**](javascript:showCorrelationPlot2(db='Illum_Retina_BXD_RankInv0410',ProbeSetID='ILMN_2847269',CellID='',db2='Illum_Retina_BXD_RankInv0410',ProbeSetID2='ILMN_2648386',CellID2='',rank='1')) | [**0.674 79**](javascript:showCorrelationPlot2(db='Illum_Retina_BXD_RankInv0410',ProbeSetID='ILMN_2847269',CellID='',db2='Illum_Retina_BXD_RankInv0410',ProbeSetID2='ILMN_2869225',CellID2='',rank='1')) | [**0.533 79**](javascript:showCorrelationPlot2(db='Illum_Retina_BXD_RankInv0410',ProbeSetID='ILMN_2847269',CellID='',db2='Illum_Retina_BXD_RankInv0410',ProbeSetID2='ILMN_2728431',CellID2='',rank='1')) | [**0.351 79**](javascript:showCorrelationPlot2(db='Illum_Retina_BXD_RankInv0410',ProbeSetID='ILMN_2847269',CellID='',db2='Illum_Retina_BXD_RankInv0410',ProbeSetID2='ILMN_3094608',CellID2='',rank='1')) | [**0.586 79**](javascript:showCorrelationPlot2(db='Illum_Retina_BXD_RankInv0410',ProbeSetID='ILMN_2847269',CellID='',db2='Illum_Retina_BXD_RankInv0410',ProbeSetID2='ILMN_2896528',CellID2='',rank='1')) | [**0.454 79**](javascript:showCorrelationPlot2(db='Illum_Retina_BXD_RankInv0410',ProbeSetID='ILMN_2847269',CellID='',db2='Illum_Retina_BXD_RankInv0410',ProbeSetID2='ILMN_2701750',CellID2='',rank='1')) | [**0.343 79**](javascript:showCorrelationPlot2(db='Illum_Retina_BXD_RankInv0410',ProbeSetID='ILMN_2847269',CellID='',db2='Illum_Retina_BXD_RankInv0410',ProbeSetID2='ILMN_1239448',CellID2='',rank='1')) | [**-0.558 79**](javascript:showCorrelationPlot2(db='Illum_Retina_BXD_RankInv0410',ProbeSetID='ILMN_2847269',CellID='',db2='Illum_Retina_BXD_RankInv0410',ProbeSetID2='ILMN_2583882',CellID2='',rank='1')) | [**0.580 79**](javascript:showCorrelationPlot2(db='Illum_Retina_BXD_RankInv0410',ProbeSetID='ILMN_2847269',CellID='',db2='Illum_Retina_BXD_RankInv0410',ProbeSetID2='ILMN_2915166',CellID2='',rank='1')) | [**0.493 79**](javascript:showCorrelationPlot2(db='Illum_Retina_BXD_RankInv0410',ProbeSetID='ILMN_2847269',CellID='',db2='Illum_Retina_BXD_RankInv0410',ProbeSetID2='ILMN_2702997',CellID2='',rank='1')) | [**0.490 79**](javascript:showCorrelationPlot2(db='Illum_Retina_BXD_RankInv0410',ProbeSetID='ILMN_2847269',CellID='',db2='Illum_Retina_BXD_RankInv0410',ProbeSetID2='ILMN_2435206',CellID2='',rank='1')) | [**0.536 79**](javascript:showCorrelationPlot2(db='Illum_Retina_BXD_RankInv0410',ProbeSetID='ILMN_2847269',CellID='',db2='Illum_Retina_BXD_RankInv0410',ProbeSetID2='ILMN_2789888',CellID2='',rank='1')) | [**0.457 79**](javascript:showCorrelationPlot2(db='Illum_Retina_BXD_RankInv0410',ProbeSetID='ILMN_2847269',CellID='',db2='Illum_Retina_BXD_RankInv0410',ProbeSetID2='ILMN_1218901',CellID2='',rank='1')) | [**0.551 79**](javascript:showCorrelationPlot2(db='Illum_Retina_BXD_RankInv0410',ProbeSetID='ILMN_2847269',CellID='',db2='Illum_Retina_BXD_RankInv0410',ProbeSetID2='ILMN_1254734',CellID2='',rank='1')) | [**0.400 79**](javascript:showCorrelationPlot2(db='Illum_Retina_BXD_RankInv0410',ProbeSetID='ILMN_2847269',CellID='',db2='Illum_Retina_BXD_RankInv0410',ProbeSetID2='ILMN_3008924',CellID2='',rank='1')) | [**0.488 79**](javascript:showCorrelationPlot2(db='Illum_Retina_BXD_RankInv0410',ProbeSetID='ILMN_2847269',CellID='',db2='Illum_Retina_BXD_RankInv0410',ProbeSetID2='ILMN_2628175',CellID2='',rank='1')) | [**0.619 79**](javascript:showCorrelationPlot2(db='Illum_Retina_BXD_RankInv0410',ProbeSetID='ILMN_2847269',CellID='',db2='Illum_Retina_BXD_RankInv0410',ProbeSetID2='ILMN_2891573',CellID2='',rank='1')) | [**0.583 79**](javascript:showCorrelationPlot2(db='Illum_Retina_BXD_RankInv0410',ProbeSetID='ILMN_2847269',CellID='',db2='Illum_Retina_BXD_RankInv0410',ProbeSetID2='ILMN_1220846',CellID2='',rank='1')) | [**0.579 79**](javascript:showCorrelationPlot2(db='Illum_Retina_BXD_RankInv0410',ProbeSetID='ILMN_2847269',CellID='',db2='Illum_Retina_BXD_RankInv0410',ProbeSetID2='ILMN_1241962',CellID2='',rank='1')) | [**0.724 79**](javascript:showCorrelationPlot2(db='Illum_Retina_BXD_RankInv0410',ProbeSetID='ILMN_2847269',CellID='',db2='Illum_Retina_BXD_RankInv0410',ProbeSetID2='ILMN_3163027',CellID2='',rank='1')) | [**0.388 79**](javascript:showCorrelationPlot2(db='Illum_Retina_BXD_RankInv0410',ProbeSetID='ILMN_2847269',CellID='',db2='Illum_Retina_BXD_RankInv0410',ProbeSetID2='ILMN_2775098',CellID2='',rank='1')) | [**0.550 79**](javascript:showCorrelationPlot2(db='Illum_Retina_BXD_RankInv0410',ProbeSetID='ILMN_2847269',CellID='',db2='Illum_Retina_BXD_RankInv0410',ProbeSetID2='ILMN_2752552',CellID2='',rank='1')) | [**0.564 79**](javascript:showCorrelationPlot2(db='Illum_Retina_BXD_RankInv0410',ProbeSetID='ILMN_2847269',CellID='',db2='Illum_Retina_BXD_RankInv0410',ProbeSetID2='ILMN_2730293',CellID2='',rank='1')) | [**0.520 79**](javascript:showCorrelationPlot2(db='Illum_Retina_BXD_RankInv0410',ProbeSetID='ILMN_2847269',CellID='',db2='Illum_Retina_BXD_RankInv0410',ProbeSetID2='ILMN_2680872',CellID2='',rank='1')) | [**0.169 79**](javascript:showCorrelationPlot2(db='Illum_Retina_BXD_RankInv0410',ProbeSetID='ILMN_2847269',CellID='',db2='Illum_Retina_BXD_RankInv0410',ProbeSetID2='ILMN_2768533',CellID2='',rank='1')) | [**-0.014 79**](javascript:showCorrelationPlot2(db='Illum_Retina_BXD_RankInv0410',ProbeSetID='ILMN_2847269',CellID='',db2='Illum_Retina_BXD_RankInv0410',ProbeSetID2='ILMN_3121255',CellID2='',rank='1')) | [**-0.302 79**](javascript:showCorrelationPlot2(db='Illum_Retina_BXD_RankInv0410',ProbeSetID='ILMN_2847269',CellID='',db2='Illum_Retina_BXD_RankInv0410',ProbeSetID2='ILMN_2484527',CellID2='',rank='1')) | [**0.303 79**](javascript:showCorrelationPlot2(db='Illum_Retina_BXD_RankInv0410',ProbeSetID='ILMN_2847269',CellID='',db2='Illum_Retina_BXD_RankInv0410',ProbeSetID2='ILMN_3045723',CellID2='',rank='1')) | [**0.252 79**](javascript:showCorrelationPlot2(db='Illum_Retina_BXD_RankInv0410',ProbeSetID='ILMN_2847269',CellID='',db2='Illum_Retina_BXD_RankInv0410',ProbeSetID2='ILMN_2486573',CellID2='',rank='1')) |
|  | [**Trait 6: ILMN_2839682**](javascript:showDatabase2('Illum_Retina_BXD_RankInv0410','ILMN_2839682','');)  Symbol: Btbd6  Btbd6 | [**0.698 79**](javascript:showCorrelationPlot2(db='Illum_Retina_BXD_RankInv0410',ProbeSetID='ILMN_2839682',CellID='',db2='Illum_Retina_BXD_RankInv0410',ProbeSetID2='ILMN_2450384',CellID2='',rank='0')) | [**0.675 79**](javascript:showCorrelationPlot2(db='Illum_Retina_BXD_RankInv0410',ProbeSetID='ILMN_2839682',CellID='',db2='Illum_Retina_BXD_RankInv0410',ProbeSetID2='ILMN_3068754',CellID2='',rank='0')) | [**0.580 79**](javascript:showCorrelationPlot2(db='Illum_Retina_BXD_RankInv0410',ProbeSetID='ILMN_2839682',CellID='',db2='Illum_Retina_BXD_RankInv0410',ProbeSetID2='ILMN_2677092',CellID2='',rank='0')) | [**0.657 79**](javascript:showCorrelationPlot2(db='Illum_Retina_BXD_RankInv0410',ProbeSetID='ILMN_2839682',CellID='',db2='Illum_Retina_BXD_RankInv0410',ProbeSetID2='ILMN_2624938',CellID2='',rank='0')) | [**0.555 79**](javascript:showCorrelationPlot2(db='Illum_Retina_BXD_RankInv0410',ProbeSetID='ILMN_2839682',CellID='',db2='Illum_Retina_BXD_RankInv0410',ProbeSetID2='ILMN_2847269',CellID2='',rank='0')) | [***n* 79**](javascript:showDatabase2('Illum_Retina_BXD_RankInv0410','ILMN_2839682','')) | [**0.582 79**](javascript:showCorrelationPlot2(db='Illum_Retina_BXD_RankInv0410',ProbeSetID='ILMN_2839682',CellID='',db2='Illum_Retina_BXD_RankInv0410',ProbeSetID2='ILMN_3147135',CellID2='',rank='1')) | [**0.412 79**](javascript:showCorrelationPlot2(db='Illum_Retina_BXD_RankInv0410',ProbeSetID='ILMN_2839682',CellID='',db2='Illum_Retina_BXD_RankInv0410',ProbeSetID2='ILMN_1255823',CellID2='',rank='1')) | [**0.492 79**](javascript:showCorrelationPlot2(db='Illum_Retina_BXD_RankInv0410',ProbeSetID='ILMN_2839682',CellID='',db2='Illum_Retina_BXD_RankInv0410',ProbeSetID2='ILMN_2626143',CellID2='',rank='1')) | [**0.520 79**](javascript:showCorrelationPlot2(db='Illum_Retina_BXD_RankInv0410',ProbeSetID='ILMN_2839682',CellID='',db2='Illum_Retina_BXD_RankInv0410',ProbeSetID2='ILMN_1234930',CellID2='',rank='1')) | [**0.436 79**](javascript:showCorrelationPlot2(db='Illum_Retina_BXD_RankInv0410',ProbeSetID='ILMN_2839682',CellID='',db2='Illum_Retina_BXD_RankInv0410',ProbeSetID2='ILMN_1249408',CellID2='',rank='1')) | [**0.526 79**](javascript:showCorrelationPlot2(db='Illum_Retina_BXD_RankInv0410',ProbeSetID='ILMN_2839682',CellID='',db2='Illum_Retina_BXD_RankInv0410',ProbeSetID2='ILMN_2971559',CellID2='',rank='1')) | [**0.464 79**](javascript:showCorrelationPlot2(db='Illum_Retina_BXD_RankInv0410',ProbeSetID='ILMN_2839682',CellID='',db2='Illum_Retina_BXD_RankInv0410',ProbeSetID2='ILMN_1258455',CellID2='',rank='1')) | [**0.560 79**](javascript:showCorrelationPlot2(db='Illum_Retina_BXD_RankInv0410',ProbeSetID='ILMN_2839682',CellID='',db2='Illum_Retina_BXD_RankInv0410',ProbeSetID2='ILMN_2691613',CellID2='',rank='1')) | [**0.631 79**](javascript:showCorrelationPlot2(db='Illum_Retina_BXD_RankInv0410',ProbeSetID='ILMN_2839682',CellID='',db2='Illum_Retina_BXD_RankInv0410',ProbeSetID2='ILMN_1253600',CellID2='',rank='1')) | [**0.574 79**](javascript:showCorrelationPlot2(db='Illum_Retina_BXD_RankInv0410',ProbeSetID='ILMN_2839682',CellID='',db2='Illum_Retina_BXD_RankInv0410',ProbeSetID2='ILMN_2991545',CellID2='',rank='1')) | [**0.708 79**](javascript:showCorrelationPlot2(db='Illum_Retina_BXD_RankInv0410',ProbeSetID='ILMN_2839682',CellID='',db2='Illum_Retina_BXD_RankInv0410',ProbeSetID2='ILMN_2623591',CellID2='',rank='1')) | [**0.454 79**](javascript:showCorrelationPlot2(db='Illum_Retina_BXD_RankInv0410',ProbeSetID='ILMN_2839682',CellID='',db2='Illum_Retina_BXD_RankInv0410',ProbeSetID2='ILMN_2603568',CellID2='',rank='1')) | [**0.535 79**](javascript:showCorrelationPlot2(db='Illum_Retina_BXD_RankInv0410',ProbeSetID='ILMN_2839682',CellID='',db2='Illum_Retina_BXD_RankInv0410',ProbeSetID2='ILMN_2992541',CellID2='',rank='1')) | [**0.369 79**](javascript:showCorrelationPlot2(db='Illum_Retina_BXD_RankInv0410',ProbeSetID='ILMN_2839682',CellID='',db2='Illum_Retina_BXD_RankInv0410',ProbeSetID2='ILMN_2792868',CellID2='',rank='1')) | [**0.512 79**](javascript:showCorrelationPlot2(db='Illum_Retina_BXD_RankInv0410',ProbeSetID='ILMN_2839682',CellID='',db2='Illum_Retina_BXD_RankInv0410',ProbeSetID2='ILMN_3129497',CellID2='',rank='1')) | [**0.516 79**](javascript:showCorrelationPlot2(db='Illum_Retina_BXD_RankInv0410',ProbeSetID='ILMN_2839682',CellID='',db2='Illum_Retina_BXD_RankInv0410',ProbeSetID2='ILMN_1224034',CellID2='',rank='1')) | [**0.508 79**](javascript:showCorrelationPlot2(db='Illum_Retina_BXD_RankInv0410',ProbeSetID='ILMN_2839682',CellID='',db2='Illum_Retina_BXD_RankInv0410',ProbeSetID2='ILMN_2639819',CellID2='',rank='1')) | [**0.399 79**](javascript:showCorrelationPlot2(db='Illum_Retina_BXD_RankInv0410',ProbeSetID='ILMN_2839682',CellID='',db2='Illum_Retina_BXD_RankInv0410',ProbeSetID2='ILMN_2994779',CellID2='',rank='1')) | [**0.605 79**](javascript:showCorrelationPlot2(db='Illum_Retina_BXD_RankInv0410',ProbeSetID='ILMN_2839682',CellID='',db2='Illum_Retina_BXD_RankInv0410',ProbeSetID2='ILMN_2625047',CellID2='',rank='1')) | [**0.579 79**](javascript:showCorrelationPlot2(db='Illum_Retina_BXD_RankInv0410',ProbeSetID='ILMN_2839682',CellID='',db2='Illum_Retina_BXD_RankInv0410',ProbeSetID2='ILMN_2808186',CellID2='',rank='1')) | [**0.612 79**](javascript:showCorrelationPlot2(db='Illum_Retina_BXD_RankInv0410',ProbeSetID='ILMN_2839682',CellID='',db2='Illum_Retina_BXD_RankInv0410',ProbeSetID2='ILMN_2689056',CellID2='',rank='1')) | [**0.506 79**](javascript:showCorrelationPlot2(db='Illum_Retina_BXD_RankInv0410',ProbeSetID='ILMN_2839682',CellID='',db2='Illum_Retina_BXD_RankInv0410',ProbeSetID2='ILMN_2648386',CellID2='',rank='1')) | [**0.422 79**](javascript:showCorrelationPlot2(db='Illum_Retina_BXD_RankInv0410',ProbeSetID='ILMN_2839682',CellID='',db2='Illum_Retina_BXD_RankInv0410',ProbeSetID2='ILMN_2869225',CellID2='',rank='1')) | [**0.587 79**](javascript:showCorrelationPlot2(db='Illum_Retina_BXD_RankInv0410',ProbeSetID='ILMN_2839682',CellID='',db2='Illum_Retina_BXD_RankInv0410',ProbeSetID2='ILMN_2728431',CellID2='',rank='1')) | [**0.605 79**](javascript:showCorrelationPlot2(db='Illum_Retina_BXD_RankInv0410',ProbeSetID='ILMN_2839682',CellID='',db2='Illum_Retina_BXD_RankInv0410',ProbeSetID2='ILMN_3094608',CellID2='',rank='1')) | [**0.399 79**](javascript:showCorrelationPlot2(db='Illum_Retina_BXD_RankInv0410',ProbeSetID='ILMN_2839682',CellID='',db2='Illum_Retina_BXD_RankInv0410',ProbeSetID2='ILMN_2896528',CellID2='',rank='1')) | [**0.436 79**](javascript:showCorrelationPlot2(db='Illum_Retina_BXD_RankInv0410',ProbeSetID='ILMN_2839682',CellID='',db2='Illum_Retina_BXD_RankInv0410',ProbeSetID2='ILMN_2701750',CellID2='',rank='1')) | [**0.581 79**](javascript:showCorrelationPlot2(db='Illum_Retina_BXD_RankInv0410',ProbeSetID='ILMN_2839682',CellID='',db2='Illum_Retina_BXD_RankInv0410',ProbeSetID2='ILMN_1239448',CellID2='',rank='1')) | [**-0.467 79**](javascript:showCorrelationPlot2(db='Illum_Retina_BXD_RankInv0410',ProbeSetID='ILMN_2839682',CellID='',db2='Illum_Retina_BXD_RankInv0410',ProbeSetID2='ILMN_2583882',CellID2='',rank='1')) | [**0.365 79**](javascript:showCorrelationPlot2(db='Illum_Retina_BXD_RankInv0410',ProbeSetID='ILMN_2839682',CellID='',db2='Illum_Retina_BXD_RankInv0410',ProbeSetID2='ILMN_2915166',CellID2='',rank='1')) | [**0.444 79**](javascript:showCorrelationPlot2(db='Illum_Retina_BXD_RankInv0410',ProbeSetID='ILMN_2839682',CellID='',db2='Illum_Retina_BXD_RankInv0410',ProbeSetID2='ILMN_2702997',CellID2='',rank='1')) | [**0.441 79**](javascript:showCorrelationPlot2(db='Illum_Retina_BXD_RankInv0410',ProbeSetID='ILMN_2839682',CellID='',db2='Illum_Retina_BXD_RankInv0410',ProbeSetID2='ILMN_2435206',CellID2='',rank='1')) | [**0.602 79**](javascript:showCorrelationPlot2(db='Illum_Retina_BXD_RankInv0410',ProbeSetID='ILMN_2839682',CellID='',db2='Illum_Retina_BXD_RankInv0410',ProbeSetID2='ILMN_2789888',CellID2='',rank='1')) | [**0.490 79**](javascript:showCorrelationPlot2(db='Illum_Retina_BXD_RankInv0410',ProbeSetID='ILMN_2839682',CellID='',db2='Illum_Retina_BXD_RankInv0410',ProbeSetID2='ILMN_1218901',CellID2='',rank='1')) | [**0.307 79**](javascript:showCorrelationPlot2(db='Illum_Retina_BXD_RankInv0410',ProbeSetID='ILMN_2839682',CellID='',db2='Illum_Retina_BXD_RankInv0410',ProbeSetID2='ILMN_1254734',CellID2='',rank='1')) | [**0.645 79**](javascript:showCorrelationPlot2(db='Illum_Retina_BXD_RankInv0410',ProbeSetID='ILMN_2839682',CellID='',db2='Illum_Retina_BXD_RankInv0410',ProbeSetID2='ILMN_3008924',CellID2='',rank='1')) | [**0.588 79**](javascript:showCorrelationPlot2(db='Illum_Retina_BXD_RankInv0410',ProbeSetID='ILMN_2839682',CellID='',db2='Illum_Retina_BXD_RankInv0410',ProbeSetID2='ILMN_2628175',CellID2='',rank='1')) | [**0.564 79**](javascript:showCorrelationPlot2(db='Illum_Retina_BXD_RankInv0410',ProbeSetID='ILMN_2839682',CellID='',db2='Illum_Retina_BXD_RankInv0410',ProbeSetID2='ILMN_2891573',CellID2='',rank='1')) | [**0.506 79**](javascript:showCorrelationPlot2(db='Illum_Retina_BXD_RankInv0410',ProbeSetID='ILMN_2839682',CellID='',db2='Illum_Retina_BXD_RankInv0410',ProbeSetID2='ILMN_1220846',CellID2='',rank='1')) | [**0.413 79**](javascript:showCorrelationPlot2(db='Illum_Retina_BXD_RankInv0410',ProbeSetID='ILMN_2839682',CellID='',db2='Illum_Retina_BXD_RankInv0410',ProbeSetID2='ILMN_1241962',CellID2='',rank='1')) | [**0.408 79**](javascript:showCorrelationPlot2(db='Illum_Retina_BXD_RankInv0410',ProbeSetID='ILMN_2839682',CellID='',db2='Illum_Retina_BXD_RankInv0410',ProbeSetID2='ILMN_3163027',CellID2='',rank='1')) | [**0.275 79**](javascript:showCorrelationPlot2(db='Illum_Retina_BXD_RankInv0410',ProbeSetID='ILMN_2839682',CellID='',db2='Illum_Retina_BXD_RankInv0410',ProbeSetID2='ILMN_2775098',CellID2='',rank='1')) | [**0.518 79**](javascript:showCorrelationPlot2(db='Illum_Retina_BXD_RankInv0410',ProbeSetID='ILMN_2839682',CellID='',db2='Illum_Retina_BXD_RankInv0410',ProbeSetID2='ILMN_2752552',CellID2='',rank='1')) | [**0.542 79**](javascript:showCorrelationPlot2(db='Illum_Retina_BXD_RankInv0410',ProbeSetID='ILMN_2839682',CellID='',db2='Illum_Retina_BXD_RankInv0410',ProbeSetID2='ILMN_2730293',CellID2='',rank='1')) | [**0.463 79**](javascript:showCorrelationPlot2(db='Illum_Retina_BXD_RankInv0410',ProbeSetID='ILMN_2839682',CellID='',db2='Illum_Retina_BXD_RankInv0410',ProbeSetID2='ILMN_2680872',CellID2='',rank='1')) | [**0.213 79**](javascript:showCorrelationPlot2(db='Illum_Retina_BXD_RankInv0410',ProbeSetID='ILMN_2839682',CellID='',db2='Illum_Retina_BXD_RankInv0410',ProbeSetID2='ILMN_2768533',CellID2='',rank='1')) | [**-0.253 79**](javascript:showCorrelationPlot2(db='Illum_Retina_BXD_RankInv0410',ProbeSetID='ILMN_2839682',CellID='',db2='Illum_Retina_BXD_RankInv0410',ProbeSetID2='ILMN_3121255',CellID2='',rank='1')) | [**-0.371 79**](javascript:showCorrelationPlot2(db='Illum_Retina_BXD_RankInv0410',ProbeSetID='ILMN_2839682',CellID='',db2='Illum_Retina_BXD_RankInv0410',ProbeSetID2='ILMN_2484527',CellID2='',rank='1')) | [**0.223 79**](javascript:showCorrelationPlot2(db='Illum_Retina_BXD_RankInv0410',ProbeSetID='ILMN_2839682',CellID='',db2='Illum_Retina_BXD_RankInv0410',ProbeSetID2='ILMN_3045723',CellID2='',rank='1')) | [**0.079 79**](javascript:showCorrelationPlot2(db='Illum_Retina_BXD_RankInv0410',ProbeSetID='ILMN_2839682',CellID='',db2='Illum_Retina_BXD_RankInv0410',ProbeSetID2='ILMN_2486573',CellID2='',rank='1')) |
|  | [**Trait 7: ILMN_3147135**](javascript:showDatabase2('Illum_Retina_BXD_RankInv0410','ILMN_3147135','');)  Symbol: Akr1a4  Akr1a4 | [**0.697 79**](javascript:showCorrelationPlot2(db='Illum_Retina_BXD_RankInv0410',ProbeSetID='ILMN_3147135',CellID='',db2='Illum_Retina_BXD_RankInv0410',ProbeSetID2='ILMN_2450384',CellID2='',rank='0')) | [**0.668 79**](javascript:showCorrelationPlot2(db='Illum_Retina_BXD_RankInv0410',ProbeSetID='ILMN_3147135',CellID='',db2='Illum_Retina_BXD_RankInv0410',ProbeSetID2='ILMN_3068754',CellID2='',rank='0')) | [**0.618 79**](javascript:showCorrelationPlot2(db='Illum_Retina_BXD_RankInv0410',ProbeSetID='ILMN_3147135',CellID='',db2='Illum_Retina_BXD_RankInv0410',ProbeSetID2='ILMN_2677092',CellID2='',rank='0')) | [**0.583 79**](javascript:showCorrelationPlot2(db='Illum_Retina_BXD_RankInv0410',ProbeSetID='ILMN_3147135',CellID='',db2='Illum_Retina_BXD_RankInv0410',ProbeSetID2='ILMN_2624938',CellID2='',rank='0')) | [**0.728 79**](javascript:showCorrelationPlot2(db='Illum_Retina_BXD_RankInv0410',ProbeSetID='ILMN_3147135',CellID='',db2='Illum_Retina_BXD_RankInv0410',ProbeSetID2='ILMN_2847269',CellID2='',rank='0')) | [**0.571 79**](javascript:showCorrelationPlot2(db='Illum_Retina_BXD_RankInv0410',ProbeSetID='ILMN_3147135',CellID='',db2='Illum_Retina_BXD_RankInv0410',ProbeSetID2='ILMN_2839682',CellID2='',rank='0')) | [***n* 79**](javascript:showDatabase2('Illum_Retina_BXD_RankInv0410','ILMN_3147135','')) | [**0.762 79**](javascript:showCorrelationPlot2(db='Illum_Retina_BXD_RankInv0410',ProbeSetID='ILMN_3147135',CellID='',db2='Illum_Retina_BXD_RankInv0410',ProbeSetID2='ILMN_1255823',CellID2='',rank='1')) | [**0.494 79**](javascript:showCorrelationPlot2(db='Illum_Retina_BXD_RankInv0410',ProbeSetID='ILMN_3147135',CellID='',db2='Illum_Retina_BXD_RankInv0410',ProbeSetID2='ILMN_2626143',CellID2='',rank='1')) | [**0.465 79**](javascript:showCorrelationPlot2(db='Illum_Retina_BXD_RankInv0410',ProbeSetID='ILMN_3147135',CellID='',db2='Illum_Retina_BXD_RankInv0410',ProbeSetID2='ILMN_1234930',CellID2='',rank='1')) | [**0.718 79**](javascript:showCorrelationPlot2(db='Illum_Retina_BXD_RankInv0410',ProbeSetID='ILMN_3147135',CellID='',db2='Illum_Retina_BXD_RankInv0410',ProbeSetID2='ILMN_1249408',CellID2='',rank='1')) | [**0.413 79**](javascript:showCorrelationPlot2(db='Illum_Retina_BXD_RankInv0410',ProbeSetID='ILMN_3147135',CellID='',db2='Illum_Retina_BXD_RankInv0410',ProbeSetID2='ILMN_2971559',CellID2='',rank='1')) | [**0.322 79**](javascript:showCorrelationPlot2(db='Illum_Retina_BXD_RankInv0410',ProbeSetID='ILMN_3147135',CellID='',db2='Illum_Retina_BXD_RankInv0410',ProbeSetID2='ILMN_1258455',CellID2='',rank='1')) | [**0.841 79**](javascript:showCorrelationPlot2(db='Illum_Retina_BXD_RankInv0410',ProbeSetID='ILMN_3147135',CellID='',db2='Illum_Retina_BXD_RankInv0410',ProbeSetID2='ILMN_2691613',CellID2='',rank='1')) | [**0.473 79**](javascript:showCorrelationPlot2(db='Illum_Retina_BXD_RankInv0410',ProbeSetID='ILMN_3147135',CellID='',db2='Illum_Retina_BXD_RankInv0410',ProbeSetID2='ILMN_1253600',CellID2='',rank='1')) | [**0.526 79**](javascript:showCorrelationPlot2(db='Illum_Retina_BXD_RankInv0410',ProbeSetID='ILMN_3147135',CellID='',db2='Illum_Retina_BXD_RankInv0410',ProbeSetID2='ILMN_2991545',CellID2='',rank='1')) | [**0.455 79**](javascript:showCorrelationPlot2(db='Illum_Retina_BXD_RankInv0410',ProbeSetID='ILMN_3147135',CellID='',db2='Illum_Retina_BXD_RankInv0410',ProbeSetID2='ILMN_2623591',CellID2='',rank='1')) | [**0.586 79**](javascript:showCorrelationPlot2(db='Illum_Retina_BXD_RankInv0410',ProbeSetID='ILMN_3147135',CellID='',db2='Illum_Retina_BXD_RankInv0410',ProbeSetID2='ILMN_2603568',CellID2='',rank='1')) | [**0.751 79**](javascript:showCorrelationPlot2(db='Illum_Retina_BXD_RankInv0410',ProbeSetID='ILMN_3147135',CellID='',db2='Illum_Retina_BXD_RankInv0410',ProbeSetID2='ILMN_2992541',CellID2='',rank='1')) | [**0.557 79**](javascript:showCorrelationPlot2(db='Illum_Retina_BXD_RankInv0410',ProbeSetID='ILMN_3147135',CellID='',db2='Illum_Retina_BXD_RankInv0410',ProbeSetID2='ILMN_2792868',CellID2='',rank='1')) | [**0.421 79**](javascript:showCorrelationPlot2(db='Illum_Retina_BXD_RankInv0410',ProbeSetID='ILMN_3147135',CellID='',db2='Illum_Retina_BXD_RankInv0410',ProbeSetID2='ILMN_3129497',CellID2='',rank='1')) | [**0.499 79**](javascript:showCorrelationPlot2(db='Illum_Retina_BXD_RankInv0410',ProbeSetID='ILMN_3147135',CellID='',db2='Illum_Retina_BXD_RankInv0410',ProbeSetID2='ILMN_1224034',CellID2='',rank='1')) | [**0.537 79**](javascript:showCorrelationPlot2(db='Illum_Retina_BXD_RankInv0410',ProbeSetID='ILMN_3147135',CellID='',db2='Illum_Retina_BXD_RankInv0410',ProbeSetID2='ILMN_2639819',CellID2='',rank='1')) | [**0.665 79**](javascript:showCorrelationPlot2(db='Illum_Retina_BXD_RankInv0410',ProbeSetID='ILMN_3147135',CellID='',db2='Illum_Retina_BXD_RankInv0410',ProbeSetID2='ILMN_2994779',CellID2='',rank='1')) | [**0.618 79**](javascript:showCorrelationPlot2(db='Illum_Retina_BXD_RankInv0410',ProbeSetID='ILMN_3147135',CellID='',db2='Illum_Retina_BXD_RankInv0410',ProbeSetID2='ILMN_2625047',CellID2='',rank='1')) | [**0.387 79**](javascript:showCorrelationPlot2(db='Illum_Retina_BXD_RankInv0410',ProbeSetID='ILMN_3147135',CellID='',db2='Illum_Retina_BXD_RankInv0410',ProbeSetID2='ILMN_2808186',CellID2='',rank='1')) | [**0.559 79**](javascript:showCorrelationPlot2(db='Illum_Retina_BXD_RankInv0410',ProbeSetID='ILMN_3147135',CellID='',db2='Illum_Retina_BXD_RankInv0410',ProbeSetID2='ILMN_2689056',CellID2='',rank='1')) | [**0.675 79**](javascript:showCorrelationPlot2(db='Illum_Retina_BXD_RankInv0410',ProbeSetID='ILMN_3147135',CellID='',db2='Illum_Retina_BXD_RankInv0410',ProbeSetID2='ILMN_2648386',CellID2='',rank='1')) | [**0.567 79**](javascript:showCorrelationPlot2(db='Illum_Retina_BXD_RankInv0410',ProbeSetID='ILMN_3147135',CellID='',db2='Illum_Retina_BXD_RankInv0410',ProbeSetID2='ILMN_2869225',CellID2='',rank='1')) | [**0.602 79**](javascript:showCorrelationPlot2(db='Illum_Retina_BXD_RankInv0410',ProbeSetID='ILMN_3147135',CellID='',db2='Illum_Retina_BXD_RankInv0410',ProbeSetID2='ILMN_2728431',CellID2='',rank='1')) | [**0.448 79**](javascript:showCorrelationPlot2(db='Illum_Retina_BXD_RankInv0410',ProbeSetID='ILMN_3147135',CellID='',db2='Illum_Retina_BXD_RankInv0410',ProbeSetID2='ILMN_3094608',CellID2='',rank='1')) | [**0.653 79**](javascript:showCorrelationPlot2(db='Illum_Retina_BXD_RankInv0410',ProbeSetID='ILMN_3147135',CellID='',db2='Illum_Retina_BXD_RankInv0410',ProbeSetID2='ILMN_2896528',CellID2='',rank='1')) | [**0.431 79**](javascript:showCorrelationPlot2(db='Illum_Retina_BXD_RankInv0410',ProbeSetID='ILMN_3147135',CellID='',db2='Illum_Retina_BXD_RankInv0410',ProbeSetID2='ILMN_2701750',CellID2='',rank='1')) | [**0.493 79**](javascript:showCorrelationPlot2(db='Illum_Retina_BXD_RankInv0410',ProbeSetID='ILMN_3147135',CellID='',db2='Illum_Retina_BXD_RankInv0410',ProbeSetID2='ILMN_1239448',CellID2='',rank='1')) | [**-0.741 79**](javascript:showCorrelationPlot2(db='Illum_Retina_BXD_RankInv0410',ProbeSetID='ILMN_3147135',CellID='',db2='Illum_Retina_BXD_RankInv0410',ProbeSetID2='ILMN_2583882',CellID2='',rank='1')) | [**0.493 79**](javascript:showCorrelationPlot2(db='Illum_Retina_BXD_RankInv0410',ProbeSetID='ILMN_3147135',CellID='',db2='Illum_Retina_BXD_RankInv0410',ProbeSetID2='ILMN_2915166',CellID2='',rank='1')) | [**0.331 79**](javascript:showCorrelationPlot2(db='Illum_Retina_BXD_RankInv0410',ProbeSetID='ILMN_3147135',CellID='',db2='Illum_Retina_BXD_RankInv0410',ProbeSetID2='ILMN_2702997',CellID2='',rank='1')) | [**0.334 79**](javascript:showCorrelationPlot2(db='Illum_Retina_BXD_RankInv0410',ProbeSetID='ILMN_3147135',CellID='',db2='Illum_Retina_BXD_RankInv0410',ProbeSetID2='ILMN_2435206',CellID2='',rank='1')) | [**0.548 79**](javascript:showCorrelationPlot2(db='Illum_Retina_BXD_RankInv0410',ProbeSetID='ILMN_3147135',CellID='',db2='Illum_Retina_BXD_RankInv0410',ProbeSetID2='ILMN_2789888',CellID2='',rank='1')) | [**0.397 79**](javascript:showCorrelationPlot2(db='Illum_Retina_BXD_RankInv0410',ProbeSetID='ILMN_3147135',CellID='',db2='Illum_Retina_BXD_RankInv0410',ProbeSetID2='ILMN_1218901',CellID2='',rank='1')) | [**0.613 79**](javascript:showCorrelationPlot2(db='Illum_Retina_BXD_RankInv0410',ProbeSetID='ILMN_3147135',CellID='',db2='Illum_Retina_BXD_RankInv0410',ProbeSetID2='ILMN_1254734',CellID2='',rank='1')) | [**0.470 79**](javascript:showCorrelationPlot2(db='Illum_Retina_BXD_RankInv0410',ProbeSetID='ILMN_3147135',CellID='',db2='Illum_Retina_BXD_RankInv0410',ProbeSetID2='ILMN_3008924',CellID2='',rank='1')) | [**0.637 79**](javascript:showCorrelationPlot2(db='Illum_Retina_BXD_RankInv0410',ProbeSetID='ILMN_3147135',CellID='',db2='Illum_Retina_BXD_RankInv0410',ProbeSetID2='ILMN_2628175',CellID2='',rank='1')) | [**0.652 79**](javascript:showCorrelationPlot2(db='Illum_Retina_BXD_RankInv0410',ProbeSetID='ILMN_3147135',CellID='',db2='Illum_Retina_BXD_RankInv0410',ProbeSetID2='ILMN_2891573',CellID2='',rank='1')) | [**0.526 79**](javascript:showCorrelationPlot2(db='Illum_Retina_BXD_RankInv0410',ProbeSetID='ILMN_3147135',CellID='',db2='Illum_Retina_BXD_RankInv0410',ProbeSetID2='ILMN_1220846',CellID2='',rank='1')) | [**0.642 79**](javascript:showCorrelationPlot2(db='Illum_Retina_BXD_RankInv0410',ProbeSetID='ILMN_3147135',CellID='',db2='Illum_Retina_BXD_RankInv0410',ProbeSetID2='ILMN_1241962',CellID2='',rank='1')) | [**0.605 79**](javascript:showCorrelationPlot2(db='Illum_Retina_BXD_RankInv0410',ProbeSetID='ILMN_3147135',CellID='',db2='Illum_Retina_BXD_RankInv0410',ProbeSetID2='ILMN_3163027',CellID2='',rank='1')) | [**0.484 79**](javascript:showCorrelationPlot2(db='Illum_Retina_BXD_RankInv0410',ProbeSetID='ILMN_3147135',CellID='',db2='Illum_Retina_BXD_RankInv0410',ProbeSetID2='ILMN_2775098',CellID2='',rank='1')) | [**0.507 79**](javascript:showCorrelationPlot2(db='Illum_Retina_BXD_RankInv0410',ProbeSetID='ILMN_3147135',CellID='',db2='Illum_Retina_BXD_RankInv0410',ProbeSetID2='ILMN_2752552',CellID2='',rank='1')) | [**0.453 79**](javascript:showCorrelationPlot2(db='Illum_Retina_BXD_RankInv0410',ProbeSetID='ILMN_3147135',CellID='',db2='Illum_Retina_BXD_RankInv0410',ProbeSetID2='ILMN_2730293',CellID2='',rank='1')) | [**0.358 79**](javascript:showCorrelationPlot2(db='Illum_Retina_BXD_RankInv0410',ProbeSetID='ILMN_3147135',CellID='',db2='Illum_Retina_BXD_RankInv0410',ProbeSetID2='ILMN_2680872',CellID2='',rank='1')) | [**0.253 79**](javascript:showCorrelationPlot2(db='Illum_Retina_BXD_RankInv0410',ProbeSetID='ILMN_3147135',CellID='',db2='Illum_Retina_BXD_RankInv0410',ProbeSetID2='ILMN_2768533',CellID2='',rank='1')) | [**0.024 79**](javascript:showCorrelationPlot2(db='Illum_Retina_BXD_RankInv0410',ProbeSetID='ILMN_3147135',CellID='',db2='Illum_Retina_BXD_RankInv0410',ProbeSetID2='ILMN_3121255',CellID2='',rank='1')) | [**-0.381 79**](javascript:showCorrelationPlot2(db='Illum_Retina_BXD_RankInv0410',ProbeSetID='ILMN_3147135',CellID='',db2='Illum_Retina_BXD_RankInv0410',ProbeSetID2='ILMN_2484527',CellID2='',rank='1')) | [**0.449 79**](javascript:showCorrelationPlot2(db='Illum_Retina_BXD_RankInv0410',ProbeSetID='ILMN_3147135',CellID='',db2='Illum_Retina_BXD_RankInv0410',ProbeSetID2='ILMN_3045723',CellID2='',rank='1')) | [**0.075 79**](javascript:showCorrelationPlot2(db='Illum_Retina_BXD_RankInv0410',ProbeSetID='ILMN_3147135',CellID='',db2='Illum_Retina_BXD_RankInv0410',ProbeSetID2='ILMN_2486573',CellID2='',rank='1')) |
|  | [**Trait 8: ILMN_1255823**](javascript:showDatabase2('Illum_Retina_BXD_RankInv0410','ILMN_1255823','');)  Symbol: Higd2a  Higd2a | [**0.695 79**](javascript:showCorrelationPlot2(db='Illum_Retina_BXD_RankInv0410',ProbeSetID='ILMN_1255823',CellID='',db2='Illum_Retina_BXD_RankInv0410',ProbeSetID2='ILMN_2450384',CellID2='',rank='0')) | [**0.602 79**](javascript:showCorrelationPlot2(db='Illum_Retina_BXD_RankInv0410',ProbeSetID='ILMN_1255823',CellID='',db2='Illum_Retina_BXD_RankInv0410',ProbeSetID2='ILMN_3068754',CellID2='',rank='0')) | [**0.599 79**](javascript:showCorrelationPlot2(db='Illum_Retina_BXD_RankInv0410',ProbeSetID='ILMN_1255823',CellID='',db2='Illum_Retina_BXD_RankInv0410',ProbeSetID2='ILMN_2677092',CellID2='',rank='0')) | [**0.577 79**](javascript:showCorrelationPlot2(db='Illum_Retina_BXD_RankInv0410',ProbeSetID='ILMN_1255823',CellID='',db2='Illum_Retina_BXD_RankInv0410',ProbeSetID2='ILMN_2624938',CellID2='',rank='0')) | [**0.658 79**](javascript:showCorrelationPlot2(db='Illum_Retina_BXD_RankInv0410',ProbeSetID='ILMN_1255823',CellID='',db2='Illum_Retina_BXD_RankInv0410',ProbeSetID2='ILMN_2847269',CellID2='',rank='0')) | [**0.400 79**](javascript:showCorrelationPlot2(db='Illum_Retina_BXD_RankInv0410',ProbeSetID='ILMN_1255823',CellID='',db2='Illum_Retina_BXD_RankInv0410',ProbeSetID2='ILMN_2839682',CellID2='',rank='0')) | [**0.712 79**](javascript:showCorrelationPlot2(db='Illum_Retina_BXD_RankInv0410',ProbeSetID='ILMN_1255823',CellID='',db2='Illum_Retina_BXD_RankInv0410',ProbeSetID2='ILMN_3147135',CellID2='',rank='0')) | [***n* 79**](javascript:showDatabase2('Illum_Retina_BXD_RankInv0410','ILMN_1255823','')) | [**0.623 79**](javascript:showCorrelationPlot2(db='Illum_Retina_BXD_RankInv0410',ProbeSetID='ILMN_1255823',CellID='',db2='Illum_Retina_BXD_RankInv0410',ProbeSetID2='ILMN_2626143',CellID2='',rank='1')) | [**0.589 79**](javascript:showCorrelationPlot2(db='Illum_Retina_BXD_RankInv0410',ProbeSetID='ILMN_1255823',CellID='',db2='Illum_Retina_BXD_RankInv0410',ProbeSetID2='ILMN_1234930',CellID2='',rank='1')) | [**0.834 79**](javascript:showCorrelationPlot2(db='Illum_Retina_BXD_RankInv0410',ProbeSetID='ILMN_1255823',CellID='',db2='Illum_Retina_BXD_RankInv0410',ProbeSetID2='ILMN_1249408',CellID2='',rank='1')) | [**0.478 79**](javascript:showCorrelationPlot2(db='Illum_Retina_BXD_RankInv0410',ProbeSetID='ILMN_1255823',CellID='',db2='Illum_Retina_BXD_RankInv0410',ProbeSetID2='ILMN_2971559',CellID2='',rank='1')) | [**0.470 79**](javascript:showCorrelationPlot2(db='Illum_Retina_BXD_RankInv0410',ProbeSetID='ILMN_1255823',CellID='',db2='Illum_Retina_BXD_RankInv0410',ProbeSetID2='ILMN_1258455',CellID2='',rank='1')) | [**0.787 79**](javascript:showCorrelationPlot2(db='Illum_Retina_BXD_RankInv0410',ProbeSetID='ILMN_1255823',CellID='',db2='Illum_Retina_BXD_RankInv0410',ProbeSetID2='ILMN_2691613',CellID2='',rank='1')) | [**0.494 79**](javascript:showCorrelationPlot2(db='Illum_Retina_BXD_RankInv0410',ProbeSetID='ILMN_1255823',CellID='',db2='Illum_Retina_BXD_RankInv0410',ProbeSetID2='ILMN_1253600',CellID2='',rank='1')) | [**0.558 79**](javascript:showCorrelationPlot2(db='Illum_Retina_BXD_RankInv0410',ProbeSetID='ILMN_1255823',CellID='',db2='Illum_Retina_BXD_RankInv0410',ProbeSetID2='ILMN_2991545',CellID2='',rank='1')) | [**0.488 79**](javascript:showCorrelationPlot2(db='Illum_Retina_BXD_RankInv0410',ProbeSetID='ILMN_1255823',CellID='',db2='Illum_Retina_BXD_RankInv0410',ProbeSetID2='ILMN_2623591',CellID2='',rank='1')) | [**0.652 79**](javascript:showCorrelationPlot2(db='Illum_Retina_BXD_RankInv0410',ProbeSetID='ILMN_1255823',CellID='',db2='Illum_Retina_BXD_RankInv0410',ProbeSetID2='ILMN_2603568',CellID2='',rank='1')) | [**0.729 79**](javascript:showCorrelationPlot2(db='Illum_Retina_BXD_RankInv0410',ProbeSetID='ILMN_1255823',CellID='',db2='Illum_Retina_BXD_RankInv0410',ProbeSetID2='ILMN_2992541',CellID2='',rank='1')) | [**0.564 79**](javascript:showCorrelationPlot2(db='Illum_Retina_BXD_RankInv0410',ProbeSetID='ILMN_1255823',CellID='',db2='Illum_Retina_BXD_RankInv0410',ProbeSetID2='ILMN_2792868',CellID2='',rank='1')) | [**0.460 79**](javascript:showCorrelationPlot2(db='Illum_Retina_BXD_RankInv0410',ProbeSetID='ILMN_1255823',CellID='',db2='Illum_Retina_BXD_RankInv0410',ProbeSetID2='ILMN_3129497',CellID2='',rank='1')) | [**0.441 79**](javascript:showCorrelationPlot2(db='Illum_Retina_BXD_RankInv0410',ProbeSetID='ILMN_1255823',CellID='',db2='Illum_Retina_BXD_RankInv0410',ProbeSetID2='ILMN_1224034',CellID2='',rank='1')) | [**0.549 79**](javascript:showCorrelationPlot2(db='Illum_Retina_BXD_RankInv0410',ProbeSetID='ILMN_1255823',CellID='',db2='Illum_Retina_BXD_RankInv0410',ProbeSetID2='ILMN_2639819',CellID2='',rank='1')) | [**0.572 79**](javascript:showCorrelationPlot2(db='Illum_Retina_BXD_RankInv0410',ProbeSetID='ILMN_1255823',CellID='',db2='Illum_Retina_BXD_RankInv0410',ProbeSetID2='ILMN_2994779',CellID2='',rank='1')) | [**0.505 79**](javascript:showCorrelationPlot2(db='Illum_Retina_BXD_RankInv0410',ProbeSetID='ILMN_1255823',CellID='',db2='Illum_Retina_BXD_RankInv0410',ProbeSetID2='ILMN_2625047',CellID2='',rank='1')) | [**0.360 79**](javascript:showCorrelationPlot2(db='Illum_Retina_BXD_RankInv0410',ProbeSetID='ILMN_1255823',CellID='',db2='Illum_Retina_BXD_RankInv0410',ProbeSetID2='ILMN_2808186',CellID2='',rank='1')) | [**0.624 79**](javascript:showCorrelationPlot2(db='Illum_Retina_BXD_RankInv0410',ProbeSetID='ILMN_1255823',CellID='',db2='Illum_Retina_BXD_RankInv0410',ProbeSetID2='ILMN_2689056',CellID2='',rank='1')) | [**0.676 79**](javascript:showCorrelationPlot2(db='Illum_Retina_BXD_RankInv0410',ProbeSetID='ILMN_1255823',CellID='',db2='Illum_Retina_BXD_RankInv0410',ProbeSetID2='ILMN_2648386',CellID2='',rank='1')) | [**0.690 79**](javascript:showCorrelationPlot2(db='Illum_Retina_BXD_RankInv0410',ProbeSetID='ILMN_1255823',CellID='',db2='Illum_Retina_BXD_RankInv0410',ProbeSetID2='ILMN_2869225',CellID2='',rank='1')) | [**0.581 79**](javascript:showCorrelationPlot2(db='Illum_Retina_BXD_RankInv0410',ProbeSetID='ILMN_1255823',CellID='',db2='Illum_Retina_BXD_RankInv0410',ProbeSetID2='ILMN_2728431',CellID2='',rank='1')) | [**0.411 79**](javascript:showCorrelationPlot2(db='Illum_Retina_BXD_RankInv0410',ProbeSetID='ILMN_1255823',CellID='',db2='Illum_Retina_BXD_RankInv0410',ProbeSetID2='ILMN_3094608',CellID2='',rank='1')) | [**0.685 79**](javascript:showCorrelationPlot2(db='Illum_Retina_BXD_RankInv0410',ProbeSetID='ILMN_1255823',CellID='',db2='Illum_Retina_BXD_RankInv0410',ProbeSetID2='ILMN_2896528',CellID2='',rank='1')) | [**0.441 79**](javascript:showCorrelationPlot2(db='Illum_Retina_BXD_RankInv0410',ProbeSetID='ILMN_1255823',CellID='',db2='Illum_Retina_BXD_RankInv0410',ProbeSetID2='ILMN_2701750',CellID2='',rank='1')) | [**0.553 79**](javascript:showCorrelationPlot2(db='Illum_Retina_BXD_RankInv0410',ProbeSetID='ILMN_1255823',CellID='',db2='Illum_Retina_BXD_RankInv0410',ProbeSetID2='ILMN_1239448',CellID2='',rank='1')) | [**-0.668 79**](javascript:showCorrelationPlot2(db='Illum_Retina_BXD_RankInv0410',ProbeSetID='ILMN_1255823',CellID='',db2='Illum_Retina_BXD_RankInv0410',ProbeSetID2='ILMN_2583882',CellID2='',rank='1')) | [**0.519 79**](javascript:showCorrelationPlot2(db='Illum_Retina_BXD_RankInv0410',ProbeSetID='ILMN_1255823',CellID='',db2='Illum_Retina_BXD_RankInv0410',ProbeSetID2='ILMN_2915166',CellID2='',rank='1')) | [**0.428 79**](javascript:showCorrelationPlot2(db='Illum_Retina_BXD_RankInv0410',ProbeSetID='ILMN_1255823',CellID='',db2='Illum_Retina_BXD_RankInv0410',ProbeSetID2='ILMN_2702997',CellID2='',rank='1')) | [**0.497 79**](javascript:showCorrelationPlot2(db='Illum_Retina_BXD_RankInv0410',ProbeSetID='ILMN_1255823',CellID='',db2='Illum_Retina_BXD_RankInv0410',ProbeSetID2='ILMN_2435206',CellID2='',rank='1')) | [**0.523 79**](javascript:showCorrelationPlot2(db='Illum_Retina_BXD_RankInv0410',ProbeSetID='ILMN_1255823',CellID='',db2='Illum_Retina_BXD_RankInv0410',ProbeSetID2='ILMN_2789888',CellID2='',rank='1')) | [**0.462 79**](javascript:showCorrelationPlot2(db='Illum_Retina_BXD_RankInv0410',ProbeSetID='ILMN_1255823',CellID='',db2='Illum_Retina_BXD_RankInv0410',ProbeSetID2='ILMN_1218901',CellID2='',rank='1')) | [**0.811 79**](javascript:showCorrelationPlot2(db='Illum_Retina_BXD_RankInv0410',ProbeSetID='ILMN_1255823',CellID='',db2='Illum_Retina_BXD_RankInv0410',ProbeSetID2='ILMN_1254734',CellID2='',rank='1')) | [**0.372 79**](javascript:showCorrelationPlot2(db='Illum_Retina_BXD_RankInv0410',ProbeSetID='ILMN_1255823',CellID='',db2='Illum_Retina_BXD_RankInv0410',ProbeSetID2='ILMN_3008924',CellID2='',rank='1')) | [**0.596 79**](javascript:showCorrelationPlot2(db='Illum_Retina_BXD_RankInv0410',ProbeSetID='ILMN_1255823',CellID='',db2='Illum_Retina_BXD_RankInv0410',ProbeSetID2='ILMN_2628175',CellID2='',rank='1')) | [**0.608 79**](javascript:showCorrelationPlot2(db='Illum_Retina_BXD_RankInv0410',ProbeSetID='ILMN_1255823',CellID='',db2='Illum_Retina_BXD_RankInv0410',ProbeSetID2='ILMN_2891573',CellID2='',rank='1')) | [**0.638 79**](javascript:showCorrelationPlot2(db='Illum_Retina_BXD_RankInv0410',ProbeSetID='ILMN_1255823',CellID='',db2='Illum_Retina_BXD_RankInv0410',ProbeSetID2='ILMN_1220846',CellID2='',rank='1')) | [**0.746 79**](javascript:showCorrelationPlot2(db='Illum_Retina_BXD_RankInv0410',ProbeSetID='ILMN_1255823',CellID='',db2='Illum_Retina_BXD_RankInv0410',ProbeSetID2='ILMN_1241962',CellID2='',rank='1')) | [**0.658 79**](javascript:showCorrelationPlot2(db='Illum_Retina_BXD_RankInv0410',ProbeSetID='ILMN_1255823',CellID='',db2='Illum_Retina_BXD_RankInv0410',ProbeSetID2='ILMN_3163027',CellID2='',rank='1')) | [**0.542 79**](javascript:showCorrelationPlot2(db='Illum_Retina_BXD_RankInv0410',ProbeSetID='ILMN_1255823',CellID='',db2='Illum_Retina_BXD_RankInv0410',ProbeSetID2='ILMN_2775098',CellID2='',rank='1')) | [**0.592 79**](javascript:showCorrelationPlot2(db='Illum_Retina_BXD_RankInv0410',ProbeSetID='ILMN_1255823',CellID='',db2='Illum_Retina_BXD_RankInv0410',ProbeSetID2='ILMN_2752552',CellID2='',rank='1')) | [**0.440 79**](javascript:showCorrelationPlot2(db='Illum_Retina_BXD_RankInv0410',ProbeSetID='ILMN_1255823',CellID='',db2='Illum_Retina_BXD_RankInv0410',ProbeSetID2='ILMN_2730293',CellID2='',rank='1')) | [**0.369 79**](javascript:showCorrelationPlot2(db='Illum_Retina_BXD_RankInv0410',ProbeSetID='ILMN_1255823',CellID='',db2='Illum_Retina_BXD_RankInv0410',ProbeSetID2='ILMN_2680872',CellID2='',rank='1')) | [**0.204 79**](javascript:showCorrelationPlot2(db='Illum_Retina_BXD_RankInv0410',ProbeSetID='ILMN_1255823',CellID='',db2='Illum_Retina_BXD_RankInv0410',ProbeSetID2='ILMN_2768533',CellID2='',rank='1')) | [**-0.052 79**](javascript:showCorrelationPlot2(db='Illum_Retina_BXD_RankInv0410',ProbeSetID='ILMN_1255823',CellID='',db2='Illum_Retina_BXD_RankInv0410',ProbeSetID2='ILMN_3121255',CellID2='',rank='1')) | [**-0.456 79**](javascript:showCorrelationPlot2(db='Illum_Retina_BXD_RankInv0410',ProbeSetID='ILMN_1255823',CellID='',db2='Illum_Retina_BXD_RankInv0410',ProbeSetID2='ILMN_2484527',CellID2='',rank='1')) | [**0.311 79**](javascript:showCorrelationPlot2(db='Illum_Retina_BXD_RankInv0410',ProbeSetID='ILMN_1255823',CellID='',db2='Illum_Retina_BXD_RankInv0410',ProbeSetID2='ILMN_3045723',CellID2='',rank='1')) | [**0.110 79**](javascript:showCorrelationPlot2(db='Illum_Retina_BXD_RankInv0410',ProbeSetID='ILMN_1255823',CellID='',db2='Illum_Retina_BXD_RankInv0410',ProbeSetID2='ILMN_2486573',CellID2='',rank='1')) |
|  | [**Trait 9: ILMN_2626143**](javascript:showDatabase2('Illum_Retina_BXD_RankInv0410','ILMN_2626143','');)  Symbol: Phpt1  Phpt1 | [**0.694 79**](javascript:showCorrelationPlot2(db='Illum_Retina_BXD_RankInv0410',ProbeSetID='ILMN_2626143',CellID='',db2='Illum_Retina_BXD_RankInv0410',ProbeSetID2='ILMN_2450384',CellID2='',rank='0')) | [**0.731 79**](javascript:showCorrelationPlot2(db='Illum_Retina_BXD_RankInv0410',ProbeSetID='ILMN_2626143',CellID='',db2='Illum_Retina_BXD_RankInv0410',ProbeSetID2='ILMN_3068754',CellID2='',rank='0')) | [**0.627 79**](javascript:showCorrelationPlot2(db='Illum_Retina_BXD_RankInv0410',ProbeSetID='ILMN_2626143',CellID='',db2='Illum_Retina_BXD_RankInv0410',ProbeSetID2='ILMN_2677092',CellID2='',rank='0')) | [**0.764 79**](javascript:showCorrelationPlot2(db='Illum_Retina_BXD_RankInv0410',ProbeSetID='ILMN_2626143',CellID='',db2='Illum_Retina_BXD_RankInv0410',ProbeSetID2='ILMN_2624938',CellID2='',rank='0')) | [**0.558 79**](javascript:showCorrelationPlot2(db='Illum_Retina_BXD_RankInv0410',ProbeSetID='ILMN_2626143',CellID='',db2='Illum_Retina_BXD_RankInv0410',ProbeSetID2='ILMN_2847269',CellID2='',rank='0')) | [**0.515 79**](javascript:showCorrelationPlot2(db='Illum_Retina_BXD_RankInv0410',ProbeSetID='ILMN_2626143',CellID='',db2='Illum_Retina_BXD_RankInv0410',ProbeSetID2='ILMN_2839682',CellID2='',rank='0')) | [**0.474 79**](javascript:showCorrelationPlot2(db='Illum_Retina_BXD_RankInv0410',ProbeSetID='ILMN_2626143',CellID='',db2='Illum_Retina_BXD_RankInv0410',ProbeSetID2='ILMN_3147135',CellID2='',rank='0')) | [**0.653 79**](javascript:showCorrelationPlot2(db='Illum_Retina_BXD_RankInv0410',ProbeSetID='ILMN_2626143',CellID='',db2='Illum_Retina_BXD_RankInv0410',ProbeSetID2='ILMN_1255823',CellID2='',rank='0')) | [***n* 79**](javascript:showDatabase2('Illum_Retina_BXD_RankInv0410','ILMN_2626143','')) | [**0.706 79**](javascript:showCorrelationPlot2(db='Illum_Retina_BXD_RankInv0410',ProbeSetID='ILMN_2626143',CellID='',db2='Illum_Retina_BXD_RankInv0410',ProbeSetID2='ILMN_1234930',CellID2='',rank='1')) | [**0.659 79**](javascript:showCorrelationPlot2(db='Illum_Retina_BXD_RankInv0410',ProbeSetID='ILMN_2626143',CellID='',db2='Illum_Retina_BXD_RankInv0410',ProbeSetID2='ILMN_1249408',CellID2='',rank='1')) | [**0.590 79**](javascript:showCorrelationPlot2(db='Illum_Retina_BXD_RankInv0410',ProbeSetID='ILMN_2626143',CellID='',db2='Illum_Retina_BXD_RankInv0410',ProbeSetID2='ILMN_2971559',CellID2='',rank='1')) | [**0.598 79**](javascript:showCorrelationPlot2(db='Illum_Retina_BXD_RankInv0410',ProbeSetID='ILMN_2626143',CellID='',db2='Illum_Retina_BXD_RankInv0410',ProbeSetID2='ILMN_1258455',CellID2='',rank='1')) | [**0.512 79**](javascript:showCorrelationPlot2(db='Illum_Retina_BXD_RankInv0410',ProbeSetID='ILMN_2626143',CellID='',db2='Illum_Retina_BXD_RankInv0410',ProbeSetID2='ILMN_2691613',CellID2='',rank='1')) | [**0.482 79**](javascript:showCorrelationPlot2(db='Illum_Retina_BXD_RankInv0410',ProbeSetID='ILMN_2626143',CellID='',db2='Illum_Retina_BXD_RankInv0410',ProbeSetID2='ILMN_1253600',CellID2='',rank='1')) | [**0.668 79**](javascript:showCorrelationPlot2(db='Illum_Retina_BXD_RankInv0410',ProbeSetID='ILMN_2626143',CellID='',db2='Illum_Retina_BXD_RankInv0410',ProbeSetID2='ILMN_2991545',CellID2='',rank='1')) | [**0.570 79**](javascript:showCorrelationPlot2(db='Illum_Retina_BXD_RankInv0410',ProbeSetID='ILMN_2626143',CellID='',db2='Illum_Retina_BXD_RankInv0410',ProbeSetID2='ILMN_2623591',CellID2='',rank='1')) | [**0.448 79**](javascript:showCorrelationPlot2(db='Illum_Retina_BXD_RankInv0410',ProbeSetID='ILMN_2626143',CellID='',db2='Illum_Retina_BXD_RankInv0410',ProbeSetID2='ILMN_2603568',CellID2='',rank='1')) | [**0.571 79**](javascript:showCorrelationPlot2(db='Illum_Retina_BXD_RankInv0410',ProbeSetID='ILMN_2626143',CellID='',db2='Illum_Retina_BXD_RankInv0410',ProbeSetID2='ILMN_2992541',CellID2='',rank='1')) | [**0.476 79**](javascript:showCorrelationPlot2(db='Illum_Retina_BXD_RankInv0410',ProbeSetID='ILMN_2626143',CellID='',db2='Illum_Retina_BXD_RankInv0410',ProbeSetID2='ILMN_2792868',CellID2='',rank='1')) | [**0.573 79**](javascript:showCorrelationPlot2(db='Illum_Retina_BXD_RankInv0410',ProbeSetID='ILMN_2626143',CellID='',db2='Illum_Retina_BXD_RankInv0410',ProbeSetID2='ILMN_3129497',CellID2='',rank='1')) | [**0.454 79**](javascript:showCorrelationPlot2(db='Illum_Retina_BXD_RankInv0410',ProbeSetID='ILMN_2626143',CellID='',db2='Illum_Retina_BXD_RankInv0410',ProbeSetID2='ILMN_1224034',CellID2='',rank='1')) | [**0.657 79**](javascript:showCorrelationPlot2(db='Illum_Retina_BXD_RankInv0410',ProbeSetID='ILMN_2626143',CellID='',db2='Illum_Retina_BXD_RankInv0410',ProbeSetID2='ILMN_2639819',CellID2='',rank='1')) | [**0.609 79**](javascript:showCorrelationPlot2(db='Illum_Retina_BXD_RankInv0410',ProbeSetID='ILMN_2626143',CellID='',db2='Illum_Retina_BXD_RankInv0410',ProbeSetID2='ILMN_2994779',CellID2='',rank='1')) | [**0.509 79**](javascript:showCorrelationPlot2(db='Illum_Retina_BXD_RankInv0410',ProbeSetID='ILMN_2626143',CellID='',db2='Illum_Retina_BXD_RankInv0410',ProbeSetID2='ILMN_2625047',CellID2='',rank='1')) | [**0.445 79**](javascript:showCorrelationPlot2(db='Illum_Retina_BXD_RankInv0410',ProbeSetID='ILMN_2626143',CellID='',db2='Illum_Retina_BXD_RankInv0410',ProbeSetID2='ILMN_2808186',CellID2='',rank='1')) | [**0.634 79**](javascript:showCorrelationPlot2(db='Illum_Retina_BXD_RankInv0410',ProbeSetID='ILMN_2626143',CellID='',db2='Illum_Retina_BXD_RankInv0410',ProbeSetID2='ILMN_2689056',CellID2='',rank='1')) | [**0.556 79**](javascript:showCorrelationPlot2(db='Illum_Retina_BXD_RankInv0410',ProbeSetID='ILMN_2626143',CellID='',db2='Illum_Retina_BXD_RankInv0410',ProbeSetID2='ILMN_2648386',CellID2='',rank='1')) | [**0.704 79**](javascript:showCorrelationPlot2(db='Illum_Retina_BXD_RankInv0410',ProbeSetID='ILMN_2626143',CellID='',db2='Illum_Retina_BXD_RankInv0410',ProbeSetID2='ILMN_2869225',CellID2='',rank='1')) | [**0.502 79**](javascript:showCorrelationPlot2(db='Illum_Retina_BXD_RankInv0410',ProbeSetID='ILMN_2626143',CellID='',db2='Illum_Retina_BXD_RankInv0410',ProbeSetID2='ILMN_2728431',CellID2='',rank='1')) | [**0.621 79**](javascript:showCorrelationPlot2(db='Illum_Retina_BXD_RankInv0410',ProbeSetID='ILMN_2626143',CellID='',db2='Illum_Retina_BXD_RankInv0410',ProbeSetID2='ILMN_3094608',CellID2='',rank='1')) | [**0.488 79**](javascript:showCorrelationPlot2(db='Illum_Retina_BXD_RankInv0410',ProbeSetID='ILMN_2626143',CellID='',db2='Illum_Retina_BXD_RankInv0410',ProbeSetID2='ILMN_2896528',CellID2='',rank='1')) | [**0.445 79**](javascript:showCorrelationPlot2(db='Illum_Retina_BXD_RankInv0410',ProbeSetID='ILMN_2626143',CellID='',db2='Illum_Retina_BXD_RankInv0410',ProbeSetID2='ILMN_2701750',CellID2='',rank='1')) | [**0.651 79**](javascript:showCorrelationPlot2(db='Illum_Retina_BXD_RankInv0410',ProbeSetID='ILMN_2626143',CellID='',db2='Illum_Retina_BXD_RankInv0410',ProbeSetID2='ILMN_1239448',CellID2='',rank='1')) | [**-0.294 79**](javascript:showCorrelationPlot2(db='Illum_Retina_BXD_RankInv0410',ProbeSetID='ILMN_2626143',CellID='',db2='Illum_Retina_BXD_RankInv0410',ProbeSetID2='ILMN_2583882',CellID2='',rank='1')) | [**0.604 79**](javascript:showCorrelationPlot2(db='Illum_Retina_BXD_RankInv0410',ProbeSetID='ILMN_2626143',CellID='',db2='Illum_Retina_BXD_RankInv0410',ProbeSetID2='ILMN_2915166',CellID2='',rank='1')) | [**0.529 79**](javascript:showCorrelationPlot2(db='Illum_Retina_BXD_RankInv0410',ProbeSetID='ILMN_2626143',CellID='',db2='Illum_Retina_BXD_RankInv0410',ProbeSetID2='ILMN_2702997',CellID2='',rank='1')) | [**0.647 79**](javascript:showCorrelationPlot2(db='Illum_Retina_BXD_RankInv0410',ProbeSetID='ILMN_2626143',CellID='',db2='Illum_Retina_BXD_RankInv0410',ProbeSetID2='ILMN_2435206',CellID2='',rank='1')) | [**0.461 79**](javascript:showCorrelationPlot2(db='Illum_Retina_BXD_RankInv0410',ProbeSetID='ILMN_2626143',CellID='',db2='Illum_Retina_BXD_RankInv0410',ProbeSetID2='ILMN_2789888',CellID2='',rank='1')) | [**0.702 79**](javascript:showCorrelationPlot2(db='Illum_Retina_BXD_RankInv0410',ProbeSetID='ILMN_2626143',CellID='',db2='Illum_Retina_BXD_RankInv0410',ProbeSetID2='ILMN_1218901',CellID2='',rank='1')) | [**0.593 79**](javascript:showCorrelationPlot2(db='Illum_Retina_BXD_RankInv0410',ProbeSetID='ILMN_2626143',CellID='',db2='Illum_Retina_BXD_RankInv0410',ProbeSetID2='ILMN_1254734',CellID2='',rank='1')) | [**0.455 79**](javascript:showCorrelationPlot2(db='Illum_Retina_BXD_RankInv0410',ProbeSetID='ILMN_2626143',CellID='',db2='Illum_Retina_BXD_RankInv0410',ProbeSetID2='ILMN_3008924',CellID2='',rank='1')) | [**0.503 79**](javascript:showCorrelationPlot2(db='Illum_Retina_BXD_RankInv0410',ProbeSetID='ILMN_2626143',CellID='',db2='Illum_Retina_BXD_RankInv0410',ProbeSetID2='ILMN_2628175',CellID2='',rank='1')) | [**0.657 79**](javascript:showCorrelationPlot2(db='Illum_Retina_BXD_RankInv0410',ProbeSetID='ILMN_2626143',CellID='',db2='Illum_Retina_BXD_RankInv0410',ProbeSetID2='ILMN_2891573',CellID2='',rank='1')) | [**0.561 79**](javascript:showCorrelationPlot2(db='Illum_Retina_BXD_RankInv0410',ProbeSetID='ILMN_2626143',CellID='',db2='Illum_Retina_BXD_RankInv0410',ProbeSetID2='ILMN_1220846',CellID2='',rank='1')) | [**0.541 79**](javascript:showCorrelationPlot2(db='Illum_Retina_BXD_RankInv0410',ProbeSetID='ILMN_2626143',CellID='',db2='Illum_Retina_BXD_RankInv0410',ProbeSetID2='ILMN_1241962',CellID2='',rank='1')) | [**0.515 79**](javascript:showCorrelationPlot2(db='Illum_Retina_BXD_RankInv0410',ProbeSetID='ILMN_2626143',CellID='',db2='Illum_Retina_BXD_RankInv0410',ProbeSetID2='ILMN_3163027',CellID2='',rank='1')) | [**0.418 79**](javascript:showCorrelationPlot2(db='Illum_Retina_BXD_RankInv0410',ProbeSetID='ILMN_2626143',CellID='',db2='Illum_Retina_BXD_RankInv0410',ProbeSetID2='ILMN_2775098',CellID2='',rank='1')) | [**0.848 79**](javascript:showCorrelationPlot2(db='Illum_Retina_BXD_RankInv0410',ProbeSetID='ILMN_2626143',CellID='',db2='Illum_Retina_BXD_RankInv0410',ProbeSetID2='ILMN_2752552',CellID2='',rank='1')) | [**0.492 79**](javascript:showCorrelationPlot2(db='Illum_Retina_BXD_RankInv0410',ProbeSetID='ILMN_2626143',CellID='',db2='Illum_Retina_BXD_RankInv0410',ProbeSetID2='ILMN_2730293',CellID2='',rank='1')) | [**0.479 79**](javascript:showCorrelationPlot2(db='Illum_Retina_BXD_RankInv0410',ProbeSetID='ILMN_2626143',CellID='',db2='Illum_Retina_BXD_RankInv0410',ProbeSetID2='ILMN_2680872',CellID2='',rank='1')) | [**0.235 79**](javascript:showCorrelationPlot2(db='Illum_Retina_BXD_RankInv0410',ProbeSetID='ILMN_2626143',CellID='',db2='Illum_Retina_BXD_RankInv0410',ProbeSetID2='ILMN_2768533',CellID2='',rank='1')) | [**-0.380 79**](javascript:showCorrelationPlot2(db='Illum_Retina_BXD_RankInv0410',ProbeSetID='ILMN_2626143',CellID='',db2='Illum_Retina_BXD_RankInv0410',ProbeSetID2='ILMN_3121255',CellID2='',rank='1')) | [**-0.344 79**](javascript:showCorrelationPlot2(db='Illum_Retina_BXD_RankInv0410',ProbeSetID='ILMN_2626143',CellID='',db2='Illum_Retina_BXD_RankInv0410',ProbeSetID2='ILMN_2484527',CellID2='',rank='1')) | [**0.119 79**](javascript:showCorrelationPlot2(db='Illum_Retina_BXD_RankInv0410',ProbeSetID='ILMN_2626143',CellID='',db2='Illum_Retina_BXD_RankInv0410',ProbeSetID2='ILMN_3045723',CellID2='',rank='1')) | [**0.296 79**](javascript:showCorrelationPlot2(db='Illum_Retina_BXD_RankInv0410',ProbeSetID='ILMN_2626143',CellID='',db2='Illum_Retina_BXD_RankInv0410',ProbeSetID2='ILMN_2486573',CellID2='',rank='1')) |
|  | [**Trait 10: ILMN_1234930**](javascript:showDatabase2('Illum_Retina_BXD_RankInv0410','ILMN_1234930','');)  Symbol: Vps37d  Vps37d | [**0.693 79**](javascript:showCorrelationPlot2(db='Illum_Retina_BXD_RankInv0410',ProbeSetID='ILMN_1234930',CellID='',db2='Illum_Retina_BXD_RankInv0410',ProbeSetID2='ILMN_2450384',CellID2='',rank='0')) | [**0.616 79**](javascript:showCorrelationPlot2(db='Illum_Retina_BXD_RankInv0410',ProbeSetID='ILMN_1234930',CellID='',db2='Illum_Retina_BXD_RankInv0410',ProbeSetID2='ILMN_3068754',CellID2='',rank='0')) | [**0.576 79**](javascript:showCorrelationPlot2(db='Illum_Retina_BXD_RankInv0410',ProbeSetID='ILMN_1234930',CellID='',db2='Illum_Retina_BXD_RankInv0410',ProbeSetID2='ILMN_2677092',CellID2='',rank='0')) | [**0.721 79**](javascript:showCorrelationPlot2(db='Illum_Retina_BXD_RankInv0410',ProbeSetID='ILMN_1234930',CellID='',db2='Illum_Retina_BXD_RankInv0410',ProbeSetID2='ILMN_2624938',CellID2='',rank='0')) | [**0.601 79**](javascript:showCorrelationPlot2(db='Illum_Retina_BXD_RankInv0410',ProbeSetID='ILMN_1234930',CellID='',db2='Illum_Retina_BXD_RankInv0410',ProbeSetID2='ILMN_2847269',CellID2='',rank='0')) | [**0.618 79**](javascript:showCorrelationPlot2(db='Illum_Retina_BXD_RankInv0410',ProbeSetID='ILMN_1234930',CellID='',db2='Illum_Retina_BXD_RankInv0410',ProbeSetID2='ILMN_2839682',CellID2='',rank='0')) | [**0.460 79**](javascript:showCorrelationPlot2(db='Illum_Retina_BXD_RankInv0410',ProbeSetID='ILMN_1234930',CellID='',db2='Illum_Retina_BXD_RankInv0410',ProbeSetID2='ILMN_3147135',CellID2='',rank='0')) | [**0.566 79**](javascript:showCorrelationPlot2(db='Illum_Retina_BXD_RankInv0410',ProbeSetID='ILMN_1234930',CellID='',db2='Illum_Retina_BXD_RankInv0410',ProbeSetID2='ILMN_1255823',CellID2='',rank='0')) | [**0.680 79**](javascript:showCorrelationPlot2(db='Illum_Retina_BXD_RankInv0410',ProbeSetID='ILMN_1234930',CellID='',db2='Illum_Retina_BXD_RankInv0410',ProbeSetID2='ILMN_2626143',CellID2='',rank='0')) | [***n* 79**](javascript:showDatabase2('Illum_Retina_BXD_RankInv0410','ILMN_1234930','')) | [**0.616 79**](javascript:showCorrelationPlot2(db='Illum_Retina_BXD_RankInv0410',ProbeSetID='ILMN_1234930',CellID='',db2='Illum_Retina_BXD_RankInv0410',ProbeSetID2='ILMN_1249408',CellID2='',rank='1')) | [**0.640 79**](javascript:showCorrelationPlot2(db='Illum_Retina_BXD_RankInv0410',ProbeSetID='ILMN_1234930',CellID='',db2='Illum_Retina_BXD_RankInv0410',ProbeSetID2='ILMN_2971559',CellID2='',rank='1')) | [**0.576 79**](javascript:showCorrelationPlot2(db='Illum_Retina_BXD_RankInv0410',ProbeSetID='ILMN_1234930',CellID='',db2='Illum_Retina_BXD_RankInv0410',ProbeSetID2='ILMN_1258455',CellID2='',rank='1')) | [**0.498 79**](javascript:showCorrelationPlot2(db='Illum_Retina_BXD_RankInv0410',ProbeSetID='ILMN_1234930',CellID='',db2='Illum_Retina_BXD_RankInv0410',ProbeSetID2='ILMN_2691613',CellID2='',rank='1')) | [**0.651 79**](javascript:showCorrelationPlot2(db='Illum_Retina_BXD_RankInv0410',ProbeSetID='ILMN_1234930',CellID='',db2='Illum_Retina_BXD_RankInv0410',ProbeSetID2='ILMN_1253600',CellID2='',rank='1')) | [**0.545 79**](javascript:showCorrelationPlot2(db='Illum_Retina_BXD_RankInv0410',ProbeSetID='ILMN_1234930',CellID='',db2='Illum_Retina_BXD_RankInv0410',ProbeSetID2='ILMN_2991545',CellID2='',rank='1')) | [**0.647 79**](javascript:showCorrelationPlot2(db='Illum_Retina_BXD_RankInv0410',ProbeSetID='ILMN_1234930',CellID='',db2='Illum_Retina_BXD_RankInv0410',ProbeSetID2='ILMN_2623591',CellID2='',rank='1')) | [**0.438 79**](javascript:showCorrelationPlot2(db='Illum_Retina_BXD_RankInv0410',ProbeSetID='ILMN_1234930',CellID='',db2='Illum_Retina_BXD_RankInv0410',ProbeSetID2='ILMN_2603568',CellID2='',rank='1')) | [**0.504 79**](javascript:showCorrelationPlot2(db='Illum_Retina_BXD_RankInv0410',ProbeSetID='ILMN_1234930',CellID='',db2='Illum_Retina_BXD_RankInv0410',ProbeSetID2='ILMN_2992541',CellID2='',rank='1')) | [**0.566 79**](javascript:showCorrelationPlot2(db='Illum_Retina_BXD_RankInv0410',ProbeSetID='ILMN_1234930',CellID='',db2='Illum_Retina_BXD_RankInv0410',ProbeSetID2='ILMN_2792868',CellID2='',rank='1')) | [**0.720 79**](javascript:showCorrelationPlot2(db='Illum_Retina_BXD_RankInv0410',ProbeSetID='ILMN_1234930',CellID='',db2='Illum_Retina_BXD_RankInv0410',ProbeSetID2='ILMN_3129497',CellID2='',rank='1')) | [**0.587 79**](javascript:showCorrelationPlot2(db='Illum_Retina_BXD_RankInv0410',ProbeSetID='ILMN_1234930',CellID='',db2='Illum_Retina_BXD_RankInv0410',ProbeSetID2='ILMN_1224034',CellID2='',rank='1')) | [**0.529 79**](javascript:showCorrelationPlot2(db='Illum_Retina_BXD_RankInv0410',ProbeSetID='ILMN_1234930',CellID='',db2='Illum_Retina_BXD_RankInv0410',ProbeSetID2='ILMN_2639819',CellID2='',rank='1')) | [**0.504 79**](javascript:showCorrelationPlot2(db='Illum_Retina_BXD_RankInv0410',ProbeSetID='ILMN_1234930',CellID='',db2='Illum_Retina_BXD_RankInv0410',ProbeSetID2='ILMN_2994779',CellID2='',rank='1')) | [**0.605 79**](javascript:showCorrelationPlot2(db='Illum_Retina_BXD_RankInv0410',ProbeSetID='ILMN_1234930',CellID='',db2='Illum_Retina_BXD_RankInv0410',ProbeSetID2='ILMN_2625047',CellID2='',rank='1')) | [**0.449 79**](javascript:showCorrelationPlot2(db='Illum_Retina_BXD_RankInv0410',ProbeSetID='ILMN_1234930',CellID='',db2='Illum_Retina_BXD_RankInv0410',ProbeSetID2='ILMN_2808186',CellID2='',rank='1')) | [**0.550 79**](javascript:showCorrelationPlot2(db='Illum_Retina_BXD_RankInv0410',ProbeSetID='ILMN_1234930',CellID='',db2='Illum_Retina_BXD_RankInv0410',ProbeSetID2='ILMN_2689056',CellID2='',rank='1')) | [**0.488 79**](javascript:showCorrelationPlot2(db='Illum_Retina_BXD_RankInv0410',ProbeSetID='ILMN_1234930',CellID='',db2='Illum_Retina_BXD_RankInv0410',ProbeSetID2='ILMN_2648386',CellID2='',rank='1')) | [**0.636 79**](javascript:showCorrelationPlot2(db='Illum_Retina_BXD_RankInv0410',ProbeSetID='ILMN_1234930',CellID='',db2='Illum_Retina_BXD_RankInv0410',ProbeSetID2='ILMN_2869225',CellID2='',rank='1')) | [**0.591 79**](javascript:showCorrelationPlot2(db='Illum_Retina_BXD_RankInv0410',ProbeSetID='ILMN_1234930',CellID='',db2='Illum_Retina_BXD_RankInv0410',ProbeSetID2='ILMN_2728431',CellID2='',rank='1')) | [**0.594 79**](javascript:showCorrelationPlot2(db='Illum_Retina_BXD_RankInv0410',ProbeSetID='ILMN_1234930',CellID='',db2='Illum_Retina_BXD_RankInv0410',ProbeSetID2='ILMN_3094608',CellID2='',rank='1')) | [**0.406 79**](javascript:showCorrelationPlot2(db='Illum_Retina_BXD_RankInv0410',ProbeSetID='ILMN_1234930',CellID='',db2='Illum_Retina_BXD_RankInv0410',ProbeSetID2='ILMN_2896528',CellID2='',rank='1')) | [**0.464 79**](javascript:showCorrelationPlot2(db='Illum_Retina_BXD_RankInv0410',ProbeSetID='ILMN_1234930',CellID='',db2='Illum_Retina_BXD_RankInv0410',ProbeSetID2='ILMN_2701750',CellID2='',rank='1')) | [**0.638 79**](javascript:showCorrelationPlot2(db='Illum_Retina_BXD_RankInv0410',ProbeSetID='ILMN_1234930',CellID='',db2='Illum_Retina_BXD_RankInv0410',ProbeSetID2='ILMN_1239448',CellID2='',rank='1')) | [**-0.341 79**](javascript:showCorrelationPlot2(db='Illum_Retina_BXD_RankInv0410',ProbeSetID='ILMN_1234930',CellID='',db2='Illum_Retina_BXD_RankInv0410',ProbeSetID2='ILMN_2583882',CellID2='',rank='1')) | [**0.523 79**](javascript:showCorrelationPlot2(db='Illum_Retina_BXD_RankInv0410',ProbeSetID='ILMN_1234930',CellID='',db2='Illum_Retina_BXD_RankInv0410',ProbeSetID2='ILMN_2915166',CellID2='',rank='1')) | [**0.568 79**](javascript:showCorrelationPlot2(db='Illum_Retina_BXD_RankInv0410',ProbeSetID='ILMN_1234930',CellID='',db2='Illum_Retina_BXD_RankInv0410',ProbeSetID2='ILMN_2702997',CellID2='',rank='1')) | [**0.587 79**](javascript:showCorrelationPlot2(db='Illum_Retina_BXD_RankInv0410',ProbeSetID='ILMN_1234930',CellID='',db2='Illum_Retina_BXD_RankInv0410',ProbeSetID2='ILMN_2435206',CellID2='',rank='1')) | [**0.461 79**](javascript:showCorrelationPlot2(db='Illum_Retina_BXD_RankInv0410',ProbeSetID='ILMN_1234930',CellID='',db2='Illum_Retina_BXD_RankInv0410',ProbeSetID2='ILMN_2789888',CellID2='',rank='1')) | [**0.528 79**](javascript:showCorrelationPlot2(db='Illum_Retina_BXD_RankInv0410',ProbeSetID='ILMN_1234930',CellID='',db2='Illum_Retina_BXD_RankInv0410',ProbeSetID2='ILMN_1218901',CellID2='',rank='1')) | [**0.477 79**](javascript:showCorrelationPlot2(db='Illum_Retina_BXD_RankInv0410',ProbeSetID='ILMN_1234930',CellID='',db2='Illum_Retina_BXD_RankInv0410',ProbeSetID2='ILMN_1254734',CellID2='',rank='1')) | [**0.540 79**](javascript:showCorrelationPlot2(db='Illum_Retina_BXD_RankInv0410',ProbeSetID='ILMN_1234930',CellID='',db2='Illum_Retina_BXD_RankInv0410',ProbeSetID2='ILMN_3008924',CellID2='',rank='1')) | [**0.513 79**](javascript:showCorrelationPlot2(db='Illum_Retina_BXD_RankInv0410',ProbeSetID='ILMN_1234930',CellID='',db2='Illum_Retina_BXD_RankInv0410',ProbeSetID2='ILMN_2628175',CellID2='',rank='1')) | [**0.560 79**](javascript:showCorrelationPlot2(db='Illum_Retina_BXD_RankInv0410',ProbeSetID='ILMN_1234930',CellID='',db2='Illum_Retina_BXD_RankInv0410',ProbeSetID2='ILMN_2891573',CellID2='',rank='1')) | [**0.568 79**](javascript:showCorrelationPlot2(db='Illum_Retina_BXD_RankInv0410',ProbeSetID='ILMN_1234930',CellID='',db2='Illum_Retina_BXD_RankInv0410',ProbeSetID2='ILMN_1220846',CellID2='',rank='1')) | [**0.508 79**](javascript:showCorrelationPlot2(db='Illum_Retina_BXD_RankInv0410',ProbeSetID='ILMN_1234930',CellID='',db2='Illum_Retina_BXD_RankInv0410',ProbeSetID2='ILMN_1241962',CellID2='',rank='1')) | [**0.486 79**](javascript:showCorrelationPlot2(db='Illum_Retina_BXD_RankInv0410',ProbeSetID='ILMN_1234930',CellID='',db2='Illum_Retina_BXD_RankInv0410',ProbeSetID2='ILMN_3163027',CellID2='',rank='1')) | [**0.345 79**](javascript:showCorrelationPlot2(db='Illum_Retina_BXD_RankInv0410',ProbeSetID='ILMN_1234930',CellID='',db2='Illum_Retina_BXD_RankInv0410',ProbeSetID2='ILMN_2775098',CellID2='',rank='1')) | [**0.638 79**](javascript:showCorrelationPlot2(db='Illum_Retina_BXD_RankInv0410',ProbeSetID='ILMN_1234930',CellID='',db2='Illum_Retina_BXD_RankInv0410',ProbeSetID2='ILMN_2752552',CellID2='',rank='1')) | [**0.614 79**](javascript:showCorrelationPlot2(db='Illum_Retina_BXD_RankInv0410',ProbeSetID='ILMN_1234930',CellID='',db2='Illum_Retina_BXD_RankInv0410',ProbeSetID2='ILMN_2730293',CellID2='',rank='1')) | [**0.630 79**](javascript:showCorrelationPlot2(db='Illum_Retina_BXD_RankInv0410',ProbeSetID='ILMN_1234930',CellID='',db2='Illum_Retina_BXD_RankInv0410',ProbeSetID2='ILMN_2680872',CellID2='',rank='1')) | [**0.177 79**](javascript:showCorrelationPlot2(db='Illum_Retina_BXD_RankInv0410',ProbeSetID='ILMN_1234930',CellID='',db2='Illum_Retina_BXD_RankInv0410',ProbeSetID2='ILMN_2768533',CellID2='',rank='1')) | [**-0.110 79**](javascript:showCorrelationPlot2(db='Illum_Retina_BXD_RankInv0410',ProbeSetID='ILMN_1234930',CellID='',db2='Illum_Retina_BXD_RankInv0410',ProbeSetID2='ILMN_3121255',CellID2='',rank='1')) | [**-0.160 79**](javascript:showCorrelationPlot2(db='Illum_Retina_BXD_RankInv0410',ProbeSetID='ILMN_1234930',CellID='',db2='Illum_Retina_BXD_RankInv0410',ProbeSetID2='ILMN_2484527',CellID2='',rank='1')) | [**0.040 79**](javascript:showCorrelationPlot2(db='Illum_Retina_BXD_RankInv0410',ProbeSetID='ILMN_1234930',CellID='',db2='Illum_Retina_BXD_RankInv0410',ProbeSetID2='ILMN_3045723',CellID2='',rank='1')) | [**0.295 79**](javascript:showCorrelationPlot2(db='Illum_Retina_BXD_RankInv0410',ProbeSetID='ILMN_1234930',CellID='',db2='Illum_Retina_BXD_RankInv0410',ProbeSetID2='ILMN_2486573',CellID2='',rank='1')) |
|  | [**Trait 11: ILMN_1249408**](javascript:showDatabase2('Illum_Retina_BXD_RankInv0410','ILMN_1249408','');)  Symbol: Cox14  Cox14 | [**0.692 79**](javascript:showCorrelationPlot2(db='Illum_Retina_BXD_RankInv0410',ProbeSetID='ILMN_1249408',CellID='',db2='Illum_Retina_BXD_RankInv0410',ProbeSetID2='ILMN_2450384',CellID2='',rank='0')) | [**0.591 79**](javascript:showCorrelationPlot2(db='Illum_Retina_BXD_RankInv0410',ProbeSetID='ILMN_1249408',CellID='',db2='Illum_Retina_BXD_RankInv0410',ProbeSetID2='ILMN_3068754',CellID2='',rank='0')) | [**0.591 79**](javascript:showCorrelationPlot2(db='Illum_Retina_BXD_RankInv0410',ProbeSetID='ILMN_1249408',CellID='',db2='Illum_Retina_BXD_RankInv0410',ProbeSetID2='ILMN_2677092',CellID2='',rank='0')) | [**0.579 79**](javascript:showCorrelationPlot2(db='Illum_Retina_BXD_RankInv0410',ProbeSetID='ILMN_1249408',CellID='',db2='Illum_Retina_BXD_RankInv0410',ProbeSetID2='ILMN_2624938',CellID2='',rank='0')) | [**0.617 79**](javascript:showCorrelationPlot2(db='Illum_Retina_BXD_RankInv0410',ProbeSetID='ILMN_1249408',CellID='',db2='Illum_Retina_BXD_RankInv0410',ProbeSetID2='ILMN_2847269',CellID2='',rank='0')) | [**0.376 79**](javascript:showCorrelationPlot2(db='Illum_Retina_BXD_RankInv0410',ProbeSetID='ILMN_1249408',CellID='',db2='Illum_Retina_BXD_RankInv0410',ProbeSetID2='ILMN_2839682',CellID2='',rank='0')) | [**0.672 79**](javascript:showCorrelationPlot2(db='Illum_Retina_BXD_RankInv0410',ProbeSetID='ILMN_1249408',CellID='',db2='Illum_Retina_BXD_RankInv0410',ProbeSetID2='ILMN_3147135',CellID2='',rank='0')) | [**0.857 79**](javascript:showCorrelationPlot2(db='Illum_Retina_BXD_RankInv0410',ProbeSetID='ILMN_1249408',CellID='',db2='Illum_Retina_BXD_RankInv0410',ProbeSetID2='ILMN_1255823',CellID2='',rank='0')) | [**0.674 79**](javascript:showCorrelationPlot2(db='Illum_Retina_BXD_RankInv0410',ProbeSetID='ILMN_1249408',CellID='',db2='Illum_Retina_BXD_RankInv0410',ProbeSetID2='ILMN_2626143',CellID2='',rank='0')) | [**0.593 79**](javascript:showCorrelationPlot2(db='Illum_Retina_BXD_RankInv0410',ProbeSetID='ILMN_1249408',CellID='',db2='Illum_Retina_BXD_RankInv0410',ProbeSetID2='ILMN_1234930',CellID2='',rank='0')) | [***n* 79**](javascript:showDatabase2('Illum_Retina_BXD_RankInv0410','ILMN_1249408','')) | [**0.428 79**](javascript:showCorrelationPlot2(db='Illum_Retina_BXD_RankInv0410',ProbeSetID='ILMN_1249408',CellID='',db2='Illum_Retina_BXD_RankInv0410',ProbeSetID2='ILMN_2971559',CellID2='',rank='1')) | [**0.475 79**](javascript:showCorrelationPlot2(db='Illum_Retina_BXD_RankInv0410',ProbeSetID='ILMN_1249408',CellID='',db2='Illum_Retina_BXD_RankInv0410',ProbeSetID2='ILMN_1258455',CellID2='',rank='1')) | [**0.737 79**](javascript:showCorrelationPlot2(db='Illum_Retina_BXD_RankInv0410',ProbeSetID='ILMN_1249408',CellID='',db2='Illum_Retina_BXD_RankInv0410',ProbeSetID2='ILMN_2691613',CellID2='',rank='1')) | [**0.544 79**](javascript:showCorrelationPlot2(db='Illum_Retina_BXD_RankInv0410',ProbeSetID='ILMN_1249408',CellID='',db2='Illum_Retina_BXD_RankInv0410',ProbeSetID2='ILMN_1253600',CellID2='',rank='1')) | [**0.583 79**](javascript:showCorrelationPlot2(db='Illum_Retina_BXD_RankInv0410',ProbeSetID='ILMN_1249408',CellID='',db2='Illum_Retina_BXD_RankInv0410',ProbeSetID2='ILMN_2991545',CellID2='',rank='1')) | [**0.563 79**](javascript:showCorrelationPlot2(db='Illum_Retina_BXD_RankInv0410',ProbeSetID='ILMN_1249408',CellID='',db2='Illum_Retina_BXD_RankInv0410',ProbeSetID2='ILMN_2623591',CellID2='',rank='1')) | [**0.636 79**](javascript:showCorrelationPlot2(db='Illum_Retina_BXD_RankInv0410',ProbeSetID='ILMN_1249408',CellID='',db2='Illum_Retina_BXD_RankInv0410',ProbeSetID2='ILMN_2603568',CellID2='',rank='1')) | [**0.696 79**](javascript:showCorrelationPlot2(db='Illum_Retina_BXD_RankInv0410',ProbeSetID='ILMN_1249408',CellID='',db2='Illum_Retina_BXD_RankInv0410',ProbeSetID2='ILMN_2992541',CellID2='',rank='1')) | [**0.675 79**](javascript:showCorrelationPlot2(db='Illum_Retina_BXD_RankInv0410',ProbeSetID='ILMN_1249408',CellID='',db2='Illum_Retina_BXD_RankInv0410',ProbeSetID2='ILMN_2792868',CellID2='',rank='1')) | [**0.533 79**](javascript:showCorrelationPlot2(db='Illum_Retina_BXD_RankInv0410',ProbeSetID='ILMN_1249408',CellID='',db2='Illum_Retina_BXD_RankInv0410',ProbeSetID2='ILMN_3129497',CellID2='',rank='1')) | [**0.464 79**](javascript:showCorrelationPlot2(db='Illum_Retina_BXD_RankInv0410',ProbeSetID='ILMN_1249408',CellID='',db2='Illum_Retina_BXD_RankInv0410',ProbeSetID2='ILMN_1224034',CellID2='',rank='1')) | [**0.596 79**](javascript:showCorrelationPlot2(db='Illum_Retina_BXD_RankInv0410',ProbeSetID='ILMN_1249408',CellID='',db2='Illum_Retina_BXD_RankInv0410',ProbeSetID2='ILMN_2639819',CellID2='',rank='1')) | [**0.590 79**](javascript:showCorrelationPlot2(db='Illum_Retina_BXD_RankInv0410',ProbeSetID='ILMN_1249408',CellID='',db2='Illum_Retina_BXD_RankInv0410',ProbeSetID2='ILMN_2994779',CellID2='',rank='1')) | [**0.560 79**](javascript:showCorrelationPlot2(db='Illum_Retina_BXD_RankInv0410',ProbeSetID='ILMN_1249408',CellID='',db2='Illum_Retina_BXD_RankInv0410',ProbeSetID2='ILMN_2625047',CellID2='',rank='1')) | [**0.348 79**](javascript:showCorrelationPlot2(db='Illum_Retina_BXD_RankInv0410',ProbeSetID='ILMN_1249408',CellID='',db2='Illum_Retina_BXD_RankInv0410',ProbeSetID2='ILMN_2808186',CellID2='',rank='1')) | [**0.504 79**](javascript:showCorrelationPlot2(db='Illum_Retina_BXD_RankInv0410',ProbeSetID='ILMN_1249408',CellID='',db2='Illum_Retina_BXD_RankInv0410',ProbeSetID2='ILMN_2689056',CellID2='',rank='1')) | [**0.753 79**](javascript:showCorrelationPlot2(db='Illum_Retina_BXD_RankInv0410',ProbeSetID='ILMN_1249408',CellID='',db2='Illum_Retina_BXD_RankInv0410',ProbeSetID2='ILMN_2648386',CellID2='',rank='1')) | [**0.692 79**](javascript:showCorrelationPlot2(db='Illum_Retina_BXD_RankInv0410',ProbeSetID='ILMN_1249408',CellID='',db2='Illum_Retina_BXD_RankInv0410',ProbeSetID2='ILMN_2869225',CellID2='',rank='1')) | [**0.674 79**](javascript:showCorrelationPlot2(db='Illum_Retina_BXD_RankInv0410',ProbeSetID='ILMN_1249408',CellID='',db2='Illum_Retina_BXD_RankInv0410',ProbeSetID2='ILMN_2728431',CellID2='',rank='1')) | [**0.429 79**](javascript:showCorrelationPlot2(db='Illum_Retina_BXD_RankInv0410',ProbeSetID='ILMN_1249408',CellID='',db2='Illum_Retina_BXD_RankInv0410',ProbeSetID2='ILMN_3094608',CellID2='',rank='1')) | [**0.651 79**](javascript:showCorrelationPlot2(db='Illum_Retina_BXD_RankInv0410',ProbeSetID='ILMN_1249408',CellID='',db2='Illum_Retina_BXD_RankInv0410',ProbeSetID2='ILMN_2896528',CellID2='',rank='1')) | [**0.455 79**](javascript:showCorrelationPlot2(db='Illum_Retina_BXD_RankInv0410',ProbeSetID='ILMN_1249408',CellID='',db2='Illum_Retina_BXD_RankInv0410',ProbeSetID2='ILMN_2701750',CellID2='',rank='1')) | [**0.609 79**](javascript:showCorrelationPlot2(db='Illum_Retina_BXD_RankInv0410',ProbeSetID='ILMN_1249408',CellID='',db2='Illum_Retina_BXD_RankInv0410',ProbeSetID2='ILMN_1239448',CellID2='',rank='1')) | [**-0.546 79**](javascript:showCorrelationPlot2(db='Illum_Retina_BXD_RankInv0410',ProbeSetID='ILMN_1249408',CellID='',db2='Illum_Retina_BXD_RankInv0410',ProbeSetID2='ILMN_2583882',CellID2='',rank='1')) | [**0.486 79**](javascript:showCorrelationPlot2(db='Illum_Retina_BXD_RankInv0410',ProbeSetID='ILMN_1249408',CellID='',db2='Illum_Retina_BXD_RankInv0410',ProbeSetID2='ILMN_2915166',CellID2='',rank='1')) | [**0.449 79**](javascript:showCorrelationPlot2(db='Illum_Retina_BXD_RankInv0410',ProbeSetID='ILMN_1249408',CellID='',db2='Illum_Retina_BXD_RankInv0410',ProbeSetID2='ILMN_2702997',CellID2='',rank='1')) | [**0.449 79**](javascript:showCorrelationPlot2(db='Illum_Retina_BXD_RankInv0410',ProbeSetID='ILMN_1249408',CellID='',db2='Illum_Retina_BXD_RankInv0410',ProbeSetID2='ILMN_2435206',CellID2='',rank='1')) | [**0.498 79**](javascript:showCorrelationPlot2(db='Illum_Retina_BXD_RankInv0410',ProbeSetID='ILMN_1249408',CellID='',db2='Illum_Retina_BXD_RankInv0410',ProbeSetID2='ILMN_2789888',CellID2='',rank='1')) | [**0.431 79**](javascript:showCorrelationPlot2(db='Illum_Retina_BXD_RankInv0410',ProbeSetID='ILMN_1249408',CellID='',db2='Illum_Retina_BXD_RankInv0410',ProbeSetID2='ILMN_1218901',CellID2='',rank='1')) | [**0.738 79**](javascript:showCorrelationPlot2(db='Illum_Retina_BXD_RankInv0410',ProbeSetID='ILMN_1249408',CellID='',db2='Illum_Retina_BXD_RankInv0410',ProbeSetID2='ILMN_1254734',CellID2='',rank='1')) | [**0.521 79**](javascript:showCorrelationPlot2(db='Illum_Retina_BXD_RankInv0410',ProbeSetID='ILMN_1249408',CellID='',db2='Illum_Retina_BXD_RankInv0410',ProbeSetID2='ILMN_3008924',CellID2='',rank='1')) | [**0.550 79**](javascript:showCorrelationPlot2(db='Illum_Retina_BXD_RankInv0410',ProbeSetID='ILMN_1249408',CellID='',db2='Illum_Retina_BXD_RankInv0410',ProbeSetID2='ILMN_2628175',CellID2='',rank='1')) | [**0.552 79**](javascript:showCorrelationPlot2(db='Illum_Retina_BXD_RankInv0410',ProbeSetID='ILMN_1249408',CellID='',db2='Illum_Retina_BXD_RankInv0410',ProbeSetID2='ILMN_2891573',CellID2='',rank='1')) | [**0.630 79**](javascript:showCorrelationPlot2(db='Illum_Retina_BXD_RankInv0410',ProbeSetID='ILMN_1249408',CellID='',db2='Illum_Retina_BXD_RankInv0410',ProbeSetID2='ILMN_1220846',CellID2='',rank='1')) | [**0.721 79**](javascript:showCorrelationPlot2(db='Illum_Retina_BXD_RankInv0410',ProbeSetID='ILMN_1249408',CellID='',db2='Illum_Retina_BXD_RankInv0410',ProbeSetID2='ILMN_1241962',CellID2='',rank='1')) | [**0.459 79**](javascript:showCorrelationPlot2(db='Illum_Retina_BXD_RankInv0410',ProbeSetID='ILMN_1249408',CellID='',db2='Illum_Retina_BXD_RankInv0410',ProbeSetID2='ILMN_3163027',CellID2='',rank='1')) | [**0.568 79**](javascript:showCorrelationPlot2(db='Illum_Retina_BXD_RankInv0410',ProbeSetID='ILMN_1249408',CellID='',db2='Illum_Retina_BXD_RankInv0410',ProbeSetID2='ILMN_2775098',CellID2='',rank='1')) | [**0.622 79**](javascript:showCorrelationPlot2(db='Illum_Retina_BXD_RankInv0410',ProbeSetID='ILMN_1249408',CellID='',db2='Illum_Retina_BXD_RankInv0410',ProbeSetID2='ILMN_2752552',CellID2='',rank='1')) | [**0.454 79**](javascript:showCorrelationPlot2(db='Illum_Retina_BXD_RankInv0410',ProbeSetID='ILMN_1249408',CellID='',db2='Illum_Retina_BXD_RankInv0410',ProbeSetID2='ILMN_2730293',CellID2='',rank='1')) | [**0.399 79**](javascript:showCorrelationPlot2(db='Illum_Retina_BXD_RankInv0410',ProbeSetID='ILMN_1249408',CellID='',db2='Illum_Retina_BXD_RankInv0410',ProbeSetID2='ILMN_2680872',CellID2='',rank='1')) | [**0.309 79**](javascript:showCorrelationPlot2(db='Illum_Retina_BXD_RankInv0410',ProbeSetID='ILMN_1249408',CellID='',db2='Illum_Retina_BXD_RankInv0410',ProbeSetID2='ILMN_2768533',CellID2='',rank='1')) | [**-0.092 79**](javascript:showCorrelationPlot2(db='Illum_Retina_BXD_RankInv0410',ProbeSetID='ILMN_1249408',CellID='',db2='Illum_Retina_BXD_RankInv0410',ProbeSetID2='ILMN_3121255',CellID2='',rank='1')) | [**-0.338 79**](javascript:showCorrelationPlot2(db='Illum_Retina_BXD_RankInv0410',ProbeSetID='ILMN_1249408',CellID='',db2='Illum_Retina_BXD_RankInv0410',ProbeSetID2='ILMN_2484527',CellID2='',rank='1')) | [**0.260 79**](javascript:showCorrelationPlot2(db='Illum_Retina_BXD_RankInv0410',ProbeSetID='ILMN_1249408',CellID='',db2='Illum_Retina_BXD_RankInv0410',ProbeSetID2='ILMN_3045723',CellID2='',rank='1')) | [**0.206 79**](javascript:showCorrelationPlot2(db='Illum_Retina_BXD_RankInv0410',ProbeSetID='ILMN_1249408',CellID='',db2='Illum_Retina_BXD_RankInv0410',ProbeSetID2='ILMN_2486573',CellID2='',rank='1')) |
|  | [**ILMN_2971559**](javascript:showDatabase2('Illum_Retina_BXD_RankInv0410','ILMN_2971559','');)  Symbol: Eef1a2  Eef1a2 | [**0.677 79**](javascript:showCorrelationPlot2(db='Illum_Retina_BXD_RankInv0410',ProbeSetID='ILMN_2971559',CellID='',db2='Illum_Retina_BXD_RankInv0410',ProbeSetID2='ILMN_2450384',CellID2='',rank='0')) | [**0.516 79**](javascript:showCorrelationPlot2(db='Illum_Retina_BXD_RankInv0410',ProbeSetID='ILMN_2971559',CellID='',db2='Illum_Retina_BXD_RankInv0410',ProbeSetID2='ILMN_3068754',CellID2='',rank='0')) | [**0.489 79**](javascript:showCorrelationPlot2(db='Illum_Retina_BXD_RankInv0410',ProbeSetID='ILMN_2971559',CellID='',db2='Illum_Retina_BXD_RankInv0410',ProbeSetID2='ILMN_2677092',CellID2='',rank='0')) | [**0.744 79**](javascript:showCorrelationPlot2(db='Illum_Retina_BXD_RankInv0410',ProbeSetID='ILMN_2971559',CellID='',db2='Illum_Retina_BXD_RankInv0410',ProbeSetID2='ILMN_2624938',CellID2='',rank='0')) | [**0.684 79**](javascript:showCorrelationPlot2(db='Illum_Retina_BXD_RankInv0410',ProbeSetID='ILMN_2971559',CellID='',db2='Illum_Retina_BXD_RankInv0410',ProbeSetID2='ILMN_2847269',CellID2='',rank='0')) | [**0.643 79**](javascript:showCorrelationPlot2(db='Illum_Retina_BXD_RankInv0410',ProbeSetID='ILMN_2971559',CellID='',db2='Illum_Retina_BXD_RankInv0410',ProbeSetID2='ILMN_2839682',CellID2='',rank='0')) | [**0.394 79**](javascript:showCorrelationPlot2(db='Illum_Retina_BXD_RankInv0410',ProbeSetID='ILMN_2971559',CellID='',db2='Illum_Retina_BXD_RankInv0410',ProbeSetID2='ILMN_3147135',CellID2='',rank='0')) | [**0.403 79**](javascript:showCorrelationPlot2(db='Illum_Retina_BXD_RankInv0410',ProbeSetID='ILMN_2971559',CellID='',db2='Illum_Retina_BXD_RankInv0410',ProbeSetID2='ILMN_1255823',CellID2='',rank='0')) | [**0.568 79**](javascript:showCorrelationPlot2(db='Illum_Retina_BXD_RankInv0410',ProbeSetID='ILMN_2971559',CellID='',db2='Illum_Retina_BXD_RankInv0410',ProbeSetID2='ILMN_2626143',CellID2='',rank='0')) | [**0.729 79**](javascript:showCorrelationPlot2(db='Illum_Retina_BXD_RankInv0410',ProbeSetID='ILMN_2971559',CellID='',db2='Illum_Retina_BXD_RankInv0410',ProbeSetID2='ILMN_1234930',CellID2='',rank='0')) | [**0.341 79**](javascript:showCorrelationPlot2(db='Illum_Retina_BXD_RankInv0410',ProbeSetID='ILMN_2971559',CellID='',db2='Illum_Retina_BXD_RankInv0410',ProbeSetID2='ILMN_1249408',CellID2='',rank='0')) | [***n* 79**](javascript:showDatabase2('Illum_Retina_BXD_RankInv0410','ILMN_2971559','')) | [**0.602 79**](javascript:showCorrelationPlot2(db='Illum_Retina_BXD_RankInv0410',ProbeSetID='ILMN_2971559',CellID='',db2='Illum_Retina_BXD_RankInv0410',ProbeSetID2='ILMN_1258455',CellID2='',rank='1')) | [**0.521 79**](javascript:showCorrelationPlot2(db='Illum_Retina_BXD_RankInv0410',ProbeSetID='ILMN_2971559',CellID='',db2='Illum_Retina_BXD_RankInv0410',ProbeSetID2='ILMN_2691613',CellID2='',rank='1')) | [**0.543 79**](javascript:showCorrelationPlot2(db='Illum_Retina_BXD_RankInv0410',ProbeSetID='ILMN_2971559',CellID='',db2='Illum_Retina_BXD_RankInv0410',ProbeSetID2='ILMN_1253600',CellID2='',rank='1')) | [**0.456 79**](javascript:showCorrelationPlot2(db='Illum_Retina_BXD_RankInv0410',ProbeSetID='ILMN_2971559',CellID='',db2='Illum_Retina_BXD_RankInv0410',ProbeSetID2='ILMN_2991545',CellID2='',rank='1')) | [**0.601 79**](javascript:showCorrelationPlot2(db='Illum_Retina_BXD_RankInv0410',ProbeSetID='ILMN_2971559',CellID='',db2='Illum_Retina_BXD_RankInv0410',ProbeSetID2='ILMN_2623591',CellID2='',rank='1')) | [**0.273 79**](javascript:showCorrelationPlot2(db='Illum_Retina_BXD_RankInv0410',ProbeSetID='ILMN_2971559',CellID='',db2='Illum_Retina_BXD_RankInv0410',ProbeSetID2='ILMN_2603568',CellID2='',rank='1')) | [**0.552 79**](javascript:showCorrelationPlot2(db='Illum_Retina_BXD_RankInv0410',ProbeSetID='ILMN_2971559',CellID='',db2='Illum_Retina_BXD_RankInv0410',ProbeSetID2='ILMN_2992541',CellID2='',rank='1')) | [**0.335 79**](javascript:showCorrelationPlot2(db='Illum_Retina_BXD_RankInv0410',ProbeSetID='ILMN_2971559',CellID='',db2='Illum_Retina_BXD_RankInv0410',ProbeSetID2='ILMN_2792868',CellID2='',rank='1')) | [**0.699 79**](javascript:showCorrelationPlot2(db='Illum_Retina_BXD_RankInv0410',ProbeSetID='ILMN_2971559',CellID='',db2='Illum_Retina_BXD_RankInv0410',ProbeSetID2='ILMN_3129497',CellID2='',rank='1')) | [**0.595 79**](javascript:showCorrelationPlot2(db='Illum_Retina_BXD_RankInv0410',ProbeSetID='ILMN_2971559',CellID='',db2='Illum_Retina_BXD_RankInv0410',ProbeSetID2='ILMN_1224034',CellID2='',rank='1')) | [**0.456 79**](javascript:showCorrelationPlot2(db='Illum_Retina_BXD_RankInv0410',ProbeSetID='ILMN_2971559',CellID='',db2='Illum_Retina_BXD_RankInv0410',ProbeSetID2='ILMN_2639819',CellID2='',rank='1')) | [**0.569 79**](javascript:showCorrelationPlot2(db='Illum_Retina_BXD_RankInv0410',ProbeSetID='ILMN_2971559',CellID='',db2='Illum_Retina_BXD_RankInv0410',ProbeSetID2='ILMN_2994779',CellID2='',rank='1')) | [**0.554 79**](javascript:showCorrelationPlot2(db='Illum_Retina_BXD_RankInv0410',ProbeSetID='ILMN_2971559',CellID='',db2='Illum_Retina_BXD_RankInv0410',ProbeSetID2='ILMN_2625047',CellID2='',rank='1')) | [**0.528 79**](javascript:showCorrelationPlot2(db='Illum_Retina_BXD_RankInv0410',ProbeSetID='ILMN_2971559',CellID='',db2='Illum_Retina_BXD_RankInv0410',ProbeSetID2='ILMN_2808186',CellID2='',rank='1')) | [**0.587 79**](javascript:showCorrelationPlot2(db='Illum_Retina_BXD_RankInv0410',ProbeSetID='ILMN_2971559',CellID='',db2='Illum_Retina_BXD_RankInv0410',ProbeSetID2='ILMN_2689056',CellID2='',rank='1')) | [**0.359 79**](javascript:showCorrelationPlot2(db='Illum_Retina_BXD_RankInv0410',ProbeSetID='ILMN_2971559',CellID='',db2='Illum_Retina_BXD_RankInv0410',ProbeSetID2='ILMN_2648386',CellID2='',rank='1')) | [**0.522 79**](javascript:showCorrelationPlot2(db='Illum_Retina_BXD_RankInv0410',ProbeSetID='ILMN_2971559',CellID='',db2='Illum_Retina_BXD_RankInv0410',ProbeSetID2='ILMN_2869225',CellID2='',rank='1')) | [**0.433 79**](javascript:showCorrelationPlot2(db='Illum_Retina_BXD_RankInv0410',ProbeSetID='ILMN_2971559',CellID='',db2='Illum_Retina_BXD_RankInv0410',ProbeSetID2='ILMN_2728431',CellID2='',rank='1')) | [**0.407 79**](javascript:showCorrelationPlot2(db='Illum_Retina_BXD_RankInv0410',ProbeSetID='ILMN_2971559',CellID='',db2='Illum_Retina_BXD_RankInv0410',ProbeSetID2='ILMN_3094608',CellID2='',rank='1')) | [**0.460 79**](javascript:showCorrelationPlot2(db='Illum_Retina_BXD_RankInv0410',ProbeSetID='ILMN_2971559',CellID='',db2='Illum_Retina_BXD_RankInv0410',ProbeSetID2='ILMN_2896528',CellID2='',rank='1')) | [**0.407 79**](javascript:showCorrelationPlot2(db='Illum_Retina_BXD_RankInv0410',ProbeSetID='ILMN_2971559',CellID='',db2='Illum_Retina_BXD_RankInv0410',ProbeSetID2='ILMN_2701750',CellID2='',rank='1')) | [**0.371 79**](javascript:showCorrelationPlot2(db='Illum_Retina_BXD_RankInv0410',ProbeSetID='ILMN_2971559',CellID='',db2='Illum_Retina_BXD_RankInv0410',ProbeSetID2='ILMN_1239448',CellID2='',rank='1')) | [**-0.315 79**](javascript:showCorrelationPlot2(db='Illum_Retina_BXD_RankInv0410',ProbeSetID='ILMN_2971559',CellID='',db2='Illum_Retina_BXD_RankInv0410',ProbeSetID2='ILMN_2583882',CellID2='',rank='1')) | [**0.520 79**](javascript:showCorrelationPlot2(db='Illum_Retina_BXD_RankInv0410',ProbeSetID='ILMN_2971559',CellID='',db2='Illum_Retina_BXD_RankInv0410',ProbeSetID2='ILMN_2915166',CellID2='',rank='1')) | [**0.579 79**](javascript:showCorrelationPlot2(db='Illum_Retina_BXD_RankInv0410',ProbeSetID='ILMN_2971559',CellID='',db2='Illum_Retina_BXD_RankInv0410',ProbeSetID2='ILMN_2702997',CellID2='',rank='1')) | [**0.567 79**](javascript:showCorrelationPlot2(db='Illum_Retina_BXD_RankInv0410',ProbeSetID='ILMN_2971559',CellID='',db2='Illum_Retina_BXD_RankInv0410',ProbeSetID2='ILMN_2435206',CellID2='',rank='1')) | [**0.459 79**](javascript:showCorrelationPlot2(db='Illum_Retina_BXD_RankInv0410',ProbeSetID='ILMN_2971559',CellID='',db2='Illum_Retina_BXD_RankInv0410',ProbeSetID2='ILMN_2789888',CellID2='',rank='1')) | [**0.584 79**](javascript:showCorrelationPlot2(db='Illum_Retina_BXD_RankInv0410',ProbeSetID='ILMN_2971559',CellID='',db2='Illum_Retina_BXD_RankInv0410',ProbeSetID2='ILMN_1218901',CellID2='',rank='1')) | [**0.378 79**](javascript:showCorrelationPlot2(db='Illum_Retina_BXD_RankInv0410',ProbeSetID='ILMN_2971559',CellID='',db2='Illum_Retina_BXD_RankInv0410',ProbeSetID2='ILMN_1254734',CellID2='',rank='1')) | [**0.415 79**](javascript:showCorrelationPlot2(db='Illum_Retina_BXD_RankInv0410',ProbeSetID='ILMN_2971559',CellID='',db2='Illum_Retina_BXD_RankInv0410',ProbeSetID2='ILMN_3008924',CellID2='',rank='1')) | [**0.371 79**](javascript:showCorrelationPlot2(db='Illum_Retina_BXD_RankInv0410',ProbeSetID='ILMN_2971559',CellID='',db2='Illum_Retina_BXD_RankInv0410',ProbeSetID2='ILMN_2628175',CellID2='',rank='1')) | [**0.562 79**](javascript:showCorrelationPlot2(db='Illum_Retina_BXD_RankInv0410',ProbeSetID='ILMN_2971559',CellID='',db2='Illum_Retina_BXD_RankInv0410',ProbeSetID2='ILMN_2891573',CellID2='',rank='1')) | [**0.453 79**](javascript:showCorrelationPlot2(db='Illum_Retina_BXD_RankInv0410',ProbeSetID='ILMN_2971559',CellID='',db2='Illum_Retina_BXD_RankInv0410',ProbeSetID2='ILMN_1220846',CellID2='',rank='1')) | [**0.429 79**](javascript:showCorrelationPlot2(db='Illum_Retina_BXD_RankInv0410',ProbeSetID='ILMN_2971559',CellID='',db2='Illum_Retina_BXD_RankInv0410',ProbeSetID2='ILMN_1241962',CellID2='',rank='1')) | [**0.704 79**](javascript:showCorrelationPlot2(db='Illum_Retina_BXD_RankInv0410',ProbeSetID='ILMN_2971559',CellID='',db2='Illum_Retina_BXD_RankInv0410',ProbeSetID2='ILMN_3163027',CellID2='',rank='1')) | [**0.205 79**](javascript:showCorrelationPlot2(db='Illum_Retina_BXD_RankInv0410',ProbeSetID='ILMN_2971559',CellID='',db2='Illum_Retina_BXD_RankInv0410',ProbeSetID2='ILMN_2775098',CellID2='',rank='1')) | [**0.551 79**](javascript:showCorrelationPlot2(db='Illum_Retina_BXD_RankInv0410',ProbeSetID='ILMN_2971559',CellID='',db2='Illum_Retina_BXD_RankInv0410',ProbeSetID2='ILMN_2752552',CellID2='',rank='1')) | [**0.629 79**](javascript:showCorrelationPlot2(db='Illum_Retina_BXD_RankInv0410',ProbeSetID='ILMN_2971559',CellID='',db2='Illum_Retina_BXD_RankInv0410',ProbeSetID2='ILMN_2730293',CellID2='',rank='1')) | [**0.607 79**](javascript:showCorrelationPlot2(db='Illum_Retina_BXD_RankInv0410',ProbeSetID='ILMN_2971559',CellID='',db2='Illum_Retina_BXD_RankInv0410',ProbeSetID2='ILMN_2680872',CellID2='',rank='1')) | [**0.042 79**](javascript:showCorrelationPlot2(db='Illum_Retina_BXD_RankInv0410',ProbeSetID='ILMN_2971559',CellID='',db2='Illum_Retina_BXD_RankInv0410',ProbeSetID2='ILMN_2768533',CellID2='',rank='1')) | [**-0.160 79**](javascript:showCorrelationPlot2(db='Illum_Retina_BXD_RankInv0410',ProbeSetID='ILMN_2971559',CellID='',db2='Illum_Retina_BXD_RankInv0410',ProbeSetID2='ILMN_3121255',CellID2='',rank='1')) | [**-0.111 79**](javascript:showCorrelationPlot2(db='Illum_Retina_BXD_RankInv0410',ProbeSetID='ILMN_2971559',CellID='',db2='Illum_Retina_BXD_RankInv0410',ProbeSetID2='ILMN_2484527',CellID2='',rank='1')) | [**0.192 79**](javascript:showCorrelationPlot2(db='Illum_Retina_BXD_RankInv0410',ProbeSetID='ILMN_2971559',CellID='',db2='Illum_Retina_BXD_RankInv0410',ProbeSetID2='ILMN_3045723',CellID2='',rank='1')) | [**0.370 79**](javascript:showCorrelationPlot2(db='Illum_Retina_BXD_RankInv0410',ProbeSetID='ILMN_2971559',CellID='',db2='Illum_Retina_BXD_RankInv0410',ProbeSetID2='ILMN_2486573',CellID2='',rank='1')) |
|  | [**Trait 13: ILMN_1258455**](javascript:showDatabase2('Illum_Retina_BXD_RankInv0410','ILMN_1258455','');)  Symbol: C030014I23Rik  C030014I23Rik | [**0.676 79**](javascript:showCorrelationPlot2(db='Illum_Retina_BXD_RankInv0410',ProbeSetID='ILMN_1258455',CellID='',db2='Illum_Retina_BXD_RankInv0410',ProbeSetID2='ILMN_2450384',CellID2='',rank='0')) | [**0.558 79**](javascript:showCorrelationPlot2(db='Illum_Retina_BXD_RankInv0410',ProbeSetID='ILMN_1258455',CellID='',db2='Illum_Retina_BXD_RankInv0410',ProbeSetID2='ILMN_3068754',CellID2='',rank='0')) | [**0.616 79**](javascript:showCorrelationPlot2(db='Illum_Retina_BXD_RankInv0410',ProbeSetID='ILMN_1258455',CellID='',db2='Illum_Retina_BXD_RankInv0410',ProbeSetID2='ILMN_2677092',CellID2='',rank='0')) | [**0.539 79**](javascript:showCorrelationPlot2(db='Illum_Retina_BXD_RankInv0410',ProbeSetID='ILMN_1258455',CellID='',db2='Illum_Retina_BXD_RankInv0410',ProbeSetID2='ILMN_2624938',CellID2='',rank='0')) | [**0.408 79**](javascript:showCorrelationPlot2(db='Illum_Retina_BXD_RankInv0410',ProbeSetID='ILMN_1258455',CellID='',db2='Illum_Retina_BXD_RankInv0410',ProbeSetID2='ILMN_2847269',CellID2='',rank='0')) | [**0.430 79**](javascript:showCorrelationPlot2(db='Illum_Retina_BXD_RankInv0410',ProbeSetID='ILMN_1258455',CellID='',db2='Illum_Retina_BXD_RankInv0410',ProbeSetID2='ILMN_2839682',CellID2='',rank='0')) | [**0.257 79**](javascript:showCorrelationPlot2(db='Illum_Retina_BXD_RankInv0410',ProbeSetID='ILMN_1258455',CellID='',db2='Illum_Retina_BXD_RankInv0410',ProbeSetID2='ILMN_3147135',CellID2='',rank='0')) | [**0.364 79**](javascript:showCorrelationPlot2(db='Illum_Retina_BXD_RankInv0410',ProbeSetID='ILMN_1258455',CellID='',db2='Illum_Retina_BXD_RankInv0410',ProbeSetID2='ILMN_1255823',CellID2='',rank='0')) | [**0.566 79**](javascript:showCorrelationPlot2(db='Illum_Retina_BXD_RankInv0410',ProbeSetID='ILMN_1258455',CellID='',db2='Illum_Retina_BXD_RankInv0410',ProbeSetID2='ILMN_2626143',CellID2='',rank='0')) | [**0.527 79**](javascript:showCorrelationPlot2(db='Illum_Retina_BXD_RankInv0410',ProbeSetID='ILMN_1258455',CellID='',db2='Illum_Retina_BXD_RankInv0410',ProbeSetID2='ILMN_1234930',CellID2='',rank='0')) | [**0.365 79**](javascript:showCorrelationPlot2(db='Illum_Retina_BXD_RankInv0410',ProbeSetID='ILMN_1258455',CellID='',db2='Illum_Retina_BXD_RankInv0410',ProbeSetID2='ILMN_1249408',CellID2='',rank='0')) | [**0.589 79**](javascript:showCorrelationPlot2(db='Illum_Retina_BXD_RankInv0410',ProbeSetID='ILMN_1258455',CellID='',db2='Illum_Retina_BXD_RankInv0410',ProbeSetID2='ILMN_2971559',CellID2='',rank='0')) | [***n* 79**](javascript:showDatabase2('Illum_Retina_BXD_RankInv0410','ILMN_1258455','')) | [**0.321 79**](javascript:showCorrelationPlot2(db='Illum_Retina_BXD_RankInv0410',ProbeSetID='ILMN_1258455',CellID='',db2='Illum_Retina_BXD_RankInv0410',ProbeSetID2='ILMN_2691613',CellID2='',rank='1')) | [**0.585 79**](javascript:showCorrelationPlot2(db='Illum_Retina_BXD_RankInv0410',ProbeSetID='ILMN_1258455',CellID='',db2='Illum_Retina_BXD_RankInv0410',ProbeSetID2='ILMN_1253600',CellID2='',rank='1')) | [**0.453 79**](javascript:showCorrelationPlot2(db='Illum_Retina_BXD_RankInv0410',ProbeSetID='ILMN_1258455',CellID='',db2='Illum_Retina_BXD_RankInv0410',ProbeSetID2='ILMN_2991545',CellID2='',rank='1')) | [**0.529 79**](javascript:showCorrelationPlot2(db='Illum_Retina_BXD_RankInv0410',ProbeSetID='ILMN_1258455',CellID='',db2='Illum_Retina_BXD_RankInv0410',ProbeSetID2='ILMN_2623591',CellID2='',rank='1')) | [**0.505 79**](javascript:showCorrelationPlot2(db='Illum_Retina_BXD_RankInv0410',ProbeSetID='ILMN_1258455',CellID='',db2='Illum_Retina_BXD_RankInv0410',ProbeSetID2='ILMN_2603568',CellID2='',rank='1')) | [**0.318 79**](javascript:showCorrelationPlot2(db='Illum_Retina_BXD_RankInv0410',ProbeSetID='ILMN_1258455',CellID='',db2='Illum_Retina_BXD_RankInv0410',ProbeSetID2='ILMN_2992541',CellID2='',rank='1')) | [**0.539 79**](javascript:showCorrelationPlot2(db='Illum_Retina_BXD_RankInv0410',ProbeSetID='ILMN_1258455',CellID='',db2='Illum_Retina_BXD_RankInv0410',ProbeSetID2='ILMN_2792868',CellID2='',rank='1')) | [**0.572 79**](javascript:showCorrelationPlot2(db='Illum_Retina_BXD_RankInv0410',ProbeSetID='ILMN_1258455',CellID='',db2='Illum_Retina_BXD_RankInv0410',ProbeSetID2='ILMN_3129497',CellID2='',rank='1')) | [**0.659 79**](javascript:showCorrelationPlot2(db='Illum_Retina_BXD_RankInv0410',ProbeSetID='ILMN_1258455',CellID='',db2='Illum_Retina_BXD_RankInv0410',ProbeSetID2='ILMN_1224034',CellID2='',rank='1')) | [**0.550 79**](javascript:showCorrelationPlot2(db='Illum_Retina_BXD_RankInv0410',ProbeSetID='ILMN_1258455',CellID='',db2='Illum_Retina_BXD_RankInv0410',ProbeSetID2='ILMN_2639819',CellID2='',rank='1')) | [**0.350 79**](javascript:showCorrelationPlot2(db='Illum_Retina_BXD_RankInv0410',ProbeSetID='ILMN_1258455',CellID='',db2='Illum_Retina_BXD_RankInv0410',ProbeSetID2='ILMN_2994779',CellID2='',rank='1')) | [**0.497 79**](javascript:showCorrelationPlot2(db='Illum_Retina_BXD_RankInv0410',ProbeSetID='ILMN_1258455',CellID='',db2='Illum_Retina_BXD_RankInv0410',ProbeSetID2='ILMN_2625047',CellID2='',rank='1')) | [**0.682 79**](javascript:showCorrelationPlot2(db='Illum_Retina_BXD_RankInv0410',ProbeSetID='ILMN_1258455',CellID='',db2='Illum_Retina_BXD_RankInv0410',ProbeSetID2='ILMN_2808186',CellID2='',rank='1')) | [**0.586 79**](javascript:showCorrelationPlot2(db='Illum_Retina_BXD_RankInv0410',ProbeSetID='ILMN_1258455',CellID='',db2='Illum_Retina_BXD_RankInv0410',ProbeSetID2='ILMN_2689056',CellID2='',rank='1')) | [**0.453 79**](javascript:showCorrelationPlot2(db='Illum_Retina_BXD_RankInv0410',ProbeSetID='ILMN_1258455',CellID='',db2='Illum_Retina_BXD_RankInv0410',ProbeSetID2='ILMN_2648386',CellID2='',rank='1')) | [**0.405 79**](javascript:showCorrelationPlot2(db='Illum_Retina_BXD_RankInv0410',ProbeSetID='ILMN_1258455',CellID='',db2='Illum_Retina_BXD_RankInv0410',ProbeSetID2='ILMN_2869225',CellID2='',rank='1')) | [**0.457 79**](javascript:showCorrelationPlot2(db='Illum_Retina_BXD_RankInv0410',ProbeSetID='ILMN_1258455',CellID='',db2='Illum_Retina_BXD_RankInv0410',ProbeSetID2='ILMN_2728431',CellID2='',rank='1')) | [**0.496 79**](javascript:showCorrelationPlot2(db='Illum_Retina_BXD_RankInv0410',ProbeSetID='ILMN_1258455',CellID='',db2='Illum_Retina_BXD_RankInv0410',ProbeSetID2='ILMN_3094608',CellID2='',rank='1')) | [**0.418 79**](javascript:showCorrelationPlot2(db='Illum_Retina_BXD_RankInv0410',ProbeSetID='ILMN_1258455',CellID='',db2='Illum_Retina_BXD_RankInv0410',ProbeSetID2='ILMN_2896528',CellID2='',rank='1')) | [**0.727 79**](javascript:showCorrelationPlot2(db='Illum_Retina_BXD_RankInv0410',ProbeSetID='ILMN_1258455',CellID='',db2='Illum_Retina_BXD_RankInv0410',ProbeSetID2='ILMN_2701750',CellID2='',rank='1')) | [**0.389 79**](javascript:showCorrelationPlot2(db='Illum_Retina_BXD_RankInv0410',ProbeSetID='ILMN_1258455',CellID='',db2='Illum_Retina_BXD_RankInv0410',ProbeSetID2='ILMN_1239448',CellID2='',rank='1')) | [**-0.403 79**](javascript:showCorrelationPlot2(db='Illum_Retina_BXD_RankInv0410',ProbeSetID='ILMN_1258455',CellID='',db2='Illum_Retina_BXD_RankInv0410',ProbeSetID2='ILMN_2583882',CellID2='',rank='1')) | [**0.483 79**](javascript:showCorrelationPlot2(db='Illum_Retina_BXD_RankInv0410',ProbeSetID='ILMN_1258455',CellID='',db2='Illum_Retina_BXD_RankInv0410',ProbeSetID2='ILMN_2915166',CellID2='',rank='1')) | [**0.668 79**](javascript:showCorrelationPlot2(db='Illum_Retina_BXD_RankInv0410',ProbeSetID='ILMN_1258455',CellID='',db2='Illum_Retina_BXD_RankInv0410',ProbeSetID2='ILMN_2702997',CellID2='',rank='1')) | [**0.547 79**](javascript:showCorrelationPlot2(db='Illum_Retina_BXD_RankInv0410',ProbeSetID='ILMN_1258455',CellID='',db2='Illum_Retina_BXD_RankInv0410',ProbeSetID2='ILMN_2435206',CellID2='',rank='1')) | [**0.531 79**](javascript:showCorrelationPlot2(db='Illum_Retina_BXD_RankInv0410',ProbeSetID='ILMN_1258455',CellID='',db2='Illum_Retina_BXD_RankInv0410',ProbeSetID2='ILMN_2789888',CellID2='',rank='1')) | [**0.660 79**](javascript:showCorrelationPlot2(db='Illum_Retina_BXD_RankInv0410',ProbeSetID='ILMN_1258455',CellID='',db2='Illum_Retina_BXD_RankInv0410',ProbeSetID2='ILMN_1218901',CellID2='',rank='1')) | [**0.564 79**](javascript:showCorrelationPlot2(db='Illum_Retina_BXD_RankInv0410',ProbeSetID='ILMN_1258455',CellID='',db2='Illum_Retina_BXD_RankInv0410',ProbeSetID2='ILMN_1254734',CellID2='',rank='1')) | [**0.433 79**](javascript:showCorrelationPlot2(db='Illum_Retina_BXD_RankInv0410',ProbeSetID='ILMN_1258455',CellID='',db2='Illum_Retina_BXD_RankInv0410',ProbeSetID2='ILMN_3008924',CellID2='',rank='1')) | [**0.388 79**](javascript:showCorrelationPlot2(db='Illum_Retina_BXD_RankInv0410',ProbeSetID='ILMN_1258455',CellID='',db2='Illum_Retina_BXD_RankInv0410',ProbeSetID2='ILMN_2628175',CellID2='',rank='1')) | [**0.342 79**](javascript:showCorrelationPlot2(db='Illum_Retina_BXD_RankInv0410',ProbeSetID='ILMN_1258455',CellID='',db2='Illum_Retina_BXD_RankInv0410',ProbeSetID2='ILMN_2891573',CellID2='',rank='1')) | [**0.425 79**](javascript:showCorrelationPlot2(db='Illum_Retina_BXD_RankInv0410',ProbeSetID='ILMN_1258455',CellID='',db2='Illum_Retina_BXD_RankInv0410',ProbeSetID2='ILMN_1220846',CellID2='',rank='1')) | [**0.337 79**](javascript:showCorrelationPlot2(db='Illum_Retina_BXD_RankInv0410',ProbeSetID='ILMN_1258455',CellID='',db2='Illum_Retina_BXD_RankInv0410',ProbeSetID2='ILMN_1241962',CellID2='',rank='1')) | [**0.493 79**](javascript:showCorrelationPlot2(db='Illum_Retina_BXD_RankInv0410',ProbeSetID='ILMN_1258455',CellID='',db2='Illum_Retina_BXD_RankInv0410',ProbeSetID2='ILMN_3163027',CellID2='',rank='1')) | [**0.574 79**](javascript:showCorrelationPlot2(db='Illum_Retina_BXD_RankInv0410',ProbeSetID='ILMN_1258455',CellID='',db2='Illum_Retina_BXD_RankInv0410',ProbeSetID2='ILMN_2775098',CellID2='',rank='1')) | [**0.413 79**](javascript:showCorrelationPlot2(db='Illum_Retina_BXD_RankInv0410',ProbeSetID='ILMN_1258455',CellID='',db2='Illum_Retina_BXD_RankInv0410',ProbeSetID2='ILMN_2752552',CellID2='',rank='1')) | [**0.684 79**](javascript:showCorrelationPlot2(db='Illum_Retina_BXD_RankInv0410',ProbeSetID='ILMN_1258455',CellID='',db2='Illum_Retina_BXD_RankInv0410',ProbeSetID2='ILMN_2730293',CellID2='',rank='1')) | [**0.436 79**](javascript:showCorrelationPlot2(db='Illum_Retina_BXD_RankInv0410',ProbeSetID='ILMN_1258455',CellID='',db2='Illum_Retina_BXD_RankInv0410',ProbeSetID2='ILMN_2680872',CellID2='',rank='1')) | [**0.210 79**](javascript:showCorrelationPlot2(db='Illum_Retina_BXD_RankInv0410',ProbeSetID='ILMN_1258455',CellID='',db2='Illum_Retina_BXD_RankInv0410',ProbeSetID2='ILMN_2768533',CellID2='',rank='1')) | [**-0.397 79**](javascript:showCorrelationPlot2(db='Illum_Retina_BXD_RankInv0410',ProbeSetID='ILMN_1258455',CellID='',db2='Illum_Retina_BXD_RankInv0410',ProbeSetID2='ILMN_3121255',CellID2='',rank='1')) | [**-0.200 79**](javascript:showCorrelationPlot2(db='Illum_Retina_BXD_RankInv0410',ProbeSetID='ILMN_1258455',CellID='',db2='Illum_Retina_BXD_RankInv0410',ProbeSetID2='ILMN_2484527',CellID2='',rank='1')) | [**-0.018 79**](javascript:showCorrelationPlot2(db='Illum_Retina_BXD_RankInv0410',ProbeSetID='ILMN_1258455',CellID='',db2='Illum_Retina_BXD_RankInv0410',ProbeSetID2='ILMN_3045723',CellID2='',rank='1')) | [**0.594 79**](javascript:showCorrelationPlot2(db='Illum_Retina_BXD_RankInv0410',ProbeSetID='ILMN_1258455',CellID='',db2='Illum_Retina_BXD_RankInv0410',ProbeSetID2='ILMN_2486573',CellID2='',rank='1')) |
|  | [**ILMN_2691613**](javascript:showDatabase2('Illum_Retina_BXD_RankInv0410','ILMN_2691613','');)  Symbol: Ppp1ca  Ppp1ca on | [**0.675 79**](javascript:showCorrelationPlot2(db='Illum_Retina_BXD_RankInv0410',ProbeSetID='ILMN_2691613',CellID='',db2='Illum_Retina_BXD_RankInv0410',ProbeSetID2='ILMN_2450384',CellID2='',rank='0')) | [**0.610 79**](javascript:showCorrelationPlot2(db='Illum_Retina_BXD_RankInv0410',ProbeSetID='ILMN_2691613',CellID='',db2='Illum_Retina_BXD_RankInv0410',ProbeSetID2='ILMN_3068754',CellID2='',rank='0')) | [**0.612 79**](javascript:showCorrelationPlot2(db='Illum_Retina_BXD_RankInv0410',ProbeSetID='ILMN_2691613',CellID='',db2='Illum_Retina_BXD_RankInv0410',ProbeSetID2='ILMN_2677092',CellID2='',rank='0')) | [**0.655 79**](javascript:showCorrelationPlot2(db='Illum_Retina_BXD_RankInv0410',ProbeSetID='ILMN_2691613',CellID='',db2='Illum_Retina_BXD_RankInv0410',ProbeSetID2='ILMN_2624938',CellID2='',rank='0')) | [**0.772 79**](javascript:showCorrelationPlot2(db='Illum_Retina_BXD_RankInv0410',ProbeSetID='ILMN_2691613',CellID='',db2='Illum_Retina_BXD_RankInv0410',ProbeSetID2='ILMN_2847269',CellID2='',rank='0')) | [**0.594 79**](javascript:showCorrelationPlot2(db='Illum_Retina_BXD_RankInv0410',ProbeSetID='ILMN_2691613',CellID='',db2='Illum_Retina_BXD_RankInv0410',ProbeSetID2='ILMN_2839682',CellID2='',rank='0')) | [**0.823 79**](javascript:showCorrelationPlot2(db='Illum_Retina_BXD_RankInv0410',ProbeSetID='ILMN_2691613',CellID='',db2='Illum_Retina_BXD_RankInv0410',ProbeSetID2='ILMN_3147135',CellID2='',rank='0')) | [**0.741 79**](javascript:showCorrelationPlot2(db='Illum_Retina_BXD_RankInv0410',ProbeSetID='ILMN_2691613',CellID='',db2='Illum_Retina_BXD_RankInv0410',ProbeSetID2='ILMN_1255823',CellID2='',rank='0')) | [**0.546 79**](javascript:showCorrelationPlot2(db='Illum_Retina_BXD_RankInv0410',ProbeSetID='ILMN_2691613',CellID='',db2='Illum_Retina_BXD_RankInv0410',ProbeSetID2='ILMN_2626143',CellID2='',rank='0')) | [**0.580 79**](javascript:showCorrelationPlot2(db='Illum_Retina_BXD_RankInv0410',ProbeSetID='ILMN_2691613',CellID='',db2='Illum_Retina_BXD_RankInv0410',ProbeSetID2='ILMN_1234930',CellID2='',rank='0')) | [**0.728 79**](javascript:showCorrelationPlot2(db='Illum_Retina_BXD_RankInv0410',ProbeSetID='ILMN_2691613',CellID='',db2='Illum_Retina_BXD_RankInv0410',ProbeSetID2='ILMN_1249408',CellID2='',rank='0')) | [**0.531 79**](javascript:showCorrelationPlot2(db='Illum_Retina_BXD_RankInv0410',ProbeSetID='ILMN_2691613',CellID='',db2='Illum_Retina_BXD_RankInv0410',ProbeSetID2='ILMN_2971559',CellID2='',rank='0')) | [**0.234 79**](javascript:showCorrelationPlot2(db='Illum_Retina_BXD_RankInv0410',ProbeSetID='ILMN_2691613',CellID='',db2='Illum_Retina_BXD_RankInv0410',ProbeSetID2='ILMN_1258455',CellID2='',rank='0')) | [***n* 79**](javascript:showDatabase2('Illum_Retina_BXD_RankInv0410','ILMN_2691613','')) | [**0.484 79**](javascript:showCorrelationPlot2(db='Illum_Retina_BXD_RankInv0410',ProbeSetID='ILMN_2691613',CellID='',db2='Illum_Retina_BXD_RankInv0410',ProbeSetID2='ILMN_1253600',CellID2='',rank='1')) | [**0.626 79**](javascript:showCorrelationPlot2(db='Illum_Retina_BXD_RankInv0410',ProbeSetID='ILMN_2691613',CellID='',db2='Illum_Retina_BXD_RankInv0410',ProbeSetID2='ILMN_2991545',CellID2='',rank='1')) | [**0.523 79**](javascript:showCorrelationPlot2(db='Illum_Retina_BXD_RankInv0410',ProbeSetID='ILMN_2691613',CellID='',db2='Illum_Retina_BXD_RankInv0410',ProbeSetID2='ILMN_2623591',CellID2='',rank='1')) | [**0.516 79**](javascript:showCorrelationPlot2(db='Illum_Retina_BXD_RankInv0410',ProbeSetID='ILMN_2691613',CellID='',db2='Illum_Retina_BXD_RankInv0410',ProbeSetID2='ILMN_2603568',CellID2='',rank='1')) | [**0.824 79**](javascript:showCorrelationPlot2(db='Illum_Retina_BXD_RankInv0410',ProbeSetID='ILMN_2691613',CellID='',db2='Illum_Retina_BXD_RankInv0410',ProbeSetID2='ILMN_2992541',CellID2='',rank='1')) | [**0.509 79**](javascript:showCorrelationPlot2(db='Illum_Retina_BXD_RankInv0410',ProbeSetID='ILMN_2691613',CellID='',db2='Illum_Retina_BXD_RankInv0410',ProbeSetID2='ILMN_2792868',CellID2='',rank='1')) | [**0.431 79**](javascript:showCorrelationPlot2(db='Illum_Retina_BXD_RankInv0410',ProbeSetID='ILMN_2691613',CellID='',db2='Illum_Retina_BXD_RankInv0410',ProbeSetID2='ILMN_3129497',CellID2='',rank='1')) | [**0.412 79**](javascript:showCorrelationPlot2(db='Illum_Retina_BXD_RankInv0410',ProbeSetID='ILMN_2691613',CellID='',db2='Illum_Retina_BXD_RankInv0410',ProbeSetID2='ILMN_1224034',CellID2='',rank='1')) | [**0.515 79**](javascript:showCorrelationPlot2(db='Illum_Retina_BXD_RankInv0410',ProbeSetID='ILMN_2691613',CellID='',db2='Illum_Retina_BXD_RankInv0410',ProbeSetID2='ILMN_2639819',CellID2='',rank='1')) | [**0.638 79**](javascript:showCorrelationPlot2(db='Illum_Retina_BXD_RankInv0410',ProbeSetID='ILMN_2691613',CellID='',db2='Illum_Retina_BXD_RankInv0410',ProbeSetID2='ILMN_2994779',CellID2='',rank='1')) | [**0.539 79**](javascript:showCorrelationPlot2(db='Illum_Retina_BXD_RankInv0410',ProbeSetID='ILMN_2691613',CellID='',db2='Illum_Retina_BXD_RankInv0410',ProbeSetID2='ILMN_2625047',CellID2='',rank='1')) | [**0.354 79**](javascript:showCorrelationPlot2(db='Illum_Retina_BXD_RankInv0410',ProbeSetID='ILMN_2691613',CellID='',db2='Illum_Retina_BXD_RankInv0410',ProbeSetID2='ILMN_2808186',CellID2='',rank='1')) | [**0.536 79**](javascript:showCorrelationPlot2(db='Illum_Retina_BXD_RankInv0410',ProbeSetID='ILMN_2691613',CellID='',db2='Illum_Retina_BXD_RankInv0410',ProbeSetID2='ILMN_2689056',CellID2='',rank='1')) | [**0.693 79**](javascript:showCorrelationPlot2(db='Illum_Retina_BXD_RankInv0410',ProbeSetID='ILMN_2691613',CellID='',db2='Illum_Retina_BXD_RankInv0410',ProbeSetID2='ILMN_2648386',CellID2='',rank='1')) | [**0.642 79**](javascript:showCorrelationPlot2(db='Illum_Retina_BXD_RankInv0410',ProbeSetID='ILMN_2691613',CellID='',db2='Illum_Retina_BXD_RankInv0410',ProbeSetID2='ILMN_2869225',CellID2='',rank='1')) | [**0.581 79**](javascript:showCorrelationPlot2(db='Illum_Retina_BXD_RankInv0410',ProbeSetID='ILMN_2691613',CellID='',db2='Illum_Retina_BXD_RankInv0410',ProbeSetID2='ILMN_2728431',CellID2='',rank='1')) | [**0.372 79**](javascript:showCorrelationPlot2(db='Illum_Retina_BXD_RankInv0410',ProbeSetID='ILMN_2691613',CellID='',db2='Illum_Retina_BXD_RankInv0410',ProbeSetID2='ILMN_3094608',CellID2='',rank='1')) | [**0.690 79**](javascript:showCorrelationPlot2(db='Illum_Retina_BXD_RankInv0410',ProbeSetID='ILMN_2691613',CellID='',db2='Illum_Retina_BXD_RankInv0410',ProbeSetID2='ILMN_2896528',CellID2='',rank='1')) | [**0.322 79**](javascript:showCorrelationPlot2(db='Illum_Retina_BXD_RankInv0410',ProbeSetID='ILMN_2691613',CellID='',db2='Illum_Retina_BXD_RankInv0410',ProbeSetID2='ILMN_2701750',CellID2='',rank='1')) | [**0.461 79**](javascript:showCorrelationPlot2(db='Illum_Retina_BXD_RankInv0410',ProbeSetID='ILMN_2691613',CellID='',db2='Illum_Retina_BXD_RankInv0410',ProbeSetID2='ILMN_1239448',CellID2='',rank='1')) | [**-0.667 79**](javascript:showCorrelationPlot2(db='Illum_Retina_BXD_RankInv0410',ProbeSetID='ILMN_2691613',CellID='',db2='Illum_Retina_BXD_RankInv0410',ProbeSetID2='ILMN_2583882',CellID2='',rank='1')) | [**0.476 79**](javascript:showCorrelationPlot2(db='Illum_Retina_BXD_RankInv0410',ProbeSetID='ILMN_2691613',CellID='',db2='Illum_Retina_BXD_RankInv0410',ProbeSetID2='ILMN_2915166',CellID2='',rank='1')) | [**0.352 79**](javascript:showCorrelationPlot2(db='Illum_Retina_BXD_RankInv0410',ProbeSetID='ILMN_2691613',CellID='',db2='Illum_Retina_BXD_RankInv0410',ProbeSetID2='ILMN_2702997',CellID2='',rank='1')) | [**0.373 79**](javascript:showCorrelationPlot2(db='Illum_Retina_BXD_RankInv0410',ProbeSetID='ILMN_2691613',CellID='',db2='Illum_Retina_BXD_RankInv0410',ProbeSetID2='ILMN_2435206',CellID2='',rank='1')) | [**0.515 79**](javascript:showCorrelationPlot2(db='Illum_Retina_BXD_RankInv0410',ProbeSetID='ILMN_2691613',CellID='',db2='Illum_Retina_BXD_RankInv0410',ProbeSetID2='ILMN_2789888',CellID2='',rank='1')) | [**0.369 79**](javascript:showCorrelationPlot2(db='Illum_Retina_BXD_RankInv0410',ProbeSetID='ILMN_2691613',CellID='',db2='Illum_Retina_BXD_RankInv0410',ProbeSetID2='ILMN_1218901',CellID2='',rank='1')) | [**0.587 79**](javascript:showCorrelationPlot2(db='Illum_Retina_BXD_RankInv0410',ProbeSetID='ILMN_2691613',CellID='',db2='Illum_Retina_BXD_RankInv0410',ProbeSetID2='ILMN_1254734',CellID2='',rank='1')) | [**0.426 79**](javascript:showCorrelationPlot2(db='Illum_Retina_BXD_RankInv0410',ProbeSetID='ILMN_2691613',CellID='',db2='Illum_Retina_BXD_RankInv0410',ProbeSetID2='ILMN_3008924',CellID2='',rank='1')) | [**0.585 79**](javascript:showCorrelationPlot2(db='Illum_Retina_BXD_RankInv0410',ProbeSetID='ILMN_2691613',CellID='',db2='Illum_Retina_BXD_RankInv0410',ProbeSetID2='ILMN_2628175',CellID2='',rank='1')) | [**0.670 79**](javascript:showCorrelationPlot2(db='Illum_Retina_BXD_RankInv0410',ProbeSetID='ILMN_2691613',CellID='',db2='Illum_Retina_BXD_RankInv0410',ProbeSetID2='ILMN_2891573',CellID2='',rank='1')) | [**0.576 79**](javascript:showCorrelationPlot2(db='Illum_Retina_BXD_RankInv0410',ProbeSetID='ILMN_2691613',CellID='',db2='Illum_Retina_BXD_RankInv0410',ProbeSetID2='ILMN_1220846',CellID2='',rank='1')) | [**0.647 79**](javascript:showCorrelationPlot2(db='Illum_Retina_BXD_RankInv0410',ProbeSetID='ILMN_2691613',CellID='',db2='Illum_Retina_BXD_RankInv0410',ProbeSetID2='ILMN_1241962',CellID2='',rank='1')) | [**0.646 79**](javascript:showCorrelationPlot2(db='Illum_Retina_BXD_RankInv0410',ProbeSetID='ILMN_2691613',CellID='',db2='Illum_Retina_BXD_RankInv0410',ProbeSetID2='ILMN_3163027',CellID2='',rank='1')) | [**0.374 79**](javascript:showCorrelationPlot2(db='Illum_Retina_BXD_RankInv0410',ProbeSetID='ILMN_2691613',CellID='',db2='Illum_Retina_BXD_RankInv0410',ProbeSetID2='ILMN_2775098',CellID2='',rank='1')) | [**0.573 79**](javascript:showCorrelationPlot2(db='Illum_Retina_BXD_RankInv0410',ProbeSetID='ILMN_2691613',CellID='',db2='Illum_Retina_BXD_RankInv0410',ProbeSetID2='ILMN_2752552',CellID2='',rank='1')) | [**0.416 79**](javascript:showCorrelationPlot2(db='Illum_Retina_BXD_RankInv0410',ProbeSetID='ILMN_2691613',CellID='',db2='Illum_Retina_BXD_RankInv0410',ProbeSetID2='ILMN_2730293',CellID2='',rank='1')) | [**0.424 79**](javascript:showCorrelationPlot2(db='Illum_Retina_BXD_RankInv0410',ProbeSetID='ILMN_2691613',CellID='',db2='Illum_Retina_BXD_RankInv0410',ProbeSetID2='ILMN_2680872',CellID2='',rank='1')) | [**0.217 79**](javascript:showCorrelationPlot2(db='Illum_Retina_BXD_RankInv0410',ProbeSetID='ILMN_2691613',CellID='',db2='Illum_Retina_BXD_RankInv0410',ProbeSetID2='ILMN_2768533',CellID2='',rank='1')) | [**0.079 79**](javascript:showCorrelationPlot2(db='Illum_Retina_BXD_RankInv0410',ProbeSetID='ILMN_2691613',CellID='',db2='Illum_Retina_BXD_RankInv0410',ProbeSetID2='ILMN_3121255',CellID2='',rank='1')) | [**-0.361 79**](javascript:showCorrelationPlot2(db='Illum_Retina_BXD_RankInv0410',ProbeSetID='ILMN_2691613',CellID='',db2='Illum_Retina_BXD_RankInv0410',ProbeSetID2='ILMN_2484527',CellID2='',rank='1')) | [**0.418 79**](javascript:showCorrelationPlot2(db='Illum_Retina_BXD_RankInv0410',ProbeSetID='ILMN_2691613',CellID='',db2='Illum_Retina_BXD_RankInv0410',ProbeSetID2='ILMN_3045723',CellID2='',rank='1')) | [**0.088 79**](javascript:showCorrelationPlot2(db='Illum_Retina_BXD_RankInv0410',ProbeSetID='ILMN_2691613',CellID='',db2='Illum_Retina_BXD_RankInv0410',ProbeSetID2='ILMN_2486573',CellID2='',rank='1')) |
|  | [**Trait 15: ILMN_1253600**](javascript:showDatabase2('Illum_Retina_BXD_RankInv0410','ILMN_1253600','');)  Symbol: Trnp1  Trnp1 | [**0.675 79**](javascript:showCorrelationPlot2(db='Illum_Retina_BXD_RankInv0410',ProbeSetID='ILMN_1253600',CellID='',db2='Illum_Retina_BXD_RankInv0410',ProbeSetID2='ILMN_2450384',CellID2='',rank='0')) | [**0.649 79**](javascript:showCorrelationPlot2(db='Illum_Retina_BXD_RankInv0410',ProbeSetID='ILMN_1253600',CellID='',db2='Illum_Retina_BXD_RankInv0410',ProbeSetID2='ILMN_3068754',CellID2='',rank='0')) | [**0.649 79**](javascript:showCorrelationPlot2(db='Illum_Retina_BXD_RankInv0410',ProbeSetID='ILMN_1253600',CellID='',db2='Illum_Retina_BXD_RankInv0410',ProbeSetID2='ILMN_2677092',CellID2='',rank='0')) | [**0.626 79**](javascript:showCorrelationPlot2(db='Illum_Retina_BXD_RankInv0410',ProbeSetID='ILMN_1253600',CellID='',db2='Illum_Retina_BXD_RankInv0410',ProbeSetID2='ILMN_2624938',CellID2='',rank='0')) | [**0.468 79**](javascript:showCorrelationPlot2(db='Illum_Retina_BXD_RankInv0410',ProbeSetID='ILMN_1253600',CellID='',db2='Illum_Retina_BXD_RankInv0410',ProbeSetID2='ILMN_2847269',CellID2='',rank='0')) | [**0.639 79**](javascript:showCorrelationPlot2(db='Illum_Retina_BXD_RankInv0410',ProbeSetID='ILMN_1253600',CellID='',db2='Illum_Retina_BXD_RankInv0410',ProbeSetID2='ILMN_2839682',CellID2='',rank='0')) | [**0.460 79**](javascript:showCorrelationPlot2(db='Illum_Retina_BXD_RankInv0410',ProbeSetID='ILMN_1253600',CellID='',db2='Illum_Retina_BXD_RankInv0410',ProbeSetID2='ILMN_3147135',CellID2='',rank='0')) | [**0.439 79**](javascript:showCorrelationPlot2(db='Illum_Retina_BXD_RankInv0410',ProbeSetID='ILMN_1253600',CellID='',db2='Illum_Retina_BXD_RankInv0410',ProbeSetID2='ILMN_1255823',CellID2='',rank='0')) | [**0.480 79**](javascript:showCorrelationPlot2(db='Illum_Retina_BXD_RankInv0410',ProbeSetID='ILMN_1253600',CellID='',db2='Illum_Retina_BXD_RankInv0410',ProbeSetID2='ILMN_2626143',CellID2='',rank='0')) | [**0.667 79**](javascript:showCorrelationPlot2(db='Illum_Retina_BXD_RankInv0410',ProbeSetID='ILMN_1253600',CellID='',db2='Illum_Retina_BXD_RankInv0410',ProbeSetID2='ILMN_1234930',CellID2='',rank='0')) | [**0.509 79**](javascript:showCorrelationPlot2(db='Illum_Retina_BXD_RankInv0410',ProbeSetID='ILMN_1253600',CellID='',db2='Illum_Retina_BXD_RankInv0410',ProbeSetID2='ILMN_1249408',CellID2='',rank='0')) | [**0.584 79**](javascript:showCorrelationPlot2(db='Illum_Retina_BXD_RankInv0410',ProbeSetID='ILMN_1253600',CellID='',db2='Illum_Retina_BXD_RankInv0410',ProbeSetID2='ILMN_2971559',CellID2='',rank='0')) | [**0.551 79**](javascript:showCorrelationPlot2(db='Illum_Retina_BXD_RankInv0410',ProbeSetID='ILMN_1253600',CellID='',db2='Illum_Retina_BXD_RankInv0410',ProbeSetID2='ILMN_1258455',CellID2='',rank='0')) | [**0.529 79**](javascript:showCorrelationPlot2(db='Illum_Retina_BXD_RankInv0410',ProbeSetID='ILMN_1253600',CellID='',db2='Illum_Retina_BXD_RankInv0410',ProbeSetID2='ILMN_2691613',CellID2='',rank='0')) | [***n* 79**](javascript:showDatabase2('Illum_Retina_BXD_RankInv0410','ILMN_1253600','')) | [**0.553 79**](javascript:showCorrelationPlot2(db='Illum_Retina_BXD_RankInv0410',ProbeSetID='ILMN_1253600',CellID='',db2='Illum_Retina_BXD_RankInv0410',ProbeSetID2='ILMN_2991545',CellID2='',rank='1')) | [**0.760 79**](javascript:showCorrelationPlot2(db='Illum_Retina_BXD_RankInv0410',ProbeSetID='ILMN_1253600',CellID='',db2='Illum_Retina_BXD_RankInv0410',ProbeSetID2='ILMN_2623591',CellID2='',rank='1')) | [**0.565 79**](javascript:showCorrelationPlot2(db='Illum_Retina_BXD_RankInv0410',ProbeSetID='ILMN_1253600',CellID='',db2='Illum_Retina_BXD_RankInv0410',ProbeSetID2='ILMN_2603568',CellID2='',rank='1')) | [**0.422 79**](javascript:showCorrelationPlot2(db='Illum_Retina_BXD_RankInv0410',ProbeSetID='ILMN_1253600',CellID='',db2='Illum_Retina_BXD_RankInv0410',ProbeSetID2='ILMN_2992541',CellID2='',rank='1')) | [**0.488 79**](javascript:showCorrelationPlot2(db='Illum_Retina_BXD_RankInv0410',ProbeSetID='ILMN_1253600',CellID='',db2='Illum_Retina_BXD_RankInv0410',ProbeSetID2='ILMN_2792868',CellID2='',rank='1')) | [**0.633 79**](javascript:showCorrelationPlot2(db='Illum_Retina_BXD_RankInv0410',ProbeSetID='ILMN_1253600',CellID='',db2='Illum_Retina_BXD_RankInv0410',ProbeSetID2='ILMN_3129497',CellID2='',rank='1')) | [**0.604 79**](javascript:showCorrelationPlot2(db='Illum_Retina_BXD_RankInv0410',ProbeSetID='ILMN_1253600',CellID='',db2='Illum_Retina_BXD_RankInv0410',ProbeSetID2='ILMN_1224034',CellID2='',rank='1')) | [**0.544 79**](javascript:showCorrelationPlot2(db='Illum_Retina_BXD_RankInv0410',ProbeSetID='ILMN_1253600',CellID='',db2='Illum_Retina_BXD_RankInv0410',ProbeSetID2='ILMN_2639819',CellID2='',rank='1')) | [**0.392 79**](javascript:showCorrelationPlot2(db='Illum_Retina_BXD_RankInv0410',ProbeSetID='ILMN_1253600',CellID='',db2='Illum_Retina_BXD_RankInv0410',ProbeSetID2='ILMN_2994779',CellID2='',rank='1')) | [**0.538 79**](javascript:showCorrelationPlot2(db='Illum_Retina_BXD_RankInv0410',ProbeSetID='ILMN_1253600',CellID='',db2='Illum_Retina_BXD_RankInv0410',ProbeSetID2='ILMN_2625047',CellID2='',rank='1')) | [**0.644 79**](javascript:showCorrelationPlot2(db='Illum_Retina_BXD_RankInv0410',ProbeSetID='ILMN_1253600',CellID='',db2='Illum_Retina_BXD_RankInv0410',ProbeSetID2='ILMN_2808186',CellID2='',rank='1')) | [**0.636 79**](javascript:showCorrelationPlot2(db='Illum_Retina_BXD_RankInv0410',ProbeSetID='ILMN_1253600',CellID='',db2='Illum_Retina_BXD_RankInv0410',ProbeSetID2='ILMN_2689056',CellID2='',rank='1')) | [**0.513 79**](javascript:showCorrelationPlot2(db='Illum_Retina_BXD_RankInv0410',ProbeSetID='ILMN_1253600',CellID='',db2='Illum_Retina_BXD_RankInv0410',ProbeSetID2='ILMN_2648386',CellID2='',rank='1')) | [**0.412 79**](javascript:showCorrelationPlot2(db='Illum_Retina_BXD_RankInv0410',ProbeSetID='ILMN_1253600',CellID='',db2='Illum_Retina_BXD_RankInv0410',ProbeSetID2='ILMN_2869225',CellID2='',rank='1')) | [**0.619 79**](javascript:showCorrelationPlot2(db='Illum_Retina_BXD_RankInv0410',ProbeSetID='ILMN_1253600',CellID='',db2='Illum_Retina_BXD_RankInv0410',ProbeSetID2='ILMN_2728431',CellID2='',rank='1')) | [**0.606 79**](javascript:showCorrelationPlot2(db='Illum_Retina_BXD_RankInv0410',ProbeSetID='ILMN_1253600',CellID='',db2='Illum_Retina_BXD_RankInv0410',ProbeSetID2='ILMN_3094608',CellID2='',rank='1')) | [**0.343 79**](javascript:showCorrelationPlot2(db='Illum_Retina_BXD_RankInv0410',ProbeSetID='ILMN_1253600',CellID='',db2='Illum_Retina_BXD_RankInv0410',ProbeSetID2='ILMN_2896528',CellID2='',rank='1')) | [**0.427 79**](javascript:showCorrelationPlot2(db='Illum_Retina_BXD_RankInv0410',ProbeSetID='ILMN_1253600',CellID='',db2='Illum_Retina_BXD_RankInv0410',ProbeSetID2='ILMN_2701750',CellID2='',rank='1')) | [**0.543 79**](javascript:showCorrelationPlot2(db='Illum_Retina_BXD_RankInv0410',ProbeSetID='ILMN_1253600',CellID='',db2='Illum_Retina_BXD_RankInv0410',ProbeSetID2='ILMN_1239448',CellID2='',rank='1')) | [**-0.486 79**](javascript:showCorrelationPlot2(db='Illum_Retina_BXD_RankInv0410',ProbeSetID='ILMN_1253600',CellID='',db2='Illum_Retina_BXD_RankInv0410',ProbeSetID2='ILMN_2583882',CellID2='',rank='1')) | [**0.316 79**](javascript:showCorrelationPlot2(db='Illum_Retina_BXD_RankInv0410',ProbeSetID='ILMN_1253600',CellID='',db2='Illum_Retina_BXD_RankInv0410',ProbeSetID2='ILMN_2915166',CellID2='',rank='1')) | [**0.520 79**](javascript:showCorrelationPlot2(db='Illum_Retina_BXD_RankInv0410',ProbeSetID='ILMN_1253600',CellID='',db2='Illum_Retina_BXD_RankInv0410',ProbeSetID2='ILMN_2702997',CellID2='',rank='1')) | [**0.500 79**](javascript:showCorrelationPlot2(db='Illum_Retina_BXD_RankInv0410',ProbeSetID='ILMN_1253600',CellID='',db2='Illum_Retina_BXD_RankInv0410',ProbeSetID2='ILMN_2435206',CellID2='',rank='1')) | [**0.687 79**](javascript:showCorrelationPlot2(db='Illum_Retina_BXD_RankInv0410',ProbeSetID='ILMN_1253600',CellID='',db2='Illum_Retina_BXD_RankInv0410',ProbeSetID2='ILMN_2789888',CellID2='',rank='1')) | [**0.534 79**](javascript:showCorrelationPlot2(db='Illum_Retina_BXD_RankInv0410',ProbeSetID='ILMN_1253600',CellID='',db2='Illum_Retina_BXD_RankInv0410',ProbeSetID2='ILMN_1218901',CellID2='',rank='1')) | [**0.440 79**](javascript:showCorrelationPlot2(db='Illum_Retina_BXD_RankInv0410',ProbeSetID='ILMN_1253600',CellID='',db2='Illum_Retina_BXD_RankInv0410',ProbeSetID2='ILMN_1254734',CellID2='',rank='1')) | [**0.688 79**](javascript:showCorrelationPlot2(db='Illum_Retina_BXD_RankInv0410',ProbeSetID='ILMN_1253600',CellID='',db2='Illum_Retina_BXD_RankInv0410',ProbeSetID2='ILMN_3008924',CellID2='',rank='1')) | [**0.611 79**](javascript:showCorrelationPlot2(db='Illum_Retina_BXD_RankInv0410',ProbeSetID='ILMN_1253600',CellID='',db2='Illum_Retina_BXD_RankInv0410',ProbeSetID2='ILMN_2628175',CellID2='',rank='1')) | [**0.444 79**](javascript:showCorrelationPlot2(db='Illum_Retina_BXD_RankInv0410',ProbeSetID='ILMN_1253600',CellID='',db2='Illum_Retina_BXD_RankInv0410',ProbeSetID2='ILMN_2891573',CellID2='',rank='1')) | [**0.526 79**](javascript:showCorrelationPlot2(db='Illum_Retina_BXD_RankInv0410',ProbeSetID='ILMN_1253600',CellID='',db2='Illum_Retina_BXD_RankInv0410',ProbeSetID2='ILMN_1220846',CellID2='',rank='1')) | [**0.425 79**](javascript:showCorrelationPlot2(db='Illum_Retina_BXD_RankInv0410',ProbeSetID='ILMN_1253600',CellID='',db2='Illum_Retina_BXD_RankInv0410',ProbeSetID2='ILMN_1241962',CellID2='',rank='1')) | [**0.423 79**](javascript:showCorrelationPlot2(db='Illum_Retina_BXD_RankInv0410',ProbeSetID='ILMN_1253600',CellID='',db2='Illum_Retina_BXD_RankInv0410',ProbeSetID2='ILMN_3163027',CellID2='',rank='1')) | [**0.303 79**](javascript:showCorrelationPlot2(db='Illum_Retina_BXD_RankInv0410',ProbeSetID='ILMN_1253600',CellID='',db2='Illum_Retina_BXD_RankInv0410',ProbeSetID2='ILMN_2775098',CellID2='',rank='1')) | [**0.470 79**](javascript:showCorrelationPlot2(db='Illum_Retina_BXD_RankInv0410',ProbeSetID='ILMN_1253600',CellID='',db2='Illum_Retina_BXD_RankInv0410',ProbeSetID2='ILMN_2752552',CellID2='',rank='1')) | [**0.599 79**](javascript:showCorrelationPlot2(db='Illum_Retina_BXD_RankInv0410',ProbeSetID='ILMN_1253600',CellID='',db2='Illum_Retina_BXD_RankInv0410',ProbeSetID2='ILMN_2730293',CellID2='',rank='1')) | [**0.357 79**](javascript:showCorrelationPlot2(db='Illum_Retina_BXD_RankInv0410',ProbeSetID='ILMN_1253600',CellID='',db2='Illum_Retina_BXD_RankInv0410',ProbeSetID2='ILMN_2680872',CellID2='',rank='1')) | [**0.105 79**](javascript:showCorrelationPlot2(db='Illum_Retina_BXD_RankInv0410',ProbeSetID='ILMN_1253600',CellID='',db2='Illum_Retina_BXD_RankInv0410',ProbeSetID2='ILMN_2768533',CellID2='',rank='1')) | [**-0.102 79**](javascript:showCorrelationPlot2(db='Illum_Retina_BXD_RankInv0410',ProbeSetID='ILMN_1253600',CellID='',db2='Illum_Retina_BXD_RankInv0410',ProbeSetID2='ILMN_3121255',CellID2='',rank='1')) | [**-0.229 79**](javascript:showCorrelationPlot2(db='Illum_Retina_BXD_RankInv0410',ProbeSetID='ILMN_1253600',CellID='',db2='Illum_Retina_BXD_RankInv0410',ProbeSetID2='ILMN_2484527',CellID2='',rank='1')) | [**0.095 79**](javascript:showCorrelationPlot2(db='Illum_Retina_BXD_RankInv0410',ProbeSetID='ILMN_1253600',CellID='',db2='Illum_Retina_BXD_RankInv0410',ProbeSetID2='ILMN_3045723',CellID2='',rank='1')) | [**0.249 79**](javascript:showCorrelationPlot2(db='Illum_Retina_BXD_RankInv0410',ProbeSetID='ILMN_1253600',CellID='',db2='Illum_Retina_BXD_RankInv0410',ProbeSetID2='ILMN_2486573',CellID2='',rank='1')) |
|  | [**Trait 16: ILMN_2991545**](javascript:showDatabase2('Illum_Retina_BXD_RankInv0410','ILMN_2991545','');)  Symbol: D10Jhu81e  D10Jhu81e | [**0.674 79**](javascript:showCorrelationPlot2(db='Illum_Retina_BXD_RankInv0410',ProbeSetID='ILMN_2991545',CellID='',db2='Illum_Retina_BXD_RankInv0410',ProbeSetID2='ILMN_2450384',CellID2='',rank='0')) | [**0.669 79**](javascript:showCorrelationPlot2(db='Illum_Retina_BXD_RankInv0410',ProbeSetID='ILMN_2991545',CellID='',db2='Illum_Retina_BXD_RankInv0410',ProbeSetID2='ILMN_3068754',CellID2='',rank='0')) | [**0.614 79**](javascript:showCorrelationPlot2(db='Illum_Retina_BXD_RankInv0410',ProbeSetID='ILMN_2991545',CellID='',db2='Illum_Retina_BXD_RankInv0410',ProbeSetID2='ILMN_2677092',CellID2='',rank='0')) | [**0.598 79**](javascript:showCorrelationPlot2(db='Illum_Retina_BXD_RankInv0410',ProbeSetID='ILMN_2991545',CellID='',db2='Illum_Retina_BXD_RankInv0410',ProbeSetID2='ILMN_2624938',CellID2='',rank='0')) | [**0.578 79**](javascript:showCorrelationPlot2(db='Illum_Retina_BXD_RankInv0410',ProbeSetID='ILMN_2991545',CellID='',db2='Illum_Retina_BXD_RankInv0410',ProbeSetID2='ILMN_2847269',CellID2='',rank='0')) | [**0.603 79**](javascript:showCorrelationPlot2(db='Illum_Retina_BXD_RankInv0410',ProbeSetID='ILMN_2991545',CellID='',db2='Illum_Retina_BXD_RankInv0410',ProbeSetID2='ILMN_2839682',CellID2='',rank='0')) | [**0.537 79**](javascript:showCorrelationPlot2(db='Illum_Retina_BXD_RankInv0410',ProbeSetID='ILMN_2991545',CellID='',db2='Illum_Retina_BXD_RankInv0410',ProbeSetID2='ILMN_3147135',CellID2='',rank='0')) | [**0.574 79**](javascript:showCorrelationPlot2(db='Illum_Retina_BXD_RankInv0410',ProbeSetID='ILMN_2991545',CellID='',db2='Illum_Retina_BXD_RankInv0410',ProbeSetID2='ILMN_1255823',CellID2='',rank='0')) | [**0.723 79**](javascript:showCorrelationPlot2(db='Illum_Retina_BXD_RankInv0410',ProbeSetID='ILMN_2991545',CellID='',db2='Illum_Retina_BXD_RankInv0410',ProbeSetID2='ILMN_2626143',CellID2='',rank='0')) | [**0.643 79**](javascript:showCorrelationPlot2(db='Illum_Retina_BXD_RankInv0410',ProbeSetID='ILMN_2991545',CellID='',db2='Illum_Retina_BXD_RankInv0410',ProbeSetID2='ILMN_1234930',CellID2='',rank='0')) | [**0.599 79**](javascript:showCorrelationPlot2(db='Illum_Retina_BXD_RankInv0410',ProbeSetID='ILMN_2991545',CellID='',db2='Illum_Retina_BXD_RankInv0410',ProbeSetID2='ILMN_1249408',CellID2='',rank='0')) | [**0.508 79**](javascript:showCorrelationPlot2(db='Illum_Retina_BXD_RankInv0410',ProbeSetID='ILMN_2991545',CellID='',db2='Illum_Retina_BXD_RankInv0410',ProbeSetID2='ILMN_2971559',CellID2='',rank='0')) | [**0.456 79**](javascript:showCorrelationPlot2(db='Illum_Retina_BXD_RankInv0410',ProbeSetID='ILMN_2991545',CellID='',db2='Illum_Retina_BXD_RankInv0410',ProbeSetID2='ILMN_1258455',CellID2='',rank='0')) | [**0.672 79**](javascript:showCorrelationPlot2(db='Illum_Retina_BXD_RankInv0410',ProbeSetID='ILMN_2991545',CellID='',db2='Illum_Retina_BXD_RankInv0410',ProbeSetID2='ILMN_2691613',CellID2='',rank='0')) | [**0.565 79**](javascript:showCorrelationPlot2(db='Illum_Retina_BXD_RankInv0410',ProbeSetID='ILMN_2991545',CellID='',db2='Illum_Retina_BXD_RankInv0410',ProbeSetID2='ILMN_1253600',CellID2='',rank='0')) | [***n* 79**](javascript:showDatabase2('Illum_Retina_BXD_RankInv0410','ILMN_2991545','')) | [**0.655 79**](javascript:showCorrelationPlot2(db='Illum_Retina_BXD_RankInv0410',ProbeSetID='ILMN_2991545',CellID='',db2='Illum_Retina_BXD_RankInv0410',ProbeSetID2='ILMN_2623591',CellID2='',rank='1')) | [**0.432 79**](javascript:showCorrelationPlot2(db='Illum_Retina_BXD_RankInv0410',ProbeSetID='ILMN_2991545',CellID='',db2='Illum_Retina_BXD_RankInv0410',ProbeSetID2='ILMN_2603568',CellID2='',rank='1')) | [**0.584 79**](javascript:showCorrelationPlot2(db='Illum_Retina_BXD_RankInv0410',ProbeSetID='ILMN_2991545',CellID='',db2='Illum_Retina_BXD_RankInv0410',ProbeSetID2='ILMN_2992541',CellID2='',rank='1')) | [**0.495 79**](javascript:showCorrelationPlot2(db='Illum_Retina_BXD_RankInv0410',ProbeSetID='ILMN_2991545',CellID='',db2='Illum_Retina_BXD_RankInv0410',ProbeSetID2='ILMN_2792868',CellID2='',rank='1')) | [**0.522 79**](javascript:showCorrelationPlot2(db='Illum_Retina_BXD_RankInv0410',ProbeSetID='ILMN_2991545',CellID='',db2='Illum_Retina_BXD_RankInv0410',ProbeSetID2='ILMN_3129497',CellID2='',rank='1')) | [**0.453 79**](javascript:showCorrelationPlot2(db='Illum_Retina_BXD_RankInv0410',ProbeSetID='ILMN_2991545',CellID='',db2='Illum_Retina_BXD_RankInv0410',ProbeSetID2='ILMN_1224034',CellID2='',rank='1')) | [**0.489 79**](javascript:showCorrelationPlot2(db='Illum_Retina_BXD_RankInv0410',ProbeSetID='ILMN_2991545',CellID='',db2='Illum_Retina_BXD_RankInv0410',ProbeSetID2='ILMN_2639819',CellID2='',rank='1')) | [**0.549 79**](javascript:showCorrelationPlot2(db='Illum_Retina_BXD_RankInv0410',ProbeSetID='ILMN_2991545',CellID='',db2='Illum_Retina_BXD_RankInv0410',ProbeSetID2='ILMN_2994779',CellID2='',rank='1')) | [**0.435 79**](javascript:showCorrelationPlot2(db='Illum_Retina_BXD_RankInv0410',ProbeSetID='ILMN_2991545',CellID='',db2='Illum_Retina_BXD_RankInv0410',ProbeSetID2='ILMN_2625047',CellID2='',rank='1')) | [**0.427 79**](javascript:showCorrelationPlot2(db='Illum_Retina_BXD_RankInv0410',ProbeSetID='ILMN_2991545',CellID='',db2='Illum_Retina_BXD_RankInv0410',ProbeSetID2='ILMN_2808186',CellID2='',rank='1')) | [**0.488 79**](javascript:showCorrelationPlot2(db='Illum_Retina_BXD_RankInv0410',ProbeSetID='ILMN_2991545',CellID='',db2='Illum_Retina_BXD_RankInv0410',ProbeSetID2='ILMN_2689056',CellID2='',rank='1')) | [**0.533 79**](javascript:showCorrelationPlot2(db='Illum_Retina_BXD_RankInv0410',ProbeSetID='ILMN_2991545',CellID='',db2='Illum_Retina_BXD_RankInv0410',ProbeSetID2='ILMN_2648386',CellID2='',rank='1')) | [**0.536 79**](javascript:showCorrelationPlot2(db='Illum_Retina_BXD_RankInv0410',ProbeSetID='ILMN_2991545',CellID='',db2='Illum_Retina_BXD_RankInv0410',ProbeSetID2='ILMN_2869225',CellID2='',rank='1')) | [**0.516 79**](javascript:showCorrelationPlot2(db='Illum_Retina_BXD_RankInv0410',ProbeSetID='ILMN_2991545',CellID='',db2='Illum_Retina_BXD_RankInv0410',ProbeSetID2='ILMN_2728431',CellID2='',rank='1')) | [**0.472 79**](javascript:showCorrelationPlot2(db='Illum_Retina_BXD_RankInv0410',ProbeSetID='ILMN_2991545',CellID='',db2='Illum_Retina_BXD_RankInv0410',ProbeSetID2='ILMN_3094608',CellID2='',rank='1')) | [**0.354 79**](javascript:showCorrelationPlot2(db='Illum_Retina_BXD_RankInv0410',ProbeSetID='ILMN_2991545',CellID='',db2='Illum_Retina_BXD_RankInv0410',ProbeSetID2='ILMN_2896528',CellID2='',rank='1')) | [**0.392 79**](javascript:showCorrelationPlot2(db='Illum_Retina_BXD_RankInv0410',ProbeSetID='ILMN_2991545',CellID='',db2='Illum_Retina_BXD_RankInv0410',ProbeSetID2='ILMN_2701750',CellID2='',rank='1')) | [**0.489 79**](javascript:showCorrelationPlot2(db='Illum_Retina_BXD_RankInv0410',ProbeSetID='ILMN_2991545',CellID='',db2='Illum_Retina_BXD_RankInv0410',ProbeSetID2='ILMN_1239448',CellID2='',rank='1')) | [**-0.387 79**](javascript:showCorrelationPlot2(db='Illum_Retina_BXD_RankInv0410',ProbeSetID='ILMN_2991545',CellID='',db2='Illum_Retina_BXD_RankInv0410',ProbeSetID2='ILMN_2583882',CellID2='',rank='1')) | [**0.468 79**](javascript:showCorrelationPlot2(db='Illum_Retina_BXD_RankInv0410',ProbeSetID='ILMN_2991545',CellID='',db2='Illum_Retina_BXD_RankInv0410',ProbeSetID2='ILMN_2915166',CellID2='',rank='1')) | [**0.475 79**](javascript:showCorrelationPlot2(db='Illum_Retina_BXD_RankInv0410',ProbeSetID='ILMN_2991545',CellID='',db2='Illum_Retina_BXD_RankInv0410',ProbeSetID2='ILMN_2702997',CellID2='',rank='1')) | [**0.482 79**](javascript:showCorrelationPlot2(db='Illum_Retina_BXD_RankInv0410',ProbeSetID='ILMN_2991545',CellID='',db2='Illum_Retina_BXD_RankInv0410',ProbeSetID2='ILMN_2435206',CellID2='',rank='1')) | [**0.470 79**](javascript:showCorrelationPlot2(db='Illum_Retina_BXD_RankInv0410',ProbeSetID='ILMN_2991545',CellID='',db2='Illum_Retina_BXD_RankInv0410',ProbeSetID2='ILMN_2789888',CellID2='',rank='1')) | [**0.443 79**](javascript:showCorrelationPlot2(db='Illum_Retina_BXD_RankInv0410',ProbeSetID='ILMN_2991545',CellID='',db2='Illum_Retina_BXD_RankInv0410',ProbeSetID2='ILMN_1218901',CellID2='',rank='1')) | [**0.459 79**](javascript:showCorrelationPlot2(db='Illum_Retina_BXD_RankInv0410',ProbeSetID='ILMN_2991545',CellID='',db2='Illum_Retina_BXD_RankInv0410',ProbeSetID2='ILMN_1254734',CellID2='',rank='1')) | [**0.460 79**](javascript:showCorrelationPlot2(db='Illum_Retina_BXD_RankInv0410',ProbeSetID='ILMN_2991545',CellID='',db2='Illum_Retina_BXD_RankInv0410',ProbeSetID2='ILMN_3008924',CellID2='',rank='1')) | [**0.492 79**](javascript:showCorrelationPlot2(db='Illum_Retina_BXD_RankInv0410',ProbeSetID='ILMN_2991545',CellID='',db2='Illum_Retina_BXD_RankInv0410',ProbeSetID2='ILMN_2628175',CellID2='',rank='1')) | [**0.513 79**](javascript:showCorrelationPlot2(db='Illum_Retina_BXD_RankInv0410',ProbeSetID='ILMN_2991545',CellID='',db2='Illum_Retina_BXD_RankInv0410',ProbeSetID2='ILMN_2891573',CellID2='',rank='1')) | [**0.482 79**](javascript:showCorrelationPlot2(db='Illum_Retina_BXD_RankInv0410',ProbeSetID='ILMN_2991545',CellID='',db2='Illum_Retina_BXD_RankInv0410',ProbeSetID2='ILMN_1220846',CellID2='',rank='1')) | [**0.528 79**](javascript:showCorrelationPlot2(db='Illum_Retina_BXD_RankInv0410',ProbeSetID='ILMN_2991545',CellID='',db2='Illum_Retina_BXD_RankInv0410',ProbeSetID2='ILMN_1241962',CellID2='',rank='1')) | [**0.486 79**](javascript:showCorrelationPlot2(db='Illum_Retina_BXD_RankInv0410',ProbeSetID='ILMN_2991545',CellID='',db2='Illum_Retina_BXD_RankInv0410',ProbeSetID2='ILMN_3163027',CellID2='',rank='1')) | [**0.372 79**](javascript:showCorrelationPlot2(db='Illum_Retina_BXD_RankInv0410',ProbeSetID='ILMN_2991545',CellID='',db2='Illum_Retina_BXD_RankInv0410',ProbeSetID2='ILMN_2775098',CellID2='',rank='1')) | [**0.626 79**](javascript:showCorrelationPlot2(db='Illum_Retina_BXD_RankInv0410',ProbeSetID='ILMN_2991545',CellID='',db2='Illum_Retina_BXD_RankInv0410',ProbeSetID2='ILMN_2752552',CellID2='',rank='1')) | [**0.514 79**](javascript:showCorrelationPlot2(db='Illum_Retina_BXD_RankInv0410',ProbeSetID='ILMN_2991545',CellID='',db2='Illum_Retina_BXD_RankInv0410',ProbeSetID2='ILMN_2730293',CellID2='',rank='1')) | [**0.364 79**](javascript:showCorrelationPlot2(db='Illum_Retina_BXD_RankInv0410',ProbeSetID='ILMN_2991545',CellID='',db2='Illum_Retina_BXD_RankInv0410',ProbeSetID2='ILMN_2680872',CellID2='',rank='1')) | [**0.238 79**](javascript:showCorrelationPlot2(db='Illum_Retina_BXD_RankInv0410',ProbeSetID='ILMN_2991545',CellID='',db2='Illum_Retina_BXD_RankInv0410',ProbeSetID2='ILMN_2768533',CellID2='',rank='1')) | [**-0.147 79**](javascript:showCorrelationPlot2(db='Illum_Retina_BXD_RankInv0410',ProbeSetID='ILMN_2991545',CellID='',db2='Illum_Retina_BXD_RankInv0410',ProbeSetID2='ILMN_3121255',CellID2='',rank='1')) | [**-0.316 79**](javascript:showCorrelationPlot2(db='Illum_Retina_BXD_RankInv0410',ProbeSetID='ILMN_2991545',CellID='',db2='Illum_Retina_BXD_RankInv0410',ProbeSetID2='ILMN_2484527',CellID2='',rank='1')) | [**0.204 79**](javascript:showCorrelationPlot2(db='Illum_Retina_BXD_RankInv0410',ProbeSetID='ILMN_2991545',CellID='',db2='Illum_Retina_BXD_RankInv0410',ProbeSetID2='ILMN_3045723',CellID2='',rank='1')) | [**0.084 79**](javascript:showCorrelationPlot2(db='Illum_Retina_BXD_RankInv0410',ProbeSetID='ILMN_2991545',CellID='',db2='Illum_Retina_BXD_RankInv0410',ProbeSetID2='ILMN_2486573',CellID2='',rank='1')) |
|  | [**Trait 17: ILMN_2623591**](javascript:showDatabase2('Illum_Retina_BXD_RankInv0410','ILMN_2623591','');)  Symbol: Apbb1  Apbb1 | [**0.666 79**](javascript:showCorrelationPlot2(db='Illum_Retina_BXD_RankInv0410',ProbeSetID='ILMN_2623591',CellID='',db2='Illum_Retina_BXD_RankInv0410',ProbeSetID2='ILMN_2450384',CellID2='',rank='0')) | [**0.755 79**](javascript:showCorrelationPlot2(db='Illum_Retina_BXD_RankInv0410',ProbeSetID='ILMN_2623591',CellID='',db2='Illum_Retina_BXD_RankInv0410',ProbeSetID2='ILMN_3068754',CellID2='',rank='0')) | [**0.660 79**](javascript:showCorrelationPlot2(db='Illum_Retina_BXD_RankInv0410',ProbeSetID='ILMN_2623591',CellID='',db2='Illum_Retina_BXD_RankInv0410',ProbeSetID2='ILMN_2677092',CellID2='',rank='0')) | [**0.606 79**](javascript:showCorrelationPlot2(db='Illum_Retina_BXD_RankInv0410',ProbeSetID='ILMN_2623591',CellID='',db2='Illum_Retina_BXD_RankInv0410',ProbeSetID2='ILMN_2624938',CellID2='',rank='0')) | [**0.455 79**](javascript:showCorrelationPlot2(db='Illum_Retina_BXD_RankInv0410',ProbeSetID='ILMN_2623591',CellID='',db2='Illum_Retina_BXD_RankInv0410',ProbeSetID2='ILMN_2847269',CellID2='',rank='0')) | [**0.641 79**](javascript:showCorrelationPlot2(db='Illum_Retina_BXD_RankInv0410',ProbeSetID='ILMN_2623591',CellID='',db2='Illum_Retina_BXD_RankInv0410',ProbeSetID2='ILMN_2839682',CellID2='',rank='0')) | [**0.417 79**](javascript:showCorrelationPlot2(db='Illum_Retina_BXD_RankInv0410',ProbeSetID='ILMN_2623591',CellID='',db2='Illum_Retina_BXD_RankInv0410',ProbeSetID2='ILMN_3147135',CellID2='',rank='0')) | [**0.439 79**](javascript:showCorrelationPlot2(db='Illum_Retina_BXD_RankInv0410',ProbeSetID='ILMN_2623591',CellID='',db2='Illum_Retina_BXD_RankInv0410',ProbeSetID2='ILMN_1255823',CellID2='',rank='0')) | [**0.603 79**](javascript:showCorrelationPlot2(db='Illum_Retina_BXD_RankInv0410',ProbeSetID='ILMN_2623591',CellID='',db2='Illum_Retina_BXD_RankInv0410',ProbeSetID2='ILMN_2626143',CellID2='',rank='0')) | [**0.636 79**](javascript:showCorrelationPlot2(db='Illum_Retina_BXD_RankInv0410',ProbeSetID='ILMN_2623591',CellID='',db2='Illum_Retina_BXD_RankInv0410',ProbeSetID2='ILMN_1234930',CellID2='',rank='0')) | [**0.510 79**](javascript:showCorrelationPlot2(db='Illum_Retina_BXD_RankInv0410',ProbeSetID='ILMN_2623591',CellID='',db2='Illum_Retina_BXD_RankInv0410',ProbeSetID2='ILMN_1249408',CellID2='',rank='0')) | [**0.570 79**](javascript:showCorrelationPlot2(db='Illum_Retina_BXD_RankInv0410',ProbeSetID='ILMN_2623591',CellID='',db2='Illum_Retina_BXD_RankInv0410',ProbeSetID2='ILMN_2971559',CellID2='',rank='0')) | [**0.539 79**](javascript:showCorrelationPlot2(db='Illum_Retina_BXD_RankInv0410',ProbeSetID='ILMN_2623591',CellID='',db2='Illum_Retina_BXD_RankInv0410',ProbeSetID2='ILMN_1258455',CellID2='',rank='0')) | [**0.493 79**](javascript:showCorrelationPlot2(db='Illum_Retina_BXD_RankInv0410',ProbeSetID='ILMN_2623591',CellID='',db2='Illum_Retina_BXD_RankInv0410',ProbeSetID2='ILMN_2691613',CellID2='',rank='0')) | [**0.761 79**](javascript:showCorrelationPlot2(db='Illum_Retina_BXD_RankInv0410',ProbeSetID='ILMN_2623591',CellID='',db2='Illum_Retina_BXD_RankInv0410',ProbeSetID2='ILMN_1253600',CellID2='',rank='0')) | [**0.656 79**](javascript:showCorrelationPlot2(db='Illum_Retina_BXD_RankInv0410',ProbeSetID='ILMN_2623591',CellID='',db2='Illum_Retina_BXD_RankInv0410',ProbeSetID2='ILMN_2991545',CellID2='',rank='0')) | [***n* 79**](javascript:showDatabase2('Illum_Retina_BXD_RankInv0410','ILMN_2623591','')) | [**0.455 79**](javascript:showCorrelationPlot2(db='Illum_Retina_BXD_RankInv0410',ProbeSetID='ILMN_2623591',CellID='',db2='Illum_Retina_BXD_RankInv0410',ProbeSetID2='ILMN_2603568',CellID2='',rank='1')) | [**0.505 79**](javascript:showCorrelationPlot2(db='Illum_Retina_BXD_RankInv0410',ProbeSetID='ILMN_2623591',CellID='',db2='Illum_Retina_BXD_RankInv0410',ProbeSetID2='ILMN_2992541',CellID2='',rank='1')) | [**0.334 79**](javascript:showCorrelationPlot2(db='Illum_Retina_BXD_RankInv0410',ProbeSetID='ILMN_2623591',CellID='',db2='Illum_Retina_BXD_RankInv0410',ProbeSetID2='ILMN_2792868',CellID2='',rank='1')) | [**0.686 79**](javascript:showCorrelationPlot2(db='Illum_Retina_BXD_RankInv0410',ProbeSetID='ILMN_2623591',CellID='',db2='Illum_Retina_BXD_RankInv0410',ProbeSetID2='ILMN_3129497',CellID2='',rank='1')) | [**0.572 79**](javascript:showCorrelationPlot2(db='Illum_Retina_BXD_RankInv0410',ProbeSetID='ILMN_2623591',CellID='',db2='Illum_Retina_BXD_RankInv0410',ProbeSetID2='ILMN_1224034',CellID2='',rank='1')) | [**0.518 79**](javascript:showCorrelationPlot2(db='Illum_Retina_BXD_RankInv0410',ProbeSetID='ILMN_2623591',CellID='',db2='Illum_Retina_BXD_RankInv0410',ProbeSetID2='ILMN_2639819',CellID2='',rank='1')) | [**0.417 79**](javascript:showCorrelationPlot2(db='Illum_Retina_BXD_RankInv0410',ProbeSetID='ILMN_2623591',CellID='',db2='Illum_Retina_BXD_RankInv0410',ProbeSetID2='ILMN_2994779',CellID2='',rank='1')) | [**0.512 79**](javascript:showCorrelationPlot2(db='Illum_Retina_BXD_RankInv0410',ProbeSetID='ILMN_2623591',CellID='',db2='Illum_Retina_BXD_RankInv0410',ProbeSetID2='ILMN_2625047',CellID2='',rank='1')) | [**0.610 79**](javascript:showCorrelationPlot2(db='Illum_Retina_BXD_RankInv0410',ProbeSetID='ILMN_2623591',CellID='',db2='Illum_Retina_BXD_RankInv0410',ProbeSetID2='ILMN_2808186',CellID2='',rank='1')) | [**0.588 79**](javascript:showCorrelationPlot2(db='Illum_Retina_BXD_RankInv0410',ProbeSetID='ILMN_2623591',CellID='',db2='Illum_Retina_BXD_RankInv0410',ProbeSetID2='ILMN_2689056',CellID2='',rank='1')) | [**0.492 79**](javascript:showCorrelationPlot2(db='Illum_Retina_BXD_RankInv0410',ProbeSetID='ILMN_2623591',CellID='',db2='Illum_Retina_BXD_RankInv0410',ProbeSetID2='ILMN_2648386',CellID2='',rank='1')) | [**0.509 79**](javascript:showCorrelationPlot2(db='Illum_Retina_BXD_RankInv0410',ProbeSetID='ILMN_2623591',CellID='',db2='Illum_Retina_BXD_RankInv0410',ProbeSetID2='ILMN_2869225',CellID2='',rank='1')) | [**0.580 79**](javascript:showCorrelationPlot2(db='Illum_Retina_BXD_RankInv0410',ProbeSetID='ILMN_2623591',CellID='',db2='Illum_Retina_BXD_RankInv0410',ProbeSetID2='ILMN_2728431',CellID2='',rank='1')) | [**0.588 79**](javascript:showCorrelationPlot2(db='Illum_Retina_BXD_RankInv0410',ProbeSetID='ILMN_2623591',CellID='',db2='Illum_Retina_BXD_RankInv0410',ProbeSetID2='ILMN_3094608',CellID2='',rank='1')) | [**0.313 79**](javascript:showCorrelationPlot2(db='Illum_Retina_BXD_RankInv0410',ProbeSetID='ILMN_2623591',CellID='',db2='Illum_Retina_BXD_RankInv0410',ProbeSetID2='ILMN_2896528',CellID2='',rank='1')) | [**0.311 79**](javascript:showCorrelationPlot2(db='Illum_Retina_BXD_RankInv0410',ProbeSetID='ILMN_2623591',CellID='',db2='Illum_Retina_BXD_RankInv0410',ProbeSetID2='ILMN_2701750',CellID2='',rank='1')) | [**0.552 79**](javascript:showCorrelationPlot2(db='Illum_Retina_BXD_RankInv0410',ProbeSetID='ILMN_2623591',CellID='',db2='Illum_Retina_BXD_RankInv0410',ProbeSetID2='ILMN_1239448',CellID2='',rank='1')) | [**-0.324 79**](javascript:showCorrelationPlot2(db='Illum_Retina_BXD_RankInv0410',ProbeSetID='ILMN_2623591',CellID='',db2='Illum_Retina_BXD_RankInv0410',ProbeSetID2='ILMN_2583882',CellID2='',rank='1')) | [**0.405 79**](javascript:showCorrelationPlot2(db='Illum_Retina_BXD_RankInv0410',ProbeSetID='ILMN_2623591',CellID='',db2='Illum_Retina_BXD_RankInv0410',ProbeSetID2='ILMN_2915166',CellID2='',rank='1')) | [**0.543 79**](javascript:showCorrelationPlot2(db='Illum_Retina_BXD_RankInv0410',ProbeSetID='ILMN_2623591',CellID='',db2='Illum_Retina_BXD_RankInv0410',ProbeSetID2='ILMN_2702997',CellID2='',rank='1')) | [**0.555 79**](javascript:showCorrelationPlot2(db='Illum_Retina_BXD_RankInv0410',ProbeSetID='ILMN_2623591',CellID='',db2='Illum_Retina_BXD_RankInv0410',ProbeSetID2='ILMN_2435206',CellID2='',rank='1')) | [**0.754 79**](javascript:showCorrelationPlot2(db='Illum_Retina_BXD_RankInv0410',ProbeSetID='ILMN_2623591',CellID='',db2='Illum_Retina_BXD_RankInv0410',ProbeSetID2='ILMN_2789888',CellID2='',rank='1')) | [**0.602 79**](javascript:showCorrelationPlot2(db='Illum_Retina_BXD_RankInv0410',ProbeSetID='ILMN_2623591',CellID='',db2='Illum_Retina_BXD_RankInv0410',ProbeSetID2='ILMN_1218901',CellID2='',rank='1')) | [**0.472 79**](javascript:showCorrelationPlot2(db='Illum_Retina_BXD_RankInv0410',ProbeSetID='ILMN_2623591',CellID='',db2='Illum_Retina_BXD_RankInv0410',ProbeSetID2='ILMN_1254734',CellID2='',rank='1')) | [**0.658 79**](javascript:showCorrelationPlot2(db='Illum_Retina_BXD_RankInv0410',ProbeSetID='ILMN_2623591',CellID='',db2='Illum_Retina_BXD_RankInv0410',ProbeSetID2='ILMN_3008924',CellID2='',rank='1')) | [**0.685 79**](javascript:showCorrelationPlot2(db='Illum_Retina_BXD_RankInv0410',ProbeSetID='ILMN_2623591',CellID='',db2='Illum_Retina_BXD_RankInv0410',ProbeSetID2='ILMN_2628175',CellID2='',rank='1')) | [**0.535 79**](javascript:showCorrelationPlot2(db='Illum_Retina_BXD_RankInv0410',ProbeSetID='ILMN_2623591',CellID='',db2='Illum_Retina_BXD_RankInv0410',ProbeSetID2='ILMN_2891573',CellID2='',rank='1')) | [**0.556 79**](javascript:showCorrelationPlot2(db='Illum_Retina_BXD_RankInv0410',ProbeSetID='ILMN_2623591',CellID='',db2='Illum_Retina_BXD_RankInv0410',ProbeSetID2='ILMN_1220846',CellID2='',rank='1')) | [**0.447 79**](javascript:showCorrelationPlot2(db='Illum_Retina_BXD_RankInv0410',ProbeSetID='ILMN_2623591',CellID='',db2='Illum_Retina_BXD_RankInv0410',ProbeSetID2='ILMN_1241962',CellID2='',rank='1')) | [**0.507 79**](javascript:showCorrelationPlot2(db='Illum_Retina_BXD_RankInv0410',ProbeSetID='ILMN_2623591',CellID='',db2='Illum_Retina_BXD_RankInv0410',ProbeSetID2='ILMN_3163027',CellID2='',rank='1')) | [**0.242 79**](javascript:showCorrelationPlot2(db='Illum_Retina_BXD_RankInv0410',ProbeSetID='ILMN_2623591',CellID='',db2='Illum_Retina_BXD_RankInv0410',ProbeSetID2='ILMN_2775098',CellID2='',rank='1')) | [**0.567 79**](javascript:showCorrelationPlot2(db='Illum_Retina_BXD_RankInv0410',ProbeSetID='ILMN_2623591',CellID='',db2='Illum_Retina_BXD_RankInv0410',ProbeSetID2='ILMN_2752552',CellID2='',rank='1')) | [**0.603 79**](javascript:showCorrelationPlot2(db='Illum_Retina_BXD_RankInv0410',ProbeSetID='ILMN_2623591',CellID='',db2='Illum_Retina_BXD_RankInv0410',ProbeSetID2='ILMN_2730293',CellID2='',rank='1')) | [**0.448 79**](javascript:showCorrelationPlot2(db='Illum_Retina_BXD_RankInv0410',ProbeSetID='ILMN_2623591',CellID='',db2='Illum_Retina_BXD_RankInv0410',ProbeSetID2='ILMN_2680872',CellID2='',rank='1')) | [**0.026 79**](javascript:showCorrelationPlot2(db='Illum_Retina_BXD_RankInv0410',ProbeSetID='ILMN_2623591',CellID='',db2='Illum_Retina_BXD_RankInv0410',ProbeSetID2='ILMN_2768533',CellID2='',rank='1')) | [**-0.159 79**](javascript:showCorrelationPlot2(db='Illum_Retina_BXD_RankInv0410',ProbeSetID='ILMN_2623591',CellID='',db2='Illum_Retina_BXD_RankInv0410',ProbeSetID2='ILMN_3121255',CellID2='',rank='1')) | [**-0.271 79**](javascript:showCorrelationPlot2(db='Illum_Retina_BXD_RankInv0410',ProbeSetID='ILMN_2623591',CellID='',db2='Illum_Retina_BXD_RankInv0410',ProbeSetID2='ILMN_2484527',CellID2='',rank='1')) | [**0.090 79**](javascript:showCorrelationPlot2(db='Illum_Retina_BXD_RankInv0410',ProbeSetID='ILMN_2623591',CellID='',db2='Illum_Retina_BXD_RankInv0410',ProbeSetID2='ILMN_3045723',CellID2='',rank='1')) | [**0.197 79**](javascript:showCorrelationPlot2(db='Illum_Retina_BXD_RankInv0410',ProbeSetID='ILMN_2623591',CellID='',db2='Illum_Retina_BXD_RankInv0410',ProbeSetID2='ILMN_2486573',CellID2='',rank='1')) |
|  | [**Trait 18: ILMN_2603568**](javascript:showDatabase2('Illum_Retina_BXD_RankInv0410','ILMN_2603568','');)  Symbol: Leprot  Leprot | [**0.665 79**](javascript:showCorrelationPlot2(db='Illum_Retina_BXD_RankInv0410',ProbeSetID='ILMN_2603568',CellID='',db2='Illum_Retina_BXD_RankInv0410',ProbeSetID2='ILMN_2450384',CellID2='',rank='0')) | [**0.627 79**](javascript:showCorrelationPlot2(db='Illum_Retina_BXD_RankInv0410',ProbeSetID='ILMN_2603568',CellID='',db2='Illum_Retina_BXD_RankInv0410',ProbeSetID2='ILMN_3068754',CellID2='',rank='0')) | [**0.669 79**](javascript:showCorrelationPlot2(db='Illum_Retina_BXD_RankInv0410',ProbeSetID='ILMN_2603568',CellID='',db2='Illum_Retina_BXD_RankInv0410',ProbeSetID2='ILMN_2677092',CellID2='',rank='0')) | [**0.452 79**](javascript:showCorrelationPlot2(db='Illum_Retina_BXD_RankInv0410',ProbeSetID='ILMN_2603568',CellID='',db2='Illum_Retina_BXD_RankInv0410',ProbeSetID2='ILMN_2624938',CellID2='',rank='0')) | [**0.463 79**](javascript:showCorrelationPlot2(db='Illum_Retina_BXD_RankInv0410',ProbeSetID='ILMN_2603568',CellID='',db2='Illum_Retina_BXD_RankInv0410',ProbeSetID2='ILMN_2847269',CellID2='',rank='0')) | [**0.451 79**](javascript:showCorrelationPlot2(db='Illum_Retina_BXD_RankInv0410',ProbeSetID='ILMN_2603568',CellID='',db2='Illum_Retina_BXD_RankInv0410',ProbeSetID2='ILMN_2839682',CellID2='',rank='0')) | [**0.601 79**](javascript:showCorrelationPlot2(db='Illum_Retina_BXD_RankInv0410',ProbeSetID='ILMN_2603568',CellID='',db2='Illum_Retina_BXD_RankInv0410',ProbeSetID2='ILMN_3147135',CellID2='',rank='0')) | [**0.660 79**](javascript:showCorrelationPlot2(db='Illum_Retina_BXD_RankInv0410',ProbeSetID='ILMN_2603568',CellID='',db2='Illum_Retina_BXD_RankInv0410',ProbeSetID2='ILMN_1255823',CellID2='',rank='0')) | [**0.471 79**](javascript:showCorrelationPlot2(db='Illum_Retina_BXD_RankInv0410',ProbeSetID='ILMN_2603568',CellID='',db2='Illum_Retina_BXD_RankInv0410',ProbeSetID2='ILMN_2626143',CellID2='',rank='0')) | [**0.414 79**](javascript:showCorrelationPlot2(db='Illum_Retina_BXD_RankInv0410',ProbeSetID='ILMN_2603568',CellID='',db2='Illum_Retina_BXD_RankInv0410',ProbeSetID2='ILMN_1234930',CellID2='',rank='0')) | [**0.640 79**](javascript:showCorrelationPlot2(db='Illum_Retina_BXD_RankInv0410',ProbeSetID='ILMN_2603568',CellID='',db2='Illum_Retina_BXD_RankInv0410',ProbeSetID2='ILMN_1249408',CellID2='',rank='0')) | [**0.228 79**](javascript:showCorrelationPlot2(db='Illum_Retina_BXD_RankInv0410',ProbeSetID='ILMN_2603568',CellID='',db2='Illum_Retina_BXD_RankInv0410',ProbeSetID2='ILMN_2971559',CellID2='',rank='0')) | [**0.408 79**](javascript:showCorrelationPlot2(db='Illum_Retina_BXD_RankInv0410',ProbeSetID='ILMN_2603568',CellID='',db2='Illum_Retina_BXD_RankInv0410',ProbeSetID2='ILMN_1258455',CellID2='',rank='0')) | [**0.520 79**](javascript:showCorrelationPlot2(db='Illum_Retina_BXD_RankInv0410',ProbeSetID='ILMN_2603568',CellID='',db2='Illum_Retina_BXD_RankInv0410',ProbeSetID2='ILMN_2691613',CellID2='',rank='0')) | [**0.507 79**](javascript:showCorrelationPlot2(db='Illum_Retina_BXD_RankInv0410',ProbeSetID='ILMN_2603568',CellID='',db2='Illum_Retina_BXD_RankInv0410',ProbeSetID2='ILMN_1253600',CellID2='',rank='0')) | [**0.460 79**](javascript:showCorrelationPlot2(db='Illum_Retina_BXD_RankInv0410',ProbeSetID='ILMN_2603568',CellID='',db2='Illum_Retina_BXD_RankInv0410',ProbeSetID2='ILMN_2991545',CellID2='',rank='0')) | [**0.400 79**](javascript:showCorrelationPlot2(db='Illum_Retina_BXD_RankInv0410',ProbeSetID='ILMN_2603568',CellID='',db2='Illum_Retina_BXD_RankInv0410',ProbeSetID2='ILMN_2623591',CellID2='',rank='0')) | [***n* 79**](javascript:showDatabase2('Illum_Retina_BXD_RankInv0410','ILMN_2603568','')) | [**0.404 79**](javascript:showCorrelationPlot2(db='Illum_Retina_BXD_RankInv0410',ProbeSetID='ILMN_2603568',CellID='',db2='Illum_Retina_BXD_RankInv0410',ProbeSetID2='ILMN_2992541',CellID2='',rank='1')) | [**0.596 79**](javascript:showCorrelationPlot2(db='Illum_Retina_BXD_RankInv0410',ProbeSetID='ILMN_2603568',CellID='',db2='Illum_Retina_BXD_RankInv0410',ProbeSetID2='ILMN_2792868',CellID2='',rank='1')) | [**0.384 79**](javascript:showCorrelationPlot2(db='Illum_Retina_BXD_RankInv0410',ProbeSetID='ILMN_2603568',CellID='',db2='Illum_Retina_BXD_RankInv0410',ProbeSetID2='ILMN_3129497',CellID2='',rank='1')) | [**0.364 79**](javascript:showCorrelationPlot2(db='Illum_Retina_BXD_RankInv0410',ProbeSetID='ILMN_2603568',CellID='',db2='Illum_Retina_BXD_RankInv0410',ProbeSetID2='ILMN_1224034',CellID2='',rank='1')) | [**0.478 79**](javascript:showCorrelationPlot2(db='Illum_Retina_BXD_RankInv0410',ProbeSetID='ILMN_2603568',CellID='',db2='Illum_Retina_BXD_RankInv0410',ProbeSetID2='ILMN_2639819',CellID2='',rank='1')) | [**0.248 79**](javascript:showCorrelationPlot2(db='Illum_Retina_BXD_RankInv0410',ProbeSetID='ILMN_2603568',CellID='',db2='Illum_Retina_BXD_RankInv0410',ProbeSetID2='ILMN_2994779',CellID2='',rank='1')) | [**0.366 79**](javascript:showCorrelationPlot2(db='Illum_Retina_BXD_RankInv0410',ProbeSetID='ILMN_2603568',CellID='',db2='Illum_Retina_BXD_RankInv0410',ProbeSetID2='ILMN_2625047',CellID2='',rank='1')) | [**0.469 79**](javascript:showCorrelationPlot2(db='Illum_Retina_BXD_RankInv0410',ProbeSetID='ILMN_2603568',CellID='',db2='Illum_Retina_BXD_RankInv0410',ProbeSetID2='ILMN_2808186',CellID2='',rank='1')) | [**0.616 79**](javascript:showCorrelationPlot2(db='Illum_Retina_BXD_RankInv0410',ProbeSetID='ILMN_2603568',CellID='',db2='Illum_Retina_BXD_RankInv0410',ProbeSetID2='ILMN_2689056',CellID2='',rank='1')) | [**0.558 79**](javascript:showCorrelationPlot2(db='Illum_Retina_BXD_RankInv0410',ProbeSetID='ILMN_2603568',CellID='',db2='Illum_Retina_BXD_RankInv0410',ProbeSetID2='ILMN_2648386',CellID2='',rank='1')) | [**0.458 79**](javascript:showCorrelationPlot2(db='Illum_Retina_BXD_RankInv0410',ProbeSetID='ILMN_2603568',CellID='',db2='Illum_Retina_BXD_RankInv0410',ProbeSetID2='ILMN_2869225',CellID2='',rank='1')) | [**0.544 79**](javascript:showCorrelationPlot2(db='Illum_Retina_BXD_RankInv0410',ProbeSetID='ILMN_2603568',CellID='',db2='Illum_Retina_BXD_RankInv0410',ProbeSetID2='ILMN_2728431',CellID2='',rank='1')) | [**0.522 79**](javascript:showCorrelationPlot2(db='Illum_Retina_BXD_RankInv0410',ProbeSetID='ILMN_2603568',CellID='',db2='Illum_Retina_BXD_RankInv0410',ProbeSetID2='ILMN_3094608',CellID2='',rank='1')) | [**0.439 79**](javascript:showCorrelationPlot2(db='Illum_Retina_BXD_RankInv0410',ProbeSetID='ILMN_2603568',CellID='',db2='Illum_Retina_BXD_RankInv0410',ProbeSetID2='ILMN_2896528',CellID2='',rank='1')) | [**0.543 79**](javascript:showCorrelationPlot2(db='Illum_Retina_BXD_RankInv0410',ProbeSetID='ILMN_2603568',CellID='',db2='Illum_Retina_BXD_RankInv0410',ProbeSetID2='ILMN_2701750',CellID2='',rank='1')) | [**0.532 79**](javascript:showCorrelationPlot2(db='Illum_Retina_BXD_RankInv0410',ProbeSetID='ILMN_2603568',CellID='',db2='Illum_Retina_BXD_RankInv0410',ProbeSetID2='ILMN_1239448',CellID2='',rank='1')) | [**-0.648 79**](javascript:showCorrelationPlot2(db='Illum_Retina_BXD_RankInv0410',ProbeSetID='ILMN_2603568',CellID='',db2='Illum_Retina_BXD_RankInv0410',ProbeSetID2='ILMN_2583882',CellID2='',rank='1')) | [**0.352 79**](javascript:showCorrelationPlot2(db='Illum_Retina_BXD_RankInv0410',ProbeSetID='ILMN_2603568',CellID='',db2='Illum_Retina_BXD_RankInv0410',ProbeSetID2='ILMN_2915166',CellID2='',rank='1')) | [**0.460 79**](javascript:showCorrelationPlot2(db='Illum_Retina_BXD_RankInv0410',ProbeSetID='ILMN_2603568',CellID='',db2='Illum_Retina_BXD_RankInv0410',ProbeSetID2='ILMN_2702997',CellID2='',rank='1')) | [**0.475 79**](javascript:showCorrelationPlot2(db='Illum_Retina_BXD_RankInv0410',ProbeSetID='ILMN_2603568',CellID='',db2='Illum_Retina_BXD_RankInv0410',ProbeSetID2='ILMN_2435206',CellID2='',rank='1')) | [**0.572 79**](javascript:showCorrelationPlot2(db='Illum_Retina_BXD_RankInv0410',ProbeSetID='ILMN_2603568',CellID='',db2='Illum_Retina_BXD_RankInv0410',ProbeSetID2='ILMN_2789888',CellID2='',rank='1')) | [**0.431 79**](javascript:showCorrelationPlot2(db='Illum_Retina_BXD_RankInv0410',ProbeSetID='ILMN_2603568',CellID='',db2='Illum_Retina_BXD_RankInv0410',ProbeSetID2='ILMN_1218901',CellID2='',rank='1')) | [**0.688 79**](javascript:showCorrelationPlot2(db='Illum_Retina_BXD_RankInv0410',ProbeSetID='ILMN_2603568',CellID='',db2='Illum_Retina_BXD_RankInv0410',ProbeSetID2='ILMN_1254734',CellID2='',rank='1')) | [**0.414 79**](javascript:showCorrelationPlot2(db='Illum_Retina_BXD_RankInv0410',ProbeSetID='ILMN_2603568',CellID='',db2='Illum_Retina_BXD_RankInv0410',ProbeSetID2='ILMN_3008924',CellID2='',rank='1')) | [**0.604 79**](javascript:showCorrelationPlot2(db='Illum_Retina_BXD_RankInv0410',ProbeSetID='ILMN_2603568',CellID='',db2='Illum_Retina_BXD_RankInv0410',ProbeSetID2='ILMN_2628175',CellID2='',rank='1')) | [**0.467 79**](javascript:showCorrelationPlot2(db='Illum_Retina_BXD_RankInv0410',ProbeSetID='ILMN_2603568',CellID='',db2='Illum_Retina_BXD_RankInv0410',ProbeSetID2='ILMN_2891573',CellID2='',rank='1')) | [**0.537 79**](javascript:showCorrelationPlot2(db='Illum_Retina_BXD_RankInv0410',ProbeSetID='ILMN_2603568',CellID='',db2='Illum_Retina_BXD_RankInv0410',ProbeSetID2='ILMN_1220846',CellID2='',rank='1')) | [**0.543 79**](javascript:showCorrelationPlot2(db='Illum_Retina_BXD_RankInv0410',ProbeSetID='ILMN_2603568',CellID='',db2='Illum_Retina_BXD_RankInv0410',ProbeSetID2='ILMN_1241962',CellID2='',rank='1')) | [**0.280 79**](javascript:showCorrelationPlot2(db='Illum_Retina_BXD_RankInv0410',ProbeSetID='ILMN_2603568',CellID='',db2='Illum_Retina_BXD_RankInv0410',ProbeSetID2='ILMN_3163027',CellID2='',rank='1')) | [**0.710 79**](javascript:showCorrelationPlot2(db='Illum_Retina_BXD_RankInv0410',ProbeSetID='ILMN_2603568',CellID='',db2='Illum_Retina_BXD_RankInv0410',ProbeSetID2='ILMN_2775098',CellID2='',rank='1')) | **[0.470](javascript:showCorrelationPlot2(db='Illum_Retina_BXD_RankInv0410',ProbeSetID='ILMN_2603568',CellID='',db2='Illum_Retina_BXD_RankInv0410',ProbeSetID2='ILMN_2752552',CellID2='',rank='1'))**  **[79](javascript:showCorrelationPlot2(db='Illum_Retina_BXD_RankInv0410',ProbeSetID='ILMN_2603568',CellID='',db2='Illum_Retina_BXD_RankInv0410',ProbeSetID2='ILMN_2752552',CellID2='',rank='1'))** | [**0.324 79**](javascript:showCorrelationPlot2(db='Illum_Retina_BXD_RankInv0410',ProbeSetID='ILMN_2603568',CellID='',db2='Illum_Retina_BXD_RankInv0410',ProbeSetID2='ILMN_2730293',CellID2='',rank='1')) | [**0.284 79**](javascript:showCorrelationPlot2(db='Illum_Retina_BXD_RankInv0410',ProbeSetID='ILMN_2603568',CellID='',db2='Illum_Retina_BXD_RankInv0410',ProbeSetID2='ILMN_2680872',CellID2='',rank='1')) | [**0.264 79**](javascript:showCorrelationPlot2(db='Illum_Retina_BXD_RankInv0410',ProbeSetID='ILMN_2603568',CellID='',db2='Illum_Retina_BXD_RankInv0410',ProbeSetID2='ILMN_2768533',CellID2='',rank='1')) | [**-0.218 79**](javascript:showCorrelationPlot2(db='Illum_Retina_BXD_RankInv0410',ProbeSetID='ILMN_2603568',CellID='',db2='Illum_Retina_BXD_RankInv0410',ProbeSetID2='ILMN_3121255',CellID2='',rank='1')) | [**-0.510 79**](javascript:showCorrelationPlot2(db='Illum_Retina_BXD_RankInv0410',ProbeSetID='ILMN_2603568',CellID='',db2='Illum_Retina_BXD_RankInv0410',ProbeSetID2='ILMN_2484527',CellID2='',rank='1')) | [**0.069 79**](javascript:showCorrelationPlot2(db='Illum_Retina_BXD_RankInv0410',ProbeSetID='ILMN_2603568',CellID='',db2='Illum_Retina_BXD_RankInv0410',ProbeSetID2='ILMN_3045723',CellID2='',rank='1')) | [**0.230 79**](javascript:showCorrelationPlot2(db='Illum_Retina_BXD_RankInv0410',ProbeSetID='ILMN_2603568',CellID='',db2='Illum_Retina_BXD_RankInv0410',ProbeSetID2='ILMN_2486573',CellID2='',rank='1')) |
|  | [**Trait 19: ILMN_2992541**](javascript:showDatabase2('Illum_Retina_BXD_RankInv0410','ILMN_2992541','');)  Symbol: Ergic3  Ergic3 | [**0.664 79**](javascript:showCorrelationPlot2(db='Illum_Retina_BXD_RankInv0410',ProbeSetID='ILMN_2992541',CellID='',db2='Illum_Retina_BXD_RankInv0410',ProbeSetID2='ILMN_2450384',CellID2='',rank='0')) | [**0.597 79**](javascript:showCorrelationPlot2(db='Illum_Retina_BXD_RankInv0410',ProbeSetID='ILMN_2992541',CellID='',db2='Illum_Retina_BXD_RankInv0410',ProbeSetID2='ILMN_3068754',CellID2='',rank='0')) | [**0.605 79**](javascript:showCorrelationPlot2(db='Illum_Retina_BXD_RankInv0410',ProbeSetID='ILMN_2992541',CellID='',db2='Illum_Retina_BXD_RankInv0410',ProbeSetID2='ILMN_2677092',CellID2='',rank='0')) | [**0.676 79**](javascript:showCorrelationPlot2(db='Illum_Retina_BXD_RankInv0410',ProbeSetID='ILMN_2992541',CellID='',db2='Illum_Retina_BXD_RankInv0410',ProbeSetID2='ILMN_2624938',CellID2='',rank='0')) | [**0.765 79**](javascript:showCorrelationPlot2(db='Illum_Retina_BXD_RankInv0410',ProbeSetID='ILMN_2992541',CellID='',db2='Illum_Retina_BXD_RankInv0410',ProbeSetID2='ILMN_2847269',CellID2='',rank='0')) | [**0.551 79**](javascript:showCorrelationPlot2(db='Illum_Retina_BXD_RankInv0410',ProbeSetID='ILMN_2992541',CellID='',db2='Illum_Retina_BXD_RankInv0410',ProbeSetID2='ILMN_2839682',CellID2='',rank='0')) | [**0.766 79**](javascript:showCorrelationPlot2(db='Illum_Retina_BXD_RankInv0410',ProbeSetID='ILMN_2992541',CellID='',db2='Illum_Retina_BXD_RankInv0410',ProbeSetID2='ILMN_3147135',CellID2='',rank='0')) | [**0.680 79**](javascript:showCorrelationPlot2(db='Illum_Retina_BXD_RankInv0410',ProbeSetID='ILMN_2992541',CellID='',db2='Illum_Retina_BXD_RankInv0410',ProbeSetID2='ILMN_1255823',CellID2='',rank='0')) | [**0.609 79**](javascript:showCorrelationPlot2(db='Illum_Retina_BXD_RankInv0410',ProbeSetID='ILMN_2992541',CellID='',db2='Illum_Retina_BXD_RankInv0410',ProbeSetID2='ILMN_2626143',CellID2='',rank='0')) | [**0.502 79**](javascript:showCorrelationPlot2(db='Illum_Retina_BXD_RankInv0410',ProbeSetID='ILMN_2992541',CellID='',db2='Illum_Retina_BXD_RankInv0410',ProbeSetID2='ILMN_1234930',CellID2='',rank='0')) | [**0.644 79**](javascript:showCorrelationPlot2(db='Illum_Retina_BXD_RankInv0410',ProbeSetID='ILMN_2992541',CellID='',db2='Illum_Retina_BXD_RankInv0410',ProbeSetID2='ILMN_1249408',CellID2='',rank='0')) | [**0.535 79**](javascript:showCorrelationPlot2(db='Illum_Retina_BXD_RankInv0410',ProbeSetID='ILMN_2992541',CellID='',db2='Illum_Retina_BXD_RankInv0410',ProbeSetID2='ILMN_2971559',CellID2='',rank='0')) | [**0.298 79**](javascript:showCorrelationPlot2(db='Illum_Retina_BXD_RankInv0410',ProbeSetID='ILMN_2992541',CellID='',db2='Illum_Retina_BXD_RankInv0410',ProbeSetID2='ILMN_1258455',CellID2='',rank='0')) | [**0.812 79**](javascript:showCorrelationPlot2(db='Illum_Retina_BXD_RankInv0410',ProbeSetID='ILMN_2992541',CellID='',db2='Illum_Retina_BXD_RankInv0410',ProbeSetID2='ILMN_2691613',CellID2='',rank='0')) | [**0.451 79**](javascript:showCorrelationPlot2(db='Illum_Retina_BXD_RankInv0410',ProbeSetID='ILMN_2992541',CellID='',db2='Illum_Retina_BXD_RankInv0410',ProbeSetID2='ILMN_1253600',CellID2='',rank='0')) | [**0.611 79**](javascript:showCorrelationPlot2(db='Illum_Retina_BXD_RankInv0410',ProbeSetID='ILMN_2992541',CellID='',db2='Illum_Retina_BXD_RankInv0410',ProbeSetID2='ILMN_2991545',CellID2='',rank='0')) | [**0.514 79**](javascript:showCorrelationPlot2(db='Illum_Retina_BXD_RankInv0410',ProbeSetID='ILMN_2992541',CellID='',db2='Illum_Retina_BXD_RankInv0410',ProbeSetID2='ILMN_2623591',CellID2='',rank='0')) | [**0.420 79**](javascript:showCorrelationPlot2(db='Illum_Retina_BXD_RankInv0410',ProbeSetID='ILMN_2992541',CellID='',db2='Illum_Retina_BXD_RankInv0410',ProbeSetID2='ILMN_2603568',CellID2='',rank='0')) | [***n* 79**](javascript:showDatabase2('Illum_Retina_BXD_RankInv0410','ILMN_2992541','')) | [**0.524 79**](javascript:showCorrelationPlot2(db='Illum_Retina_BXD_RankInv0410',ProbeSetID='ILMN_2992541',CellID='',db2='Illum_Retina_BXD_RankInv0410',ProbeSetID2='ILMN_2792868',CellID2='',rank='1')) | [**0.479 79**](javascript:showCorrelationPlot2(db='Illum_Retina_BXD_RankInv0410',ProbeSetID='ILMN_2992541',CellID='',db2='Illum_Retina_BXD_RankInv0410',ProbeSetID2='ILMN_3129497',CellID2='',rank='1')) | [**0.448 79**](javascript:showCorrelationPlot2(db='Illum_Retina_BXD_RankInv0410',ProbeSetID='ILMN_2992541',CellID='',db2='Illum_Retina_BXD_RankInv0410',ProbeSetID2='ILMN_1224034',CellID2='',rank='1')) | [**0.596 79**](javascript:showCorrelationPlot2(db='Illum_Retina_BXD_RankInv0410',ProbeSetID='ILMN_2992541',CellID='',db2='Illum_Retina_BXD_RankInv0410',ProbeSetID2='ILMN_2639819',CellID2='',rank='1')) | [**0.714 79**](javascript:showCorrelationPlot2(db='Illum_Retina_BXD_RankInv0410',ProbeSetID='ILMN_2992541',CellID='',db2='Illum_Retina_BXD_RankInv0410',ProbeSetID2='ILMN_2994779',CellID2='',rank='1')) | [**0.609 79**](javascript:showCorrelationPlot2(db='Illum_Retina_BXD_RankInv0410',ProbeSetID='ILMN_2992541',CellID='',db2='Illum_Retina_BXD_RankInv0410',ProbeSetID2='ILMN_2625047',CellID2='',rank='1')) | [**0.325 79**](javascript:showCorrelationPlot2(db='Illum_Retina_BXD_RankInv0410',ProbeSetID='ILMN_2992541',CellID='',db2='Illum_Retina_BXD_RankInv0410',ProbeSetID2='ILMN_2808186',CellID2='',rank='1')) | [**0.498 79**](javascript:showCorrelationPlot2(db='Illum_Retina_BXD_RankInv0410',ProbeSetID='ILMN_2992541',CellID='',db2='Illum_Retina_BXD_RankInv0410',ProbeSetID2='ILMN_2689056',CellID2='',rank='1')) | [**0.648 79**](javascript:showCorrelationPlot2(db='Illum_Retina_BXD_RankInv0410',ProbeSetID='ILMN_2992541',CellID='',db2='Illum_Retina_BXD_RankInv0410',ProbeSetID2='ILMN_2648386',CellID2='',rank='1')) | [**0.736 79**](javascript:showCorrelationPlot2(db='Illum_Retina_BXD_RankInv0410',ProbeSetID='ILMN_2992541',CellID='',db2='Illum_Retina_BXD_RankInv0410',ProbeSetID2='ILMN_2869225',CellID2='',rank='1')) | [**0.513 79**](javascript:showCorrelationPlot2(db='Illum_Retina_BXD_RankInv0410',ProbeSetID='ILMN_2992541',CellID='',db2='Illum_Retina_BXD_RankInv0410',ProbeSetID2='ILMN_2728431',CellID2='',rank='1')) | [**0.395 79**](javascript:showCorrelationPlot2(db='Illum_Retina_BXD_RankInv0410',ProbeSetID='ILMN_2992541',CellID='',db2='Illum_Retina_BXD_RankInv0410',ProbeSetID2='ILMN_3094608',CellID2='',rank='1')) | [**0.671 79**](javascript:showCorrelationPlot2(db='Illum_Retina_BXD_RankInv0410',ProbeSetID='ILMN_2992541',CellID='',db2='Illum_Retina_BXD_RankInv0410',ProbeSetID2='ILMN_2896528',CellID2='',rank='1')) | [**0.325 79**](javascript:showCorrelationPlot2(db='Illum_Retina_BXD_RankInv0410',ProbeSetID='ILMN_2992541',CellID='',db2='Illum_Retina_BXD_RankInv0410',ProbeSetID2='ILMN_2701750',CellID2='',rank='1')) | [**0.399 79**](javascript:showCorrelationPlot2(db='Illum_Retina_BXD_RankInv0410',ProbeSetID='ILMN_2992541',CellID='',db2='Illum_Retina_BXD_RankInv0410',ProbeSetID2='ILMN_1239448',CellID2='',rank='1')) | [**-0.479 79**](javascript:showCorrelationPlot2(db='Illum_Retina_BXD_RankInv0410',ProbeSetID='ILMN_2992541',CellID='',db2='Illum_Retina_BXD_RankInv0410',ProbeSetID2='ILMN_2583882',CellID2='',rank='1')) | [**0.596 79**](javascript:showCorrelationPlot2(db='Illum_Retina_BXD_RankInv0410',ProbeSetID='ILMN_2992541',CellID='',db2='Illum_Retina_BXD_RankInv0410',ProbeSetID2='ILMN_2915166',CellID2='',rank='1')) | [**0.473 79**](javascript:showCorrelationPlot2(db='Illum_Retina_BXD_RankInv0410',ProbeSetID='ILMN_2992541',CellID='',db2='Illum_Retina_BXD_RankInv0410',ProbeSetID2='ILMN_2702997',CellID2='',rank='1')) | [**0.433 79**](javascript:showCorrelationPlot2(db='Illum_Retina_BXD_RankInv0410',ProbeSetID='ILMN_2992541',CellID='',db2='Illum_Retina_BXD_RankInv0410',ProbeSetID2='ILMN_2435206',CellID2='',rank='1')) | [**0.471 79**](javascript:showCorrelationPlot2(db='Illum_Retina_BXD_RankInv0410',ProbeSetID='ILMN_2992541',CellID='',db2='Illum_Retina_BXD_RankInv0410',ProbeSetID2='ILMN_2789888',CellID2='',rank='1')) | [**0.397 79**](javascript:showCorrelationPlot2(db='Illum_Retina_BXD_RankInv0410',ProbeSetID='ILMN_2992541',CellID='',db2='Illum_Retina_BXD_RankInv0410',ProbeSetID2='ILMN_1218901',CellID2='',rank='1')) | [**0.481 79**](javascript:showCorrelationPlot2(db='Illum_Retina_BXD_RankInv0410',ProbeSetID='ILMN_2992541',CellID='',db2='Illum_Retina_BXD_RankInv0410',ProbeSetID2='ILMN_1254734',CellID2='',rank='1')) | [**0.449 79**](javascript:showCorrelationPlot2(db='Illum_Retina_BXD_RankInv0410',ProbeSetID='ILMN_2992541',CellID='',db2='Illum_Retina_BXD_RankInv0410',ProbeSetID2='ILMN_3008924',CellID2='',rank='1')) | [**0.536 79**](javascript:showCorrelationPlot2(db='Illum_Retina_BXD_RankInv0410',ProbeSetID='ILMN_2992541',CellID='',db2='Illum_Retina_BXD_RankInv0410',ProbeSetID2='ILMN_2628175',CellID2='',rank='1')) | [**0.617 79**](javascript:showCorrelationPlot2(db='Illum_Retina_BXD_RankInv0410',ProbeSetID='ILMN_2992541',CellID='',db2='Illum_Retina_BXD_RankInv0410',ProbeSetID2='ILMN_2891573',CellID2='',rank='1')) | [**0.583 79**](javascript:showCorrelationPlot2(db='Illum_Retina_BXD_RankInv0410',ProbeSetID='ILMN_2992541',CellID='',db2='Illum_Retina_BXD_RankInv0410',ProbeSetID2='ILMN_1220846',CellID2='',rank='1')) | [**0.641 79**](javascript:showCorrelationPlot2(db='Illum_Retina_BXD_RankInv0410',ProbeSetID='ILMN_2992541',CellID='',db2='Illum_Retina_BXD_RankInv0410',ProbeSetID2='ILMN_1241962',CellID2='',rank='1')) | [**0.709 79**](javascript:showCorrelationPlot2(db='Illum_Retina_BXD_RankInv0410',ProbeSetID='ILMN_2992541',CellID='',db2='Illum_Retina_BXD_RankInv0410',ProbeSetID2='ILMN_3163027',CellID2='',rank='1')) | [**0.324 79**](javascript:showCorrelationPlot2(db='Illum_Retina_BXD_RankInv0410',ProbeSetID='ILMN_2992541',CellID='',db2='Illum_Retina_BXD_RankInv0410',ProbeSetID2='ILMN_2775098',CellID2='',rank='1')) | [**0.563 79**](javascript:showCorrelationPlot2(db='Illum_Retina_BXD_RankInv0410',ProbeSetID='ILMN_2992541',CellID='',db2='Illum_Retina_BXD_RankInv0410',ProbeSetID2='ILMN_2752552',CellID2='',rank='1')) | [**0.449 79**](javascript:showCorrelationPlot2(db='Illum_Retina_BXD_RankInv0410',ProbeSetID='ILMN_2992541',CellID='',db2='Illum_Retina_BXD_RankInv0410',ProbeSetID2='ILMN_2730293',CellID2='',rank='1')) | [**0.377 79**](javascript:showCorrelationPlot2(db='Illum_Retina_BXD_RankInv0410',ProbeSetID='ILMN_2992541',CellID='',db2='Illum_Retina_BXD_RankInv0410',ProbeSetID2='ILMN_2680872',CellID2='',rank='1')) | [**0.227 79**](javascript:showCorrelationPlot2(db='Illum_Retina_BXD_RankInv0410',ProbeSetID='ILMN_2992541',CellID='',db2='Illum_Retina_BXD_RankInv0410',ProbeSetID2='ILMN_2768533',CellID2='',rank='1')) | [**0.049 79**](javascript:showCorrelationPlot2(db='Illum_Retina_BXD_RankInv0410',ProbeSetID='ILMN_2992541',CellID='',db2='Illum_Retina_BXD_RankInv0410',ProbeSetID2='ILMN_3121255',CellID2='',rank='1')) | [**-0.305 79**](javascript:showCorrelationPlot2(db='Illum_Retina_BXD_RankInv0410',ProbeSetID='ILMN_2992541',CellID='',db2='Illum_Retina_BXD_RankInv0410',ProbeSetID2='ILMN_2484527',CellID2='',rank='1')) | [**0.360 79**](javascript:showCorrelationPlot2(db='Illum_Retina_BXD_RankInv0410',ProbeSetID='ILMN_2992541',CellID='',db2='Illum_Retina_BXD_RankInv0410',ProbeSetID2='ILMN_3045723',CellID2='',rank='1')) | [**0.029 79**](javascript:showCorrelationPlot2(db='Illum_Retina_BXD_RankInv0410',ProbeSetID='ILMN_2992541',CellID='',db2='Illum_Retina_BXD_RankInv0410',ProbeSetID2='ILMN_2486573',CellID2='',rank='1')) |
|  | [**Trait 20: ILMN_2792868**](javascript:showDatabase2('Illum_Retina_BXD_RankInv0410','ILMN_2792868','');)  Symbol: Blvrb  Blvrb | [**0.663 79**](javascript:showCorrelationPlot2(db='Illum_Retina_BXD_RankInv0410',ProbeSetID='ILMN_2792868',CellID='',db2='Illum_Retina_BXD_RankInv0410',ProbeSetID2='ILMN_2450384',CellID2='',rank='0')) | [**0.437 79**](javascript:showCorrelationPlot2(db='Illum_Retina_BXD_RankInv0410',ProbeSetID='ILMN_2792868',CellID='',db2='Illum_Retina_BXD_RankInv0410',ProbeSetID2='ILMN_3068754',CellID2='',rank='0')) | [**0.473 79**](javascript:showCorrelationPlot2(db='Illum_Retina_BXD_RankInv0410',ProbeSetID='ILMN_2792868',CellID='',db2='Illum_Retina_BXD_RankInv0410',ProbeSetID2='ILMN_2677092',CellID2='',rank='0')) | [**0.479 79**](javascript:showCorrelationPlot2(db='Illum_Retina_BXD_RankInv0410',ProbeSetID='ILMN_2792868',CellID='',db2='Illum_Retina_BXD_RankInv0410',ProbeSetID2='ILMN_2624938',CellID2='',rank='0')) | [**0.566 79**](javascript:showCorrelationPlot2(db='Illum_Retina_BXD_RankInv0410',ProbeSetID='ILMN_2792868',CellID='',db2='Illum_Retina_BXD_RankInv0410',ProbeSetID2='ILMN_2847269',CellID2='',rank='0')) | [**0.409 79**](javascript:showCorrelationPlot2(db='Illum_Retina_BXD_RankInv0410',ProbeSetID='ILMN_2792868',CellID='',db2='Illum_Retina_BXD_RankInv0410',ProbeSetID2='ILMN_2839682',CellID2='',rank='0')) | [**0.538 79**](javascript:showCorrelationPlot2(db='Illum_Retina_BXD_RankInv0410',ProbeSetID='ILMN_2792868',CellID='',db2='Illum_Retina_BXD_RankInv0410',ProbeSetID2='ILMN_3147135',CellID2='',rank='0')) | [**0.579 79**](javascript:showCorrelationPlot2(db='Illum_Retina_BXD_RankInv0410',ProbeSetID='ILMN_2792868',CellID='',db2='Illum_Retina_BXD_RankInv0410',ProbeSetID2='ILMN_1255823',CellID2='',rank='0')) | [**0.481 79**](javascript:showCorrelationPlot2(db='Illum_Retina_BXD_RankInv0410',ProbeSetID='ILMN_2792868',CellID='',db2='Illum_Retina_BXD_RankInv0410',ProbeSetID2='ILMN_2626143',CellID2='',rank='0')) | [**0.610 79**](javascript:showCorrelationPlot2(db='Illum_Retina_BXD_RankInv0410',ProbeSetID='ILMN_2792868',CellID='',db2='Illum_Retina_BXD_RankInv0410',ProbeSetID2='ILMN_1234930',CellID2='',rank='0')) | [**0.698 79**](javascript:showCorrelationPlot2(db='Illum_Retina_BXD_RankInv0410',ProbeSetID='ILMN_2792868',CellID='',db2='Illum_Retina_BXD_RankInv0410',ProbeSetID2='ILMN_1249408',CellID2='',rank='0')) | [**0.382 79**](javascript:showCorrelationPlot2(db='Illum_Retina_BXD_RankInv0410',ProbeSetID='ILMN_2792868',CellID='',db2='Illum_Retina_BXD_RankInv0410',ProbeSetID2='ILMN_2971559',CellID2='',rank='0')) | [**0.501 79**](javascript:showCorrelationPlot2(db='Illum_Retina_BXD_RankInv0410',ProbeSetID='ILMN_2792868',CellID='',db2='Illum_Retina_BXD_RankInv0410',ProbeSetID2='ILMN_1258455',CellID2='',rank='0')) | [**0.529 79**](javascript:showCorrelationPlot2(db='Illum_Retina_BXD_RankInv0410',ProbeSetID='ILMN_2792868',CellID='',db2='Illum_Retina_BXD_RankInv0410',ProbeSetID2='ILMN_2691613',CellID2='',rank='0')) | [**0.452 79**](javascript:showCorrelationPlot2(db='Illum_Retina_BXD_RankInv0410',ProbeSetID='ILMN_2792868',CellID='',db2='Illum_Retina_BXD_RankInv0410',ProbeSetID2='ILMN_1253600',CellID2='',rank='0')) | [**0.522 79**](javascript:showCorrelationPlot2(db='Illum_Retina_BXD_RankInv0410',ProbeSetID='ILMN_2792868',CellID='',db2='Illum_Retina_BXD_RankInv0410',ProbeSetID2='ILMN_2991545',CellID2='',rank='0')) | [**0.340 79**](javascript:showCorrelationPlot2(db='Illum_Retina_BXD_RankInv0410',ProbeSetID='ILMN_2792868',CellID='',db2='Illum_Retina_BXD_RankInv0410',ProbeSetID2='ILMN_2623591',CellID2='',rank='0')) | [**0.578 79**](javascript:showCorrelationPlot2(db='Illum_Retina_BXD_RankInv0410',ProbeSetID='ILMN_2792868',CellID='',db2='Illum_Retina_BXD_RankInv0410',ProbeSetID2='ILMN_2603568',CellID2='',rank='0')) | [**0.495 79**](javascript:showCorrelationPlot2(db='Illum_Retina_BXD_RankInv0410',ProbeSetID='ILMN_2792868',CellID='',db2='Illum_Retina_BXD_RankInv0410',ProbeSetID2='ILMN_2992541',CellID2='',rank='0')) | [***n* 79**](javascript:showDatabase2('Illum_Retina_BXD_RankInv0410','ILMN_2792868','')) | [**0.418 79**](javascript:showCorrelationPlot2(db='Illum_Retina_BXD_RankInv0410',ProbeSetID='ILMN_2792868',CellID='',db2='Illum_Retina_BXD_RankInv0410',ProbeSetID2='ILMN_3129497',CellID2='',rank='1')) | [**0.534 79**](javascript:showCorrelationPlot2(db='Illum_Retina_BXD_RankInv0410',ProbeSetID='ILMN_2792868',CellID='',db2='Illum_Retina_BXD_RankInv0410',ProbeSetID2='ILMN_1224034',CellID2='',rank='1')) | [**0.520 79**](javascript:showCorrelationPlot2(db='Illum_Retina_BXD_RankInv0410',ProbeSetID='ILMN_2792868',CellID='',db2='Illum_Retina_BXD_RankInv0410',ProbeSetID2='ILMN_2639819',CellID2='',rank='1')) | [**0.455 79**](javascript:showCorrelationPlot2(db='Illum_Retina_BXD_RankInv0410',ProbeSetID='ILMN_2792868',CellID='',db2='Illum_Retina_BXD_RankInv0410',ProbeSetID2='ILMN_2994779',CellID2='',rank='1')) | [**0.539 79**](javascript:showCorrelationPlot2(db='Illum_Retina_BXD_RankInv0410',ProbeSetID='ILMN_2792868',CellID='',db2='Illum_Retina_BXD_RankInv0410',ProbeSetID2='ILMN_2625047',CellID2='',rank='1')) | [**0.269 79**](javascript:showCorrelationPlot2(db='Illum_Retina_BXD_RankInv0410',ProbeSetID='ILMN_2792868',CellID='',db2='Illum_Retina_BXD_RankInv0410',ProbeSetID2='ILMN_2808186',CellID2='',rank='1')) | [**0.302 79**](javascript:showCorrelationPlot2(db='Illum_Retina_BXD_RankInv0410',ProbeSetID='ILMN_2792868',CellID='',db2='Illum_Retina_BXD_RankInv0410',ProbeSetID2='ILMN_2689056',CellID2='',rank='1')) | [**0.646 79**](javascript:showCorrelationPlot2(db='Illum_Retina_BXD_RankInv0410',ProbeSetID='ILMN_2792868',CellID='',db2='Illum_Retina_BXD_RankInv0410',ProbeSetID2='ILMN_2648386',CellID2='',rank='1')) | [**0.547 79**](javascript:showCorrelationPlot2(db='Illum_Retina_BXD_RankInv0410',ProbeSetID='ILMN_2792868',CellID='',db2='Illum_Retina_BXD_RankInv0410',ProbeSetID2='ILMN_2869225',CellID2='',rank='1')) | [**0.555 79**](javascript:showCorrelationPlot2(db='Illum_Retina_BXD_RankInv0410',ProbeSetID='ILMN_2792868',CellID='',db2='Illum_Retina_BXD_RankInv0410',ProbeSetID2='ILMN_2728431',CellID2='',rank='1')) | [**0.381 79**](javascript:showCorrelationPlot2(db='Illum_Retina_BXD_RankInv0410',ProbeSetID='ILMN_2792868',CellID='',db2='Illum_Retina_BXD_RankInv0410',ProbeSetID2='ILMN_3094608',CellID2='',rank='1')) | [**0.586 79**](javascript:showCorrelationPlot2(db='Illum_Retina_BXD_RankInv0410',ProbeSetID='ILMN_2792868',CellID='',db2='Illum_Retina_BXD_RankInv0410',ProbeSetID2='ILMN_2896528',CellID2='',rank='1')) | [**0.664 79**](javascript:showCorrelationPlot2(db='Illum_Retina_BXD_RankInv0410',ProbeSetID='ILMN_2792868',CellID='',db2='Illum_Retina_BXD_RankInv0410',ProbeSetID2='ILMN_2701750',CellID2='',rank='1')) | [**0.403 79**](javascript:showCorrelationPlot2(db='Illum_Retina_BXD_RankInv0410',ProbeSetID='ILMN_2792868',CellID='',db2='Illum_Retina_BXD_RankInv0410',ProbeSetID2='ILMN_1239448',CellID2='',rank='1')) | [**-0.444 79**](javascript:showCorrelationPlot2(db='Illum_Retina_BXD_RankInv0410',ProbeSetID='ILMN_2792868',CellID='',db2='Illum_Retina_BXD_RankInv0410',ProbeSetID2='ILMN_2583882',CellID2='',rank='1')) | [**0.461 79**](javascript:showCorrelationPlot2(db='Illum_Retina_BXD_RankInv0410',ProbeSetID='ILMN_2792868',CellID='',db2='Illum_Retina_BXD_RankInv0410',ProbeSetID2='ILMN_2915166',CellID2='',rank='1')) | [**0.526 79**](javascript:showCorrelationPlot2(db='Illum_Retina_BXD_RankInv0410',ProbeSetID='ILMN_2792868',CellID='',db2='Illum_Retina_BXD_RankInv0410',ProbeSetID2='ILMN_2702997',CellID2='',rank='1')) | [**0.415 79**](javascript:showCorrelationPlot2(db='Illum_Retina_BXD_RankInv0410',ProbeSetID='ILMN_2792868',CellID='',db2='Illum_Retina_BXD_RankInv0410',ProbeSetID2='ILMN_2435206',CellID2='',rank='1')) | [**0.287 79**](javascript:showCorrelationPlot2(db='Illum_Retina_BXD_RankInv0410',ProbeSetID='ILMN_2792868',CellID='',db2='Illum_Retina_BXD_RankInv0410',ProbeSetID2='ILMN_2789888',CellID2='',rank='1')) | [**0.207 79**](javascript:showCorrelationPlot2(db='Illum_Retina_BXD_RankInv0410',ProbeSetID='ILMN_2792868',CellID='',db2='Illum_Retina_BXD_RankInv0410',ProbeSetID2='ILMN_1218901',CellID2='',rank='1')) | [**0.479 79**](javascript:showCorrelationPlot2(db='Illum_Retina_BXD_RankInv0410',ProbeSetID='ILMN_2792868',CellID='',db2='Illum_Retina_BXD_RankInv0410',ProbeSetID2='ILMN_1254734',CellID2='',rank='1')) | [**0.450 79**](javascript:showCorrelationPlot2(db='Illum_Retina_BXD_RankInv0410',ProbeSetID='ILMN_2792868',CellID='',db2='Illum_Retina_BXD_RankInv0410',ProbeSetID2='ILMN_3008924',CellID2='',rank='1')) | [**0.339 79**](javascript:showCorrelationPlot2(db='Illum_Retina_BXD_RankInv0410',ProbeSetID='ILMN_2792868',CellID='',db2='Illum_Retina_BXD_RankInv0410',ProbeSetID2='ILMN_2628175',CellID2='',rank='1')) | [**0.314 79**](javascript:showCorrelationPlot2(db='Illum_Retina_BXD_RankInv0410',ProbeSetID='ILMN_2792868',CellID='',db2='Illum_Retina_BXD_RankInv0410',ProbeSetID2='ILMN_2891573',CellID2='',rank='1')) | [**0.455 79**](javascript:showCorrelationPlot2(db='Illum_Retina_BXD_RankInv0410',ProbeSetID='ILMN_2792868',CellID='',db2='Illum_Retina_BXD_RankInv0410',ProbeSetID2='ILMN_1220846',CellID2='',rank='1')) | [**0.594 79**](javascript:showCorrelationPlot2(db='Illum_Retina_BXD_RankInv0410',ProbeSetID='ILMN_2792868',CellID='',db2='Illum_Retina_BXD_RankInv0410',ProbeSetID2='ILMN_1241962',CellID2='',rank='1')) | [**0.344 79**](javascript:showCorrelationPlot2(db='Illum_Retina_BXD_RankInv0410',ProbeSetID='ILMN_2792868',CellID='',db2='Illum_Retina_BXD_RankInv0410',ProbeSetID2='ILMN_3163027',CellID2='',rank='1')) | [**0.667 79**](javascript:showCorrelationPlot2(db='Illum_Retina_BXD_RankInv0410',ProbeSetID='ILMN_2792868',CellID='',db2='Illum_Retina_BXD_RankInv0410',ProbeSetID2='ILMN_2775098',CellID2='',rank='1')) | [**0.400 79**](javascript:showCorrelationPlot2(db='Illum_Retina_BXD_RankInv0410',ProbeSetID='ILMN_2792868',CellID='',db2='Illum_Retina_BXD_RankInv0410',ProbeSetID2='ILMN_2752552',CellID2='',rank='1')) | [**0.508 79**](javascript:showCorrelationPlot2(db='Illum_Retina_BXD_RankInv0410',ProbeSetID='ILMN_2792868',CellID='',db2='Illum_Retina_BXD_RankInv0410',ProbeSetID2='ILMN_2730293',CellID2='',rank='1')) | [**0.416 79**](javascript:showCorrelationPlot2(db='Illum_Retina_BXD_RankInv0410',ProbeSetID='ILMN_2792868',CellID='',db2='Illum_Retina_BXD_RankInv0410',ProbeSetID2='ILMN_2680872',CellID2='',rank='1')) | [**0.369 79**](javascript:showCorrelationPlot2(db='Illum_Retina_BXD_RankInv0410',ProbeSetID='ILMN_2792868',CellID='',db2='Illum_Retina_BXD_RankInv0410',ProbeSetID2='ILMN_2768533',CellID2='',rank='1')) | [**-0.022 79**](javascript:showCorrelationPlot2(db='Illum_Retina_BXD_RankInv0410',ProbeSetID='ILMN_2792868',CellID='',db2='Illum_Retina_BXD_RankInv0410',ProbeSetID2='ILMN_3121255',CellID2='',rank='1')) | [**-0.096 79**](javascript:showCorrelationPlot2(db='Illum_Retina_BXD_RankInv0410',ProbeSetID='ILMN_2792868',CellID='',db2='Illum_Retina_BXD_RankInv0410',ProbeSetID2='ILMN_2484527',CellID2='',rank='1')) | [**0.065 79**](javascript:showCorrelationPlot2(db='Illum_Retina_BXD_RankInv0410',ProbeSetID='ILMN_2792868',CellID='',db2='Illum_Retina_BXD_RankInv0410',ProbeSetID2='ILMN_3045723',CellID2='',rank='1')) | [**0.380 79**](javascript:showCorrelationPlot2(db='Illum_Retina_BXD_RankInv0410',ProbeSetID='ILMN_2792868',CellID='',db2='Illum_Retina_BXD_RankInv0410',ProbeSetID2='ILMN_2486573',CellID2='',rank='1')) |
|  | [**Trait 21: ILMN_3129497**](javascript:showDatabase2('Illum_Retina_BXD_RankInv0410','ILMN_3129497','');)  Symbol: Nsmf  Nsmf | [**0.663 79**](javascript:showCorrelationPlot2(db='Illum_Retina_BXD_RankInv0410',ProbeSetID='ILMN_3129497',CellID='',db2='Illum_Retina_BXD_RankInv0410',ProbeSetID2='ILMN_2450384',CellID2='',rank='0')) | [**0.588 79**](javascript:showCorrelationPlot2(db='Illum_Retina_BXD_RankInv0410',ProbeSetID='ILMN_3129497',CellID='',db2='Illum_Retina_BXD_RankInv0410',ProbeSetID2='ILMN_3068754',CellID2='',rank='0')) | [**0.616 79**](javascript:showCorrelationPlot2(db='Illum_Retina_BXD_RankInv0410',ProbeSetID='ILMN_3129497',CellID='',db2='Illum_Retina_BXD_RankInv0410',ProbeSetID2='ILMN_2677092',CellID2='',rank='0')) | [**0.717 79**](javascript:showCorrelationPlot2(db='Illum_Retina_BXD_RankInv0410',ProbeSetID='ILMN_3129497',CellID='',db2='Illum_Retina_BXD_RankInv0410',ProbeSetID2='ILMN_2624938',CellID2='',rank='0')) | [**0.581 79**](javascript:showCorrelationPlot2(db='Illum_Retina_BXD_RankInv0410',ProbeSetID='ILMN_3129497',CellID='',db2='Illum_Retina_BXD_RankInv0410',ProbeSetID2='ILMN_2847269',CellID2='',rank='0')) | [**0.521 79**](javascript:showCorrelationPlot2(db='Illum_Retina_BXD_RankInv0410',ProbeSetID='ILMN_3129497',CellID='',db2='Illum_Retina_BXD_RankInv0410',ProbeSetID2='ILMN_2839682',CellID2='',rank='0')) | [**0.386 79**](javascript:showCorrelationPlot2(db='Illum_Retina_BXD_RankInv0410',ProbeSetID='ILMN_3129497',CellID='',db2='Illum_Retina_BXD_RankInv0410',ProbeSetID2='ILMN_3147135',CellID2='',rank='0')) | [**0.413 79**](javascript:showCorrelationPlot2(db='Illum_Retina_BXD_RankInv0410',ProbeSetID='ILMN_3129497',CellID='',db2='Illum_Retina_BXD_RankInv0410',ProbeSetID2='ILMN_1255823',CellID2='',rank='0')) | [**0.592 79**](javascript:showCorrelationPlot2(db='Illum_Retina_BXD_RankInv0410',ProbeSetID='ILMN_3129497',CellID='',db2='Illum_Retina_BXD_RankInv0410',ProbeSetID2='ILMN_2626143',CellID2='',rank='0')) | [**0.690 79**](javascript:showCorrelationPlot2(db='Illum_Retina_BXD_RankInv0410',ProbeSetID='ILMN_3129497',CellID='',db2='Illum_Retina_BXD_RankInv0410',ProbeSetID2='ILMN_1234930',CellID2='',rank='0')) | [**0.487 79**](javascript:showCorrelationPlot2(db='Illum_Retina_BXD_RankInv0410',ProbeSetID='ILMN_3129497',CellID='',db2='Illum_Retina_BXD_RankInv0410',ProbeSetID2='ILMN_1249408',CellID2='',rank='0')) | [**0.646 79**](javascript:showCorrelationPlot2(db='Illum_Retina_BXD_RankInv0410',ProbeSetID='ILMN_3129497',CellID='',db2='Illum_Retina_BXD_RankInv0410',ProbeSetID2='ILMN_2971559',CellID2='',rank='0')) | [**0.563 79**](javascript:showCorrelationPlot2(db='Illum_Retina_BXD_RankInv0410',ProbeSetID='ILMN_3129497',CellID='',db2='Illum_Retina_BXD_RankInv0410',ProbeSetID2='ILMN_1258455',CellID2='',rank='0')) | [**0.384 79**](javascript:showCorrelationPlot2(db='Illum_Retina_BXD_RankInv0410',ProbeSetID='ILMN_3129497',CellID='',db2='Illum_Retina_BXD_RankInv0410',ProbeSetID2='ILMN_2691613',CellID2='',rank='0')) | [**0.603 79**](javascript:showCorrelationPlot2(db='Illum_Retina_BXD_RankInv0410',ProbeSetID='ILMN_3129497',CellID='',db2='Illum_Retina_BXD_RankInv0410',ProbeSetID2='ILMN_1253600',CellID2='',rank='0')) | [**0.508 79**](javascript:showCorrelationPlot2(db='Illum_Retina_BXD_RankInv0410',ProbeSetID='ILMN_3129497',CellID='',db2='Illum_Retina_BXD_RankInv0410',ProbeSetID2='ILMN_2991545',CellID2='',rank='0')) | [**0.671 79**](javascript:showCorrelationPlot2(db='Illum_Retina_BXD_RankInv0410',ProbeSetID='ILMN_3129497',CellID='',db2='Illum_Retina_BXD_RankInv0410',ProbeSetID2='ILMN_2623591',CellID2='',rank='0')) | [**0.337 79**](javascript:showCorrelationPlot2(db='Illum_Retina_BXD_RankInv0410',ProbeSetID='ILMN_3129497',CellID='',db2='Illum_Retina_BXD_RankInv0410',ProbeSetID2='ILMN_2603568',CellID2='',rank='0')) | [**0.473 79**](javascript:showCorrelationPlot2(db='Illum_Retina_BXD_RankInv0410',ProbeSetID='ILMN_3129497',CellID='',db2='Illum_Retina_BXD_RankInv0410',ProbeSetID2='ILMN_2992541',CellID2='',rank='0')) | [**0.420 79**](javascript:showCorrelationPlot2(db='Illum_Retina_BXD_RankInv0410',ProbeSetID='ILMN_3129497',CellID='',db2='Illum_Retina_BXD_RankInv0410',ProbeSetID2='ILMN_2792868',CellID2='',rank='0')) | [***n* 79**](javascript:showDatabase2('Illum_Retina_BXD_RankInv0410','ILMN_3129497','')) | [**0.718 79**](javascript:showCorrelationPlot2(db='Illum_Retina_BXD_RankInv0410',ProbeSetID='ILMN_3129497',CellID='',db2='Illum_Retina_BXD_RankInv0410',ProbeSetID2='ILMN_1224034',CellID2='',rank='1')) | [**0.511 79**](javascript:showCorrelationPlot2(db='Illum_Retina_BXD_RankInv0410',ProbeSetID='ILMN_3129497',CellID='',db2='Illum_Retina_BXD_RankInv0410',ProbeSetID2='ILMN_2639819',CellID2='',rank='1')) | [**0.480 79**](javascript:showCorrelationPlot2(db='Illum_Retina_BXD_RankInv0410',ProbeSetID='ILMN_3129497',CellID='',db2='Illum_Retina_BXD_RankInv0410',ProbeSetID2='ILMN_2994779',CellID2='',rank='1')) | [**0.613 79**](javascript:showCorrelationPlot2(db='Illum_Retina_BXD_RankInv0410',ProbeSetID='ILMN_3129497',CellID='',db2='Illum_Retina_BXD_RankInv0410',ProbeSetID2='ILMN_2625047',CellID2='',rank='1')) | [**0.555 79**](javascript:showCorrelationPlot2(db='Illum_Retina_BXD_RankInv0410',ProbeSetID='ILMN_3129497',CellID='',db2='Illum_Retina_BXD_RankInv0410',ProbeSetID2='ILMN_2808186',CellID2='',rank='1')) | [**0.408 79**](javascript:showCorrelationPlot2(db='Illum_Retina_BXD_RankInv0410',ProbeSetID='ILMN_3129497',CellID='',db2='Illum_Retina_BXD_RankInv0410',ProbeSetID2='ILMN_2689056',CellID2='',rank='1')) | [**0.408 79**](javascript:showCorrelationPlot2(db='Illum_Retina_BXD_RankInv0410',ProbeSetID='ILMN_3129497',CellID='',db2='Illum_Retina_BXD_RankInv0410',ProbeSetID2='ILMN_2648386',CellID2='',rank='1')) | [**0.522 79**](javascript:showCorrelationPlot2(db='Illum_Retina_BXD_RankInv0410',ProbeSetID='ILMN_3129497',CellID='',db2='Illum_Retina_BXD_RankInv0410',ProbeSetID2='ILMN_2869225',CellID2='',rank='1')) | [**0.480 79**](javascript:showCorrelationPlot2(db='Illum_Retina_BXD_RankInv0410',ProbeSetID='ILMN_3129497',CellID='',db2='Illum_Retina_BXD_RankInv0410',ProbeSetID2='ILMN_2728431',CellID2='',rank='1')) | [**0.493 79**](javascript:showCorrelationPlot2(db='Illum_Retina_BXD_RankInv0410',ProbeSetID='ILMN_3129497',CellID='',db2='Illum_Retina_BXD_RankInv0410',ProbeSetID2='ILMN_3094608',CellID2='',rank='1')) | [**0.384 79**](javascript:showCorrelationPlot2(db='Illum_Retina_BXD_RankInv0410',ProbeSetID='ILMN_3129497',CellID='',db2='Illum_Retina_BXD_RankInv0410',ProbeSetID2='ILMN_2896528',CellID2='',rank='1')) | [**0.419 79**](javascript:showCorrelationPlot2(db='Illum_Retina_BXD_RankInv0410',ProbeSetID='ILMN_3129497',CellID='',db2='Illum_Retina_BXD_RankInv0410',ProbeSetID2='ILMN_2701750',CellID2='',rank='1')) | [**0.541 79**](javascript:showCorrelationPlot2(db='Illum_Retina_BXD_RankInv0410',ProbeSetID='ILMN_3129497',CellID='',db2='Illum_Retina_BXD_RankInv0410',ProbeSetID2='ILMN_1239448',CellID2='',rank='1')) | [**-0.283 79**](javascript:showCorrelationPlot2(db='Illum_Retina_BXD_RankInv0410',ProbeSetID='ILMN_3129497',CellID='',db2='Illum_Retina_BXD_RankInv0410',ProbeSetID2='ILMN_2583882',CellID2='',rank='1')) | [**0.451 79**](javascript:showCorrelationPlot2(db='Illum_Retina_BXD_RankInv0410',ProbeSetID='ILMN_3129497',CellID='',db2='Illum_Retina_BXD_RankInv0410',ProbeSetID2='ILMN_2915166',CellID2='',rank='1')) | [**0.519 79**](javascript:showCorrelationPlot2(db='Illum_Retina_BXD_RankInv0410',ProbeSetID='ILMN_3129497',CellID='',db2='Illum_Retina_BXD_RankInv0410',ProbeSetID2='ILMN_2702997',CellID2='',rank='1')) | [**0.604 79**](javascript:showCorrelationPlot2(db='Illum_Retina_BXD_RankInv0410',ProbeSetID='ILMN_3129497',CellID='',db2='Illum_Retina_BXD_RankInv0410',ProbeSetID2='ILMN_2435206',CellID2='',rank='1')) | [**0.531 79**](javascript:showCorrelationPlot2(db='Illum_Retina_BXD_RankInv0410',ProbeSetID='ILMN_3129497',CellID='',db2='Illum_Retina_BXD_RankInv0410',ProbeSetID2='ILMN_2789888',CellID2='',rank='1')) | [**0.536 79**](javascript:showCorrelationPlot2(db='Illum_Retina_BXD_RankInv0410',ProbeSetID='ILMN_3129497',CellID='',db2='Illum_Retina_BXD_RankInv0410',ProbeSetID2='ILMN_1218901',CellID2='',rank='1')) | [**0.425 79**](javascript:showCorrelationPlot2(db='Illum_Retina_BXD_RankInv0410',ProbeSetID='ILMN_3129497',CellID='',db2='Illum_Retina_BXD_RankInv0410',ProbeSetID2='ILMN_1254734',CellID2='',rank='1')) | [**0.555 79**](javascript:showCorrelationPlot2(db='Illum_Retina_BXD_RankInv0410',ProbeSetID='ILMN_3129497',CellID='',db2='Illum_Retina_BXD_RankInv0410',ProbeSetID2='ILMN_3008924',CellID2='',rank='1')) | [**0.429 79**](javascript:showCorrelationPlot2(db='Illum_Retina_BXD_RankInv0410',ProbeSetID='ILMN_3129497',CellID='',db2='Illum_Retina_BXD_RankInv0410',ProbeSetID2='ILMN_2628175',CellID2='',rank='1')) | [**0.441 79**](javascript:showCorrelationPlot2(db='Illum_Retina_BXD_RankInv0410',ProbeSetID='ILMN_3129497',CellID='',db2='Illum_Retina_BXD_RankInv0410',ProbeSetID2='ILMN_2891573',CellID2='',rank='1')) | [**0.613 79**](javascript:showCorrelationPlot2(db='Illum_Retina_BXD_RankInv0410',ProbeSetID='ILMN_3129497',CellID='',db2='Illum_Retina_BXD_RankInv0410',ProbeSetID2='ILMN_1220846',CellID2='',rank='1')) | [**0.407 79**](javascript:showCorrelationPlot2(db='Illum_Retina_BXD_RankInv0410',ProbeSetID='ILMN_3129497',CellID='',db2='Illum_Retina_BXD_RankInv0410',ProbeSetID2='ILMN_1241962',CellID2='',rank='1')) | [**0.513 79**](javascript:showCorrelationPlot2(db='Illum_Retina_BXD_RankInv0410',ProbeSetID='ILMN_3129497',CellID='',db2='Illum_Retina_BXD_RankInv0410',ProbeSetID2='ILMN_3163027',CellID2='',rank='1')) | [**0.249 79**](javascript:showCorrelationPlot2(db='Illum_Retina_BXD_RankInv0410',ProbeSetID='ILMN_3129497',CellID='',db2='Illum_Retina_BXD_RankInv0410',ProbeSetID2='ILMN_2775098',CellID2='',rank='1')) | [**0.536 79**](javascript:showCorrelationPlot2(db='Illum_Retina_BXD_RankInv0410',ProbeSetID='ILMN_3129497',CellID='',db2='Illum_Retina_BXD_RankInv0410',ProbeSetID2='ILMN_2752552',CellID2='',rank='1')) | [**0.729 79**](javascript:showCorrelationPlot2(db='Illum_Retina_BXD_RankInv0410',ProbeSetID='ILMN_3129497',CellID='',db2='Illum_Retina_BXD_RankInv0410',ProbeSetID2='ILMN_2730293',CellID2='',rank='1')) | [**0.601 79**](javascript:showCorrelationPlot2(db='Illum_Retina_BXD_RankInv0410',ProbeSetID='ILMN_3129497',CellID='',db2='Illum_Retina_BXD_RankInv0410',ProbeSetID2='ILMN_2680872',CellID2='',rank='1')) | [**0.050 79**](javascript:showCorrelationPlot2(db='Illum_Retina_BXD_RankInv0410',ProbeSetID='ILMN_3129497',CellID='',db2='Illum_Retina_BXD_RankInv0410',ProbeSetID2='ILMN_2768533',CellID2='',rank='1')) | [**-0.120 79**](javascript:showCorrelationPlot2(db='Illum_Retina_BXD_RankInv0410',ProbeSetID='ILMN_3129497',CellID='',db2='Illum_Retina_BXD_RankInv0410',ProbeSetID2='ILMN_3121255',CellID2='',rank='1')) | [**-0.140 79**](javascript:showCorrelationPlot2(db='Illum_Retina_BXD_RankInv0410',ProbeSetID='ILMN_3129497',CellID='',db2='Illum_Retina_BXD_RankInv0410',ProbeSetID2='ILMN_2484527',CellID2='',rank='1')) | [**-0.043 79**](javascript:showCorrelationPlot2(db='Illum_Retina_BXD_RankInv0410',ProbeSetID='ILMN_3129497',CellID='',db2='Illum_Retina_BXD_RankInv0410',ProbeSetID2='ILMN_3045723',CellID2='',rank='1')) | [**0.241 79**](javascript:showCorrelationPlot2(db='Illum_Retina_BXD_RankInv0410',ProbeSetID='ILMN_3129497',CellID='',db2='Illum_Retina_BXD_RankInv0410',ProbeSetID2='ILMN_2486573',CellID2='',rank='1')) |
|  | [**Trait 22: ILMN_1224034**](javascript:showDatabase2('Illum_Retina_BXD_RankInv0410','ILMN_1224034','');)  Symbol: Pde1b  Pde1b | [**0.662 79**](javascript:showCorrelationPlot2(db='Illum_Retina_BXD_RankInv0410',ProbeSetID='ILMN_1224034',CellID='',db2='Illum_Retina_BXD_RankInv0410',ProbeSetID2='ILMN_2450384',CellID2='',rank='0')) | [**0.496 79**](javascript:showCorrelationPlot2(db='Illum_Retina_BXD_RankInv0410',ProbeSetID='ILMN_1224034',CellID='',db2='Illum_Retina_BXD_RankInv0410',ProbeSetID2='ILMN_3068754',CellID2='',rank='0')) | [**0.615 79**](javascript:showCorrelationPlot2(db='Illum_Retina_BXD_RankInv0410',ProbeSetID='ILMN_1224034',CellID='',db2='Illum_Retina_BXD_RankInv0410',ProbeSetID2='ILMN_2677092',CellID2='',rank='0')) | [**0.594 79**](javascript:showCorrelationPlot2(db='Illum_Retina_BXD_RankInv0410',ProbeSetID='ILMN_1224034',CellID='',db2='Illum_Retina_BXD_RankInv0410',ProbeSetID2='ILMN_2624938',CellID2='',rank='0')) | [**0.623 79**](javascript:showCorrelationPlot2(db='Illum_Retina_BXD_RankInv0410',ProbeSetID='ILMN_1224034',CellID='',db2='Illum_Retina_BXD_RankInv0410',ProbeSetID2='ILMN_2847269',CellID2='',rank='0')) | [**0.566 79**](javascript:showCorrelationPlot2(db='Illum_Retina_BXD_RankInv0410',ProbeSetID='ILMN_1224034',CellID='',db2='Illum_Retina_BXD_RankInv0410',ProbeSetID2='ILMN_2839682',CellID2='',rank='0')) | [**0.500 79**](javascript:showCorrelationPlot2(db='Illum_Retina_BXD_RankInv0410',ProbeSetID='ILMN_1224034',CellID='',db2='Illum_Retina_BXD_RankInv0410',ProbeSetID2='ILMN_3147135',CellID2='',rank='0')) | [**0.396 79**](javascript:showCorrelationPlot2(db='Illum_Retina_BXD_RankInv0410',ProbeSetID='ILMN_1224034',CellID='',db2='Illum_Retina_BXD_RankInv0410',ProbeSetID2='ILMN_1255823',CellID2='',rank='0')) | [**0.471 79**](javascript:showCorrelationPlot2(db='Illum_Retina_BXD_RankInv0410',ProbeSetID='ILMN_1224034',CellID='',db2='Illum_Retina_BXD_RankInv0410',ProbeSetID2='ILMN_2626143',CellID2='',rank='0')) | [**0.678 79**](javascript:showCorrelationPlot2(db='Illum_Retina_BXD_RankInv0410',ProbeSetID='ILMN_1224034',CellID='',db2='Illum_Retina_BXD_RankInv0410',ProbeSetID2='ILMN_1234930',CellID2='',rank='0')) | [**0.435 79**](javascript:showCorrelationPlot2(db='Illum_Retina_BXD_RankInv0410',ProbeSetID='ILMN_1224034',CellID='',db2='Illum_Retina_BXD_RankInv0410',ProbeSetID2='ILMN_1249408',CellID2='',rank='0')) | [**0.657 79**](javascript:showCorrelationPlot2(db='Illum_Retina_BXD_RankInv0410',ProbeSetID='ILMN_1224034',CellID='',db2='Illum_Retina_BXD_RankInv0410',ProbeSetID2='ILMN_2971559',CellID2='',rank='0')) | [**0.617 79**](javascript:showCorrelationPlot2(db='Illum_Retina_BXD_RankInv0410',ProbeSetID='ILMN_1224034',CellID='',db2='Illum_Retina_BXD_RankInv0410',ProbeSetID2='ILMN_1258455',CellID2='',rank='0')) | [**0.474 79**](javascript:showCorrelationPlot2(db='Illum_Retina_BXD_RankInv0410',ProbeSetID='ILMN_1224034',CellID='',db2='Illum_Retina_BXD_RankInv0410',ProbeSetID2='ILMN_2691613',CellID2='',rank='0')) | [**0.577 79**](javascript:showCorrelationPlot2(db='Illum_Retina_BXD_RankInv0410',ProbeSetID='ILMN_1224034',CellID='',db2='Illum_Retina_BXD_RankInv0410',ProbeSetID2='ILMN_1253600',CellID2='',rank='0')) | [**0.483 79**](javascript:showCorrelationPlot2(db='Illum_Retina_BXD_RankInv0410',ProbeSetID='ILMN_1224034',CellID='',db2='Illum_Retina_BXD_RankInv0410',ProbeSetID2='ILMN_2991545',CellID2='',rank='0')) | [**0.546 79**](javascript:showCorrelationPlot2(db='Illum_Retina_BXD_RankInv0410',ProbeSetID='ILMN_1224034',CellID='',db2='Illum_Retina_BXD_RankInv0410',ProbeSetID2='ILMN_2623591',CellID2='',rank='0')) | [**0.327 79**](javascript:showCorrelationPlot2(db='Illum_Retina_BXD_RankInv0410',ProbeSetID='ILMN_1224034',CellID='',db2='Illum_Retina_BXD_RankInv0410',ProbeSetID2='ILMN_2603568',CellID2='',rank='0')) | [**0.462 79**](javascript:showCorrelationPlot2(db='Illum_Retina_BXD_RankInv0410',ProbeSetID='ILMN_1224034',CellID='',db2='Illum_Retina_BXD_RankInv0410',ProbeSetID2='ILMN_2992541',CellID2='',rank='0')) | [**0.585 79**](javascript:showCorrelationPlot2(db='Illum_Retina_BXD_RankInv0410',ProbeSetID='ILMN_1224034',CellID='',db2='Illum_Retina_BXD_RankInv0410',ProbeSetID2='ILMN_2792868',CellID2='',rank='0')) | [**0.669 79**](javascript:showCorrelationPlot2(db='Illum_Retina_BXD_RankInv0410',ProbeSetID='ILMN_1224034',CellID='',db2='Illum_Retina_BXD_RankInv0410',ProbeSetID2='ILMN_3129497',CellID2='',rank='0')) | [***n* 79**](javascript:showDatabase2('Illum_Retina_BXD_RankInv0410','ILMN_1224034','')) | [**0.571 79**](javascript:showCorrelationPlot2(db='Illum_Retina_BXD_RankInv0410',ProbeSetID='ILMN_1224034',CellID='',db2='Illum_Retina_BXD_RankInv0410',ProbeSetID2='ILMN_2639819',CellID2='',rank='1')) | [**0.472 79**](javascript:showCorrelationPlot2(db='Illum_Retina_BXD_RankInv0410',ProbeSetID='ILMN_1224034',CellID='',db2='Illum_Retina_BXD_RankInv0410',ProbeSetID2='ILMN_2994779',CellID2='',rank='1')) | [**0.620 79**](javascript:showCorrelationPlot2(db='Illum_Retina_BXD_RankInv0410',ProbeSetID='ILMN_1224034',CellID='',db2='Illum_Retina_BXD_RankInv0410',ProbeSetID2='ILMN_2625047',CellID2='',rank='1')) | [**0.676 79**](javascript:showCorrelationPlot2(db='Illum_Retina_BXD_RankInv0410',ProbeSetID='ILMN_1224034',CellID='',db2='Illum_Retina_BXD_RankInv0410',ProbeSetID2='ILMN_2808186',CellID2='',rank='1')) | [**0.431 79**](javascript:showCorrelationPlot2(db='Illum_Retina_BXD_RankInv0410',ProbeSetID='ILMN_1224034',CellID='',db2='Illum_Retina_BXD_RankInv0410',ProbeSetID2='ILMN_2689056',CellID2='',rank='1')) | [**0.409 79**](javascript:showCorrelationPlot2(db='Illum_Retina_BXD_RankInv0410',ProbeSetID='ILMN_1224034',CellID='',db2='Illum_Retina_BXD_RankInv0410',ProbeSetID2='ILMN_2648386',CellID2='',rank='1')) | [**0.356 79**](javascript:showCorrelationPlot2(db='Illum_Retina_BXD_RankInv0410',ProbeSetID='ILMN_1224034',CellID='',db2='Illum_Retina_BXD_RankInv0410',ProbeSetID2='ILMN_2869225',CellID2='',rank='1')) | [**0.514 79**](javascript:showCorrelationPlot2(db='Illum_Retina_BXD_RankInv0410',ProbeSetID='ILMN_1224034',CellID='',db2='Illum_Retina_BXD_RankInv0410',ProbeSetID2='ILMN_2728431',CellID2='',rank='1')) | [**0.418 79**](javascript:showCorrelationPlot2(db='Illum_Retina_BXD_RankInv0410',ProbeSetID='ILMN_1224034',CellID='',db2='Illum_Retina_BXD_RankInv0410',ProbeSetID2='ILMN_3094608',CellID2='',rank='1')) | [**0.344 79**](javascript:showCorrelationPlot2(db='Illum_Retina_BXD_RankInv0410',ProbeSetID='ILMN_1224034',CellID='',db2='Illum_Retina_BXD_RankInv0410',ProbeSetID2='ILMN_2896528',CellID2='',rank='1')) | [**0.588 79**](javascript:showCorrelationPlot2(db='Illum_Retina_BXD_RankInv0410',ProbeSetID='ILMN_1224034',CellID='',db2='Illum_Retina_BXD_RankInv0410',ProbeSetID2='ILMN_2701750',CellID2='',rank='1')) | [**0.348 79**](javascript:showCorrelationPlot2(db='Illum_Retina_BXD_RankInv0410',ProbeSetID='ILMN_1224034',CellID='',db2='Illum_Retina_BXD_RankInv0410',ProbeSetID2='ILMN_1239448',CellID2='',rank='1')) | [**-0.489 79**](javascript:showCorrelationPlot2(db='Illum_Retina_BXD_RankInv0410',ProbeSetID='ILMN_1224034',CellID='',db2='Illum_Retina_BXD_RankInv0410',ProbeSetID2='ILMN_2583882',CellID2='',rank='1')) | [**0.440 79**](javascript:showCorrelationPlot2(db='Illum_Retina_BXD_RankInv0410',ProbeSetID='ILMN_1224034',CellID='',db2='Illum_Retina_BXD_RankInv0410',ProbeSetID2='ILMN_2915166',CellID2='',rank='1')) | [**0.439 79**](javascript:showCorrelationPlot2(db='Illum_Retina_BXD_RankInv0410',ProbeSetID='ILMN_1224034',CellID='',db2='Illum_Retina_BXD_RankInv0410',ProbeSetID2='ILMN_2702997',CellID2='',rank='1')) | [**0.293 79**](javascript:showCorrelationPlot2(db='Illum_Retina_BXD_RankInv0410',ProbeSetID='ILMN_1224034',CellID='',db2='Illum_Retina_BXD_RankInv0410',ProbeSetID2='ILMN_2435206',CellID2='',rank='1')) | [**0.563 79**](javascript:showCorrelationPlot2(db='Illum_Retina_BXD_RankInv0410',ProbeSetID='ILMN_1224034',CellID='',db2='Illum_Retina_BXD_RankInv0410',ProbeSetID2='ILMN_2789888',CellID2='',rank='1')) | [**0.577 79**](javascript:showCorrelationPlot2(db='Illum_Retina_BXD_RankInv0410',ProbeSetID='ILMN_1224034',CellID='',db2='Illum_Retina_BXD_RankInv0410',ProbeSetID2='ILMN_1218901',CellID2='',rank='1')) | [**0.431 79**](javascript:showCorrelationPlot2(db='Illum_Retina_BXD_RankInv0410',ProbeSetID='ILMN_1224034',CellID='',db2='Illum_Retina_BXD_RankInv0410',ProbeSetID2='ILMN_1254734',CellID2='',rank='1')) | [**0.605 79**](javascript:showCorrelationPlot2(db='Illum_Retina_BXD_RankInv0410',ProbeSetID='ILMN_1224034',CellID='',db2='Illum_Retina_BXD_RankInv0410',ProbeSetID2='ILMN_3008924',CellID2='',rank='1')) | [**0.430 79**](javascript:showCorrelationPlot2(db='Illum_Retina_BXD_RankInv0410',ProbeSetID='ILMN_1224034',CellID='',db2='Illum_Retina_BXD_RankInv0410',ProbeSetID2='ILMN_2628175',CellID2='',rank='1')) | [**0.434 79**](javascript:showCorrelationPlot2(db='Illum_Retina_BXD_RankInv0410',ProbeSetID='ILMN_1224034',CellID='',db2='Illum_Retina_BXD_RankInv0410',ProbeSetID2='ILMN_2891573',CellID2='',rank='1')) | [**0.425 79**](javascript:showCorrelationPlot2(db='Illum_Retina_BXD_RankInv0410',ProbeSetID='ILMN_1224034',CellID='',db2='Illum_Retina_BXD_RankInv0410',ProbeSetID2='ILMN_1220846',CellID2='',rank='1')) | [**0.306 79**](javascript:showCorrelationPlot2(db='Illum_Retina_BXD_RankInv0410',ProbeSetID='ILMN_1224034',CellID='',db2='Illum_Retina_BXD_RankInv0410',ProbeSetID2='ILMN_1241962',CellID2='',rank='1')) | [**0.517 79**](javascript:showCorrelationPlot2(db='Illum_Retina_BXD_RankInv0410',ProbeSetID='ILMN_1224034',CellID='',db2='Illum_Retina_BXD_RankInv0410',ProbeSetID2='ILMN_3163027',CellID2='',rank='1')) | [**0.380 79**](javascript:showCorrelationPlot2(db='Illum_Retina_BXD_RankInv0410',ProbeSetID='ILMN_1224034',CellID='',db2='Illum_Retina_BXD_RankInv0410',ProbeSetID2='ILMN_2775098',CellID2='',rank='1')) | [**0.290 79**](javascript:showCorrelationPlot2(db='Illum_Retina_BXD_RankInv0410',ProbeSetID='ILMN_1224034',CellID='',db2='Illum_Retina_BXD_RankInv0410',ProbeSetID2='ILMN_2752552',CellID2='',rank='1')) | [**0.947 79**](javascript:showCorrelationPlot2(db='Illum_Retina_BXD_RankInv0410',ProbeSetID='ILMN_1224034',CellID='',db2='Illum_Retina_BXD_RankInv0410',ProbeSetID2='ILMN_2730293',CellID2='',rank='1')) | [**0.455 79**](javascript:showCorrelationPlot2(db='Illum_Retina_BXD_RankInv0410',ProbeSetID='ILMN_1224034',CellID='',db2='Illum_Retina_BXD_RankInv0410',ProbeSetID2='ILMN_2680872',CellID2='',rank='1')) | [**0.250 79**](javascript:showCorrelationPlot2(db='Illum_Retina_BXD_RankInv0410',ProbeSetID='ILMN_1224034',CellID='',db2='Illum_Retina_BXD_RankInv0410',ProbeSetID2='ILMN_2768533',CellID2='',rank='1')) | [**-0.096 79**](javascript:showCorrelationPlot2(db='Illum_Retina_BXD_RankInv0410',ProbeSetID='ILMN_1224034',CellID='',db2='Illum_Retina_BXD_RankInv0410',ProbeSetID2='ILMN_3121255',CellID2='',rank='1')) | [**-0.125 79**](javascript:showCorrelationPlot2(db='Illum_Retina_BXD_RankInv0410',ProbeSetID='ILMN_1224034',CellID='',db2='Illum_Retina_BXD_RankInv0410',ProbeSetID2='ILMN_2484527',CellID2='',rank='1')) | [**0.100 79**](javascript:showCorrelationPlot2(db='Illum_Retina_BXD_RankInv0410',ProbeSetID='ILMN_1224034',CellID='',db2='Illum_Retina_BXD_RankInv0410',ProbeSetID2='ILMN_3045723',CellID2='',rank='1')) | [**0.403 79**](javascript:showCorrelationPlot2(db='Illum_Retina_BXD_RankInv0410',ProbeSetID='ILMN_1224034',CellID='',db2='Illum_Retina_BXD_RankInv0410',ProbeSetID2='ILMN_2486573',CellID2='',rank='1')) |
|  | [**Trait 23: ILMN_2639819**](javascript:showDatabase2('Illum_Retina_BXD_RankInv0410','ILMN_2639819','');)  Symbol: Bet1l  Bet1l | [**0.661 79**](javascript:showCorrelationPlot2(db='Illum_Retina_BXD_RankInv0410',ProbeSetID='ILMN_2639819',CellID='',db2='Illum_Retina_BXD_RankInv0410',ProbeSetID2='ILMN_2450384',CellID2='',rank='0')) | [**0.687 79**](javascript:showCorrelationPlot2(db='Illum_Retina_BXD_RankInv0410',ProbeSetID='ILMN_2639819',CellID='',db2='Illum_Retina_BXD_RankInv0410',ProbeSetID2='ILMN_3068754',CellID2='',rank='0')) | [**0.684 79**](javascript:showCorrelationPlot2(db='Illum_Retina_BXD_RankInv0410',ProbeSetID='ILMN_2639819',CellID='',db2='Illum_Retina_BXD_RankInv0410',ProbeSetID2='ILMN_2677092',CellID2='',rank='0')) | [**0.600 79**](javascript:showCorrelationPlot2(db='Illum_Retina_BXD_RankInv0410',ProbeSetID='ILMN_2639819',CellID='',db2='Illum_Retina_BXD_RankInv0410',ProbeSetID2='ILMN_2624938',CellID2='',rank='0')) | [**0.565 79**](javascript:showCorrelationPlot2(db='Illum_Retina_BXD_RankInv0410',ProbeSetID='ILMN_2639819',CellID='',db2='Illum_Retina_BXD_RankInv0410',ProbeSetID2='ILMN_2847269',CellID2='',rank='0')) | [**0.463 79**](javascript:showCorrelationPlot2(db='Illum_Retina_BXD_RankInv0410',ProbeSetID='ILMN_2639819',CellID='',db2='Illum_Retina_BXD_RankInv0410',ProbeSetID2='ILMN_2839682',CellID2='',rank='0')) | [**0.563 79**](javascript:showCorrelationPlot2(db='Illum_Retina_BXD_RankInv0410',ProbeSetID='ILMN_2639819',CellID='',db2='Illum_Retina_BXD_RankInv0410',ProbeSetID2='ILMN_3147135',CellID2='',rank='0')) | [**0.552 79**](javascript:showCorrelationPlot2(db='Illum_Retina_BXD_RankInv0410',ProbeSetID='ILMN_2639819',CellID='',db2='Illum_Retina_BXD_RankInv0410',ProbeSetID2='ILMN_1255823',CellID2='',rank='0')) | [**0.657 79**](javascript:showCorrelationPlot2(db='Illum_Retina_BXD_RankInv0410',ProbeSetID='ILMN_2639819',CellID='',db2='Illum_Retina_BXD_RankInv0410',ProbeSetID2='ILMN_2626143',CellID2='',rank='0')) | [**0.433 79**](javascript:showCorrelationPlot2(db='Illum_Retina_BXD_RankInv0410',ProbeSetID='ILMN_2639819',CellID='',db2='Illum_Retina_BXD_RankInv0410',ProbeSetID2='ILMN_1234930',CellID2='',rank='0')) | [**0.620 79**](javascript:showCorrelationPlot2(db='Illum_Retina_BXD_RankInv0410',ProbeSetID='ILMN_2639819',CellID='',db2='Illum_Retina_BXD_RankInv0410',ProbeSetID2='ILMN_1249408',CellID2='',rank='0')) | [**0.385 79**](javascript:showCorrelationPlot2(db='Illum_Retina_BXD_RankInv0410',ProbeSetID='ILMN_2639819',CellID='',db2='Illum_Retina_BXD_RankInv0410',ProbeSetID2='ILMN_2971559',CellID2='',rank='0')) | [**0.496 79**](javascript:showCorrelationPlot2(db='Illum_Retina_BXD_RankInv0410',ProbeSetID='ILMN_2639819',CellID='',db2='Illum_Retina_BXD_RankInv0410',ProbeSetID2='ILMN_1258455',CellID2='',rank='0')) | [**0.532 79**](javascript:showCorrelationPlot2(db='Illum_Retina_BXD_RankInv0410',ProbeSetID='ILMN_2639819',CellID='',db2='Illum_Retina_BXD_RankInv0410',ProbeSetID2='ILMN_2691613',CellID2='',rank='0')) | [**0.473 79**](javascript:showCorrelationPlot2(db='Illum_Retina_BXD_RankInv0410',ProbeSetID='ILMN_2639819',CellID='',db2='Illum_Retina_BXD_RankInv0410',ProbeSetID2='ILMN_1253600',CellID2='',rank='0')) | [**0.483 79**](javascript:showCorrelationPlot2(db='Illum_Retina_BXD_RankInv0410',ProbeSetID='ILMN_2639819',CellID='',db2='Illum_Retina_BXD_RankInv0410',ProbeSetID2='ILMN_2991545',CellID2='',rank='0')) | [**0.495 79**](javascript:showCorrelationPlot2(db='Illum_Retina_BXD_RankInv0410',ProbeSetID='ILMN_2639819',CellID='',db2='Illum_Retina_BXD_RankInv0410',ProbeSetID2='ILMN_2623591',CellID2='',rank='0')) | [**0.507 79**](javascript:showCorrelationPlot2(db='Illum_Retina_BXD_RankInv0410',ProbeSetID='ILMN_2639819',CellID='',db2='Illum_Retina_BXD_RankInv0410',ProbeSetID2='ILMN_2603568',CellID2='',rank='0')) | [**0.643 79**](javascript:showCorrelationPlot2(db='Illum_Retina_BXD_RankInv0410',ProbeSetID='ILMN_2639819',CellID='',db2='Illum_Retina_BXD_RankInv0410',ProbeSetID2='ILMN_2992541',CellID2='',rank='0')) | [**0.513 79**](javascript:showCorrelationPlot2(db='Illum_Retina_BXD_RankInv0410',ProbeSetID='ILMN_2639819',CellID='',db2='Illum_Retina_BXD_RankInv0410',ProbeSetID2='ILMN_2792868',CellID2='',rank='0')) | [**0.522 79**](javascript:showCorrelationPlot2(db='Illum_Retina_BXD_RankInv0410',ProbeSetID='ILMN_2639819',CellID='',db2='Illum_Retina_BXD_RankInv0410',ProbeSetID2='ILMN_3129497',CellID2='',rank='0')) | [**0.523 79**](javascript:showCorrelationPlot2(db='Illum_Retina_BXD_RankInv0410',ProbeSetID='ILMN_2639819',CellID='',db2='Illum_Retina_BXD_RankInv0410',ProbeSetID2='ILMN_1224034',CellID2='',rank='0')) | [***n* 79**](javascript:showDatabase2('Illum_Retina_BXD_RankInv0410','ILMN_2639819','')) | [**0.521 79**](javascript:showCorrelationPlot2(db='Illum_Retina_BXD_RankInv0410',ProbeSetID='ILMN_2639819',CellID='',db2='Illum_Retina_BXD_RankInv0410',ProbeSetID2='ILMN_2994779',CellID2='',rank='1')) | [**0.466 79**](javascript:showCorrelationPlot2(db='Illum_Retina_BXD_RankInv0410',ProbeSetID='ILMN_2639819',CellID='',db2='Illum_Retina_BXD_RankInv0410',ProbeSetID2='ILMN_2625047',CellID2='',rank='1')) | [**0.551 79**](javascript:showCorrelationPlot2(db='Illum_Retina_BXD_RankInv0410',ProbeSetID='ILMN_2639819',CellID='',db2='Illum_Retina_BXD_RankInv0410',ProbeSetID2='ILMN_2808186',CellID2='',rank='1')) | [**0.535 79**](javascript:showCorrelationPlot2(db='Illum_Retina_BXD_RankInv0410',ProbeSetID='ILMN_2639819',CellID='',db2='Illum_Retina_BXD_RankInv0410',ProbeSetID2='ILMN_2689056',CellID2='',rank='1')) | [**0.695 79**](javascript:showCorrelationPlot2(db='Illum_Retina_BXD_RankInv0410',ProbeSetID='ILMN_2639819',CellID='',db2='Illum_Retina_BXD_RankInv0410',ProbeSetID2='ILMN_2648386',CellID2='',rank='1')) | [**0.542 79**](javascript:showCorrelationPlot2(db='Illum_Retina_BXD_RankInv0410',ProbeSetID='ILMN_2639819',CellID='',db2='Illum_Retina_BXD_RankInv0410',ProbeSetID2='ILMN_2869225',CellID2='',rank='1')) | [**0.459 79**](javascript:showCorrelationPlot2(db='Illum_Retina_BXD_RankInv0410',ProbeSetID='ILMN_2639819',CellID='',db2='Illum_Retina_BXD_RankInv0410',ProbeSetID2='ILMN_2728431',CellID2='',rank='1')) | [**0.581 79**](javascript:showCorrelationPlot2(db='Illum_Retina_BXD_RankInv0410',ProbeSetID='ILMN_2639819',CellID='',db2='Illum_Retina_BXD_RankInv0410',ProbeSetID2='ILMN_3094608',CellID2='',rank='1')) | [**0.480 79**](javascript:showCorrelationPlot2(db='Illum_Retina_BXD_RankInv0410',ProbeSetID='ILMN_2639819',CellID='',db2='Illum_Retina_BXD_RankInv0410',ProbeSetID2='ILMN_2896528',CellID2='',rank='1')) | [**0.575 79**](javascript:showCorrelationPlot2(db='Illum_Retina_BXD_RankInv0410',ProbeSetID='ILMN_2639819',CellID='',db2='Illum_Retina_BXD_RankInv0410',ProbeSetID2='ILMN_2701750',CellID2='',rank='1')) | [**0.471 79**](javascript:showCorrelationPlot2(db='Illum_Retina_BXD_RankInv0410',ProbeSetID='ILMN_2639819',CellID='',db2='Illum_Retina_BXD_RankInv0410',ProbeSetID2='ILMN_1239448',CellID2='',rank='1')) | [**-0.333 79**](javascript:showCorrelationPlot2(db='Illum_Retina_BXD_RankInv0410',ProbeSetID='ILMN_2639819',CellID='',db2='Illum_Retina_BXD_RankInv0410',ProbeSetID2='ILMN_2583882',CellID2='',rank='1')) | [**0.665 79**](javascript:showCorrelationPlot2(db='Illum_Retina_BXD_RankInv0410',ProbeSetID='ILMN_2639819',CellID='',db2='Illum_Retina_BXD_RankInv0410',ProbeSetID2='ILMN_2915166',CellID2='',rank='1')) | [**0.483 79**](javascript:showCorrelationPlot2(db='Illum_Retina_BXD_RankInv0410',ProbeSetID='ILMN_2639819',CellID='',db2='Illum_Retina_BXD_RankInv0410',ProbeSetID2='ILMN_2702997',CellID2='',rank='1')) | [**0.499 79**](javascript:showCorrelationPlot2(db='Illum_Retina_BXD_RankInv0410',ProbeSetID='ILMN_2639819',CellID='',db2='Illum_Retina_BXD_RankInv0410',ProbeSetID2='ILMN_2435206',CellID2='',rank='1')) | [**0.654 79**](javascript:showCorrelationPlot2(db='Illum_Retina_BXD_RankInv0410',ProbeSetID='ILMN_2639819',CellID='',db2='Illum_Retina_BXD_RankInv0410',ProbeSetID2='ILMN_2789888',CellID2='',rank='1')) | [**0.659 79**](javascript:showCorrelationPlot2(db='Illum_Retina_BXD_RankInv0410',ProbeSetID='ILMN_2639819',CellID='',db2='Illum_Retina_BXD_RankInv0410',ProbeSetID2='ILMN_1218901',CellID2='',rank='1')) | [**0.603 79**](javascript:showCorrelationPlot2(db='Illum_Retina_BXD_RankInv0410',ProbeSetID='ILMN_2639819',CellID='',db2='Illum_Retina_BXD_RankInv0410',ProbeSetID2='ILMN_1254734',CellID2='',rank='1')) | [**0.609 79**](javascript:showCorrelationPlot2(db='Illum_Retina_BXD_RankInv0410',ProbeSetID='ILMN_2639819',CellID='',db2='Illum_Retina_BXD_RankInv0410',ProbeSetID2='ILMN_3008924',CellID2='',rank='1')) | [**0.564 79**](javascript:showCorrelationPlot2(db='Illum_Retina_BXD_RankInv0410',ProbeSetID='ILMN_2639819',CellID='',db2='Illum_Retina_BXD_RankInv0410',ProbeSetID2='ILMN_2628175',CellID2='',rank='1')) | [**0.600 79**](javascript:showCorrelationPlot2(db='Illum_Retina_BXD_RankInv0410',ProbeSetID='ILMN_2639819',CellID='',db2='Illum_Retina_BXD_RankInv0410',ProbeSetID2='ILMN_2891573',CellID2='',rank='1')) | [**0.713 79**](javascript:showCorrelationPlot2(db='Illum_Retina_BXD_RankInv0410',ProbeSetID='ILMN_2639819',CellID='',db2='Illum_Retina_BXD_RankInv0410',ProbeSetID2='ILMN_1220846',CellID2='',rank='1')) | [**0.360 79**](javascript:showCorrelationPlot2(db='Illum_Retina_BXD_RankInv0410',ProbeSetID='ILMN_2639819',CellID='',db2='Illum_Retina_BXD_RankInv0410',ProbeSetID2='ILMN_1241962',CellID2='',rank='1')) | [**0.455 79**](javascript:showCorrelationPlot2(db='Illum_Retina_BXD_RankInv0410',ProbeSetID='ILMN_2639819',CellID='',db2='Illum_Retina_BXD_RankInv0410',ProbeSetID2='ILMN_3163027',CellID2='',rank='1')) | [**0.447 79**](javascript:showCorrelationPlot2(db='Illum_Retina_BXD_RankInv0410',ProbeSetID='ILMN_2639819',CellID='',db2='Illum_Retina_BXD_RankInv0410',ProbeSetID2='ILMN_2775098',CellID2='',rank='1')) | [**0.493 79**](javascript:showCorrelationPlot2(db='Illum_Retina_BXD_RankInv0410',ProbeSetID='ILMN_2639819',CellID='',db2='Illum_Retina_BXD_RankInv0410',ProbeSetID2='ILMN_2752552',CellID2='',rank='1')) | [**0.555 79**](javascript:showCorrelationPlot2(db='Illum_Retina_BXD_RankInv0410',ProbeSetID='ILMN_2639819',CellID='',db2='Illum_Retina_BXD_RankInv0410',ProbeSetID2='ILMN_2730293',CellID2='',rank='1')) | [**0.366 79**](javascript:showCorrelationPlot2(db='Illum_Retina_BXD_RankInv0410',ProbeSetID='ILMN_2639819',CellID='',db2='Illum_Retina_BXD_RankInv0410',ProbeSetID2='ILMN_2680872',CellID2='',rank='1')) | [**0.285 79**](javascript:showCorrelationPlot2(db='Illum_Retina_BXD_RankInv0410',ProbeSetID='ILMN_2639819',CellID='',db2='Illum_Retina_BXD_RankInv0410',ProbeSetID2='ILMN_2768533',CellID2='',rank='1')) | [**-0.367 79**](javascript:showCorrelationPlot2(db='Illum_Retina_BXD_RankInv0410',ProbeSetID='ILMN_2639819',CellID='',db2='Illum_Retina_BXD_RankInv0410',ProbeSetID2='ILMN_3121255',CellID2='',rank='1')) | [**-0.430 79**](javascript:showCorrelationPlot2(db='Illum_Retina_BXD_RankInv0410',ProbeSetID='ILMN_2639819',CellID='',db2='Illum_Retina_BXD_RankInv0410',ProbeSetID2='ILMN_2484527',CellID2='',rank='1')) | [**0.096 79**](javascript:showCorrelationPlot2(db='Illum_Retina_BXD_RankInv0410',ProbeSetID='ILMN_2639819',CellID='',db2='Illum_Retina_BXD_RankInv0410',ProbeSetID2='ILMN_3045723',CellID2='',rank='1')) | [**0.293 79**](javascript:showCorrelationPlot2(db='Illum_Retina_BXD_RankInv0410',ProbeSetID='ILMN_2639819',CellID='',db2='Illum_Retina_BXD_RankInv0410',ProbeSetID2='ILMN_2486573',CellID2='',rank='1')) |
|  | [**Trait 24: ILMN_2994779**](javascript:showDatabase2('Illum_Retina_BXD_RankInv0410','ILMN_2994779','');)  Symbol: Tecr  Tecr | [**0.660 79**](javascript:showCorrelationPlot2(db='Illum_Retina_BXD_RankInv0410',ProbeSetID='ILMN_2994779',CellID='',db2='Illum_Retina_BXD_RankInv0410',ProbeSetID2='ILMN_2450384',CellID2='',rank='0')) | [**0.512 79**](javascript:showCorrelationPlot2(db='Illum_Retina_BXD_RankInv0410',ProbeSetID='ILMN_2994779',CellID='',db2='Illum_Retina_BXD_RankInv0410',ProbeSetID2='ILMN_3068754',CellID2='',rank='0')) | [**0.473 79**](javascript:showCorrelationPlot2(db='Illum_Retina_BXD_RankInv0410',ProbeSetID='ILMN_2994779',CellID='',db2='Illum_Retina_BXD_RankInv0410',ProbeSetID2='ILMN_2677092',CellID2='',rank='0')) | [**0.599 79**](javascript:showCorrelationPlot2(db='Illum_Retina_BXD_RankInv0410',ProbeSetID='ILMN_2994779',CellID='',db2='Illum_Retina_BXD_RankInv0410',ProbeSetID2='ILMN_2624938',CellID2='',rank='0')) | [**0.650 79**](javascript:showCorrelationPlot2(db='Illum_Retina_BXD_RankInv0410',ProbeSetID='ILMN_2994779',CellID='',db2='Illum_Retina_BXD_RankInv0410',ProbeSetID2='ILMN_2847269',CellID2='',rank='0')) | [**0.492 79**](javascript:showCorrelationPlot2(db='Illum_Retina_BXD_RankInv0410',ProbeSetID='ILMN_2994779',CellID='',db2='Illum_Retina_BXD_RankInv0410',ProbeSetID2='ILMN_2839682',CellID2='',rank='0')) | [**0.670 79**](javascript:showCorrelationPlot2(db='Illum_Retina_BXD_RankInv0410',ProbeSetID='ILMN_2994779',CellID='',db2='Illum_Retina_BXD_RankInv0410',ProbeSetID2='ILMN_3147135',CellID2='',rank='0')) | [**0.543 79**](javascript:showCorrelationPlot2(db='Illum_Retina_BXD_RankInv0410',ProbeSetID='ILMN_2994779',CellID='',db2='Illum_Retina_BXD_RankInv0410',ProbeSetID2='ILMN_1255823',CellID2='',rank='0')) | [**0.639 79**](javascript:showCorrelationPlot2(db='Illum_Retina_BXD_RankInv0410',ProbeSetID='ILMN_2994779',CellID='',db2='Illum_Retina_BXD_RankInv0410',ProbeSetID2='ILMN_2626143',CellID2='',rank='0')) | [**0.573 79**](javascript:showCorrelationPlot2(db='Illum_Retina_BXD_RankInv0410',ProbeSetID='ILMN_2994779',CellID='',db2='Illum_Retina_BXD_RankInv0410',ProbeSetID2='ILMN_1234930',CellID2='',rank='0')) | [**0.566 79**](javascript:showCorrelationPlot2(db='Illum_Retina_BXD_RankInv0410',ProbeSetID='ILMN_2994779',CellID='',db2='Illum_Retina_BXD_RankInv0410',ProbeSetID2='ILMN_1249408',CellID2='',rank='0')) | [**0.605 79**](javascript:showCorrelationPlot2(db='Illum_Retina_BXD_RankInv0410',ProbeSetID='ILMN_2994779',CellID='',db2='Illum_Retina_BXD_RankInv0410',ProbeSetID2='ILMN_2971559',CellID2='',rank='0')) | [**0.341 79**](javascript:showCorrelationPlot2(db='Illum_Retina_BXD_RankInv0410',ProbeSetID='ILMN_2994779',CellID='',db2='Illum_Retina_BXD_RankInv0410',ProbeSetID2='ILMN_1258455',CellID2='',rank='0')) | [**0.666 79**](javascript:showCorrelationPlot2(db='Illum_Retina_BXD_RankInv0410',ProbeSetID='ILMN_2994779',CellID='',db2='Illum_Retina_BXD_RankInv0410',ProbeSetID2='ILMN_2691613',CellID2='',rank='0')) | [**0.400 79**](javascript:showCorrelationPlot2(db='Illum_Retina_BXD_RankInv0410',ProbeSetID='ILMN_2994779',CellID='',db2='Illum_Retina_BXD_RankInv0410',ProbeSetID2='ILMN_1253600',CellID2='',rank='0')) | [**0.596 79**](javascript:showCorrelationPlot2(db='Illum_Retina_BXD_RankInv0410',ProbeSetID='ILMN_2994779',CellID='',db2='Illum_Retina_BXD_RankInv0410',ProbeSetID2='ILMN_2991545',CellID2='',rank='0')) | [**0.428 79**](javascript:showCorrelationPlot2(db='Illum_Retina_BXD_RankInv0410',ProbeSetID='ILMN_2994779',CellID='',db2='Illum_Retina_BXD_RankInv0410',ProbeSetID2='ILMN_2623591',CellID2='',rank='0')) | [**0.277 79**](javascript:showCorrelationPlot2(db='Illum_Retina_BXD_RankInv0410',ProbeSetID='ILMN_2994779',CellID='',db2='Illum_Retina_BXD_RankInv0410',ProbeSetID2='ILMN_2603568',CellID2='',rank='0')) | [**0.716 79**](javascript:showCorrelationPlot2(db='Illum_Retina_BXD_RankInv0410',ProbeSetID='ILMN_2994779',CellID='',db2='Illum_Retina_BXD_RankInv0410',ProbeSetID2='ILMN_2992541',CellID2='',rank='0')) | [**0.469 79**](javascript:showCorrelationPlot2(db='Illum_Retina_BXD_RankInv0410',ProbeSetID='ILMN_2994779',CellID='',db2='Illum_Retina_BXD_RankInv0410',ProbeSetID2='ILMN_2792868',CellID2='',rank='0')) | [**0.462 79**](javascript:showCorrelationPlot2(db='Illum_Retina_BXD_RankInv0410',ProbeSetID='ILMN_2994779',CellID='',db2='Illum_Retina_BXD_RankInv0410',ProbeSetID2='ILMN_3129497',CellID2='',rank='0')) | [**0.506 79**](javascript:showCorrelationPlot2(db='Illum_Retina_BXD_RankInv0410',ProbeSetID='ILMN_2994779',CellID='',db2='Illum_Retina_BXD_RankInv0410',ProbeSetID2='ILMN_1224034',CellID2='',rank='0')) | [**0.537 79**](javascript:showCorrelationPlot2(db='Illum_Retina_BXD_RankInv0410',ProbeSetID='ILMN_2994779',CellID='',db2='Illum_Retina_BXD_RankInv0410',ProbeSetID2='ILMN_2639819',CellID2='',rank='0')) | [***n* 79**](javascript:showDatabase2('Illum_Retina_BXD_RankInv0410','ILMN_2994779','')) | [**0.479 79**](javascript:showCorrelationPlot2(db='Illum_Retina_BXD_RankInv0410',ProbeSetID='ILMN_2994779',CellID='',db2='Illum_Retina_BXD_RankInv0410',ProbeSetID2='ILMN_2625047',CellID2='',rank='1')) | [**0.301 79**](javascript:showCorrelationPlot2(db='Illum_Retina_BXD_RankInv0410',ProbeSetID='ILMN_2994779',CellID='',db2='Illum_Retina_BXD_RankInv0410',ProbeSetID2='ILMN_2808186',CellID2='',rank='1')) | [**0.422 79**](javascript:showCorrelationPlot2(db='Illum_Retina_BXD_RankInv0410',ProbeSetID='ILMN_2994779',CellID='',db2='Illum_Retina_BXD_RankInv0410',ProbeSetID2='ILMN_2689056',CellID2='',rank='1')) | [**0.529 79**](javascript:showCorrelationPlot2(db='Illum_Retina_BXD_RankInv0410',ProbeSetID='ILMN_2994779',CellID='',db2='Illum_Retina_BXD_RankInv0410',ProbeSetID2='ILMN_2648386',CellID2='',rank='1')) | [**0.627 79**](javascript:showCorrelationPlot2(db='Illum_Retina_BXD_RankInv0410',ProbeSetID='ILMN_2994779',CellID='',db2='Illum_Retina_BXD_RankInv0410',ProbeSetID2='ILMN_2869225',CellID2='',rank='1')) | [**0.495 79**](javascript:showCorrelationPlot2(db='Illum_Retina_BXD_RankInv0410',ProbeSetID='ILMN_2994779',CellID='',db2='Illum_Retina_BXD_RankInv0410',ProbeSetID2='ILMN_2728431',CellID2='',rank='1')) | [**0.349 79**](javascript:showCorrelationPlot2(db='Illum_Retina_BXD_RankInv0410',ProbeSetID='ILMN_2994779',CellID='',db2='Illum_Retina_BXD_RankInv0410',ProbeSetID2='ILMN_3094608',CellID2='',rank='1')) | [**0.594 79**](javascript:showCorrelationPlot2(db='Illum_Retina_BXD_RankInv0410',ProbeSetID='ILMN_2994779',CellID='',db2='Illum_Retina_BXD_RankInv0410',ProbeSetID2='ILMN_2896528',CellID2='',rank='1')) | [**0.303 79**](javascript:showCorrelationPlot2(db='Illum_Retina_BXD_RankInv0410',ProbeSetID='ILMN_2994779',CellID='',db2='Illum_Retina_BXD_RankInv0410',ProbeSetID2='ILMN_2701750',CellID2='',rank='1')) | [**0.341 79**](javascript:showCorrelationPlot2(db='Illum_Retina_BXD_RankInv0410',ProbeSetID='ILMN_2994779',CellID='',db2='Illum_Retina_BXD_RankInv0410',ProbeSetID2='ILMN_1239448',CellID2='',rank='1')) | [**-0.342 79**](javascript:showCorrelationPlot2(db='Illum_Retina_BXD_RankInv0410',ProbeSetID='ILMN_2994779',CellID='',db2='Illum_Retina_BXD_RankInv0410',ProbeSetID2='ILMN_2583882',CellID2='',rank='1')) | [**0.621 79**](javascript:showCorrelationPlot2(db='Illum_Retina_BXD_RankInv0410',ProbeSetID='ILMN_2994779',CellID='',db2='Illum_Retina_BXD_RankInv0410',ProbeSetID2='ILMN_2915166',CellID2='',rank='1')) | [**0.378 79**](javascript:showCorrelationPlot2(db='Illum_Retina_BXD_RankInv0410',ProbeSetID='ILMN_2994779',CellID='',db2='Illum_Retina_BXD_RankInv0410',ProbeSetID2='ILMN_2702997',CellID2='',rank='1')) | [**0.362 79**](javascript:showCorrelationPlot2(db='Illum_Retina_BXD_RankInv0410',ProbeSetID='ILMN_2994779',CellID='',db2='Illum_Retina_BXD_RankInv0410',ProbeSetID2='ILMN_2435206',CellID2='',rank='1')) | [**0.316 79**](javascript:showCorrelationPlot2(db='Illum_Retina_BXD_RankInv0410',ProbeSetID='ILMN_2994779',CellID='',db2='Illum_Retina_BXD_RankInv0410',ProbeSetID2='ILMN_2789888',CellID2='',rank='1')) | [**0.379 79**](javascript:showCorrelationPlot2(db='Illum_Retina_BXD_RankInv0410',ProbeSetID='ILMN_2994779',CellID='',db2='Illum_Retina_BXD_RankInv0410',ProbeSetID2='ILMN_1218901',CellID2='',rank='1')) | [**0.384 79**](javascript:showCorrelationPlot2(db='Illum_Retina_BXD_RankInv0410',ProbeSetID='ILMN_2994779',CellID='',db2='Illum_Retina_BXD_RankInv0410',ProbeSetID2='ILMN_1254734',CellID2='',rank='1')) | [**0.411 79**](javascript:showCorrelationPlot2(db='Illum_Retina_BXD_RankInv0410',ProbeSetID='ILMN_2994779',CellID='',db2='Illum_Retina_BXD_RankInv0410',ProbeSetID2='ILMN_3008924',CellID2='',rank='1')) | [**0.359 79**](javascript:showCorrelationPlot2(db='Illum_Retina_BXD_RankInv0410',ProbeSetID='ILMN_2994779',CellID='',db2='Illum_Retina_BXD_RankInv0410',ProbeSetID2='ILMN_2628175',CellID2='',rank='1')) | [**0.607 79**](javascript:showCorrelationPlot2(db='Illum_Retina_BXD_RankInv0410',ProbeSetID='ILMN_2994779',CellID='',db2='Illum_Retina_BXD_RankInv0410',ProbeSetID2='ILMN_2891573',CellID2='',rank='1')) | [**0.355 79**](javascript:showCorrelationPlot2(db='Illum_Retina_BXD_RankInv0410',ProbeSetID='ILMN_2994779',CellID='',db2='Illum_Retina_BXD_RankInv0410',ProbeSetID2='ILMN_1220846',CellID2='',rank='1')) | [**0.516 79**](javascript:showCorrelationPlot2(db='Illum_Retina_BXD_RankInv0410',ProbeSetID='ILMN_2994779',CellID='',db2='Illum_Retina_BXD_RankInv0410',ProbeSetID2='ILMN_1241962',CellID2='',rank='1')) | [**0.624 79**](javascript:showCorrelationPlot2(db='Illum_Retina_BXD_RankInv0410',ProbeSetID='ILMN_2994779',CellID='',db2='Illum_Retina_BXD_RankInv0410',ProbeSetID2='ILMN_3163027',CellID2='',rank='1')) | [**0.308 79**](javascript:showCorrelationPlot2(db='Illum_Retina_BXD_RankInv0410',ProbeSetID='ILMN_2994779',CellID='',db2='Illum_Retina_BXD_RankInv0410',ProbeSetID2='ILMN_2775098',CellID2='',rank='1')) | [**0.546 79**](javascript:showCorrelationPlot2(db='Illum_Retina_BXD_RankInv0410',ProbeSetID='ILMN_2994779',CellID='',db2='Illum_Retina_BXD_RankInv0410',ProbeSetID2='ILMN_2752552',CellID2='',rank='1')) | [**0.464 79**](javascript:showCorrelationPlot2(db='Illum_Retina_BXD_RankInv0410',ProbeSetID='ILMN_2994779',CellID='',db2='Illum_Retina_BXD_RankInv0410',ProbeSetID2='ILMN_2730293',CellID2='',rank='1')) | [**0.463 79**](javascript:showCorrelationPlot2(db='Illum_Retina_BXD_RankInv0410',ProbeSetID='ILMN_2994779',CellID='',db2='Illum_Retina_BXD_RankInv0410',ProbeSetID2='ILMN_2680872',CellID2='',rank='1')) | [**0.183 79**](javascript:showCorrelationPlot2(db='Illum_Retina_BXD_RankInv0410',ProbeSetID='ILMN_2994779',CellID='',db2='Illum_Retina_BXD_RankInv0410',ProbeSetID2='ILMN_2768533',CellID2='',rank='1')) | [**0.069 79**](javascript:showCorrelationPlot2(db='Illum_Retina_BXD_RankInv0410',ProbeSetID='ILMN_2994779',CellID='',db2='Illum_Retina_BXD_RankInv0410',ProbeSetID2='ILMN_3121255',CellID2='',rank='1')) | [**-0.066 79**](javascript:showCorrelationPlot2(db='Illum_Retina_BXD_RankInv0410',ProbeSetID='ILMN_2994779',CellID='',db2='Illum_Retina_BXD_RankInv0410',ProbeSetID2='ILMN_2484527',CellID2='',rank='1')) | [**0.280 79**](javascript:showCorrelationPlot2(db='Illum_Retina_BXD_RankInv0410',ProbeSetID='ILMN_2994779',CellID='',db2='Illum_Retina_BXD_RankInv0410',ProbeSetID2='ILMN_3045723',CellID2='',rank='1')) | [**0.142 79**](javascript:showCorrelationPlot2(db='Illum_Retina_BXD_RankInv0410',ProbeSetID='ILMN_2994779',CellID='',db2='Illum_Retina_BXD_RankInv0410',ProbeSetID2='ILMN_2486573',CellID2='',rank='1')) |
|  | [**Trait 25: ILMN_2625047**](javascript:showDatabase2('Illum_Retina_BXD_RankInv0410','ILMN_2625047','');)  Symbol: C430004E15Rik  C430004E15Rik | [**0.660 79**](javascript:showCorrelationPlot2(db='Illum_Retina_BXD_RankInv0410',ProbeSetID='ILMN_2625047',CellID='',db2='Illum_Retina_BXD_RankInv0410',ProbeSetID2='ILMN_2450384',CellID2='',rank='0')) | [**0.510 79**](javascript:showCorrelationPlot2(db='Illum_Retina_BXD_RankInv0410',ProbeSetID='ILMN_2625047',CellID='',db2='Illum_Retina_BXD_RankInv0410',ProbeSetID2='ILMN_3068754',CellID2='',rank='0')) | [**0.470 79**](javascript:showCorrelationPlot2(db='Illum_Retina_BXD_RankInv0410',ProbeSetID='ILMN_2625047',CellID='',db2='Illum_Retina_BXD_RankInv0410',ProbeSetID2='ILMN_2677092',CellID2='',rank='0')) | [**0.647 79**](javascript:showCorrelationPlot2(db='Illum_Retina_BXD_RankInv0410',ProbeSetID='ILMN_2625047',CellID='',db2='Illum_Retina_BXD_RankInv0410',ProbeSetID2='ILMN_2624938',CellID2='',rank='0')) | [**0.595 79**](javascript:showCorrelationPlot2(db='Illum_Retina_BXD_RankInv0410',ProbeSetID='ILMN_2625047',CellID='',db2='Illum_Retina_BXD_RankInv0410',ProbeSetID2='ILMN_2847269',CellID2='',rank='0')) | [**0.644 79**](javascript:showCorrelationPlot2(db='Illum_Retina_BXD_RankInv0410',ProbeSetID='ILMN_2625047',CellID='',db2='Illum_Retina_BXD_RankInv0410',ProbeSetID2='ILMN_2839682',CellID2='',rank='0')) | [**0.635 79**](javascript:showCorrelationPlot2(db='Illum_Retina_BXD_RankInv0410',ProbeSetID='ILMN_2625047',CellID='',db2='Illum_Retina_BXD_RankInv0410',ProbeSetID2='ILMN_3147135',CellID2='',rank='0')) | [**0.472 79**](javascript:showCorrelationPlot2(db='Illum_Retina_BXD_RankInv0410',ProbeSetID='ILMN_2625047',CellID='',db2='Illum_Retina_BXD_RankInv0410',ProbeSetID2='ILMN_1255823',CellID2='',rank='0')) | [**0.464 79**](javascript:showCorrelationPlot2(db='Illum_Retina_BXD_RankInv0410',ProbeSetID='ILMN_2625047',CellID='',db2='Illum_Retina_BXD_RankInv0410',ProbeSetID2='ILMN_2626143',CellID2='',rank='0')) | [**0.670 79**](javascript:showCorrelationPlot2(db='Illum_Retina_BXD_RankInv0410',ProbeSetID='ILMN_2625047',CellID='',db2='Illum_Retina_BXD_RankInv0410',ProbeSetID2='ILMN_1234930',CellID2='',rank='0')) | [**0.505 79**](javascript:showCorrelationPlot2(db='Illum_Retina_BXD_RankInv0410',ProbeSetID='ILMN_2625047',CellID='',db2='Illum_Retina_BXD_RankInv0410',ProbeSetID2='ILMN_1249408',CellID2='',rank='0')) | [**0.617 79**](javascript:showCorrelationPlot2(db='Illum_Retina_BXD_RankInv0410',ProbeSetID='ILMN_2625047',CellID='',db2='Illum_Retina_BXD_RankInv0410',ProbeSetID2='ILMN_2971559',CellID2='',rank='0')) | [**0.475 79**](javascript:showCorrelationPlot2(db='Illum_Retina_BXD_RankInv0410',ProbeSetID='ILMN_2625047',CellID='',db2='Illum_Retina_BXD_RankInv0410',ProbeSetID2='ILMN_1258455',CellID2='',rank='0')) | [**0.559 79**](javascript:showCorrelationPlot2(db='Illum_Retina_BXD_RankInv0410',ProbeSetID='ILMN_2625047',CellID='',db2='Illum_Retina_BXD_RankInv0410',ProbeSetID2='ILMN_2691613',CellID2='',rank='0')) | [**0.542 79**](javascript:showCorrelationPlot2(db='Illum_Retina_BXD_RankInv0410',ProbeSetID='ILMN_2625047',CellID='',db2='Illum_Retina_BXD_RankInv0410',ProbeSetID2='ILMN_1253600',CellID2='',rank='0')) | [**0.479 79**](javascript:showCorrelationPlot2(db='Illum_Retina_BXD_RankInv0410',ProbeSetID='ILMN_2625047',CellID='',db2='Illum_Retina_BXD_RankInv0410',ProbeSetID2='ILMN_2991545',CellID2='',rank='0')) | [**0.467 79**](javascript:showCorrelationPlot2(db='Illum_Retina_BXD_RankInv0410',ProbeSetID='ILMN_2625047',CellID='',db2='Illum_Retina_BXD_RankInv0410',ProbeSetID2='ILMN_2623591',CellID2='',rank='0')) | [**0.349 79**](javascript:showCorrelationPlot2(db='Illum_Retina_BXD_RankInv0410',ProbeSetID='ILMN_2625047',CellID='',db2='Illum_Retina_BXD_RankInv0410',ProbeSetID2='ILMN_2603568',CellID2='',rank='0')) | [**0.600 79**](javascript:showCorrelationPlot2(db='Illum_Retina_BXD_RankInv0410',ProbeSetID='ILMN_2625047',CellID='',db2='Illum_Retina_BXD_RankInv0410',ProbeSetID2='ILMN_2992541',CellID2='',rank='0')) | [**0.594 79**](javascript:showCorrelationPlot2(db='Illum_Retina_BXD_RankInv0410',ProbeSetID='ILMN_2625047',CellID='',db2='Illum_Retina_BXD_RankInv0410',ProbeSetID2='ILMN_2792868',CellID2='',rank='0')) | [**0.582 79**](javascript:showCorrelationPlot2(db='Illum_Retina_BXD_RankInv0410',ProbeSetID='ILMN_2625047',CellID='',db2='Illum_Retina_BXD_RankInv0410',ProbeSetID2='ILMN_3129497',CellID2='',rank='0')) | [**0.668 79**](javascript:showCorrelationPlot2(db='Illum_Retina_BXD_RankInv0410',ProbeSetID='ILMN_2625047',CellID='',db2='Illum_Retina_BXD_RankInv0410',ProbeSetID2='ILMN_1224034',CellID2='',rank='0')) | [**0.425 79**](javascript:showCorrelationPlot2(db='Illum_Retina_BXD_RankInv0410',ProbeSetID='ILMN_2625047',CellID='',db2='Illum_Retina_BXD_RankInv0410',ProbeSetID2='ILMN_2639819',CellID2='',rank='0')) | [**0.521 79**](javascript:showCorrelationPlot2(db='Illum_Retina_BXD_RankInv0410',ProbeSetID='ILMN_2625047',CellID='',db2='Illum_Retina_BXD_RankInv0410',ProbeSetID2='ILMN_2994779',CellID2='',rank='0')) | [***n* 79**](javascript:showDatabase2('Illum_Retina_BXD_RankInv0410','ILMN_2625047','')) | [**0.372 79**](javascript:showCorrelationPlot2(db='Illum_Retina_BXD_RankInv0410',ProbeSetID='ILMN_2625047',CellID='',db2='Illum_Retina_BXD_RankInv0410',ProbeSetID2='ILMN_2808186',CellID2='',rank='1')) | [**0.436 79**](javascript:showCorrelationPlot2(db='Illum_Retina_BXD_RankInv0410',ProbeSetID='ILMN_2625047',CellID='',db2='Illum_Retina_BXD_RankInv0410',ProbeSetID2='ILMN_2689056',CellID2='',rank='1')) | [**0.444 79**](javascript:showCorrelationPlot2(db='Illum_Retina_BXD_RankInv0410',ProbeSetID='ILMN_2625047',CellID='',db2='Illum_Retina_BXD_RankInv0410',ProbeSetID2='ILMN_2648386',CellID2='',rank='1')) | [**0.494 79**](javascript:showCorrelationPlot2(db='Illum_Retina_BXD_RankInv0410',ProbeSetID='ILMN_2625047',CellID='',db2='Illum_Retina_BXD_RankInv0410',ProbeSetID2='ILMN_2869225',CellID2='',rank='1')) | [**0.522 79**](javascript:showCorrelationPlot2(db='Illum_Retina_BXD_RankInv0410',ProbeSetID='ILMN_2625047',CellID='',db2='Illum_Retina_BXD_RankInv0410',ProbeSetID2='ILMN_2728431',CellID2='',rank='1')) | [**0.478 79**](javascript:showCorrelationPlot2(db='Illum_Retina_BXD_RankInv0410',ProbeSetID='ILMN_2625047',CellID='',db2='Illum_Retina_BXD_RankInv0410',ProbeSetID2='ILMN_3094608',CellID2='',rank='1')) | [**0.591 79**](javascript:showCorrelationPlot2(db='Illum_Retina_BXD_RankInv0410',ProbeSetID='ILMN_2625047',CellID='',db2='Illum_Retina_BXD_RankInv0410',ProbeSetID2='ILMN_2896528',CellID2='',rank='1')) | [**0.411 79**](javascript:showCorrelationPlot2(db='Illum_Retina_BXD_RankInv0410',ProbeSetID='ILMN_2625047',CellID='',db2='Illum_Retina_BXD_RankInv0410',ProbeSetID2='ILMN_2701750',CellID2='',rank='1')) | [**0.530 79**](javascript:showCorrelationPlot2(db='Illum_Retina_BXD_RankInv0410',ProbeSetID='ILMN_2625047',CellID='',db2='Illum_Retina_BXD_RankInv0410',ProbeSetID2='ILMN_1239448',CellID2='',rank='1')) | [**-0.381 79**](javascript:showCorrelationPlot2(db='Illum_Retina_BXD_RankInv0410',ProbeSetID='ILMN_2625047',CellID='',db2='Illum_Retina_BXD_RankInv0410',ProbeSetID2='ILMN_2583882',CellID2='',rank='1')) | [**0.404 79**](javascript:showCorrelationPlot2(db='Illum_Retina_BXD_RankInv0410',ProbeSetID='ILMN_2625047',CellID='',db2='Illum_Retina_BXD_RankInv0410',ProbeSetID2='ILMN_2915166',CellID2='',rank='1')) | [**0.464 79**](javascript:showCorrelationPlot2(db='Illum_Retina_BXD_RankInv0410',ProbeSetID='ILMN_2625047',CellID='',db2='Illum_Retina_BXD_RankInv0410',ProbeSetID2='ILMN_2702997',CellID2='',rank='1')) | [**0.394 79**](javascript:showCorrelationPlot2(db='Illum_Retina_BXD_RankInv0410',ProbeSetID='ILMN_2625047',CellID='',db2='Illum_Retina_BXD_RankInv0410',ProbeSetID2='ILMN_2435206',CellID2='',rank='1')) | [**0.478 79**](javascript:showCorrelationPlot2(db='Illum_Retina_BXD_RankInv0410',ProbeSetID='ILMN_2625047',CellID='',db2='Illum_Retina_BXD_RankInv0410',ProbeSetID2='ILMN_2789888',CellID2='',rank='1')) | [**0.441 79**](javascript:showCorrelationPlot2(db='Illum_Retina_BXD_RankInv0410',ProbeSetID='ILMN_2625047',CellID='',db2='Illum_Retina_BXD_RankInv0410',ProbeSetID2='ILMN_1218901',CellID2='',rank='1')) | [**0.291 79**](javascript:showCorrelationPlot2(db='Illum_Retina_BXD_RankInv0410',ProbeSetID='ILMN_2625047',CellID='',db2='Illum_Retina_BXD_RankInv0410',ProbeSetID2='ILMN_1254734',CellID2='',rank='1')) | [**0.538 79**](javascript:showCorrelationPlot2(db='Illum_Retina_BXD_RankInv0410',ProbeSetID='ILMN_2625047',CellID='',db2='Illum_Retina_BXD_RankInv0410',ProbeSetID2='ILMN_3008924',CellID2='',rank='1')) | [**0.413 79**](javascript:showCorrelationPlot2(db='Illum_Retina_BXD_RankInv0410',ProbeSetID='ILMN_2625047',CellID='',db2='Illum_Retina_BXD_RankInv0410',ProbeSetID2='ILMN_2628175',CellID2='',rank='1')) | [**0.452 79**](javascript:showCorrelationPlot2(db='Illum_Retina_BXD_RankInv0410',ProbeSetID='ILMN_2625047',CellID='',db2='Illum_Retina_BXD_RankInv0410',ProbeSetID2='ILMN_2891573',CellID2='',rank='1')) | [**0.496 79**](javascript:showCorrelationPlot2(db='Illum_Retina_BXD_RankInv0410',ProbeSetID='ILMN_2625047',CellID='',db2='Illum_Retina_BXD_RankInv0410',ProbeSetID2='ILMN_1220846',CellID2='',rank='1')) | [**0.516 79**](javascript:showCorrelationPlot2(db='Illum_Retina_BXD_RankInv0410',ProbeSetID='ILMN_2625047',CellID='',db2='Illum_Retina_BXD_RankInv0410',ProbeSetID2='ILMN_1241962',CellID2='',rank='1')) | [**0.502 79**](javascript:showCorrelationPlot2(db='Illum_Retina_BXD_RankInv0410',ProbeSetID='ILMN_2625047',CellID='',db2='Illum_Retina_BXD_RankInv0410',ProbeSetID2='ILMN_3163027',CellID2='',rank='1')) | [**0.245 79**](javascript:showCorrelationPlot2(db='Illum_Retina_BXD_RankInv0410',ProbeSetID='ILMN_2625047',CellID='',db2='Illum_Retina_BXD_RankInv0410',ProbeSetID2='ILMN_2775098',CellID2='',rank='1')) | [**0.533 79**](javascript:showCorrelationPlot2(db='Illum_Retina_BXD_RankInv0410',ProbeSetID='ILMN_2625047',CellID='',db2='Illum_Retina_BXD_RankInv0410',ProbeSetID2='ILMN_2752552',CellID2='',rank='1')) | [**0.602 79**](javascript:showCorrelationPlot2(db='Illum_Retina_BXD_RankInv0410',ProbeSetID='ILMN_2625047',CellID='',db2='Illum_Retina_BXD_RankInv0410',ProbeSetID2='ILMN_2730293',CellID2='',rank='1')) | [**0.523 79**](javascript:showCorrelationPlot2(db='Illum_Retina_BXD_RankInv0410',ProbeSetID='ILMN_2625047',CellID='',db2='Illum_Retina_BXD_RankInv0410',ProbeSetID2='ILMN_2680872',CellID2='',rank='1')) | [**0.258 79**](javascript:showCorrelationPlot2(db='Illum_Retina_BXD_RankInv0410',ProbeSetID='ILMN_2625047',CellID='',db2='Illum_Retina_BXD_RankInv0410',ProbeSetID2='ILMN_2768533',CellID2='',rank='1')) | [**-0.084 79**](javascript:showCorrelationPlot2(db='Illum_Retina_BXD_RankInv0410',ProbeSetID='ILMN_2625047',CellID='',db2='Illum_Retina_BXD_RankInv0410',ProbeSetID2='ILMN_3121255',CellID2='',rank='1')) | [**-0.156 79**](javascript:showCorrelationPlot2(db='Illum_Retina_BXD_RankInv0410',ProbeSetID='ILMN_2625047',CellID='',db2='Illum_Retina_BXD_RankInv0410',ProbeSetID2='ILMN_2484527',CellID2='',rank='1')) | [**0.181 79**](javascript:showCorrelationPlot2(db='Illum_Retina_BXD_RankInv0410',ProbeSetID='ILMN_2625047',CellID='',db2='Illum_Retina_BXD_RankInv0410',ProbeSetID2='ILMN_3045723',CellID2='',rank='1')) | [**0.170 79**](javascript:showCorrelationPlot2(db='Illum_Retina_BXD_RankInv0410',ProbeSetID='ILMN_2625047',CellID='',db2='Illum_Retina_BXD_RankInv0410',ProbeSetID2='ILMN_2486573',CellID2='',rank='1')) |
|  | [**Trait 26: ILMN_2808186**](javascript:showDatabase2('Illum_Retina_BXD_RankInv0410','ILMN_2808186','');)  Symbol: Gtf2ird2  Gtf2ird2 | [**0.653 79**](javascript:showCorrelationPlot2(db='Illum_Retina_BXD_RankInv0410',ProbeSetID='ILMN_2808186',CellID='',db2='Illum_Retina_BXD_RankInv0410',ProbeSetID2='ILMN_2450384',CellID2='',rank='0')) | [**0.648 79**](javascript:showCorrelationPlot2(db='Illum_Retina_BXD_RankInv0410',ProbeSetID='ILMN_2808186',CellID='',db2='Illum_Retina_BXD_RankInv0410',ProbeSetID2='ILMN_3068754',CellID2='',rank='0')) | [**0.738 79**](javascript:showCorrelationPlot2(db='Illum_Retina_BXD_RankInv0410',ProbeSetID='ILMN_2808186',CellID='',db2='Illum_Retina_BXD_RankInv0410',ProbeSetID2='ILMN_2677092',CellID2='',rank='0')) | [**0.524 79**](javascript:showCorrelationPlot2(db='Illum_Retina_BXD_RankInv0410',ProbeSetID='ILMN_2808186',CellID='',db2='Illum_Retina_BXD_RankInv0410',ProbeSetID2='ILMN_2624938',CellID2='',rank='0')) | [**0.424 79**](javascript:showCorrelationPlot2(db='Illum_Retina_BXD_RankInv0410',ProbeSetID='ILMN_2808186',CellID='',db2='Illum_Retina_BXD_RankInv0410',ProbeSetID2='ILMN_2847269',CellID2='',rank='0')) | [**0.567 79**](javascript:showCorrelationPlot2(db='Illum_Retina_BXD_RankInv0410',ProbeSetID='ILMN_2808186',CellID='',db2='Illum_Retina_BXD_RankInv0410',ProbeSetID2='ILMN_2839682',CellID2='',rank='0')) | [**0.407 79**](javascript:showCorrelationPlot2(db='Illum_Retina_BXD_RankInv0410',ProbeSetID='ILMN_2808186',CellID='',db2='Illum_Retina_BXD_RankInv0410',ProbeSetID2='ILMN_3147135',CellID2='',rank='0')) | [**0.324 79**](javascript:showCorrelationPlot2(db='Illum_Retina_BXD_RankInv0410',ProbeSetID='ILMN_2808186',CellID='',db2='Illum_Retina_BXD_RankInv0410',ProbeSetID2='ILMN_1255823',CellID2='',rank='0')) | [**0.500 79**](javascript:showCorrelationPlot2(db='Illum_Retina_BXD_RankInv0410',ProbeSetID='ILMN_2808186',CellID='',db2='Illum_Retina_BXD_RankInv0410',ProbeSetID2='ILMN_2626143',CellID2='',rank='0')) | [**0.472 79**](javascript:showCorrelationPlot2(db='Illum_Retina_BXD_RankInv0410',ProbeSetID='ILMN_2808186',CellID='',db2='Illum_Retina_BXD_RankInv0410',ProbeSetID2='ILMN_1234930',CellID2='',rank='0')) | [**0.298 79**](javascript:showCorrelationPlot2(db='Illum_Retina_BXD_RankInv0410',ProbeSetID='ILMN_2808186',CellID='',db2='Illum_Retina_BXD_RankInv0410',ProbeSetID2='ILMN_1249408',CellID2='',rank='0')) | [**0.517 79**](javascript:showCorrelationPlot2(db='Illum_Retina_BXD_RankInv0410',ProbeSetID='ILMN_2808186',CellID='',db2='Illum_Retina_BXD_RankInv0410',ProbeSetID2='ILMN_2971559',CellID2='',rank='0')) | [**0.698 79**](javascript:showCorrelationPlot2(db='Illum_Retina_BXD_RankInv0410',ProbeSetID='ILMN_2808186',CellID='',db2='Illum_Retina_BXD_RankInv0410',ProbeSetID2='ILMN_1258455',CellID2='',rank='0')) | [**0.329 79**](javascript:showCorrelationPlot2(db='Illum_Retina_BXD_RankInv0410',ProbeSetID='ILMN_2808186',CellID='',db2='Illum_Retina_BXD_RankInv0410',ProbeSetID2='ILMN_2691613',CellID2='',rank='0')) | [**0.635 79**](javascript:showCorrelationPlot2(db='Illum_Retina_BXD_RankInv0410',ProbeSetID='ILMN_2808186',CellID='',db2='Illum_Retina_BXD_RankInv0410',ProbeSetID2='ILMN_1253600',CellID2='',rank='0')) | [**0.423 79**](javascript:showCorrelationPlot2(db='Illum_Retina_BXD_RankInv0410',ProbeSetID='ILMN_2808186',CellID='',db2='Illum_Retina_BXD_RankInv0410',ProbeSetID2='ILMN_2991545',CellID2='',rank='0')) | [**0.618 79**](javascript:showCorrelationPlot2(db='Illum_Retina_BXD_RankInv0410',ProbeSetID='ILMN_2808186',CellID='',db2='Illum_Retina_BXD_RankInv0410',ProbeSetID2='ILMN_2623591',CellID2='',rank='0')) | [**0.428 79**](javascript:showCorrelationPlot2(db='Illum_Retina_BXD_RankInv0410',ProbeSetID='ILMN_2808186',CellID='',db2='Illum_Retina_BXD_RankInv0410',ProbeSetID2='ILMN_2603568',CellID2='',rank='0')) | [**0.366 79**](javascript:showCorrelationPlot2(db='Illum_Retina_BXD_RankInv0410',ProbeSetID='ILMN_2808186',CellID='',db2='Illum_Retina_BXD_RankInv0410',ProbeSetID2='ILMN_2992541',CellID2='',rank='0')) | [**0.242 79**](javascript:showCorrelationPlot2(db='Illum_Retina_BXD_RankInv0410',ProbeSetID='ILMN_2808186',CellID='',db2='Illum_Retina_BXD_RankInv0410',ProbeSetID2='ILMN_2792868',CellID2='',rank='0')) | [**0.541 79**](javascript:showCorrelationPlot2(db='Illum_Retina_BXD_RankInv0410',ProbeSetID='ILMN_2808186',CellID='',db2='Illum_Retina_BXD_RankInv0410',ProbeSetID2='ILMN_3129497',CellID2='',rank='0')) | [**0.614 79**](javascript:showCorrelationPlot2(db='Illum_Retina_BXD_RankInv0410',ProbeSetID='ILMN_2808186',CellID='',db2='Illum_Retina_BXD_RankInv0410',ProbeSetID2='ILMN_1224034',CellID2='',rank='0')) | [**0.569 79**](javascript:showCorrelationPlot2(db='Illum_Retina_BXD_RankInv0410',ProbeSetID='ILMN_2808186',CellID='',db2='Illum_Retina_BXD_RankInv0410',ProbeSetID2='ILMN_2639819',CellID2='',rank='0')) | [**0.345 79**](javascript:showCorrelationPlot2(db='Illum_Retina_BXD_RankInv0410',ProbeSetID='ILMN_2808186',CellID='',db2='Illum_Retina_BXD_RankInv0410',ProbeSetID2='ILMN_2994779',CellID2='',rank='0')) | [**0.388 79**](javascript:showCorrelationPlot2(db='Illum_Retina_BXD_RankInv0410',ProbeSetID='ILMN_2808186',CellID='',db2='Illum_Retina_BXD_RankInv0410',ProbeSetID2='ILMN_2625047',CellID2='',rank='0')) | [***n* 79**](javascript:showDatabase2('Illum_Retina_BXD_RankInv0410','ILMN_2808186','')) | [**0.577 79**](javascript:showCorrelationPlot2(db='Illum_Retina_BXD_RankInv0410',ProbeSetID='ILMN_2808186',CellID='',db2='Illum_Retina_BXD_RankInv0410',ProbeSetID2='ILMN_2689056',CellID2='',rank='1')) | [**0.302 79**](javascript:showCorrelationPlot2(db='Illum_Retina_BXD_RankInv0410',ProbeSetID='ILMN_2808186',CellID='',db2='Illum_Retina_BXD_RankInv0410',ProbeSetID2='ILMN_2648386',CellID2='',rank='1')) | [**0.269 79**](javascript:showCorrelationPlot2(db='Illum_Retina_BXD_RankInv0410',ProbeSetID='ILMN_2808186',CellID='',db2='Illum_Retina_BXD_RankInv0410',ProbeSetID2='ILMN_2869225',CellID2='',rank='1')) | [**0.491 79**](javascript:showCorrelationPlot2(db='Illum_Retina_BXD_RankInv0410',ProbeSetID='ILMN_2808186',CellID='',db2='Illum_Retina_BXD_RankInv0410',ProbeSetID2='ILMN_2728431',CellID2='',rank='1')) | [**0.556 79**](javascript:showCorrelationPlot2(db='Illum_Retina_BXD_RankInv0410',ProbeSetID='ILMN_2808186',CellID='',db2='Illum_Retina_BXD_RankInv0410',ProbeSetID2='ILMN_3094608',CellID2='',rank='1')) | [**0.225 79**](javascript:showCorrelationPlot2(db='Illum_Retina_BXD_RankInv0410',ProbeSetID='ILMN_2808186',CellID='',db2='Illum_Retina_BXD_RankInv0410',ProbeSetID2='ILMN_2896528',CellID2='',rank='1')) | [**0.572 79**](javascript:showCorrelationPlot2(db='Illum_Retina_BXD_RankInv0410',ProbeSetID='ILMN_2808186',CellID='',db2='Illum_Retina_BXD_RankInv0410',ProbeSetID2='ILMN_2701750',CellID2='',rank='1')) | [**0.349 79**](javascript:showCorrelationPlot2(db='Illum_Retina_BXD_RankInv0410',ProbeSetID='ILMN_2808186',CellID='',db2='Illum_Retina_BXD_RankInv0410',ProbeSetID2='ILMN_1239448',CellID2='',rank='1')) | [**-0.445 79**](javascript:showCorrelationPlot2(db='Illum_Retina_BXD_RankInv0410',ProbeSetID='ILMN_2808186',CellID='',db2='Illum_Retina_BXD_RankInv0410',ProbeSetID2='ILMN_2583882',CellID2='',rank='1')) | [**0.411 79**](javascript:showCorrelationPlot2(db='Illum_Retina_BXD_RankInv0410',ProbeSetID='ILMN_2808186',CellID='',db2='Illum_Retina_BXD_RankInv0410',ProbeSetID2='ILMN_2915166',CellID2='',rank='1')) | [**0.528 79**](javascript:showCorrelationPlot2(db='Illum_Retina_BXD_RankInv0410',ProbeSetID='ILMN_2808186',CellID='',db2='Illum_Retina_BXD_RankInv0410',ProbeSetID2='ILMN_2702997',CellID2='',rank='1')) | [**0.439 79**](javascript:showCorrelationPlot2(db='Illum_Retina_BXD_RankInv0410',ProbeSetID='ILMN_2808186',CellID='',db2='Illum_Retina_BXD_RankInv0410',ProbeSetID2='ILMN_2435206',CellID2='',rank='1')) | [**0.654 79**](javascript:showCorrelationPlot2(db='Illum_Retina_BXD_RankInv0410',ProbeSetID='ILMN_2808186',CellID='',db2='Illum_Retina_BXD_RankInv0410',ProbeSetID2='ILMN_2789888',CellID2='',rank='1')) | [**0.722 79**](javascript:showCorrelationPlot2(db='Illum_Retina_BXD_RankInv0410',ProbeSetID='ILMN_2808186',CellID='',db2='Illum_Retina_BXD_RankInv0410',ProbeSetID2='ILMN_1218901',CellID2='',rank='1')) | [**0.475 79**](javascript:showCorrelationPlot2(db='Illum_Retina_BXD_RankInv0410',ProbeSetID='ILMN_2808186',CellID='',db2='Illum_Retina_BXD_RankInv0410',ProbeSetID2='ILMN_1254734',CellID2='',rank='1')) | [**0.499 79**](javascript:showCorrelationPlot2(db='Illum_Retina_BXD_RankInv0410',ProbeSetID='ILMN_2808186',CellID='',db2='Illum_Retina_BXD_RankInv0410',ProbeSetID2='ILMN_3008924',CellID2='',rank='1')) | [**0.477 79**](javascript:showCorrelationPlot2(db='Illum_Retina_BXD_RankInv0410',ProbeSetID='ILMN_2808186',CellID='',db2='Illum_Retina_BXD_RankInv0410',ProbeSetID2='ILMN_2628175',CellID2='',rank='1')) | [**0.416 79**](javascript:showCorrelationPlot2(db='Illum_Retina_BXD_RankInv0410',ProbeSetID='ILMN_2808186',CellID='',db2='Illum_Retina_BXD_RankInv0410',ProbeSetID2='ILMN_2891573',CellID2='',rank='1')) | [**0.428 79**](javascript:showCorrelationPlot2(db='Illum_Retina_BXD_RankInv0410',ProbeSetID='ILMN_2808186',CellID='',db2='Illum_Retina_BXD_RankInv0410',ProbeSetID2='ILMN_1220846',CellID2='',rank='1')) | [**0.202 79**](javascript:showCorrelationPlot2(db='Illum_Retina_BXD_RankInv0410',ProbeSetID='ILMN_2808186',CellID='',db2='Illum_Retina_BXD_RankInv0410',ProbeSetID2='ILMN_1241962',CellID2='',rank='1')) | [**0.493 79**](javascript:showCorrelationPlot2(db='Illum_Retina_BXD_RankInv0410',ProbeSetID='ILMN_2808186',CellID='',db2='Illum_Retina_BXD_RankInv0410',ProbeSetID2='ILMN_3163027',CellID2='',rank='1')) | [**0.386 79**](javascript:showCorrelationPlot2(db='Illum_Retina_BXD_RankInv0410',ProbeSetID='ILMN_2808186',CellID='',db2='Illum_Retina_BXD_RankInv0410',ProbeSetID2='ILMN_2775098',CellID2='',rank='1')) | [**0.305 79**](javascript:showCorrelationPlot2(db='Illum_Retina_BXD_RankInv0410',ProbeSetID='ILMN_2808186',CellID='',db2='Illum_Retina_BXD_RankInv0410',ProbeSetID2='ILMN_2752552',CellID2='',rank='1')) | [**0.693 79**](javascript:showCorrelationPlot2(db='Illum_Retina_BXD_RankInv0410',ProbeSetID='ILMN_2808186',CellID='',db2='Illum_Retina_BXD_RankInv0410',ProbeSetID2='ILMN_2730293',CellID2='',rank='1')) | [**0.333 79**](javascript:showCorrelationPlot2(db='Illum_Retina_BXD_RankInv0410',ProbeSetID='ILMN_2808186',CellID='',db2='Illum_Retina_BXD_RankInv0410',ProbeSetID2='ILMN_2680872',CellID2='',rank='1')) | [**0.069 79**](javascript:showCorrelationPlot2(db='Illum_Retina_BXD_RankInv0410',ProbeSetID='ILMN_2808186',CellID='',db2='Illum_Retina_BXD_RankInv0410',ProbeSetID2='ILMN_2768533',CellID2='',rank='1')) | [**-0.327 79**](javascript:showCorrelationPlot2(db='Illum_Retina_BXD_RankInv0410',ProbeSetID='ILMN_2808186',CellID='',db2='Illum_Retina_BXD_RankInv0410',ProbeSetID2='ILMN_3121255',CellID2='',rank='1')) | [**-0.363 79**](javascript:showCorrelationPlot2(db='Illum_Retina_BXD_RankInv0410',ProbeSetID='ILMN_2808186',CellID='',db2='Illum_Retina_BXD_RankInv0410',ProbeSetID2='ILMN_2484527',CellID2='',rank='1')) | [**0.160 79**](javascript:showCorrelationPlot2(db='Illum_Retina_BXD_RankInv0410',ProbeSetID='ILMN_2808186',CellID='',db2='Illum_Retina_BXD_RankInv0410',ProbeSetID2='ILMN_3045723',CellID2='',rank='1')) | [**0.369 79**](javascript:showCorrelationPlot2(db='Illum_Retina_BXD_RankInv0410',ProbeSetID='ILMN_2808186',CellID='',db2='Illum_Retina_BXD_RankInv0410',ProbeSetID2='ILMN_2486573',CellID2='',rank='1')) |
|  | [**Trait 27: ILMN_2689056**](javascript:showDatabase2('Illum_Retina_BXD_RankInv0410','ILMN_2689056','');)  Symbol: Cd2bp2  Cd2bp2 | [**0.652 79**](javascript:showCorrelationPlot2(db='Illum_Retina_BXD_RankInv0410',ProbeSetID='ILMN_2689056',CellID='',db2='Illum_Retina_BXD_RankInv0410',ProbeSetID2='ILMN_2450384',CellID2='',rank='0')) | [**0.799 79**](javascript:showCorrelationPlot2(db='Illum_Retina_BXD_RankInv0410',ProbeSetID='ILMN_2689056',CellID='',db2='Illum_Retina_BXD_RankInv0410',ProbeSetID2='ILMN_3068754',CellID2='',rank='0')) | [**0.706 79**](javascript:showCorrelationPlot2(db='Illum_Retina_BXD_RankInv0410',ProbeSetID='ILMN_2689056',CellID='',db2='Illum_Retina_BXD_RankInv0410',ProbeSetID2='ILMN_2677092',CellID2='',rank='0')) | [**0.718 79**](javascript:showCorrelationPlot2(db='Illum_Retina_BXD_RankInv0410',ProbeSetID='ILMN_2689056',CellID='',db2='Illum_Retina_BXD_RankInv0410',ProbeSetID2='ILMN_2624938',CellID2='',rank='0')) | [**0.527 79**](javascript:showCorrelationPlot2(db='Illum_Retina_BXD_RankInv0410',ProbeSetID='ILMN_2689056',CellID='',db2='Illum_Retina_BXD_RankInv0410',ProbeSetID2='ILMN_2847269',CellID2='',rank='0')) | [**0.598 79**](javascript:showCorrelationPlot2(db='Illum_Retina_BXD_RankInv0410',ProbeSetID='ILMN_2689056',CellID='',db2='Illum_Retina_BXD_RankInv0410',ProbeSetID2='ILMN_2839682',CellID2='',rank='0')) | [**0.589 79**](javascript:showCorrelationPlot2(db='Illum_Retina_BXD_RankInv0410',ProbeSetID='ILMN_2689056',CellID='',db2='Illum_Retina_BXD_RankInv0410',ProbeSetID2='ILMN_3147135',CellID2='',rank='0')) | [**0.620 79**](javascript:showCorrelationPlot2(db='Illum_Retina_BXD_RankInv0410',ProbeSetID='ILMN_2689056',CellID='',db2='Illum_Retina_BXD_RankInv0410',ProbeSetID2='ILMN_1255823',CellID2='',rank='0')) | [**0.694 79**](javascript:showCorrelationPlot2(db='Illum_Retina_BXD_RankInv0410',ProbeSetID='ILMN_2689056',CellID='',db2='Illum_Retina_BXD_RankInv0410',ProbeSetID2='ILMN_2626143',CellID2='',rank='0')) | [**0.550 79**](javascript:showCorrelationPlot2(db='Illum_Retina_BXD_RankInv0410',ProbeSetID='ILMN_2689056',CellID='',db2='Illum_Retina_BXD_RankInv0410',ProbeSetID2='ILMN_1234930',CellID2='',rank='0')) | [**0.518 79**](javascript:showCorrelationPlot2(db='Illum_Retina_BXD_RankInv0410',ProbeSetID='ILMN_2689056',CellID='',db2='Illum_Retina_BXD_RankInv0410',ProbeSetID2='ILMN_1249408',CellID2='',rank='0')) | [**0.556 79**](javascript:showCorrelationPlot2(db='Illum_Retina_BXD_RankInv0410',ProbeSetID='ILMN_2689056',CellID='',db2='Illum_Retina_BXD_RankInv0410',ProbeSetID2='ILMN_2971559',CellID2='',rank='0')) | [**0.568 79**](javascript:showCorrelationPlot2(db='Illum_Retina_BXD_RankInv0410',ProbeSetID='ILMN_2689056',CellID='',db2='Illum_Retina_BXD_RankInv0410',ProbeSetID2='ILMN_1258455',CellID2='',rank='0')) | [**0.580 79**](javascript:showCorrelationPlot2(db='Illum_Retina_BXD_RankInv0410',ProbeSetID='ILMN_2689056',CellID='',db2='Illum_Retina_BXD_RankInv0410',ProbeSetID2='ILMN_2691613',CellID2='',rank='0')) | [**0.638 79**](javascript:showCorrelationPlot2(db='Illum_Retina_BXD_RankInv0410',ProbeSetID='ILMN_2689056',CellID='',db2='Illum_Retina_BXD_RankInv0410',ProbeSetID2='ILMN_1253600',CellID2='',rank='0')) | [**0.562 79**](javascript:showCorrelationPlot2(db='Illum_Retina_BXD_RankInv0410',ProbeSetID='ILMN_2689056',CellID='',db2='Illum_Retina_BXD_RankInv0410',ProbeSetID2='ILMN_2991545',CellID2='',rank='0')) | [**0.604 79**](javascript:showCorrelationPlot2(db='Illum_Retina_BXD_RankInv0410',ProbeSetID='ILMN_2689056',CellID='',db2='Illum_Retina_BXD_RankInv0410',ProbeSetID2='ILMN_2623591',CellID2='',rank='0')) | [**0.600 79**](javascript:showCorrelationPlot2(db='Illum_Retina_BXD_RankInv0410',ProbeSetID='ILMN_2689056',CellID='',db2='Illum_Retina_BXD_RankInv0410',ProbeSetID2='ILMN_2603568',CellID2='',rank='0')) | [**0.551 79**](javascript:showCorrelationPlot2(db='Illum_Retina_BXD_RankInv0410',ProbeSetID='ILMN_2689056',CellID='',db2='Illum_Retina_BXD_RankInv0410',ProbeSetID2='ILMN_2992541',CellID2='',rank='0')) | [**0.292 79**](javascript:showCorrelationPlot2(db='Illum_Retina_BXD_RankInv0410',ProbeSetID='ILMN_2689056',CellID='',db2='Illum_Retina_BXD_RankInv0410',ProbeSetID2='ILMN_2792868',CellID2='',rank='0')) | [**0.429 79**](javascript:showCorrelationPlot2(db='Illum_Retina_BXD_RankInv0410',ProbeSetID='ILMN_2689056',CellID='',db2='Illum_Retina_BXD_RankInv0410',ProbeSetID2='ILMN_3129497',CellID2='',rank='0')) | [**0.425 79**](javascript:showCorrelationPlot2(db='Illum_Retina_BXD_RankInv0410',ProbeSetID='ILMN_2689056',CellID='',db2='Illum_Retina_BXD_RankInv0410',ProbeSetID2='ILMN_1224034',CellID2='',rank='0')) | [**0.559 79**](javascript:showCorrelationPlot2(db='Illum_Retina_BXD_RankInv0410',ProbeSetID='ILMN_2689056',CellID='',db2='Illum_Retina_BXD_RankInv0410',ProbeSetID2='ILMN_2639819',CellID2='',rank='0')) | [**0.502 79**](javascript:showCorrelationPlot2(db='Illum_Retina_BXD_RankInv0410',ProbeSetID='ILMN_2689056',CellID='',db2='Illum_Retina_BXD_RankInv0410',ProbeSetID2='ILMN_2994779',CellID2='',rank='0')) | [**0.434 79**](javascript:showCorrelationPlot2(db='Illum_Retina_BXD_RankInv0410',ProbeSetID='ILMN_2689056',CellID='',db2='Illum_Retina_BXD_RankInv0410',ProbeSetID2='ILMN_2625047',CellID2='',rank='0')) | [**0.638 79**](javascript:showCorrelationPlot2(db='Illum_Retina_BXD_RankInv0410',ProbeSetID='ILMN_2689056',CellID='',db2='Illum_Retina_BXD_RankInv0410',ProbeSetID2='ILMN_2808186',CellID2='',rank='0')) | [***n* 79**](javascript:showDatabase2('Illum_Retina_BXD_RankInv0410','ILMN_2689056','')) | [**0.528 79**](javascript:showCorrelationPlot2(db='Illum_Retina_BXD_RankInv0410',ProbeSetID='ILMN_2689056',CellID='',db2='Illum_Retina_BXD_RankInv0410',ProbeSetID2='ILMN_2648386',CellID2='',rank='1')) | [**0.511 79**](javascript:showCorrelationPlot2(db='Illum_Retina_BXD_RankInv0410',ProbeSetID='ILMN_2689056',CellID='',db2='Illum_Retina_BXD_RankInv0410',ProbeSetID2='ILMN_2869225',CellID2='',rank='1')) | [**0.473 79**](javascript:showCorrelationPlot2(db='Illum_Retina_BXD_RankInv0410',ProbeSetID='ILMN_2689056',CellID='',db2='Illum_Retina_BXD_RankInv0410',ProbeSetID2='ILMN_2728431',CellID2='',rank='1')) | [**0.703 79**](javascript:showCorrelationPlot2(db='Illum_Retina_BXD_RankInv0410',ProbeSetID='ILMN_2689056',CellID='',db2='Illum_Retina_BXD_RankInv0410',ProbeSetID2='ILMN_3094608',CellID2='',rank='1')) | [**0.424 79**](javascript:showCorrelationPlot2(db='Illum_Retina_BXD_RankInv0410',ProbeSetID='ILMN_2689056',CellID='',db2='Illum_Retina_BXD_RankInv0410',ProbeSetID2='ILMN_2896528',CellID2='',rank='1')) | [**0.352 79**](javascript:showCorrelationPlot2(db='Illum_Retina_BXD_RankInv0410',ProbeSetID='ILMN_2689056',CellID='',db2='Illum_Retina_BXD_RankInv0410',ProbeSetID2='ILMN_2701750',CellID2='',rank='1')) | [**0.568 79**](javascript:showCorrelationPlot2(db='Illum_Retina_BXD_RankInv0410',ProbeSetID='ILMN_2689056',CellID='',db2='Illum_Retina_BXD_RankInv0410',ProbeSetID2='ILMN_1239448',CellID2='',rank='1')) | [**-0.558 79**](javascript:showCorrelationPlot2(db='Illum_Retina_BXD_RankInv0410',ProbeSetID='ILMN_2689056',CellID='',db2='Illum_Retina_BXD_RankInv0410',ProbeSetID2='ILMN_2583882',CellID2='',rank='1')) | [**0.394 79**](javascript:showCorrelationPlot2(db='Illum_Retina_BXD_RankInv0410',ProbeSetID='ILMN_2689056',CellID='',db2='Illum_Retina_BXD_RankInv0410',ProbeSetID2='ILMN_2915166',CellID2='',rank='1')) | [**0.508 79**](javascript:showCorrelationPlot2(db='Illum_Retina_BXD_RankInv0410',ProbeSetID='ILMN_2689056',CellID='',db2='Illum_Retina_BXD_RankInv0410',ProbeSetID2='ILMN_2702997',CellID2='',rank='1')) | [**0.532 79**](javascript:showCorrelationPlot2(db='Illum_Retina_BXD_RankInv0410',ProbeSetID='ILMN_2689056',CellID='',db2='Illum_Retina_BXD_RankInv0410',ProbeSetID2='ILMN_2435206',CellID2='',rank='1')) | [**0.691 79**](javascript:showCorrelationPlot2(db='Illum_Retina_BXD_RankInv0410',ProbeSetID='ILMN_2689056',CellID='',db2='Illum_Retina_BXD_RankInv0410',ProbeSetID2='ILMN_2789888',CellID2='',rank='1')) | [**0.728 79**](javascript:showCorrelationPlot2(db='Illum_Retina_BXD_RankInv0410',ProbeSetID='ILMN_2689056',CellID='',db2='Illum_Retina_BXD_RankInv0410',ProbeSetID2='ILMN_1218901',CellID2='',rank='1')) | [**0.580 79**](javascript:showCorrelationPlot2(db='Illum_Retina_BXD_RankInv0410',ProbeSetID='ILMN_2689056',CellID='',db2='Illum_Retina_BXD_RankInv0410',ProbeSetID2='ILMN_1254734',CellID2='',rank='1')) | [**0.431 79**](javascript:showCorrelationPlot2(db='Illum_Retina_BXD_RankInv0410',ProbeSetID='ILMN_2689056',CellID='',db2='Illum_Retina_BXD_RankInv0410',ProbeSetID2='ILMN_3008924',CellID2='',rank='1')) | [**0.706 79**](javascript:showCorrelationPlot2(db='Illum_Retina_BXD_RankInv0410',ProbeSetID='ILMN_2689056',CellID='',db2='Illum_Retina_BXD_RankInv0410',ProbeSetID2='ILMN_2628175',CellID2='',rank='1')) | [**0.713 79**](javascript:showCorrelationPlot2(db='Illum_Retina_BXD_RankInv0410',ProbeSetID='ILMN_2689056',CellID='',db2='Illum_Retina_BXD_RankInv0410',ProbeSetID2='ILMN_2891573',CellID2='',rank='1')) | [**0.525 79**](javascript:showCorrelationPlot2(db='Illum_Retina_BXD_RankInv0410',ProbeSetID='ILMN_2689056',CellID='',db2='Illum_Retina_BXD_RankInv0410',ProbeSetID2='ILMN_1220846',CellID2='',rank='1')) | [**0.386 79**](javascript:showCorrelationPlot2(db='Illum_Retina_BXD_RankInv0410',ProbeSetID='ILMN_2689056',CellID='',db2='Illum_Retina_BXD_RankInv0410',ProbeSetID2='ILMN_1241962',CellID2='',rank='1')) | [**0.508 79**](javascript:showCorrelationPlot2(db='Illum_Retina_BXD_RankInv0410',ProbeSetID='ILMN_2689056',CellID='',db2='Illum_Retina_BXD_RankInv0410',ProbeSetID2='ILMN_3163027',CellID2='',rank='1')) | [**0.380 79**](javascript:showCorrelationPlot2(db='Illum_Retina_BXD_RankInv0410',ProbeSetID='ILMN_2689056',CellID='',db2='Illum_Retina_BXD_RankInv0410',ProbeSetID2='ILMN_2775098',CellID2='',rank='1')) | [**0.640 79**](javascript:showCorrelationPlot2(db='Illum_Retina_BXD_RankInv0410',ProbeSetID='ILMN_2689056',CellID='',db2='Illum_Retina_BXD_RankInv0410',ProbeSetID2='ILMN_2752552',CellID2='',rank='1')) | [**0.442 79**](javascript:showCorrelationPlot2(db='Illum_Retina_BXD_RankInv0410',ProbeSetID='ILMN_2689056',CellID='',db2='Illum_Retina_BXD_RankInv0410',ProbeSetID2='ILMN_2730293',CellID2='',rank='1')) | [**0.350 79**](javascript:showCorrelationPlot2(db='Illum_Retina_BXD_RankInv0410',ProbeSetID='ILMN_2689056',CellID='',db2='Illum_Retina_BXD_RankInv0410',ProbeSetID2='ILMN_2680872',CellID2='',rank='1')) | [**0.218 79**](javascript:showCorrelationPlot2(db='Illum_Retina_BXD_RankInv0410',ProbeSetID='ILMN_2689056',CellID='',db2='Illum_Retina_BXD_RankInv0410',ProbeSetID2='ILMN_2768533',CellID2='',rank='1')) | [**-0.346 79**](javascript:showCorrelationPlot2(db='Illum_Retina_BXD_RankInv0410',ProbeSetID='ILMN_2689056',CellID='',db2='Illum_Retina_BXD_RankInv0410',ProbeSetID2='ILMN_3121255',CellID2='',rank='1')) | [**-0.495 79**](javascript:showCorrelationPlot2(db='Illum_Retina_BXD_RankInv0410',ProbeSetID='ILMN_2689056',CellID='',db2='Illum_Retina_BXD_RankInv0410',ProbeSetID2='ILMN_2484527',CellID2='',rank='1')) | [**0.241 79**](javascript:showCorrelationPlot2(db='Illum_Retina_BXD_RankInv0410',ProbeSetID='ILMN_2689056',CellID='',db2='Illum_Retina_BXD_RankInv0410',ProbeSetID2='ILMN_3045723',CellID2='',rank='1')) | [**0.226 79**](javascript:showCorrelationPlot2(db='Illum_Retina_BXD_RankInv0410',ProbeSetID='ILMN_2689056',CellID='',db2='Illum_Retina_BXD_RankInv0410',ProbeSetID2='ILMN_2486573',CellID2='',rank='1')) |
|  | [**Trait 28: ILMN_2648386**](javascript:showDatabase2('Illum_Retina_BXD_RankInv0410','ILMN_2648386','');)  Symbol: 5730427N09Rik  5730427N09Rik | [**0.648 79**](javascript:showCorrelationPlot2(db='Illum_Retina_BXD_RankInv0410',ProbeSetID='ILMN_2648386',CellID='',db2='Illum_Retina_BXD_RankInv0410',ProbeSetID2='ILMN_2450384',CellID2='',rank='0')) | [**0.593 79**](javascript:showCorrelationPlot2(db='Illum_Retina_BXD_RankInv0410',ProbeSetID='ILMN_2648386',CellID='',db2='Illum_Retina_BXD_RankInv0410',ProbeSetID2='ILMN_3068754',CellID2='',rank='0')) | [**0.614 79**](javascript:showCorrelationPlot2(db='Illum_Retina_BXD_RankInv0410',ProbeSetID='ILMN_2648386',CellID='',db2='Illum_Retina_BXD_RankInv0410',ProbeSetID2='ILMN_2677092',CellID2='',rank='0')) | [**0.536 79**](javascript:showCorrelationPlot2(db='Illum_Retina_BXD_RankInv0410',ProbeSetID='ILMN_2648386',CellID='',db2='Illum_Retina_BXD_RankInv0410',ProbeSetID2='ILMN_2624938',CellID2='',rank='0')) | [**0.597 79**](javascript:showCorrelationPlot2(db='Illum_Retina_BXD_RankInv0410',ProbeSetID='ILMN_2648386',CellID='',db2='Illum_Retina_BXD_RankInv0410',ProbeSetID2='ILMN_2847269',CellID2='',rank='0')) | [**0.426 79**](javascript:showCorrelationPlot2(db='Illum_Retina_BXD_RankInv0410',ProbeSetID='ILMN_2648386',CellID='',db2='Illum_Retina_BXD_RankInv0410',ProbeSetID2='ILMN_2839682',CellID2='',rank='0')) | [**0.637 79**](javascript:showCorrelationPlot2(db='Illum_Retina_BXD_RankInv0410',ProbeSetID='ILMN_2648386',CellID='',db2='Illum_Retina_BXD_RankInv0410',ProbeSetID2='ILMN_3147135',CellID2='',rank='0')) | [**0.667 79**](javascript:showCorrelationPlot2(db='Illum_Retina_BXD_RankInv0410',ProbeSetID='ILMN_2648386',CellID='',db2='Illum_Retina_BXD_RankInv0410',ProbeSetID2='ILMN_1255823',CellID2='',rank='0')) | [**0.576 79**](javascript:showCorrelationPlot2(db='Illum_Retina_BXD_RankInv0410',ProbeSetID='ILMN_2648386',CellID='',db2='Illum_Retina_BXD_RankInv0410',ProbeSetID2='ILMN_2626143',CellID2='',rank='0')) | [**0.450 79**](javascript:showCorrelationPlot2(db='Illum_Retina_BXD_RankInv0410',ProbeSetID='ILMN_2648386',CellID='',db2='Illum_Retina_BXD_RankInv0410',ProbeSetID2='ILMN_1234930',CellID2='',rank='0')) | [**0.736 79**](javascript:showCorrelationPlot2(db='Illum_Retina_BXD_RankInv0410',ProbeSetID='ILMN_2648386',CellID='',db2='Illum_Retina_BXD_RankInv0410',ProbeSetID2='ILMN_1249408',CellID2='',rank='0')) | [**0.326 79**](javascript:showCorrelationPlot2(db='Illum_Retina_BXD_RankInv0410',ProbeSetID='ILMN_2648386',CellID='',db2='Illum_Retina_BXD_RankInv0410',ProbeSetID2='ILMN_2971559',CellID2='',rank='0')) | [**0.411 79**](javascript:showCorrelationPlot2(db='Illum_Retina_BXD_RankInv0410',ProbeSetID='ILMN_2648386',CellID='',db2='Illum_Retina_BXD_RankInv0410',ProbeSetID2='ILMN_1258455',CellID2='',rank='0')) | [**0.630 79**](javascript:showCorrelationPlot2(db='Illum_Retina_BXD_RankInv0410',ProbeSetID='ILMN_2648386',CellID='',db2='Illum_Retina_BXD_RankInv0410',ProbeSetID2='ILMN_2691613',CellID2='',rank='0')) | [**0.472 79**](javascript:showCorrelationPlot2(db='Illum_Retina_BXD_RankInv0410',ProbeSetID='ILMN_2648386',CellID='',db2='Illum_Retina_BXD_RankInv0410',ProbeSetID2='ILMN_1253600',CellID2='',rank='0')) | [**0.506 79**](javascript:showCorrelationPlot2(db='Illum_Retina_BXD_RankInv0410',ProbeSetID='ILMN_2648386',CellID='',db2='Illum_Retina_BXD_RankInv0410',ProbeSetID2='ILMN_2991545',CellID2='',rank='0')) | [**0.473 79**](javascript:showCorrelationPlot2(db='Illum_Retina_BXD_RankInv0410',ProbeSetID='ILMN_2648386',CellID='',db2='Illum_Retina_BXD_RankInv0410',ProbeSetID2='ILMN_2623591',CellID2='',rank='0')) | [**0.533 79**](javascript:showCorrelationPlot2(db='Illum_Retina_BXD_RankInv0410',ProbeSetID='ILMN_2648386',CellID='',db2='Illum_Retina_BXD_RankInv0410',ProbeSetID2='ILMN_2603568',CellID2='',rank='0')) | [**0.619 79**](javascript:showCorrelationPlot2(db='Illum_Retina_BXD_RankInv0410',ProbeSetID='ILMN_2648386',CellID='',db2='Illum_Retina_BXD_RankInv0410',ProbeSetID2='ILMN_2992541',CellID2='',rank='0')) | [**0.648 79**](javascript:showCorrelationPlot2(db='Illum_Retina_BXD_RankInv0410',ProbeSetID='ILMN_2648386',CellID='',db2='Illum_Retina_BXD_RankInv0410',ProbeSetID2='ILMN_2792868',CellID2='',rank='0')) | [**0.433 79**](javascript:showCorrelationPlot2(db='Illum_Retina_BXD_RankInv0410',ProbeSetID='ILMN_2648386',CellID='',db2='Illum_Retina_BXD_RankInv0410',ProbeSetID2='ILMN_3129497',CellID2='',rank='0')) | [**0.409 79**](javascript:showCorrelationPlot2(db='Illum_Retina_BXD_RankInv0410',ProbeSetID='ILMN_2648386',CellID='',db2='Illum_Retina_BXD_RankInv0410',ProbeSetID2='ILMN_1224034',CellID2='',rank='0')) | [**0.666 79**](javascript:showCorrelationPlot2(db='Illum_Retina_BXD_RankInv0410',ProbeSetID='ILMN_2648386',CellID='',db2='Illum_Retina_BXD_RankInv0410',ProbeSetID2='ILMN_2639819',CellID2='',rank='0')) | [**0.499 79**](javascript:showCorrelationPlot2(db='Illum_Retina_BXD_RankInv0410',ProbeSetID='ILMN_2648386',CellID='',db2='Illum_Retina_BXD_RankInv0410',ProbeSetID2='ILMN_2994779',CellID2='',rank='0')) | [**0.448 79**](javascript:showCorrelationPlot2(db='Illum_Retina_BXD_RankInv0410',ProbeSetID='ILMN_2648386',CellID='',db2='Illum_Retina_BXD_RankInv0410',ProbeSetID2='ILMN_2625047',CellID2='',rank='0')) | [**0.307 79**](javascript:showCorrelationPlot2(db='Illum_Retina_BXD_RankInv0410',ProbeSetID='ILMN_2648386',CellID='',db2='Illum_Retina_BXD_RankInv0410',ProbeSetID2='ILMN_2808186',CellID2='',rank='0')) | [**0.500 79**](javascript:showCorrelationPlot2(db='Illum_Retina_BXD_RankInv0410',ProbeSetID='ILMN_2648386',CellID='',db2='Illum_Retina_BXD_RankInv0410',ProbeSetID2='ILMN_2689056',CellID2='',rank='0')) | [***n* 79**](javascript:showDatabase2('Illum_Retina_BXD_RankInv0410','ILMN_2648386','')) | [**0.659 79**](javascript:showCorrelationPlot2(db='Illum_Retina_BXD_RankInv0410',ProbeSetID='ILMN_2648386',CellID='',db2='Illum_Retina_BXD_RankInv0410',ProbeSetID2='ILMN_2869225',CellID2='',rank='1')) | [**0.469 79**](javascript:showCorrelationPlot2(db='Illum_Retina_BXD_RankInv0410',ProbeSetID='ILMN_2648386',CellID='',db2='Illum_Retina_BXD_RankInv0410',ProbeSetID2='ILMN_2728431',CellID2='',rank='1')) | [**0.456 79**](javascript:showCorrelationPlot2(db='Illum_Retina_BXD_RankInv0410',ProbeSetID='ILMN_2648386',CellID='',db2='Illum_Retina_BXD_RankInv0410',ProbeSetID2='ILMN_3094608',CellID2='',rank='1')) | [**0.612 79**](javascript:showCorrelationPlot2(db='Illum_Retina_BXD_RankInv0410',ProbeSetID='ILMN_2648386',CellID='',db2='Illum_Retina_BXD_RankInv0410',ProbeSetID2='ILMN_2896528',CellID2='',rank='1')) | [**0.518 79**](javascript:showCorrelationPlot2(db='Illum_Retina_BXD_RankInv0410',ProbeSetID='ILMN_2648386',CellID='',db2='Illum_Retina_BXD_RankInv0410',ProbeSetID2='ILMN_2701750',CellID2='',rank='1')) | [**0.478 79**](javascript:showCorrelationPlot2(db='Illum_Retina_BXD_RankInv0410',ProbeSetID='ILMN_2648386',CellID='',db2='Illum_Retina_BXD_RankInv0410',ProbeSetID2='ILMN_1239448',CellID2='',rank='1')) | [**-0.532 79**](javascript:showCorrelationPlot2(db='Illum_Retina_BXD_RankInv0410',ProbeSetID='ILMN_2648386',CellID='',db2='Illum_Retina_BXD_RankInv0410',ProbeSetID2='ILMN_2583882',CellID2='',rank='1')) | [**0.568 79**](javascript:showCorrelationPlot2(db='Illum_Retina_BXD_RankInv0410',ProbeSetID='ILMN_2648386',CellID='',db2='Illum_Retina_BXD_RankInv0410',ProbeSetID2='ILMN_2915166',CellID2='',rank='1')) | [**0.486 79**](javascript:showCorrelationPlot2(db='Illum_Retina_BXD_RankInv0410',ProbeSetID='ILMN_2648386',CellID='',db2='Illum_Retina_BXD_RankInv0410',ProbeSetID2='ILMN_2702997',CellID2='',rank='1')) | [**0.483 79**](javascript:showCorrelationPlot2(db='Illum_Retina_BXD_RankInv0410',ProbeSetID='ILMN_2648386',CellID='',db2='Illum_Retina_BXD_RankInv0410',ProbeSetID2='ILMN_2435206',CellID2='',rank='1')) | [**0.547 79**](javascript:showCorrelationPlot2(db='Illum_Retina_BXD_RankInv0410',ProbeSetID='ILMN_2648386',CellID='',db2='Illum_Retina_BXD_RankInv0410',ProbeSetID2='ILMN_2789888',CellID2='',rank='1')) | [**0.381 79**](javascript:showCorrelationPlot2(db='Illum_Retina_BXD_RankInv0410',ProbeSetID='ILMN_2648386',CellID='',db2='Illum_Retina_BXD_RankInv0410',ProbeSetID2='ILMN_1218901',CellID2='',rank='1')) | [**0.605 79**](javascript:showCorrelationPlot2(db='Illum_Retina_BXD_RankInv0410',ProbeSetID='ILMN_2648386',CellID='',db2='Illum_Retina_BXD_RankInv0410',ProbeSetID2='ILMN_1254734',CellID2='',rank='1')) | [**0.484 79**](javascript:showCorrelationPlot2(db='Illum_Retina_BXD_RankInv0410',ProbeSetID='ILMN_2648386',CellID='',db2='Illum_Retina_BXD_RankInv0410',ProbeSetID2='ILMN_3008924',CellID2='',rank='1')) | [**0.526 79**](javascript:showCorrelationPlot2(db='Illum_Retina_BXD_RankInv0410',ProbeSetID='ILMN_2648386',CellID='',db2='Illum_Retina_BXD_RankInv0410',ProbeSetID2='ILMN_2628175',CellID2='',rank='1')) | [**0.526 79**](javascript:showCorrelationPlot2(db='Illum_Retina_BXD_RankInv0410',ProbeSetID='ILMN_2648386',CellID='',db2='Illum_Retina_BXD_RankInv0410',ProbeSetID2='ILMN_2891573',CellID2='',rank='1')) | [**0.596 79**](javascript:showCorrelationPlot2(db='Illum_Retina_BXD_RankInv0410',ProbeSetID='ILMN_2648386',CellID='',db2='Illum_Retina_BXD_RankInv0410',ProbeSetID2='ILMN_1220846',CellID2='',rank='1')) | [**0.588 79**](javascript:showCorrelationPlot2(db='Illum_Retina_BXD_RankInv0410',ProbeSetID='ILMN_2648386',CellID='',db2='Illum_Retina_BXD_RankInv0410',ProbeSetID2='ILMN_1241962',CellID2='',rank='1')) | [**0.447 79**](javascript:showCorrelationPlot2(db='Illum_Retina_BXD_RankInv0410',ProbeSetID='ILMN_2648386',CellID='',db2='Illum_Retina_BXD_RankInv0410',ProbeSetID2='ILMN_3163027',CellID2='',rank='1')) | [**0.580 79**](javascript:showCorrelationPlot2(db='Illum_Retina_BXD_RankInv0410',ProbeSetID='ILMN_2648386',CellID='',db2='Illum_Retina_BXD_RankInv0410',ProbeSetID2='ILMN_2775098',CellID2='',rank='1')) | [**0.500 79**](javascript:showCorrelationPlot2(db='Illum_Retina_BXD_RankInv0410',ProbeSetID='ILMN_2648386',CellID='',db2='Illum_Retina_BXD_RankInv0410',ProbeSetID2='ILMN_2752552',CellID2='',rank='1')) | [**0.386 79**](javascript:showCorrelationPlot2(db='Illum_Retina_BXD_RankInv0410',ProbeSetID='ILMN_2648386',CellID='',db2='Illum_Retina_BXD_RankInv0410',ProbeSetID2='ILMN_2730293',CellID2='',rank='1')) | [**0.297 79**](javascript:showCorrelationPlot2(db='Illum_Retina_BXD_RankInv0410',ProbeSetID='ILMN_2648386',CellID='',db2='Illum_Retina_BXD_RankInv0410',ProbeSetID2='ILMN_2680872',CellID2='',rank='1')) | [**0.336 79**](javascript:showCorrelationPlot2(db='Illum_Retina_BXD_RankInv0410',ProbeSetID='ILMN_2648386',CellID='',db2='Illum_Retina_BXD_RankInv0410',ProbeSetID2='ILMN_2768533',CellID2='',rank='1')) | [**-0.160 79**](javascript:showCorrelationPlot2(db='Illum_Retina_BXD_RankInv0410',ProbeSetID='ILMN_2648386',CellID='',db2='Illum_Retina_BXD_RankInv0410',ProbeSetID2='ILMN_3121255',CellID2='',rank='1')) | [**-0.308 79**](javascript:showCorrelationPlot2(db='Illum_Retina_BXD_RankInv0410',ProbeSetID='ILMN_2648386',CellID='',db2='Illum_Retina_BXD_RankInv0410',ProbeSetID2='ILMN_2484527',CellID2='',rank='1')) | [**0.203 79**](javascript:showCorrelationPlot2(db='Illum_Retina_BXD_RankInv0410',ProbeSetID='ILMN_2648386',CellID='',db2='Illum_Retina_BXD_RankInv0410',ProbeSetID2='ILMN_3045723',CellID2='',rank='1')) | [**0.291 79**](javascript:showCorrelationPlot2(db='Illum_Retina_BXD_RankInv0410',ProbeSetID='ILMN_2648386',CellID='',db2='Illum_Retina_BXD_RankInv0410',ProbeSetID2='ILMN_2486573',CellID2='',rank='1')) |
|  | [**Trait 29: ILMN_2869225**](javascript:showDatabase2('Illum_Retina_BXD_RankInv0410','ILMN_2869225','');)  Symbol: Rpl19  Rpl19 | [**0.645 79**](javascript:showCorrelationPlot2(db='Illum_Retina_BXD_RankInv0410',ProbeSetID='ILMN_2869225',CellID='',db2='Illum_Retina_BXD_RankInv0410',ProbeSetID2='ILMN_2450384',CellID2='',rank='0')) | [**0.553 79**](javascript:showCorrelationPlot2(db='Illum_Retina_BXD_RankInv0410',ProbeSetID='ILMN_2869225',CellID='',db2='Illum_Retina_BXD_RankInv0410',ProbeSetID2='ILMN_3068754',CellID2='',rank='0')) | [**0.507 79**](javascript:showCorrelationPlot2(db='Illum_Retina_BXD_RankInv0410',ProbeSetID='ILMN_2869225',CellID='',db2='Illum_Retina_BXD_RankInv0410',ProbeSetID2='ILMN_2677092',CellID2='',rank='0')) | [**0.625 79**](javascript:showCorrelationPlot2(db='Illum_Retina_BXD_RankInv0410',ProbeSetID='ILMN_2869225',CellID='',db2='Illum_Retina_BXD_RankInv0410',ProbeSetID2='ILMN_2624938',CellID2='',rank='0')) | [**0.664 79**](javascript:showCorrelationPlot2(db='Illum_Retina_BXD_RankInv0410',ProbeSetID='ILMN_2869225',CellID='',db2='Illum_Retina_BXD_RankInv0410',ProbeSetID2='ILMN_2847269',CellID2='',rank='0')) | [**0.427 79**](javascript:showCorrelationPlot2(db='Illum_Retina_BXD_RankInv0410',ProbeSetID='ILMN_2869225',CellID='',db2='Illum_Retina_BXD_RankInv0410',ProbeSetID2='ILMN_2839682',CellID2='',rank='0')) | [**0.526 79**](javascript:showCorrelationPlot2(db='Illum_Retina_BXD_RankInv0410',ProbeSetID='ILMN_2869225',CellID='',db2='Illum_Retina_BXD_RankInv0410',ProbeSetID2='ILMN_3147135',CellID2='',rank='0')) | [**0.724 79**](javascript:showCorrelationPlot2(db='Illum_Retina_BXD_RankInv0410',ProbeSetID='ILMN_2869225',CellID='',db2='Illum_Retina_BXD_RankInv0410',ProbeSetID2='ILMN_1255823',CellID2='',rank='0')) | [**0.720 79**](javascript:showCorrelationPlot2(db='Illum_Retina_BXD_RankInv0410',ProbeSetID='ILMN_2869225',CellID='',db2='Illum_Retina_BXD_RankInv0410',ProbeSetID2='ILMN_2626143',CellID2='',rank='0')) | [**0.609 79**](javascript:showCorrelationPlot2(db='Illum_Retina_BXD_RankInv0410',ProbeSetID='ILMN_2869225',CellID='',db2='Illum_Retina_BXD_RankInv0410',ProbeSetID2='ILMN_1234930',CellID2='',rank='0')) | [**0.738 79**](javascript:showCorrelationPlot2(db='Illum_Retina_BXD_RankInv0410',ProbeSetID='ILMN_2869225',CellID='',db2='Illum_Retina_BXD_RankInv0410',ProbeSetID2='ILMN_1249408',CellID2='',rank='0')) | [**0.492 79**](javascript:showCorrelationPlot2(db='Illum_Retina_BXD_RankInv0410',ProbeSetID='ILMN_2869225',CellID='',db2='Illum_Retina_BXD_RankInv0410',ProbeSetID2='ILMN_2971559',CellID2='',rank='0')) | [**0.334 79**](javascript:showCorrelationPlot2(db='Illum_Retina_BXD_RankInv0410',ProbeSetID='ILMN_2869225',CellID='',db2='Illum_Retina_BXD_RankInv0410',ProbeSetID2='ILMN_1258455',CellID2='',rank='0')) | [**0.637 79**](javascript:showCorrelationPlot2(db='Illum_Retina_BXD_RankInv0410',ProbeSetID='ILMN_2869225',CellID='',db2='Illum_Retina_BXD_RankInv0410',ProbeSetID2='ILMN_2691613',CellID2='',rank='0')) | [**0.403 79**](javascript:showCorrelationPlot2(db='Illum_Retina_BXD_RankInv0410',ProbeSetID='ILMN_2869225',CellID='',db2='Illum_Retina_BXD_RankInv0410',ProbeSetID2='ILMN_1253600',CellID2='',rank='0')) | [**0.576 79**](javascript:showCorrelationPlot2(db='Illum_Retina_BXD_RankInv0410',ProbeSetID='ILMN_2869225',CellID='',db2='Illum_Retina_BXD_RankInv0410',ProbeSetID2='ILMN_2991545',CellID2='',rank='0')) | [**0.514 79**](javascript:showCorrelationPlot2(db='Illum_Retina_BXD_RankInv0410',ProbeSetID='ILMN_2869225',CellID='',db2='Illum_Retina_BXD_RankInv0410',ProbeSetID2='ILMN_2623591',CellID2='',rank='0')) | [**0.434 79**](javascript:showCorrelationPlot2(db='Illum_Retina_BXD_RankInv0410',ProbeSetID='ILMN_2869225',CellID='',db2='Illum_Retina_BXD_RankInv0410',ProbeSetID2='ILMN_2603568',CellID2='',rank='0')) | [**0.703 79**](javascript:showCorrelationPlot2(db='Illum_Retina_BXD_RankInv0410',ProbeSetID='ILMN_2869225',CellID='',db2='Illum_Retina_BXD_RankInv0410',ProbeSetID2='ILMN_2992541',CellID2='',rank='0')) | [**0.572 79**](javascript:showCorrelationPlot2(db='Illum_Retina_BXD_RankInv0410',ProbeSetID='ILMN_2869225',CellID='',db2='Illum_Retina_BXD_RankInv0410',ProbeSetID2='ILMN_2792868',CellID2='',rank='0')) | [**0.524 79**](javascript:showCorrelationPlot2(db='Illum_Retina_BXD_RankInv0410',ProbeSetID='ILMN_2869225',CellID='',db2='Illum_Retina_BXD_RankInv0410',ProbeSetID2='ILMN_3129497',CellID2='',rank='0')) | [**0.349 79**](javascript:showCorrelationPlot2(db='Illum_Retina_BXD_RankInv0410',ProbeSetID='ILMN_2869225',CellID='',db2='Illum_Retina_BXD_RankInv0410',ProbeSetID2='ILMN_1224034',CellID2='',rank='0')) | [**0.560 79**](javascript:showCorrelationPlot2(db='Illum_Retina_BXD_RankInv0410',ProbeSetID='ILMN_2869225',CellID='',db2='Illum_Retina_BXD_RankInv0410',ProbeSetID2='ILMN_2639819',CellID2='',rank='0')) | [**0.606 79**](javascript:showCorrelationPlot2(db='Illum_Retina_BXD_RankInv0410',ProbeSetID='ILMN_2869225',CellID='',db2='Illum_Retina_BXD_RankInv0410',ProbeSetID2='ILMN_2994779',CellID2='',rank='0')) | [**0.460 79**](javascript:showCorrelationPlot2(db='Illum_Retina_BXD_RankInv0410',ProbeSetID='ILMN_2869225',CellID='',db2='Illum_Retina_BXD_RankInv0410',ProbeSetID2='ILMN_2625047',CellID2='',rank='0')) | [**0.226 79**](javascript:showCorrelationPlot2(db='Illum_Retina_BXD_RankInv0410',ProbeSetID='ILMN_2869225',CellID='',db2='Illum_Retina_BXD_RankInv0410',ProbeSetID2='ILMN_2808186',CellID2='',rank='0')) | [**0.467 79**](javascript:showCorrelationPlot2(db='Illum_Retina_BXD_RankInv0410',ProbeSetID='ILMN_2869225',CellID='',db2='Illum_Retina_BXD_RankInv0410',ProbeSetID2='ILMN_2689056',CellID2='',rank='0')) | [**0.663 79**](javascript:showCorrelationPlot2(db='Illum_Retina_BXD_RankInv0410',ProbeSetID='ILMN_2869225',CellID='',db2='Illum_Retina_BXD_RankInv0410',ProbeSetID2='ILMN_2648386',CellID2='',rank='0')) | [***n* 79**](javascript:showDatabase2('Illum_Retina_BXD_RankInv0410','ILMN_2869225','')) | [**0.428 79**](javascript:showCorrelationPlot2(db='Illum_Retina_BXD_RankInv0410',ProbeSetID='ILMN_2869225',CellID='',db2='Illum_Retina_BXD_RankInv0410',ProbeSetID2='ILMN_2728431',CellID2='',rank='1')) | [**0.468 79**](javascript:showCorrelationPlot2(db='Illum_Retina_BXD_RankInv0410',ProbeSetID='ILMN_2869225',CellID='',db2='Illum_Retina_BXD_RankInv0410',ProbeSetID2='ILMN_3094608',CellID2='',rank='1')) | [**0.681 79**](javascript:showCorrelationPlot2(db='Illum_Retina_BXD_RankInv0410',ProbeSetID='ILMN_2869225',CellID='',db2='Illum_Retina_BXD_RankInv0410',ProbeSetID2='ILMN_2896528',CellID2='',rank='1')) | [**0.336 79**](javascript:showCorrelationPlot2(db='Illum_Retina_BXD_RankInv0410',ProbeSetID='ILMN_2869225',CellID='',db2='Illum_Retina_BXD_RankInv0410',ProbeSetID2='ILMN_2701750',CellID2='',rank='1')) | [**0.498 79**](javascript:showCorrelationPlot2(db='Illum_Retina_BXD_RankInv0410',ProbeSetID='ILMN_2869225',CellID='',db2='Illum_Retina_BXD_RankInv0410',ProbeSetID2='ILMN_1239448',CellID2='',rank='1')) | [**-0.290 79**](javascript:showCorrelationPlot2(db='Illum_Retina_BXD_RankInv0410',ProbeSetID='ILMN_2869225',CellID='',db2='Illum_Retina_BXD_RankInv0410',ProbeSetID2='ILMN_2583882',CellID2='',rank='1')) | [**0.617 79**](javascript:showCorrelationPlot2(db='Illum_Retina_BXD_RankInv0410',ProbeSetID='ILMN_2869225',CellID='',db2='Illum_Retina_BXD_RankInv0410',ProbeSetID2='ILMN_2915166',CellID2='',rank='1')) | [**0.609 79**](javascript:showCorrelationPlot2(db='Illum_Retina_BXD_RankInv0410',ProbeSetID='ILMN_2869225',CellID='',db2='Illum_Retina_BXD_RankInv0410',ProbeSetID2='ILMN_2702997',CellID2='',rank='1')) | [**0.711 79**](javascript:showCorrelationPlot2(db='Illum_Retina_BXD_RankInv0410',ProbeSetID='ILMN_2869225',CellID='',db2='Illum_Retina_BXD_RankInv0410',ProbeSetID2='ILMN_2435206',CellID2='',rank='1')) | [**0.439 79**](javascript:showCorrelationPlot2(db='Illum_Retina_BXD_RankInv0410',ProbeSetID='ILMN_2869225',CellID='',db2='Illum_Retina_BXD_RankInv0410',ProbeSetID2='ILMN_2789888',CellID2='',rank='1')) | [**0.393 79**](javascript:showCorrelationPlot2(db='Illum_Retina_BXD_RankInv0410',ProbeSetID='ILMN_2869225',CellID='',db2='Illum_Retina_BXD_RankInv0410',ProbeSetID2='ILMN_1218901',CellID2='',rank='1')) | [**0.522 79**](javascript:showCorrelationPlot2(db='Illum_Retina_BXD_RankInv0410',ProbeSetID='ILMN_2869225',CellID='',db2='Illum_Retina_BXD_RankInv0410',ProbeSetID2='ILMN_1254734',CellID2='',rank='1')) | [**0.281 79**](javascript:showCorrelationPlot2(db='Illum_Retina_BXD_RankInv0410',ProbeSetID='ILMN_2869225',CellID='',db2='Illum_Retina_BXD_RankInv0410',ProbeSetID2='ILMN_3008924',CellID2='',rank='1')) | [**0.463 79**](javascript:showCorrelationPlot2(db='Illum_Retina_BXD_RankInv0410',ProbeSetID='ILMN_2869225',CellID='',db2='Illum_Retina_BXD_RankInv0410',ProbeSetID2='ILMN_2628175',CellID2='',rank='1')) | [**0.537 79**](javascript:showCorrelationPlot2(db='Illum_Retina_BXD_RankInv0410',ProbeSetID='ILMN_2869225',CellID='',db2='Illum_Retina_BXD_RankInv0410',ProbeSetID2='ILMN_2891573',CellID2='',rank='1')) | [**0.554 79**](javascript:showCorrelationPlot2(db='Illum_Retina_BXD_RankInv0410',ProbeSetID='ILMN_2869225',CellID='',db2='Illum_Retina_BXD_RankInv0410',ProbeSetID2='ILMN_1220846',CellID2='',rank='1')) | [**0.662 79**](javascript:showCorrelationPlot2(db='Illum_Retina_BXD_RankInv0410',ProbeSetID='ILMN_2869225',CellID='',db2='Illum_Retina_BXD_RankInv0410',ProbeSetID2='ILMN_1241962',CellID2='',rank='1')) | [**0.646 79**](javascript:showCorrelationPlot2(db='Illum_Retina_BXD_RankInv0410',ProbeSetID='ILMN_2869225',CellID='',db2='Illum_Retina_BXD_RankInv0410',ProbeSetID2='ILMN_3163027',CellID2='',rank='1')) | [**0.407 79**](javascript:showCorrelationPlot2(db='Illum_Retina_BXD_RankInv0410',ProbeSetID='ILMN_2869225',CellID='',db2='Illum_Retina_BXD_RankInv0410',ProbeSetID2='ILMN_2775098',CellID2='',rank='1')) | [**0.689 79**](javascript:showCorrelationPlot2(db='Illum_Retina_BXD_RankInv0410',ProbeSetID='ILMN_2869225',CellID='',db2='Illum_Retina_BXD_RankInv0410',ProbeSetID2='ILMN_2752552',CellID2='',rank='1')) | [**0.346 79**](javascript:showCorrelationPlot2(db='Illum_Retina_BXD_RankInv0410',ProbeSetID='ILMN_2869225',CellID='',db2='Illum_Retina_BXD_RankInv0410',ProbeSetID2='ILMN_2730293',CellID2='',rank='1')) | [**0.471 79**](javascript:showCorrelationPlot2(db='Illum_Retina_BXD_RankInv0410',ProbeSetID='ILMN_2869225',CellID='',db2='Illum_Retina_BXD_RankInv0410',ProbeSetID2='ILMN_2680872',CellID2='',rank='1')) | [**0.124 79**](javascript:showCorrelationPlot2(db='Illum_Retina_BXD_RankInv0410',ProbeSetID='ILMN_2869225',CellID='',db2='Illum_Retina_BXD_RankInv0410',ProbeSetID2='ILMN_2768533',CellID2='',rank='1')) | [**-0.053 79**](javascript:showCorrelationPlot2(db='Illum_Retina_BXD_RankInv0410',ProbeSetID='ILMN_2869225',CellID='',db2='Illum_Retina_BXD_RankInv0410',ProbeSetID2='ILMN_3121255',CellID2='',rank='1')) | [**-0.161 79**](javascript:showCorrelationPlot2(db='Illum_Retina_BXD_RankInv0410',ProbeSetID='ILMN_2869225',CellID='',db2='Illum_Retina_BXD_RankInv0410',ProbeSetID2='ILMN_2484527',CellID2='',rank='1')) | [**0.109 79**](javascript:showCorrelationPlot2(db='Illum_Retina_BXD_RankInv0410',ProbeSetID='ILMN_2869225',CellID='',db2='Illum_Retina_BXD_RankInv0410',ProbeSetID2='ILMN_3045723',CellID2='',rank='1')) | [**0.157 79**](javascript:showCorrelationPlot2(db='Illum_Retina_BXD_RankInv0410',ProbeSetID='ILMN_2869225',CellID='',db2='Illum_Retina_BXD_RankInv0410',ProbeSetID2='ILMN_2486573',CellID2='',rank='1')) |
|  | [**Trait 30: ILMN_2728431**](javascript:showDatabase2('Illum_Retina_BXD_RankInv0410','ILMN_2728431','');)  Symbol: Cdc42ep4  Cdc42ep4 | [**0.645 79**](javascript:showCorrelationPlot2(db='Illum_Retina_BXD_RankInv0410',ProbeSetID='ILMN_2728431',CellID='',db2='Illum_Retina_BXD_RankInv0410',ProbeSetID2='ILMN_2450384',CellID2='',rank='0')) | [**0.458 79**](javascript:showCorrelationPlot2(db='Illum_Retina_BXD_RankInv0410',ProbeSetID='ILMN_2728431',CellID='',db2='Illum_Retina_BXD_RankInv0410',ProbeSetID2='ILMN_3068754',CellID2='',rank='0')) | [**0.471 79**](javascript:showCorrelationPlot2(db='Illum_Retina_BXD_RankInv0410',ProbeSetID='ILMN_2728431',CellID='',db2='Illum_Retina_BXD_RankInv0410',ProbeSetID2='ILMN_2677092',CellID2='',rank='0')) | [**0.600 79**](javascript:showCorrelationPlot2(db='Illum_Retina_BXD_RankInv0410',ProbeSetID='ILMN_2728431',CellID='',db2='Illum_Retina_BXD_RankInv0410',ProbeSetID2='ILMN_2624938',CellID2='',rank='0')) | [**0.559 79**](javascript:showCorrelationPlot2(db='Illum_Retina_BXD_RankInv0410',ProbeSetID='ILMN_2728431',CellID='',db2='Illum_Retina_BXD_RankInv0410',ProbeSetID2='ILMN_2847269',CellID2='',rank='0')) | [**0.691 79**](javascript:showCorrelationPlot2(db='Illum_Retina_BXD_RankInv0410',ProbeSetID='ILMN_2728431',CellID='',db2='Illum_Retina_BXD_RankInv0410',ProbeSetID2='ILMN_2839682',CellID2='',rank='0')) | [**0.543 79**](javascript:showCorrelationPlot2(db='Illum_Retina_BXD_RankInv0410',ProbeSetID='ILMN_2728431',CellID='',db2='Illum_Retina_BXD_RankInv0410',ProbeSetID2='ILMN_3147135',CellID2='',rank='0')) | [**0.534 79**](javascript:showCorrelationPlot2(db='Illum_Retina_BXD_RankInv0410',ProbeSetID='ILMN_2728431',CellID='',db2='Illum_Retina_BXD_RankInv0410',ProbeSetID2='ILMN_1255823',CellID2='',rank='0')) | [**0.484 79**](javascript:showCorrelationPlot2(db='Illum_Retina_BXD_RankInv0410',ProbeSetID='ILMN_2728431',CellID='',db2='Illum_Retina_BXD_RankInv0410',ProbeSetID2='ILMN_2626143',CellID2='',rank='0')) | [**0.723 79**](javascript:showCorrelationPlot2(db='Illum_Retina_BXD_RankInv0410',ProbeSetID='ILMN_2728431',CellID='',db2='Illum_Retina_BXD_RankInv0410',ProbeSetID2='ILMN_1234930',CellID2='',rank='0')) | [**0.567 79**](javascript:showCorrelationPlot2(db='Illum_Retina_BXD_RankInv0410',ProbeSetID='ILMN_2728431',CellID='',db2='Illum_Retina_BXD_RankInv0410',ProbeSetID2='ILMN_1249408',CellID2='',rank='0')) | [**0.588 79**](javascript:showCorrelationPlot2(db='Illum_Retina_BXD_RankInv0410',ProbeSetID='ILMN_2728431',CellID='',db2='Illum_Retina_BXD_RankInv0410',ProbeSetID2='ILMN_2971559',CellID2='',rank='0')) | [**0.382 79**](javascript:showCorrelationPlot2(db='Illum_Retina_BXD_RankInv0410',ProbeSetID='ILMN_2728431',CellID='',db2='Illum_Retina_BXD_RankInv0410',ProbeSetID2='ILMN_1258455',CellID2='',rank='0')) | [**0.633 79**](javascript:showCorrelationPlot2(db='Illum_Retina_BXD_RankInv0410',ProbeSetID='ILMN_2728431',CellID='',db2='Illum_Retina_BXD_RankInv0410',ProbeSetID2='ILMN_2691613',CellID2='',rank='0')) | [**0.599 79**](javascript:showCorrelationPlot2(db='Illum_Retina_BXD_RankInv0410',ProbeSetID='ILMN_2728431',CellID='',db2='Illum_Retina_BXD_RankInv0410',ProbeSetID2='ILMN_1253600',CellID2='',rank='0')) | [**0.552 79**](javascript:showCorrelationPlot2(db='Illum_Retina_BXD_RankInv0410',ProbeSetID='ILMN_2728431',CellID='',db2='Illum_Retina_BXD_RankInv0410',ProbeSetID2='ILMN_2991545',CellID2='',rank='0')) | [**0.495 79**](javascript:showCorrelationPlot2(db='Illum_Retina_BXD_RankInv0410',ProbeSetID='ILMN_2728431',CellID='',db2='Illum_Retina_BXD_RankInv0410',ProbeSetID2='ILMN_2623591',CellID2='',rank='0')) | [**0.472 79**](javascript:showCorrelationPlot2(db='Illum_Retina_BXD_RankInv0410',ProbeSetID='ILMN_2728431',CellID='',db2='Illum_Retina_BXD_RankInv0410',ProbeSetID2='ILMN_2603568',CellID2='',rank='0')) | [**0.486 79**](javascript:showCorrelationPlot2(db='Illum_Retina_BXD_RankInv0410',ProbeSetID='ILMN_2728431',CellID='',db2='Illum_Retina_BXD_RankInv0410',ProbeSetID2='ILMN_2992541',CellID2='',rank='0')) | [**0.583 79**](javascript:showCorrelationPlot2(db='Illum_Retina_BXD_RankInv0410',ProbeSetID='ILMN_2728431',CellID='',db2='Illum_Retina_BXD_RankInv0410',ProbeSetID2='ILMN_2792868',CellID2='',rank='0')) | [**0.447 79**](javascript:showCorrelationPlot2(db='Illum_Retina_BXD_RankInv0410',ProbeSetID='ILMN_2728431',CellID='',db2='Illum_Retina_BXD_RankInv0410',ProbeSetID2='ILMN_3129497',CellID2='',rank='0')) | [**0.658 79**](javascript:showCorrelationPlot2(db='Illum_Retina_BXD_RankInv0410',ProbeSetID='ILMN_2728431',CellID='',db2='Illum_Retina_BXD_RankInv0410',ProbeSetID2='ILMN_1224034',CellID2='',rank='0')) | [**0.362 79**](javascript:showCorrelationPlot2(db='Illum_Retina_BXD_RankInv0410',ProbeSetID='ILMN_2728431',CellID='',db2='Illum_Retina_BXD_RankInv0410',ProbeSetID2='ILMN_2639819',CellID2='',rank='0')) | [**0.510 79**](javascript:showCorrelationPlot2(db='Illum_Retina_BXD_RankInv0410',ProbeSetID='ILMN_2728431',CellID='',db2='Illum_Retina_BXD_RankInv0410',ProbeSetID2='ILMN_2994779',CellID2='',rank='0')) | [**0.642 79**](javascript:showCorrelationPlot2(db='Illum_Retina_BXD_RankInv0410',ProbeSetID='ILMN_2728431',CellID='',db2='Illum_Retina_BXD_RankInv0410',ProbeSetID2='ILMN_2625047',CellID2='',rank='0')) | [**0.445 79**](javascript:showCorrelationPlot2(db='Illum_Retina_BXD_RankInv0410',ProbeSetID='ILMN_2728431',CellID='',db2='Illum_Retina_BXD_RankInv0410',ProbeSetID2='ILMN_2808186',CellID2='',rank='0')) | [**0.449 79**](javascript:showCorrelationPlot2(db='Illum_Retina_BXD_RankInv0410',ProbeSetID='ILMN_2728431',CellID='',db2='Illum_Retina_BXD_RankInv0410',ProbeSetID2='ILMN_2689056',CellID2='',rank='0')) | [**0.390 79**](javascript:showCorrelationPlot2(db='Illum_Retina_BXD_RankInv0410',ProbeSetID='ILMN_2728431',CellID='',db2='Illum_Retina_BXD_RankInv0410',ProbeSetID2='ILMN_2648386',CellID2='',rank='0')) | [**0.426 79**](javascript:showCorrelationPlot2(db='Illum_Retina_BXD_RankInv0410',ProbeSetID='ILMN_2728431',CellID='',db2='Illum_Retina_BXD_RankInv0410',ProbeSetID2='ILMN_2869225',CellID2='',rank='0')) | [***n* 79**](javascript:showDatabase2('Illum_Retina_BXD_RankInv0410','ILMN_2728431','')) | [**0.462 79**](javascript:showCorrelationPlot2(db='Illum_Retina_BXD_RankInv0410',ProbeSetID='ILMN_2728431',CellID='',db2='Illum_Retina_BXD_RankInv0410',ProbeSetID2='ILMN_3094608',CellID2='',rank='1')) | [**0.495 79**](javascript:showCorrelationPlot2(db='Illum_Retina_BXD_RankInv0410',ProbeSetID='ILMN_2728431',CellID='',db2='Illum_Retina_BXD_RankInv0410',ProbeSetID2='ILMN_2896528',CellID2='',rank='1')) | [**0.442 79**](javascript:showCorrelationPlot2(db='Illum_Retina_BXD_RankInv0410',ProbeSetID='ILMN_2728431',CellID='',db2='Illum_Retina_BXD_RankInv0410',ProbeSetID2='ILMN_2701750',CellID2='',rank='1')) | [**0.514 79**](javascript:showCorrelationPlot2(db='Illum_Retina_BXD_RankInv0410',ProbeSetID='ILMN_2728431',CellID='',db2='Illum_Retina_BXD_RankInv0410',ProbeSetID2='ILMN_1239448',CellID2='',rank='1')) | [**-0.520 79**](javascript:showCorrelationPlot2(db='Illum_Retina_BXD_RankInv0410',ProbeSetID='ILMN_2728431',CellID='',db2='Illum_Retina_BXD_RankInv0410',ProbeSetID2='ILMN_2583882',CellID2='',rank='1')) | [**0.368 79**](javascript:showCorrelationPlot2(db='Illum_Retina_BXD_RankInv0410',ProbeSetID='ILMN_2728431',CellID='',db2='Illum_Retina_BXD_RankInv0410',ProbeSetID2='ILMN_2915166',CellID2='',rank='1')) | [**0.299 79**](javascript:showCorrelationPlot2(db='Illum_Retina_BXD_RankInv0410',ProbeSetID='ILMN_2728431',CellID='',db2='Illum_Retina_BXD_RankInv0410',ProbeSetID2='ILMN_2702997',CellID2='',rank='1')) | [**0.281 79**](javascript:showCorrelationPlot2(db='Illum_Retina_BXD_RankInv0410',ProbeSetID='ILMN_2728431',CellID='',db2='Illum_Retina_BXD_RankInv0410',ProbeSetID2='ILMN_2435206',CellID2='',rank='1')) | [**0.463 79**](javascript:showCorrelationPlot2(db='Illum_Retina_BXD_RankInv0410',ProbeSetID='ILMN_2728431',CellID='',db2='Illum_Retina_BXD_RankInv0410',ProbeSetID2='ILMN_2789888',CellID2='',rank='1')) | [**0.448 79**](javascript:showCorrelationPlot2(db='Illum_Retina_BXD_RankInv0410',ProbeSetID='ILMN_2728431',CellID='',db2='Illum_Retina_BXD_RankInv0410',ProbeSetID2='ILMN_1218901',CellID2='',rank='1')) | [**0.532 79**](javascript:showCorrelationPlot2(db='Illum_Retina_BXD_RankInv0410',ProbeSetID='ILMN_2728431',CellID='',db2='Illum_Retina_BXD_RankInv0410',ProbeSetID2='ILMN_1254734',CellID2='',rank='1')) | [**0.562 79**](javascript:showCorrelationPlot2(db='Illum_Retina_BXD_RankInv0410',ProbeSetID='ILMN_2728431',CellID='',db2='Illum_Retina_BXD_RankInv0410',ProbeSetID2='ILMN_3008924',CellID2='',rank='1')) | [**0.570 79**](javascript:showCorrelationPlot2(db='Illum_Retina_BXD_RankInv0410',ProbeSetID='ILMN_2728431',CellID='',db2='Illum_Retina_BXD_RankInv0410',ProbeSetID2='ILMN_2628175',CellID2='',rank='1')) | [**0.557 79**](javascript:showCorrelationPlot2(db='Illum_Retina_BXD_RankInv0410',ProbeSetID='ILMN_2728431',CellID='',db2='Illum_Retina_BXD_RankInv0410',ProbeSetID2='ILMN_2891573',CellID2='',rank='1')) | [**0.528 79**](javascript:showCorrelationPlot2(db='Illum_Retina_BXD_RankInv0410',ProbeSetID='ILMN_2728431',CellID='',db2='Illum_Retina_BXD_RankInv0410',ProbeSetID2='ILMN_1220846',CellID2='',rank='1')) | [**0.465 79**](javascript:showCorrelationPlot2(db='Illum_Retina_BXD_RankInv0410',ProbeSetID='ILMN_2728431',CellID='',db2='Illum_Retina_BXD_RankInv0410',ProbeSetID2='ILMN_1241962',CellID2='',rank='1')) | [**0.357 79**](javascript:showCorrelationPlot2(db='Illum_Retina_BXD_RankInv0410',ProbeSetID='ILMN_2728431',CellID='',db2='Illum_Retina_BXD_RankInv0410',ProbeSetID2='ILMN_3163027',CellID2='',rank='1')) | [**0.366 79**](javascript:showCorrelationPlot2(db='Illum_Retina_BXD_RankInv0410',ProbeSetID='ILMN_2728431',CellID='',db2='Illum_Retina_BXD_RankInv0410',ProbeSetID2='ILMN_2775098',CellID2='',rank='1')) | [**0.486 79**](javascript:showCorrelationPlot2(db='Illum_Retina_BXD_RankInv0410',ProbeSetID='ILMN_2728431',CellID='',db2='Illum_Retina_BXD_RankInv0410',ProbeSetID2='ILMN_2752552',CellID2='',rank='1')) | [**0.506 79**](javascript:showCorrelationPlot2(db='Illum_Retina_BXD_RankInv0410',ProbeSetID='ILMN_2728431',CellID='',db2='Illum_Retina_BXD_RankInv0410',ProbeSetID2='ILMN_2730293',CellID2='',rank='1')) | [**0.469 79**](javascript:showCorrelationPlot2(db='Illum_Retina_BXD_RankInv0410',ProbeSetID='ILMN_2728431',CellID='',db2='Illum_Retina_BXD_RankInv0410',ProbeSetID2='ILMN_2680872',CellID2='',rank='1')) | [**0.172 79**](javascript:showCorrelationPlot2(db='Illum_Retina_BXD_RankInv0410',ProbeSetID='ILMN_2728431',CellID='',db2='Illum_Retina_BXD_RankInv0410',ProbeSetID2='ILMN_2768533',CellID2='',rank='1')) | [**-0.029 79**](javascript:showCorrelationPlot2(db='Illum_Retina_BXD_RankInv0410',ProbeSetID='ILMN_2728431',CellID='',db2='Illum_Retina_BXD_RankInv0410',ProbeSetID2='ILMN_3121255',CellID2='',rank='1')) | [**-0.356 79**](javascript:showCorrelationPlot2(db='Illum_Retina_BXD_RankInv0410',ProbeSetID='ILMN_2728431',CellID='',db2='Illum_Retina_BXD_RankInv0410',ProbeSetID2='ILMN_2484527',CellID2='',rank='1')) | [**0.172 79**](javascript:showCorrelationPlot2(db='Illum_Retina_BXD_RankInv0410',ProbeSetID='ILMN_2728431',CellID='',db2='Illum_Retina_BXD_RankInv0410',ProbeSetID2='ILMN_3045723',CellID2='',rank='1')) | [**0.101 79**](javascript:showCorrelationPlot2(db='Illum_Retina_BXD_RankInv0410',ProbeSetID='ILMN_2728431',CellID='',db2='Illum_Retina_BXD_RankInv0410',ProbeSetID2='ILMN_2486573',CellID2='',rank='1')) |
|  | [**Trait 31: ILMN_3094608**](javascript:showDatabase2('Illum_Retina_BXD_RankInv0410','ILMN_3094608','');)  Symbol: Rdbp  Rdbp | [**0.644 79**](javascript:showCorrelationPlot2(db='Illum_Retina_BXD_RankInv0410',ProbeSetID='ILMN_3094608',CellID='',db2='Illum_Retina_BXD_RankInv0410',ProbeSetID2='ILMN_2450384',CellID2='',rank='0')) | [**0.778 79**](javascript:showCorrelationPlot2(db='Illum_Retina_BXD_RankInv0410',ProbeSetID='ILMN_3094608',CellID='',db2='Illum_Retina_BXD_RankInv0410',ProbeSetID2='ILMN_3068754',CellID2='',rank='0')) | [**0.637 79**](javascript:showCorrelationPlot2(db='Illum_Retina_BXD_RankInv0410',ProbeSetID='ILMN_3094608',CellID='',db2='Illum_Retina_BXD_RankInv0410',ProbeSetID2='ILMN_2677092',CellID2='',rank='0')) | [**0.623 79**](javascript:showCorrelationPlot2(db='Illum_Retina_BXD_RankInv0410',ProbeSetID='ILMN_3094608',CellID='',db2='Illum_Retina_BXD_RankInv0410',ProbeSetID2='ILMN_2624938',CellID2='',rank='0')) | [**0.374 79**](javascript:showCorrelationPlot2(db='Illum_Retina_BXD_RankInv0410',ProbeSetID='ILMN_3094608',CellID='',db2='Illum_Retina_BXD_RankInv0410',ProbeSetID2='ILMN_2847269',CellID2='',rank='0')) | [**0.554 79**](javascript:showCorrelationPlot2(db='Illum_Retina_BXD_RankInv0410',ProbeSetID='ILMN_3094608',CellID='',db2='Illum_Retina_BXD_RankInv0410',ProbeSetID2='ILMN_2839682',CellID2='',rank='0')) | [**0.465 79**](javascript:showCorrelationPlot2(db='Illum_Retina_BXD_RankInv0410',ProbeSetID='ILMN_3094608',CellID='',db2='Illum_Retina_BXD_RankInv0410',ProbeSetID2='ILMN_3147135',CellID2='',rank='0')) | [**0.448 79**](javascript:showCorrelationPlot2(db='Illum_Retina_BXD_RankInv0410',ProbeSetID='ILMN_3094608',CellID='',db2='Illum_Retina_BXD_RankInv0410',ProbeSetID2='ILMN_1255823',CellID2='',rank='0')) | [**0.642 79**](javascript:showCorrelationPlot2(db='Illum_Retina_BXD_RankInv0410',ProbeSetID='ILMN_3094608',CellID='',db2='Illum_Retina_BXD_RankInv0410',ProbeSetID2='ILMN_2626143',CellID2='',rank='0')) | [**0.543 79**](javascript:showCorrelationPlot2(db='Illum_Retina_BXD_RankInv0410',ProbeSetID='ILMN_3094608',CellID='',db2='Illum_Retina_BXD_RankInv0410',ProbeSetID2='ILMN_1234930',CellID2='',rank='0')) | [**0.457 79**](javascript:showCorrelationPlot2(db='Illum_Retina_BXD_RankInv0410',ProbeSetID='ILMN_3094608',CellID='',db2='Illum_Retina_BXD_RankInv0410',ProbeSetID2='ILMN_1249408',CellID2='',rank='0')) | [**0.405 79**](javascript:showCorrelationPlot2(db='Illum_Retina_BXD_RankInv0410',ProbeSetID='ILMN_3094608',CellID='',db2='Illum_Retina_BXD_RankInv0410',ProbeSetID2='ILMN_2971559',CellID2='',rank='0')) | [**0.511 79**](javascript:showCorrelationPlot2(db='Illum_Retina_BXD_RankInv0410',ProbeSetID='ILMN_3094608',CellID='',db2='Illum_Retina_BXD_RankInv0410',ProbeSetID2='ILMN_1258455',CellID2='',rank='0')) | [**0.430 79**](javascript:showCorrelationPlot2(db='Illum_Retina_BXD_RankInv0410',ProbeSetID='ILMN_3094608',CellID='',db2='Illum_Retina_BXD_RankInv0410',ProbeSetID2='ILMN_2691613',CellID2='',rank='0')) | [**0.604 79**](javascript:showCorrelationPlot2(db='Illum_Retina_BXD_RankInv0410',ProbeSetID='ILMN_3094608',CellID='',db2='Illum_Retina_BXD_RankInv0410',ProbeSetID2='ILMN_1253600',CellID2='',rank='0')) | [**0.524 79**](javascript:showCorrelationPlot2(db='Illum_Retina_BXD_RankInv0410',ProbeSetID='ILMN_3094608',CellID='',db2='Illum_Retina_BXD_RankInv0410',ProbeSetID2='ILMN_2991545',CellID2='',rank='0')) | [**0.608 79**](javascript:showCorrelationPlot2(db='Illum_Retina_BXD_RankInv0410',ProbeSetID='ILMN_3094608',CellID='',db2='Illum_Retina_BXD_RankInv0410',ProbeSetID2='ILMN_2623591',CellID2='',rank='0')) | [**0.503 79**](javascript:showCorrelationPlot2(db='Illum_Retina_BXD_RankInv0410',ProbeSetID='ILMN_3094608',CellID='',db2='Illum_Retina_BXD_RankInv0410',ProbeSetID2='ILMN_2603568',CellID2='',rank='0')) | [**0.443 79**](javascript:showCorrelationPlot2(db='Illum_Retina_BXD_RankInv0410',ProbeSetID='ILMN_3094608',CellID='',db2='Illum_Retina_BXD_RankInv0410',ProbeSetID2='ILMN_2992541',CellID2='',rank='0')) | [**0.376 79**](javascript:showCorrelationPlot2(db='Illum_Retina_BXD_RankInv0410',ProbeSetID='ILMN_3094608',CellID='',db2='Illum_Retina_BXD_RankInv0410',ProbeSetID2='ILMN_2792868',CellID2='',rank='0')) | [**0.492 79**](javascript:showCorrelationPlot2(db='Illum_Retina_BXD_RankInv0410',ProbeSetID='ILMN_3094608',CellID='',db2='Illum_Retina_BXD_RankInv0410',ProbeSetID2='ILMN_3129497',CellID2='',rank='0')) | [**0.384 79**](javascript:showCorrelationPlot2(db='Illum_Retina_BXD_RankInv0410',ProbeSetID='ILMN_3094608',CellID='',db2='Illum_Retina_BXD_RankInv0410',ProbeSetID2='ILMN_1224034',CellID2='',rank='0')) | [**0.585 79**](javascript:showCorrelationPlot2(db='Illum_Retina_BXD_RankInv0410',ProbeSetID='ILMN_3094608',CellID='',db2='Illum_Retina_BXD_RankInv0410',ProbeSetID2='ILMN_2639819',CellID2='',rank='0')) | [**0.388 79**](javascript:showCorrelationPlot2(db='Illum_Retina_BXD_RankInv0410',ProbeSetID='ILMN_3094608',CellID='',db2='Illum_Retina_BXD_RankInv0410',ProbeSetID2='ILMN_2994779',CellID2='',rank='0')) | [**0.454 79**](javascript:showCorrelationPlot2(db='Illum_Retina_BXD_RankInv0410',ProbeSetID='ILMN_3094608',CellID='',db2='Illum_Retina_BXD_RankInv0410',ProbeSetID2='ILMN_2625047',CellID2='',rank='0')) | [**0.584 79**](javascript:showCorrelationPlot2(db='Illum_Retina_BXD_RankInv0410',ProbeSetID='ILMN_3094608',CellID='',db2='Illum_Retina_BXD_RankInv0410',ProbeSetID2='ILMN_2808186',CellID2='',rank='0')) | [**0.724 79**](javascript:showCorrelationPlot2(db='Illum_Retina_BXD_RankInv0410',ProbeSetID='ILMN_3094608',CellID='',db2='Illum_Retina_BXD_RankInv0410',ProbeSetID2='ILMN_2689056',CellID2='',rank='0')) | [**0.459 79**](javascript:showCorrelationPlot2(db='Illum_Retina_BXD_RankInv0410',ProbeSetID='ILMN_3094608',CellID='',db2='Illum_Retina_BXD_RankInv0410',ProbeSetID2='ILMN_2648386',CellID2='',rank='0')) | [**0.472 79**](javascript:showCorrelationPlot2(db='Illum_Retina_BXD_RankInv0410',ProbeSetID='ILMN_3094608',CellID='',db2='Illum_Retina_BXD_RankInv0410',ProbeSetID2='ILMN_2869225',CellID2='',rank='0')) | [**0.373 79**](javascript:showCorrelationPlot2(db='Illum_Retina_BXD_RankInv0410',ProbeSetID='ILMN_3094608',CellID='',db2='Illum_Retina_BXD_RankInv0410',ProbeSetID2='ILMN_2728431',CellID2='',rank='0')) | [***n* 79**](javascript:showDatabase2('Illum_Retina_BXD_RankInv0410','ILMN_3094608','')) | [**0.364 79**](javascript:showCorrelationPlot2(db='Illum_Retina_BXD_RankInv0410',ProbeSetID='ILMN_3094608',CellID='',db2='Illum_Retina_BXD_RankInv0410',ProbeSetID2='ILMN_2896528',CellID2='',rank='1')) | [**0.464 79**](javascript:showCorrelationPlot2(db='Illum_Retina_BXD_RankInv0410',ProbeSetID='ILMN_3094608',CellID='',db2='Illum_Retina_BXD_RankInv0410',ProbeSetID2='ILMN_2701750',CellID2='',rank='1')) | [**0.580 79**](javascript:showCorrelationPlot2(db='Illum_Retina_BXD_RankInv0410',ProbeSetID='ILMN_3094608',CellID='',db2='Illum_Retina_BXD_RankInv0410',ProbeSetID2='ILMN_1239448',CellID2='',rank='1')) | [**-0.385 79**](javascript:showCorrelationPlot2(db='Illum_Retina_BXD_RankInv0410',ProbeSetID='ILMN_3094608',CellID='',db2='Illum_Retina_BXD_RankInv0410',ProbeSetID2='ILMN_2583882',CellID2='',rank='1')) | [**0.429 79**](javascript:showCorrelationPlot2(db='Illum_Retina_BXD_RankInv0410',ProbeSetID='ILMN_3094608',CellID='',db2='Illum_Retina_BXD_RankInv0410',ProbeSetID2='ILMN_2915166',CellID2='',rank='1')) | [**0.550 79**](javascript:showCorrelationPlot2(db='Illum_Retina_BXD_RankInv0410',ProbeSetID='ILMN_3094608',CellID='',db2='Illum_Retina_BXD_RankInv0410',ProbeSetID2='ILMN_2702997',CellID2='',rank='1')) | [**0.548 79**](javascript:showCorrelationPlot2(db='Illum_Retina_BXD_RankInv0410',ProbeSetID='ILMN_3094608',CellID='',db2='Illum_Retina_BXD_RankInv0410',ProbeSetID2='ILMN_2435206',CellID2='',rank='1')) | [**0.649 79**](javascript:showCorrelationPlot2(db='Illum_Retina_BXD_RankInv0410',ProbeSetID='ILMN_3094608',CellID='',db2='Illum_Retina_BXD_RankInv0410',ProbeSetID2='ILMN_2789888',CellID2='',rank='1')) | [**0.595 79**](javascript:showCorrelationPlot2(db='Illum_Retina_BXD_RankInv0410',ProbeSetID='ILMN_3094608',CellID='',db2='Illum_Retina_BXD_RankInv0410',ProbeSetID2='ILMN_1218901',CellID2='',rank='1')) | [**0.448 79**](javascript:showCorrelationPlot2(db='Illum_Retina_BXD_RankInv0410',ProbeSetID='ILMN_3094608',CellID='',db2='Illum_Retina_BXD_RankInv0410',ProbeSetID2='ILMN_1254734',CellID2='',rank='1')) | [**0.538 79**](javascript:showCorrelationPlot2(db='Illum_Retina_BXD_RankInv0410',ProbeSetID='ILMN_3094608',CellID='',db2='Illum_Retina_BXD_RankInv0410',ProbeSetID2='ILMN_3008924',CellID2='',rank='1')) | [**0.636 79**](javascript:showCorrelationPlot2(db='Illum_Retina_BXD_RankInv0410',ProbeSetID='ILMN_3094608',CellID='',db2='Illum_Retina_BXD_RankInv0410',ProbeSetID2='ILMN_2628175',CellID2='',rank='1')) | [**0.533 79**](javascript:showCorrelationPlot2(db='Illum_Retina_BXD_RankInv0410',ProbeSetID='ILMN_3094608',CellID='',db2='Illum_Retina_BXD_RankInv0410',ProbeSetID2='ILMN_2891573',CellID2='',rank='1')) | [**0.642 79**](javascript:showCorrelationPlot2(db='Illum_Retina_BXD_RankInv0410',ProbeSetID='ILMN_3094608',CellID='',db2='Illum_Retina_BXD_RankInv0410',ProbeSetID2='ILMN_1220846',CellID2='',rank='1')) | [**0.290 79**](javascript:showCorrelationPlot2(db='Illum_Retina_BXD_RankInv0410',ProbeSetID='ILMN_3094608',CellID='',db2='Illum_Retina_BXD_RankInv0410',ProbeSetID2='ILMN_1241962',CellID2='',rank='1')) | [**0.311 79**](javascript:showCorrelationPlot2(db='Illum_Retina_BXD_RankInv0410',ProbeSetID='ILMN_3094608',CellID='',db2='Illum_Retina_BXD_RankInv0410',ProbeSetID2='ILMN_3163027',CellID2='',rank='1')) | [**0.323 79**](javascript:showCorrelationPlot2(db='Illum_Retina_BXD_RankInv0410',ProbeSetID='ILMN_3094608',CellID='',db2='Illum_Retina_BXD_RankInv0410',ProbeSetID2='ILMN_2775098',CellID2='',rank='1')) | [**0.634 79**](javascript:showCorrelationPlot2(db='Illum_Retina_BXD_RankInv0410',ProbeSetID='ILMN_3094608',CellID='',db2='Illum_Retina_BXD_RankInv0410',ProbeSetID2='ILMN_2752552',CellID2='',rank='1')) | [**0.434 79**](javascript:showCorrelationPlot2(db='Illum_Retina_BXD_RankInv0410',ProbeSetID='ILMN_3094608',CellID='',db2='Illum_Retina_BXD_RankInv0410',ProbeSetID2='ILMN_2730293',CellID2='',rank='1')) | [**0.512 79**](javascript:showCorrelationPlot2(db='Illum_Retina_BXD_RankInv0410',ProbeSetID='ILMN_3094608',CellID='',db2='Illum_Retina_BXD_RankInv0410',ProbeSetID2='ILMN_2680872',CellID2='',rank='1')) | [**0.190 79**](javascript:showCorrelationPlot2(db='Illum_Retina_BXD_RankInv0410',ProbeSetID='ILMN_3094608',CellID='',db2='Illum_Retina_BXD_RankInv0410',ProbeSetID2='ILMN_2768533',CellID2='',rank='1')) | [**-0.263 79**](javascript:showCorrelationPlot2(db='Illum_Retina_BXD_RankInv0410',ProbeSetID='ILMN_3094608',CellID='',db2='Illum_Retina_BXD_RankInv0410',ProbeSetID2='ILMN_3121255',CellID2='',rank='1')) | [**-0.345 79**](javascript:showCorrelationPlot2(db='Illum_Retina_BXD_RankInv0410',ProbeSetID='ILMN_3094608',CellID='',db2='Illum_Retina_BXD_RankInv0410',ProbeSetID2='ILMN_2484527',CellID2='',rank='1')) | [**0.034 79**](javascript:showCorrelationPlot2(db='Illum_Retina_BXD_RankInv0410',ProbeSetID='ILMN_3094608',CellID='',db2='Illum_Retina_BXD_RankInv0410',ProbeSetID2='ILMN_3045723',CellID2='',rank='1')) | [**0.153 79**](javascript:showCorrelationPlot2(db='Illum_Retina_BXD_RankInv0410',ProbeSetID='ILMN_3094608',CellID='',db2='Illum_Retina_BXD_RankInv0410',ProbeSetID2='ILMN_2486573',CellID2='',rank='1')) |
|  | [**Trait 32: ILMN_2896528**](javascript:showDatabase2('Illum_Retina_BXD_RankInv0410','ILMN_2896528','');)  Symbol: Mre11a  Mre11a | [**0.642 79**](javascript:showCorrelationPlot2(db='Illum_Retina_BXD_RankInv0410',ProbeSetID='ILMN_2896528',CellID='',db2='Illum_Retina_BXD_RankInv0410',ProbeSetID2='ILMN_2450384',CellID2='',rank='0')) | [**0.403 79**](javascript:showCorrelationPlot2(db='Illum_Retina_BXD_RankInv0410',ProbeSetID='ILMN_2896528',CellID='',db2='Illum_Retina_BXD_RankInv0410',ProbeSetID2='ILMN_3068754',CellID2='',rank='0')) | [**0.405 79**](javascript:showCorrelationPlot2(db='Illum_Retina_BXD_RankInv0410',ProbeSetID='ILMN_2896528',CellID='',db2='Illum_Retina_BXD_RankInv0410',ProbeSetID2='ILMN_2677092',CellID2='',rank='0')) | [**0.603 79**](javascript:showCorrelationPlot2(db='Illum_Retina_BXD_RankInv0410',ProbeSetID='ILMN_2896528',CellID='',db2='Illum_Retina_BXD_RankInv0410',ProbeSetID2='ILMN_2624938',CellID2='',rank='0')) | [**0.595 79**](javascript:showCorrelationPlot2(db='Illum_Retina_BXD_RankInv0410',ProbeSetID='ILMN_2896528',CellID='',db2='Illum_Retina_BXD_RankInv0410',ProbeSetID2='ILMN_2847269',CellID2='',rank='0')) | [**0.471 79**](javascript:showCorrelationPlot2(db='Illum_Retina_BXD_RankInv0410',ProbeSetID='ILMN_2896528',CellID='',db2='Illum_Retina_BXD_RankInv0410',ProbeSetID2='ILMN_2839682',CellID2='',rank='0')) | [**0.604 79**](javascript:showCorrelationPlot2(db='Illum_Retina_BXD_RankInv0410',ProbeSetID='ILMN_2896528',CellID='',db2='Illum_Retina_BXD_RankInv0410',ProbeSetID2='ILMN_3147135',CellID2='',rank='0')) | [**0.678 79**](javascript:showCorrelationPlot2(db='Illum_Retina_BXD_RankInv0410',ProbeSetID='ILMN_2896528',CellID='',db2='Illum_Retina_BXD_RankInv0410',ProbeSetID2='ILMN_1255823',CellID2='',rank='0')) | [**0.514 79**](javascript:showCorrelationPlot2(db='Illum_Retina_BXD_RankInv0410',ProbeSetID='ILMN_2896528',CellID='',db2='Illum_Retina_BXD_RankInv0410',ProbeSetID2='ILMN_2626143',CellID2='',rank='0')) | [**0.517 79**](javascript:showCorrelationPlot2(db='Illum_Retina_BXD_RankInv0410',ProbeSetID='ILMN_2896528',CellID='',db2='Illum_Retina_BXD_RankInv0410',ProbeSetID2='ILMN_1234930',CellID2='',rank='0')) | [**0.636 79**](javascript:showCorrelationPlot2(db='Illum_Retina_BXD_RankInv0410',ProbeSetID='ILMN_2896528',CellID='',db2='Illum_Retina_BXD_RankInv0410',ProbeSetID2='ILMN_1249408',CellID2='',rank='0')) | [**0.548 79**](javascript:showCorrelationPlot2(db='Illum_Retina_BXD_RankInv0410',ProbeSetID='ILMN_2896528',CellID='',db2='Illum_Retina_BXD_RankInv0410',ProbeSetID2='ILMN_2971559',CellID2='',rank='0')) | [**0.370 79**](javascript:showCorrelationPlot2(db='Illum_Retina_BXD_RankInv0410',ProbeSetID='ILMN_2896528',CellID='',db2='Illum_Retina_BXD_RankInv0410',ProbeSetID2='ILMN_1258455',CellID2='',rank='0')) | [**0.697 79**](javascript:showCorrelationPlot2(db='Illum_Retina_BXD_RankInv0410',ProbeSetID='ILMN_2896528',CellID='',db2='Illum_Retina_BXD_RankInv0410',ProbeSetID2='ILMN_2691613',CellID2='',rank='0')) | [**0.376 79**](javascript:showCorrelationPlot2(db='Illum_Retina_BXD_RankInv0410',ProbeSetID='ILMN_2896528',CellID='',db2='Illum_Retina_BXD_RankInv0410',ProbeSetID2='ILMN_1253600',CellID2='',rank='0')) | [**0.437 79**](javascript:showCorrelationPlot2(db='Illum_Retina_BXD_RankInv0410',ProbeSetID='ILMN_2896528',CellID='',db2='Illum_Retina_BXD_RankInv0410',ProbeSetID2='ILMN_2991545',CellID2='',rank='0')) | [**0.308 79**](javascript:showCorrelationPlot2(db='Illum_Retina_BXD_RankInv0410',ProbeSetID='ILMN_2896528',CellID='',db2='Illum_Retina_BXD_RankInv0410',ProbeSetID2='ILMN_2623591',CellID2='',rank='0')) | [**0.428 79**](javascript:showCorrelationPlot2(db='Illum_Retina_BXD_RankInv0410',ProbeSetID='ILMN_2896528',CellID='',db2='Illum_Retina_BXD_RankInv0410',ProbeSetID2='ILMN_2603568',CellID2='',rank='0')) | [**0.619 79**](javascript:showCorrelationPlot2(db='Illum_Retina_BXD_RankInv0410',ProbeSetID='ILMN_2896528',CellID='',db2='Illum_Retina_BXD_RankInv0410',ProbeSetID2='ILMN_2992541',CellID2='',rank='0')) | [**0.566 79**](javascript:showCorrelationPlot2(db='Illum_Retina_BXD_RankInv0410',ProbeSetID='ILMN_2896528',CellID='',db2='Illum_Retina_BXD_RankInv0410',ProbeSetID2='ILMN_2792868',CellID2='',rank='0')) | [**0.400 79**](javascript:showCorrelationPlot2(db='Illum_Retina_BXD_RankInv0410',ProbeSetID='ILMN_2896528',CellID='',db2='Illum_Retina_BXD_RankInv0410',ProbeSetID2='ILMN_3129497',CellID2='',rank='0')) | [**0.407 79**](javascript:showCorrelationPlot2(db='Illum_Retina_BXD_RankInv0410',ProbeSetID='ILMN_2896528',CellID='',db2='Illum_Retina_BXD_RankInv0410',ProbeSetID2='ILMN_1224034',CellID2='',rank='0')) | [**0.457 79**](javascript:showCorrelationPlot2(db='Illum_Retina_BXD_RankInv0410',ProbeSetID='ILMN_2896528',CellID='',db2='Illum_Retina_BXD_RankInv0410',ProbeSetID2='ILMN_2639819',CellID2='',rank='0')) | [**0.586 79**](javascript:showCorrelationPlot2(db='Illum_Retina_BXD_RankInv0410',ProbeSetID='ILMN_2896528',CellID='',db2='Illum_Retina_BXD_RankInv0410',ProbeSetID2='ILMN_2994779',CellID2='',rank='0')) | [**0.589 79**](javascript:showCorrelationPlot2(db='Illum_Retina_BXD_RankInv0410',ProbeSetID='ILMN_2896528',CellID='',db2='Illum_Retina_BXD_RankInv0410',ProbeSetID2='ILMN_2625047',CellID2='',rank='0')) | [**0.192 79**](javascript:showCorrelationPlot2(db='Illum_Retina_BXD_RankInv0410',ProbeSetID='ILMN_2896528',CellID='',db2='Illum_Retina_BXD_RankInv0410',ProbeSetID2='ILMN_2808186',CellID2='',rank='0')) | [**0.472 79**](javascript:showCorrelationPlot2(db='Illum_Retina_BXD_RankInv0410',ProbeSetID='ILMN_2896528',CellID='',db2='Illum_Retina_BXD_RankInv0410',ProbeSetID2='ILMN_2689056',CellID2='',rank='0')) | [**0.572 79**](javascript:showCorrelationPlot2(db='Illum_Retina_BXD_RankInv0410',ProbeSetID='ILMN_2896528',CellID='',db2='Illum_Retina_BXD_RankInv0410',ProbeSetID2='ILMN_2648386',CellID2='',rank='0')) | [**0.666 79**](javascript:showCorrelationPlot2(db='Illum_Retina_BXD_RankInv0410',ProbeSetID='ILMN_2896528',CellID='',db2='Illum_Retina_BXD_RankInv0410',ProbeSetID2='ILMN_2869225',CellID2='',rank='0')) | [**0.550 79**](javascript:showCorrelationPlot2(db='Illum_Retina_BXD_RankInv0410',ProbeSetID='ILMN_2896528',CellID='',db2='Illum_Retina_BXD_RankInv0410',ProbeSetID2='ILMN_2728431',CellID2='',rank='0')) | [**0.393 79**](javascript:showCorrelationPlot2(db='Illum_Retina_BXD_RankInv0410',ProbeSetID='ILMN_2896528',CellID='',db2='Illum_Retina_BXD_RankInv0410',ProbeSetID2='ILMN_3094608',CellID2='',rank='0')) | [***n* 79**](javascript:showDatabase2('Illum_Retina_BXD_RankInv0410','ILMN_2896528','')) | [**0.370 79**](javascript:showCorrelationPlot2(db='Illum_Retina_BXD_RankInv0410',ProbeSetID='ILMN_2896528',CellID='',db2='Illum_Retina_BXD_RankInv0410',ProbeSetID2='ILMN_2701750',CellID2='',rank='1')) | [**0.434 79**](javascript:showCorrelationPlot2(db='Illum_Retina_BXD_RankInv0410',ProbeSetID='ILMN_2896528',CellID='',db2='Illum_Retina_BXD_RankInv0410',ProbeSetID2='ILMN_1239448',CellID2='',rank='1')) | [**-0.442 79**](javascript:showCorrelationPlot2(db='Illum_Retina_BXD_RankInv0410',ProbeSetID='ILMN_2896528',CellID='',db2='Illum_Retina_BXD_RankInv0410',ProbeSetID2='ILMN_2583882',CellID2='',rank='1')) | [**0.431 79**](javascript:showCorrelationPlot2(db='Illum_Retina_BXD_RankInv0410',ProbeSetID='ILMN_2896528',CellID='',db2='Illum_Retina_BXD_RankInv0410',ProbeSetID2='ILMN_2915166',CellID2='',rank='1')) | [**0.410 79**](javascript:showCorrelationPlot2(db='Illum_Retina_BXD_RankInv0410',ProbeSetID='ILMN_2896528',CellID='',db2='Illum_Retina_BXD_RankInv0410',ProbeSetID2='ILMN_2702997',CellID2='',rank='1')) | [**0.505 79**](javascript:showCorrelationPlot2(db='Illum_Retina_BXD_RankInv0410',ProbeSetID='ILMN_2896528',CellID='',db2='Illum_Retina_BXD_RankInv0410',ProbeSetID2='ILMN_2435206',CellID2='',rank='1')) | [**0.347 79**](javascript:showCorrelationPlot2(db='Illum_Retina_BXD_RankInv0410',ProbeSetID='ILMN_2896528',CellID='',db2='Illum_Retina_BXD_RankInv0410',ProbeSetID2='ILMN_2789888',CellID2='',rank='1')) | [**0.260 79**](javascript:showCorrelationPlot2(db='Illum_Retina_BXD_RankInv0410',ProbeSetID='ILMN_2896528',CellID='',db2='Illum_Retina_BXD_RankInv0410',ProbeSetID2='ILMN_1218901',CellID2='',rank='1')) | [**0.507 79**](javascript:showCorrelationPlot2(db='Illum_Retina_BXD_RankInv0410',ProbeSetID='ILMN_2896528',CellID='',db2='Illum_Retina_BXD_RankInv0410',ProbeSetID2='ILMN_1254734',CellID2='',rank='1')) | [**0.305 79**](javascript:showCorrelationPlot2(db='Illum_Retina_BXD_RankInv0410',ProbeSetID='ILMN_2896528',CellID='',db2='Illum_Retina_BXD_RankInv0410',ProbeSetID2='ILMN_3008924',CellID2='',rank='1')) | [**0.357 79**](javascript:showCorrelationPlot2(db='Illum_Retina_BXD_RankInv0410',ProbeSetID='ILMN_2896528',CellID='',db2='Illum_Retina_BXD_RankInv0410',ProbeSetID2='ILMN_2628175',CellID2='',rank='1')) | [**0.496 79**](javascript:showCorrelationPlot2(db='Illum_Retina_BXD_RankInv0410',ProbeSetID='ILMN_2896528',CellID='',db2='Illum_Retina_BXD_RankInv0410',ProbeSetID2='ILMN_2891573',CellID2='',rank='1')) | [**0.487 79**](javascript:showCorrelationPlot2(db='Illum_Retina_BXD_RankInv0410',ProbeSetID='ILMN_2896528',CellID='',db2='Illum_Retina_BXD_RankInv0410',ProbeSetID2='ILMN_1220846',CellID2='',rank='1')) | [**0.586 79**](javascript:showCorrelationPlot2(db='Illum_Retina_BXD_RankInv0410',ProbeSetID='ILMN_2896528',CellID='',db2='Illum_Retina_BXD_RankInv0410',ProbeSetID2='ILMN_1241962',CellID2='',rank='1')) | [**0.601 79**](javascript:showCorrelationPlot2(db='Illum_Retina_BXD_RankInv0410',ProbeSetID='ILMN_2896528',CellID='',db2='Illum_Retina_BXD_RankInv0410',ProbeSetID2='ILMN_3163027',CellID2='',rank='1')) | [**0.429 79**](javascript:showCorrelationPlot2(db='Illum_Retina_BXD_RankInv0410',ProbeSetID='ILMN_2896528',CellID='',db2='Illum_Retina_BXD_RankInv0410',ProbeSetID2='ILMN_2775098',CellID2='',rank='1')) | [**0.515 79**](javascript:showCorrelationPlot2(db='Illum_Retina_BXD_RankInv0410',ProbeSetID='ILMN_2896528',CellID='',db2='Illum_Retina_BXD_RankInv0410',ProbeSetID2='ILMN_2752552',CellID2='',rank='1')) | [**0.285 79**](javascript:showCorrelationPlot2(db='Illum_Retina_BXD_RankInv0410',ProbeSetID='ILMN_2896528',CellID='',db2='Illum_Retina_BXD_RankInv0410',ProbeSetID2='ILMN_2730293',CellID2='',rank='1')) | [**0.534 79**](javascript:showCorrelationPlot2(db='Illum_Retina_BXD_RankInv0410',ProbeSetID='ILMN_2896528',CellID='',db2='Illum_Retina_BXD_RankInv0410',ProbeSetID2='ILMN_2680872',CellID2='',rank='1')) | [**0.179 79**](javascript:showCorrelationPlot2(db='Illum_Retina_BXD_RankInv0410',ProbeSetID='ILMN_2896528',CellID='',db2='Illum_Retina_BXD_RankInv0410',ProbeSetID2='ILMN_2768533',CellID2='',rank='1')) | [**0.059 79**](javascript:showCorrelationPlot2(db='Illum_Retina_BXD_RankInv0410',ProbeSetID='ILMN_2896528',CellID='',db2='Illum_Retina_BXD_RankInv0410',ProbeSetID2='ILMN_3121255',CellID2='',rank='1')) | [**-0.114 79**](javascript:showCorrelationPlot2(db='Illum_Retina_BXD_RankInv0410',ProbeSetID='ILMN_2896528',CellID='',db2='Illum_Retina_BXD_RankInv0410',ProbeSetID2='ILMN_2484527',CellID2='',rank='1')) | [**0.247 79**](javascript:showCorrelationPlot2(db='Illum_Retina_BXD_RankInv0410',ProbeSetID='ILMN_2896528',CellID='',db2='Illum_Retina_BXD_RankInv0410',ProbeSetID2='ILMN_3045723',CellID2='',rank='1')) | [**0.122 79**](javascript:showCorrelationPlot2(db='Illum_Retina_BXD_RankInv0410',ProbeSetID='ILMN_2896528',CellID='',db2='Illum_Retina_BXD_RankInv0410',ProbeSetID2='ILMN_2486573',CellID2='',rank='1')) |
|  | [**Trait 33: ILMN_2701750**](javascript:showDatabase2('Illum_Retina_BXD_RankInv0410','ILMN_2701750','');)  Symbol: 2310061J03Rik  2310061J03Rik | [**0.640 79**](javascript:showCorrelationPlot2(db='Illum_Retina_BXD_RankInv0410',ProbeSetID='ILMN_2701750',CellID='',db2='Illum_Retina_BXD_RankInv0410',ProbeSetID2='ILMN_2450384',CellID2='',rank='0')) | [**0.510 79**](javascript:showCorrelationPlot2(db='Illum_Retina_BXD_RankInv0410',ProbeSetID='ILMN_2701750',CellID='',db2='Illum_Retina_BXD_RankInv0410',ProbeSetID2='ILMN_3068754',CellID2='',rank='0')) | [**0.566 79**](javascript:showCorrelationPlot2(db='Illum_Retina_BXD_RankInv0410',ProbeSetID='ILMN_2701750',CellID='',db2='Illum_Retina_BXD_RankInv0410',ProbeSetID2='ILMN_2677092',CellID2='',rank='0')) | [**0.376 79**](javascript:showCorrelationPlot2(db='Illum_Retina_BXD_RankInv0410',ProbeSetID='ILMN_2701750',CellID='',db2='Illum_Retina_BXD_RankInv0410',ProbeSetID2='ILMN_2624938',CellID2='',rank='0')) | [**0.434 79**](javascript:showCorrelationPlot2(db='Illum_Retina_BXD_RankInv0410',ProbeSetID='ILMN_2701750',CellID='',db2='Illum_Retina_BXD_RankInv0410',ProbeSetID2='ILMN_2847269',CellID2='',rank='0')) | [**0.409 79**](javascript:showCorrelationPlot2(db='Illum_Retina_BXD_RankInv0410',ProbeSetID='ILMN_2701750',CellID='',db2='Illum_Retina_BXD_RankInv0410',ProbeSetID2='ILMN_2839682',CellID2='',rank='0')) | [**0.371 79**](javascript:showCorrelationPlot2(db='Illum_Retina_BXD_RankInv0410',ProbeSetID='ILMN_2701750',CellID='',db2='Illum_Retina_BXD_RankInv0410',ProbeSetID2='ILMN_3147135',CellID2='',rank='0')) | [**0.384 79**](javascript:showCorrelationPlot2(db='Illum_Retina_BXD_RankInv0410',ProbeSetID='ILMN_2701750',CellID='',db2='Illum_Retina_BXD_RankInv0410',ProbeSetID2='ILMN_1255823',CellID2='',rank='0')) | [**0.396 79**](javascript:showCorrelationPlot2(db='Illum_Retina_BXD_RankInv0410',ProbeSetID='ILMN_2701750',CellID='',db2='Illum_Retina_BXD_RankInv0410',ProbeSetID2='ILMN_2626143',CellID2='',rank='0')) | [**0.439 79**](javascript:showCorrelationPlot2(db='Illum_Retina_BXD_RankInv0410',ProbeSetID='ILMN_2701750',CellID='',db2='Illum_Retina_BXD_RankInv0410',ProbeSetID2='ILMN_1234930',CellID2='',rank='0')) | [**0.393 79**](javascript:showCorrelationPlot2(db='Illum_Retina_BXD_RankInv0410',ProbeSetID='ILMN_2701750',CellID='',db2='Illum_Retina_BXD_RankInv0410',ProbeSetID2='ILMN_1249408',CellID2='',rank='0')) | [**0.371 79**](javascript:showCorrelationPlot2(db='Illum_Retina_BXD_RankInv0410',ProbeSetID='ILMN_2701750',CellID='',db2='Illum_Retina_BXD_RankInv0410',ProbeSetID2='ILMN_2971559',CellID2='',rank='0')) | [**0.707 79**](javascript:showCorrelationPlot2(db='Illum_Retina_BXD_RankInv0410',ProbeSetID='ILMN_2701750',CellID='',db2='Illum_Retina_BXD_RankInv0410',ProbeSetID2='ILMN_1258455',CellID2='',rank='0')) | [**0.247 79**](javascript:showCorrelationPlot2(db='Illum_Retina_BXD_RankInv0410',ProbeSetID='ILMN_2701750',CellID='',db2='Illum_Retina_BXD_RankInv0410',ProbeSetID2='ILMN_2691613',CellID2='',rank='0')) | [**0.356 79**](javascript:showCorrelationPlot2(db='Illum_Retina_BXD_RankInv0410',ProbeSetID='ILMN_2701750',CellID='',db2='Illum_Retina_BXD_RankInv0410',ProbeSetID2='ILMN_1253600',CellID2='',rank='0')) | [**0.386 79**](javascript:showCorrelationPlot2(db='Illum_Retina_BXD_RankInv0410',ProbeSetID='ILMN_2701750',CellID='',db2='Illum_Retina_BXD_RankInv0410',ProbeSetID2='ILMN_2991545',CellID2='',rank='0')) | [**0.289 79**](javascript:showCorrelationPlot2(db='Illum_Retina_BXD_RankInv0410',ProbeSetID='ILMN_2701750',CellID='',db2='Illum_Retina_BXD_RankInv0410',ProbeSetID2='ILMN_2623591',CellID2='',rank='0')) | [**0.544 79**](javascript:showCorrelationPlot2(db='Illum_Retina_BXD_RankInv0410',ProbeSetID='ILMN_2701750',CellID='',db2='Illum_Retina_BXD_RankInv0410',ProbeSetID2='ILMN_2603568',CellID2='',rank='0')) | [**0.267 79**](javascript:showCorrelationPlot2(db='Illum_Retina_BXD_RankInv0410',ProbeSetID='ILMN_2701750',CellID='',db2='Illum_Retina_BXD_RankInv0410',ProbeSetID2='ILMN_2992541',CellID2='',rank='0')) | [**0.652 79**](javascript:showCorrelationPlot2(db='Illum_Retina_BXD_RankInv0410',ProbeSetID='ILMN_2701750',CellID='',db2='Illum_Retina_BXD_RankInv0410',ProbeSetID2='ILMN_2792868',CellID2='',rank='0')) | [**0.420 79**](javascript:showCorrelationPlot2(db='Illum_Retina_BXD_RankInv0410',ProbeSetID='ILMN_2701750',CellID='',db2='Illum_Retina_BXD_RankInv0410',ProbeSetID2='ILMN_3129497',CellID2='',rank='0')) | [**0.537 79**](javascript:showCorrelationPlot2(db='Illum_Retina_BXD_RankInv0410',ProbeSetID='ILMN_2701750',CellID='',db2='Illum_Retina_BXD_RankInv0410',ProbeSetID2='ILMN_1224034',CellID2='',rank='0')) | [**0.502 79**](javascript:showCorrelationPlot2(db='Illum_Retina_BXD_RankInv0410',ProbeSetID='ILMN_2701750',CellID='',db2='Illum_Retina_BXD_RankInv0410',ProbeSetID2='ILMN_2639819',CellID2='',rank='0')) | [**0.265 79**](javascript:showCorrelationPlot2(db='Illum_Retina_BXD_RankInv0410',ProbeSetID='ILMN_2701750',CellID='',db2='Illum_Retina_BXD_RankInv0410',ProbeSetID2='ILMN_2994779',CellID2='',rank='0')) | [**0.408 79**](javascript:showCorrelationPlot2(db='Illum_Retina_BXD_RankInv0410',ProbeSetID='ILMN_2701750',CellID='',db2='Illum_Retina_BXD_RankInv0410',ProbeSetID2='ILMN_2625047',CellID2='',rank='0')) | [**0.519 79**](javascript:showCorrelationPlot2(db='Illum_Retina_BXD_RankInv0410',ProbeSetID='ILMN_2701750',CellID='',db2='Illum_Retina_BXD_RankInv0410',ProbeSetID2='ILMN_2808186',CellID2='',rank='0')) | [**0.328 79**](javascript:showCorrelationPlot2(db='Illum_Retina_BXD_RankInv0410',ProbeSetID='ILMN_2701750',CellID='',db2='Illum_Retina_BXD_RankInv0410',ProbeSetID2='ILMN_2689056',CellID2='',rank='0')) | [**0.467 79**](javascript:showCorrelationPlot2(db='Illum_Retina_BXD_RankInv0410',ProbeSetID='ILMN_2701750',CellID='',db2='Illum_Retina_BXD_RankInv0410',ProbeSetID2='ILMN_2648386',CellID2='',rank='0')) | [**0.272 79**](javascript:showCorrelationPlot2(db='Illum_Retina_BXD_RankInv0410',ProbeSetID='ILMN_2701750',CellID='',db2='Illum_Retina_BXD_RankInv0410',ProbeSetID2='ILMN_2869225',CellID2='',rank='0')) | [**0.383 79**](javascript:showCorrelationPlot2(db='Illum_Retina_BXD_RankInv0410',ProbeSetID='ILMN_2701750',CellID='',db2='Illum_Retina_BXD_RankInv0410',ProbeSetID2='ILMN_2728431',CellID2='',rank='0')) | [**0.445 79**](javascript:showCorrelationPlot2(db='Illum_Retina_BXD_RankInv0410',ProbeSetID='ILMN_2701750',CellID='',db2='Illum_Retina_BXD_RankInv0410',ProbeSetID2='ILMN_3094608',CellID2='',rank='0')) | [**0.293 79**](javascript:showCorrelationPlot2(db='Illum_Retina_BXD_RankInv0410',ProbeSetID='ILMN_2701750',CellID='',db2='Illum_Retina_BXD_RankInv0410',ProbeSetID2='ILMN_2896528',CellID2='',rank='0')) | [***n* 79**](javascript:showDatabase2('Illum_Retina_BXD_RankInv0410','ILMN_2701750','')) | [**0.311 79**](javascript:showCorrelationPlot2(db='Illum_Retina_BXD_RankInv0410',ProbeSetID='ILMN_2701750',CellID='',db2='Illum_Retina_BXD_RankInv0410',ProbeSetID2='ILMN_1239448',CellID2='',rank='1')) | [**-0.483 79**](javascript:showCorrelationPlot2(db='Illum_Retina_BXD_RankInv0410',ProbeSetID='ILMN_2701750',CellID='',db2='Illum_Retina_BXD_RankInv0410',ProbeSetID2='ILMN_2583882',CellID2='',rank='1')) | [**0.534 79**](javascript:showCorrelationPlot2(db='Illum_Retina_BXD_RankInv0410',ProbeSetID='ILMN_2701750',CellID='',db2='Illum_Retina_BXD_RankInv0410',ProbeSetID2='ILMN_2915166',CellID2='',rank='1')) | [**0.609 79**](javascript:showCorrelationPlot2(db='Illum_Retina_BXD_RankInv0410',ProbeSetID='ILMN_2701750',CellID='',db2='Illum_Retina_BXD_RankInv0410',ProbeSetID2='ILMN_2702997',CellID2='',rank='1')) | [**0.400 79**](javascript:showCorrelationPlot2(db='Illum_Retina_BXD_RankInv0410',ProbeSetID='ILMN_2701750',CellID='',db2='Illum_Retina_BXD_RankInv0410',ProbeSetID2='ILMN_2435206',CellID2='',rank='1')) | [**0.350 79**](javascript:showCorrelationPlot2(db='Illum_Retina_BXD_RankInv0410',ProbeSetID='ILMN_2701750',CellID='',db2='Illum_Retina_BXD_RankInv0410',ProbeSetID2='ILMN_2789888',CellID2='',rank='1')) | [**0.397 79**](javascript:showCorrelationPlot2(db='Illum_Retina_BXD_RankInv0410',ProbeSetID='ILMN_2701750',CellID='',db2='Illum_Retina_BXD_RankInv0410',ProbeSetID2='ILMN_1218901',CellID2='',rank='1')) | [**0.517 79**](javascript:showCorrelationPlot2(db='Illum_Retina_BXD_RankInv0410',ProbeSetID='ILMN_2701750',CellID='',db2='Illum_Retina_BXD_RankInv0410',ProbeSetID2='ILMN_1254734',CellID2='',rank='1')) | [**0.394 79**](javascript:showCorrelationPlot2(db='Illum_Retina_BXD_RankInv0410',ProbeSetID='ILMN_2701750',CellID='',db2='Illum_Retina_BXD_RankInv0410',ProbeSetID2='ILMN_3008924',CellID2='',rank='1')) | [**0.274 79**](javascript:showCorrelationPlot2(db='Illum_Retina_BXD_RankInv0410',ProbeSetID='ILMN_2701750',CellID='',db2='Illum_Retina_BXD_RankInv0410',ProbeSetID2='ILMN_2628175',CellID2='',rank='1')) | [**0.248 79**](javascript:showCorrelationPlot2(db='Illum_Retina_BXD_RankInv0410',ProbeSetID='ILMN_2701750',CellID='',db2='Illum_Retina_BXD_RankInv0410',ProbeSetID2='ILMN_2891573',CellID2='',rank='1')) | [**0.445 79**](javascript:showCorrelationPlot2(db='Illum_Retina_BXD_RankInv0410',ProbeSetID='ILMN_2701750',CellID='',db2='Illum_Retina_BXD_RankInv0410',ProbeSetID2='ILMN_1220846',CellID2='',rank='1')) | [**0.364 79**](javascript:showCorrelationPlot2(db='Illum_Retina_BXD_RankInv0410',ProbeSetID='ILMN_2701750',CellID='',db2='Illum_Retina_BXD_RankInv0410',ProbeSetID2='ILMN_1241962',CellID2='',rank='1')) | [**0.388 79**](javascript:showCorrelationPlot2(db='Illum_Retina_BXD_RankInv0410',ProbeSetID='ILMN_2701750',CellID='',db2='Illum_Retina_BXD_RankInv0410',ProbeSetID2='ILMN_3163027',CellID2='',rank='1')) | [**0.749 79**](javascript:showCorrelationPlot2(db='Illum_Retina_BXD_RankInv0410',ProbeSetID='ILMN_2701750',CellID='',db2='Illum_Retina_BXD_RankInv0410',ProbeSetID2='ILMN_2775098',CellID2='',rank='1')) | [**0.250 79**](javascript:showCorrelationPlot2(db='Illum_Retina_BXD_RankInv0410',ProbeSetID='ILMN_2701750',CellID='',db2='Illum_Retina_BXD_RankInv0410',ProbeSetID2='ILMN_2752552',CellID2='',rank='1')) | [**0.569 79**](javascript:showCorrelationPlot2(db='Illum_Retina_BXD_RankInv0410',ProbeSetID='ILMN_2701750',CellID='',db2='Illum_Retina_BXD_RankInv0410',ProbeSetID2='ILMN_2730293',CellID2='',rank='1')) | [**0.392 79**](javascript:showCorrelationPlot2(db='Illum_Retina_BXD_RankInv0410',ProbeSetID='ILMN_2701750',CellID='',db2='Illum_Retina_BXD_RankInv0410',ProbeSetID2='ILMN_2680872',CellID2='',rank='1')) | [**0.294 79**](javascript:showCorrelationPlot2(db='Illum_Retina_BXD_RankInv0410',ProbeSetID='ILMN_2701750',CellID='',db2='Illum_Retina_BXD_RankInv0410',ProbeSetID2='ILMN_2768533',CellID2='',rank='1')) | [**-0.243 79**](javascript:showCorrelationPlot2(db='Illum_Retina_BXD_RankInv0410',ProbeSetID='ILMN_2701750',CellID='',db2='Illum_Retina_BXD_RankInv0410',ProbeSetID2='ILMN_3121255',CellID2='',rank='1')) | [**-0.220 79**](javascript:showCorrelationPlot2(db='Illum_Retina_BXD_RankInv0410',ProbeSetID='ILMN_2701750',CellID='',db2='Illum_Retina_BXD_RankInv0410',ProbeSetID2='ILMN_2484527',CellID2='',rank='1')) | [**0.027 79**](javascript:showCorrelationPlot2(db='Illum_Retina_BXD_RankInv0410',ProbeSetID='ILMN_2701750',CellID='',db2='Illum_Retina_BXD_RankInv0410',ProbeSetID2='ILMN_3045723',CellID2='',rank='1')) | [**0.485 79**](javascript:showCorrelationPlot2(db='Illum_Retina_BXD_RankInv0410',ProbeSetID='ILMN_2701750',CellID='',db2='Illum_Retina_BXD_RankInv0410',ProbeSetID2='ILMN_2486573',CellID2='',rank='1')) |
|  | [**Trait 34: ILMN_1239448**](javascript:showDatabase2('Illum_Retina_BXD_RankInv0410','ILMN_1239448','');)  Symbol: Manbal  Manbal | [**0.640 79**](javascript:showCorrelationPlot2(db='Illum_Retina_BXD_RankInv0410',ProbeSetID='ILMN_1239448',CellID='',db2='Illum_Retina_BXD_RankInv0410',ProbeSetID2='ILMN_2450384',CellID2='',rank='0')) | [**0.672 79**](javascript:showCorrelationPlot2(db='Illum_Retina_BXD_RankInv0410',ProbeSetID='ILMN_1239448',CellID='',db2='Illum_Retina_BXD_RankInv0410',ProbeSetID2='ILMN_3068754',CellID2='',rank='0')) | [**0.441 79**](javascript:showCorrelationPlot2(db='Illum_Retina_BXD_RankInv0410',ProbeSetID='ILMN_1239448',CellID='',db2='Illum_Retina_BXD_RankInv0410',ProbeSetID2='ILMN_2677092',CellID2='',rank='0')) | [**0.647 79**](javascript:showCorrelationPlot2(db='Illum_Retina_BXD_RankInv0410',ProbeSetID='ILMN_1239448',CellID='',db2='Illum_Retina_BXD_RankInv0410',ProbeSetID2='ILMN_2624938',CellID2='',rank='0')) | [**0.344 79**](javascript:showCorrelationPlot2(db='Illum_Retina_BXD_RankInv0410',ProbeSetID='ILMN_1239448',CellID='',db2='Illum_Retina_BXD_RankInv0410',ProbeSetID2='ILMN_2847269',CellID2='',rank='0')) | [**0.600 79**](javascript:showCorrelationPlot2(db='Illum_Retina_BXD_RankInv0410',ProbeSetID='ILMN_1239448',CellID='',db2='Illum_Retina_BXD_RankInv0410',ProbeSetID2='ILMN_2839682',CellID2='',rank='0')) | [**0.480 79**](javascript:showCorrelationPlot2(db='Illum_Retina_BXD_RankInv0410',ProbeSetID='ILMN_1239448',CellID='',db2='Illum_Retina_BXD_RankInv0410',ProbeSetID2='ILMN_3147135',CellID2='',rank='0')) | [**0.578 79**](javascript:showCorrelationPlot2(db='Illum_Retina_BXD_RankInv0410',ProbeSetID='ILMN_1239448',CellID='',db2='Illum_Retina_BXD_RankInv0410',ProbeSetID2='ILMN_1255823',CellID2='',rank='0')) | [**0.671 79**](javascript:showCorrelationPlot2(db='Illum_Retina_BXD_RankInv0410',ProbeSetID='ILMN_1239448',CellID='',db2='Illum_Retina_BXD_RankInv0410',ProbeSetID2='ILMN_2626143',CellID2='',rank='0')) | [**0.655 79**](javascript:showCorrelationPlot2(db='Illum_Retina_BXD_RankInv0410',ProbeSetID='ILMN_1239448',CellID='',db2='Illum_Retina_BXD_RankInv0410',ProbeSetID2='ILMN_1234930',CellID2='',rank='0')) | [**0.644 79**](javascript:showCorrelationPlot2(db='Illum_Retina_BXD_RankInv0410',ProbeSetID='ILMN_1239448',CellID='',db2='Illum_Retina_BXD_RankInv0410',ProbeSetID2='ILMN_1249408',CellID2='',rank='0')) | [**0.398 79**](javascript:showCorrelationPlot2(db='Illum_Retina_BXD_RankInv0410',ProbeSetID='ILMN_1239448',CellID='',db2='Illum_Retina_BXD_RankInv0410',ProbeSetID2='ILMN_2971559',CellID2='',rank='0')) | [**0.317 79**](javascript:showCorrelationPlot2(db='Illum_Retina_BXD_RankInv0410',ProbeSetID='ILMN_1239448',CellID='',db2='Illum_Retina_BXD_RankInv0410',ProbeSetID2='ILMN_1258455',CellID2='',rank='0')) | [**0.515 79**](javascript:showCorrelationPlot2(db='Illum_Retina_BXD_RankInv0410',ProbeSetID='ILMN_1239448',CellID='',db2='Illum_Retina_BXD_RankInv0410',ProbeSetID2='ILMN_2691613',CellID2='',rank='0')) | [**0.527 79**](javascript:showCorrelationPlot2(db='Illum_Retina_BXD_RankInv0410',ProbeSetID='ILMN_1239448',CellID='',db2='Illum_Retina_BXD_RankInv0410',ProbeSetID2='ILMN_1253600',CellID2='',rank='0')) | [**0.523 79**](javascript:showCorrelationPlot2(db='Illum_Retina_BXD_RankInv0410',ProbeSetID='ILMN_1239448',CellID='',db2='Illum_Retina_BXD_RankInv0410',ProbeSetID2='ILMN_2991545',CellID2='',rank='0')) | [**0.550 79**](javascript:showCorrelationPlot2(db='Illum_Retina_BXD_RankInv0410',ProbeSetID='ILMN_1239448',CellID='',db2='Illum_Retina_BXD_RankInv0410',ProbeSetID2='ILMN_2623591',CellID2='',rank='0')) | [**0.546 79**](javascript:showCorrelationPlot2(db='Illum_Retina_BXD_RankInv0410',ProbeSetID='ILMN_1239448',CellID='',db2='Illum_Retina_BXD_RankInv0410',ProbeSetID2='ILMN_2603568',CellID2='',rank='0')) | [**0.398 79**](javascript:showCorrelationPlot2(db='Illum_Retina_BXD_RankInv0410',ProbeSetID='ILMN_1239448',CellID='',db2='Illum_Retina_BXD_RankInv0410',ProbeSetID2='ILMN_2992541',CellID2='',rank='0')) | [**0.439 79**](javascript:showCorrelationPlot2(db='Illum_Retina_BXD_RankInv0410',ProbeSetID='ILMN_1239448',CellID='',db2='Illum_Retina_BXD_RankInv0410',ProbeSetID2='ILMN_2792868',CellID2='',rank='0')) | [**0.556 79**](javascript:showCorrelationPlot2(db='Illum_Retina_BXD_RankInv0410',ProbeSetID='ILMN_1239448',CellID='',db2='Illum_Retina_BXD_RankInv0410',ProbeSetID2='ILMN_3129497',CellID2='',rank='0')) | [**0.390 79**](javascript:showCorrelationPlot2(db='Illum_Retina_BXD_RankInv0410',ProbeSetID='ILMN_1239448',CellID='',db2='Illum_Retina_BXD_RankInv0410',ProbeSetID2='ILMN_1224034',CellID2='',rank='0')) | [**0.470 79**](javascript:showCorrelationPlot2(db='Illum_Retina_BXD_RankInv0410',ProbeSetID='ILMN_1239448',CellID='',db2='Illum_Retina_BXD_RankInv0410',ProbeSetID2='ILMN_2639819',CellID2='',rank='0')) | [**0.398 79**](javascript:showCorrelationPlot2(db='Illum_Retina_BXD_RankInv0410',ProbeSetID='ILMN_1239448',CellID='',db2='Illum_Retina_BXD_RankInv0410',ProbeSetID2='ILMN_2994779',CellID2='',rank='0')) | [**0.535 79**](javascript:showCorrelationPlot2(db='Illum_Retina_BXD_RankInv0410',ProbeSetID='ILMN_1239448',CellID='',db2='Illum_Retina_BXD_RankInv0410',ProbeSetID2='ILMN_2625047',CellID2='',rank='0')) | [**0.337 79**](javascript:showCorrelationPlot2(db='Illum_Retina_BXD_RankInv0410',ProbeSetID='ILMN_1239448',CellID='',db2='Illum_Retina_BXD_RankInv0410',ProbeSetID2='ILMN_2808186',CellID2='',rank='0')) | [**0.559 79**](javascript:showCorrelationPlot2(db='Illum_Retina_BXD_RankInv0410',ProbeSetID='ILMN_1239448',CellID='',db2='Illum_Retina_BXD_RankInv0410',ProbeSetID2='ILMN_2689056',CellID2='',rank='0')) | [**0.475 79**](javascript:showCorrelationPlot2(db='Illum_Retina_BXD_RankInv0410',ProbeSetID='ILMN_1239448',CellID='',db2='Illum_Retina_BXD_RankInv0410',ProbeSetID2='ILMN_2648386',CellID2='',rank='0')) | [**0.537 79**](javascript:showCorrelationPlot2(db='Illum_Retina_BXD_RankInv0410',ProbeSetID='ILMN_1239448',CellID='',db2='Illum_Retina_BXD_RankInv0410',ProbeSetID2='ILMN_2869225',CellID2='',rank='0')) | [**0.545 79**](javascript:showCorrelationPlot2(db='Illum_Retina_BXD_RankInv0410',ProbeSetID='ILMN_1239448',CellID='',db2='Illum_Retina_BXD_RankInv0410',ProbeSetID2='ILMN_2728431',CellID2='',rank='0')) | [**0.574 79**](javascript:showCorrelationPlot2(db='Illum_Retina_BXD_RankInv0410',ProbeSetID='ILMN_1239448',CellID='',db2='Illum_Retina_BXD_RankInv0410',ProbeSetID2='ILMN_3094608',CellID2='',rank='0')) | [**0.519 79**](javascript:showCorrelationPlot2(db='Illum_Retina_BXD_RankInv0410',ProbeSetID='ILMN_1239448',CellID='',db2='Illum_Retina_BXD_RankInv0410',ProbeSetID2='ILMN_2896528',CellID2='',rank='0')) | [**0.288 79**](javascript:showCorrelationPlot2(db='Illum_Retina_BXD_RankInv0410',ProbeSetID='ILMN_1239448',CellID='',db2='Illum_Retina_BXD_RankInv0410',ProbeSetID2='ILMN_2701750',CellID2='',rank='0')) | [***n* 79**](javascript:showDatabase2('Illum_Retina_BXD_RankInv0410','ILMN_1239448','')) | [**-0.418 79**](javascript:showCorrelationPlot2(db='Illum_Retina_BXD_RankInv0410',ProbeSetID='ILMN_1239448',CellID='',db2='Illum_Retina_BXD_RankInv0410',ProbeSetID2='ILMN_2583882',CellID2='',rank='1')) | [**0.276 79**](javascript:showCorrelationPlot2(db='Illum_Retina_BXD_RankInv0410',ProbeSetID='ILMN_1239448',CellID='',db2='Illum_Retina_BXD_RankInv0410',ProbeSetID2='ILMN_2915166',CellID2='',rank='1')) | [**0.287 79**](javascript:showCorrelationPlot2(db='Illum_Retina_BXD_RankInv0410',ProbeSetID='ILMN_1239448',CellID='',db2='Illum_Retina_BXD_RankInv0410',ProbeSetID2='ILMN_2702997',CellID2='',rank='1')) | [**0.494 79**](javascript:showCorrelationPlot2(db='Illum_Retina_BXD_RankInv0410',ProbeSetID='ILMN_1239448',CellID='',db2='Illum_Retina_BXD_RankInv0410',ProbeSetID2='ILMN_2435206',CellID2='',rank='1')) | [**0.430 79**](javascript:showCorrelationPlot2(db='Illum_Retina_BXD_RankInv0410',ProbeSetID='ILMN_1239448',CellID='',db2='Illum_Retina_BXD_RankInv0410',ProbeSetID2='ILMN_2789888',CellID2='',rank='1')) | [**0.475 79**](javascript:showCorrelationPlot2(db='Illum_Retina_BXD_RankInv0410',ProbeSetID='ILMN_1239448',CellID='',db2='Illum_Retina_BXD_RankInv0410',ProbeSetID2='ILMN_1218901',CellID2='',rank='1')) | [**0.442 79**](javascript:showCorrelationPlot2(db='Illum_Retina_BXD_RankInv0410',ProbeSetID='ILMN_1239448',CellID='',db2='Illum_Retina_BXD_RankInv0410',ProbeSetID2='ILMN_1254734',CellID2='',rank='1')) | [**0.467 79**](javascript:showCorrelationPlot2(db='Illum_Retina_BXD_RankInv0410',ProbeSetID='ILMN_1239448',CellID='',db2='Illum_Retina_BXD_RankInv0410',ProbeSetID2='ILMN_3008924',CellID2='',rank='1')) | [**0.607 79**](javascript:showCorrelationPlot2(db='Illum_Retina_BXD_RankInv0410',ProbeSetID='ILMN_1239448',CellID='',db2='Illum_Retina_BXD_RankInv0410',ProbeSetID2='ILMN_2628175',CellID2='',rank='1')) | [**0.586 79**](javascript:showCorrelationPlot2(db='Illum_Retina_BXD_RankInv0410',ProbeSetID='ILMN_1239448',CellID='',db2='Illum_Retina_BXD_RankInv0410',ProbeSetID2='ILMN_2891573',CellID2='',rank='1')) | [**0.650 79**](javascript:showCorrelationPlot2(db='Illum_Retina_BXD_RankInv0410',ProbeSetID='ILMN_1239448',CellID='',db2='Illum_Retina_BXD_RankInv0410',ProbeSetID2='ILMN_1220846',CellID2='',rank='1')) | [**0.505 79**](javascript:showCorrelationPlot2(db='Illum_Retina_BXD_RankInv0410',ProbeSetID='ILMN_1239448',CellID='',db2='Illum_Retina_BXD_RankInv0410',ProbeSetID2='ILMN_1241962',CellID2='',rank='1')) | [**0.195 79**](javascript:showCorrelationPlot2(db='Illum_Retina_BXD_RankInv0410',ProbeSetID='ILMN_1239448',CellID='',db2='Illum_Retina_BXD_RankInv0410',ProbeSetID2='ILMN_3163027',CellID2='',rank='1')) | [**0.323 79**](javascript:showCorrelationPlot2(db='Illum_Retina_BXD_RankInv0410',ProbeSetID='ILMN_1239448',CellID='',db2='Illum_Retina_BXD_RankInv0410',ProbeSetID2='ILMN_2775098',CellID2='',rank='1')) | [**0.721 79**](javascript:showCorrelationPlot2(db='Illum_Retina_BXD_RankInv0410',ProbeSetID='ILMN_1239448',CellID='',db2='Illum_Retina_BXD_RankInv0410',ProbeSetID2='ILMN_2752552',CellID2='',rank='1')) | [**0.358 79**](javascript:showCorrelationPlot2(db='Illum_Retina_BXD_RankInv0410',ProbeSetID='ILMN_1239448',CellID='',db2='Illum_Retina_BXD_RankInv0410',ProbeSetID2='ILMN_2730293',CellID2='',rank='1')) | [**0.457 79**](javascript:showCorrelationPlot2(db='Illum_Retina_BXD_RankInv0410',ProbeSetID='ILMN_1239448',CellID='',db2='Illum_Retina_BXD_RankInv0410',ProbeSetID2='ILMN_2680872',CellID2='',rank='1')) | [**0.292 79**](javascript:showCorrelationPlot2(db='Illum_Retina_BXD_RankInv0410',ProbeSetID='ILMN_1239448',CellID='',db2='Illum_Retina_BXD_RankInv0410',ProbeSetID2='ILMN_2768533',CellID2='',rank='1')) | [**-0.236 79**](javascript:showCorrelationPlot2(db='Illum_Retina_BXD_RankInv0410',ProbeSetID='ILMN_1239448',CellID='',db2='Illum_Retina_BXD_RankInv0410',ProbeSetID2='ILMN_3121255',CellID2='',rank='1')) | [**-0.363 79**](javascript:showCorrelationPlot2(db='Illum_Retina_BXD_RankInv0410',ProbeSetID='ILMN_1239448',CellID='',db2='Illum_Retina_BXD_RankInv0410',ProbeSetID2='ILMN_2484527',CellID2='',rank='1')) | [**0.043 79**](javascript:showCorrelationPlot2(db='Illum_Retina_BXD_RankInv0410',ProbeSetID='ILMN_1239448',CellID='',db2='Illum_Retina_BXD_RankInv0410',ProbeSetID2='ILMN_3045723',CellID2='',rank='1')) | [**0.084 79**](javascript:showCorrelationPlot2(db='Illum_Retina_BXD_RankInv0410',ProbeSetID='ILMN_1239448',CellID='',db2='Illum_Retina_BXD_RankInv0410',ProbeSetID2='ILMN_2486573',CellID2='',rank='1')) |
[truncated: 251,169 more chars]
