# Supplementary material for: Data sets of eQTL loci, correlation analysis, and overlapped genes among gene sets that their expression levels are closely related to genes of Vegf family
Source: Data Brief. 2018 Sep 8;20:1854–60. doi: 10.1016/j.dib.2018.09.004 (PMC6169370; doi:10.1016/j.dib.2018.09.004)
Supplement: Supplementary file 3 — Supplementary material [file mmc3.docx]

Table 1. Top 50 genes that their expression levels are most correlated to that of VegfA2.

| **Gene** | ***Pgf*** | **3121255 *VegfA1*** | **3045723 *VegfA3*** | ***VegfB*** | ***VegfC*** | **2484527 *VegfA2*** | **N** |  | **Gene** | ***Pgf*** | **3121255 *VegfA*** | **3045723 *VegfA*** | ***VegfB*** | ***VegfC*** | **2484527 *VegfA2*** | **N** |
| --- | --- | --- | --- | --- | --- | --- | --- | --- | --- | --- | --- | --- | --- | --- | --- | --- |
| *Ryr2* | -0.135 | 0.195 | -0.179 | -0.463 | 0.161 | 0.817 | 79 |  | *Ryr2* | 0.135 | 0.195 | 0.179 | 0.463 | 0.161 | 0.817 | 79 |
| *Ccdc88b* | -0.153 | 0.173 | -0.148 | -0.279 | 0.215 | 0.793 | 79 |  | *Ccdc88b* | 0.153 | 0.173 | 0.148 | 0.279 | 0.215 | 0.793 | 79 |
| *Phf10* | -0.237 | 0.217 | -0.182 | -0.453 | 0.112 | 0.791 | 79 |  | *Phf10* | 0.237 | 0.217 | 0.182 | 0.453 | 0.112 | 0.791 | 79 |
| *Fam131a* | -0.155 | 0.14 | -0.211 | -0.335 | 0.178 | 0.753 | 79 |  | *Fam131a* | 0.155 | 0.14 | 0.211 | 0.335 | 0.178 | 0.753 | 79 |
| *Clk1* | -0.187 | 0.216 | -0.08 | -0.279 | 0.246 | 0.806 | 79 |  | *Clk1* | 0.187 | 0.216 | 0.08 | 0.279 | 0.246 | 0.806 | 79 |
| *Ptprs* | -0.125 | 0.125 | -0.226 | -0.383 | 0.109 | 0.762 | 79 |  | *Ptprs* | 0.125 | 0.125 | 0.226 | 0.383 | 0.109 | 0.762 | 79 |
| *Otud5* | -0.176 | 0.198 | -0.179 | -0.416 | 0.056 | 0.775 | 79 |  | *Otud5* | 0.176 | 0.198 | 0.179 | 0.416 | 0.056 | 0.775 | 79 |
| *Cbln2* | -0.163 | 0.256 | -0.041 | -0.193 | 0.253 | 0.781 | 79 |  | *Cbln2* | 0.163 | 0.256 | 0.041 | 0.193 | 0.253 | 0.781 | 79 |
| *Slc35d3* | -0.284 | 0.245 | -0.099 | -0.312 | 0.134 | 0.773 | 79 |  | *Slc35d3* | 0.284 | 0.245 | 0.099 | 0.312 | 0.134 | 0.773 | 79 |
| *A730046J19Rik* | -0.184 | 0.224 | -0.059 | -0.273 | 0.29 | 0.786 | 79 |  | *A730046J19Rik* | 0.184 | 0.224 | 0.059 | 0.273 | 0.29 | 0.786 | 79 |
| *Phf10* | -0.156 | 0.327 | -0.114 | -0.451 | -0.035 | 0.754 | 79 |  | *Phf10* | 0.156 | 0.327 | 0.114 | 0.451 | 0.035 | 0.754 | 79 |
| *BC003498* | -0.254 | 0.139 | -0.142 | -0.38 | 0.26 | 0.768 | 79 |  | *BC003498* | 0.254 | 0.139 | 0.142 | 0.38 | 0.26 | 0.768 | 79 |
| *Mettl21a* | -0.324 | 0.319 | 0.035 | -0.258 | 0.143 | 0.791 | 79 |  | *Mettl21a* | 0.324 | 0.319 | 0.035 | 0.258 | 0.143 | 0.791 | 79 |
| *Magi1* | -0.123 | 0.155 | -0.223 | -0.432 | 0.2 | 0.765 | 79 |  | *Magi1* | 0.123 | 0.155 | 0.223 | 0.432 | 0.2 | 0.765 | 79 |
| *Cacna1g* | -0.212 | 0.08 | -0.207 | -0.436 | 0.177 | 0.73 | 79 |  | *Cacna1g* | 0.212 | 0.08 | 0.207 | 0.436 | 0.177 | 0.73 | 79 |
| *Silg111* | -0.208 | 0.15 | -0.128 | -0.23 | 0.195 | 0.725 | 79 |  | *Silg111* | 0.208 | 0.15 | 0.128 | 0.23 | 0.195 | 0.725 | 79 |
| *Cacna1g* | -0.139 | 0.451 | -0.122 | -0.403 | -0.091 | 0.77 | 79 |  | *Cacna1g* | 0.139 | 0.451 | 0.122 | 0.403 | 0.091 | 0.77 | 79 |
| *Pole4* | -0.204 | 0.197 | -0.084 | -0.229 | 0.318 | 0.76 | 79 |  | *Pole4* | 0.204 | 0.197 | 0.084 | 0.229 | 0.318 | 0.76 | 79 |
| *Adarb2* | -0.081 | 0.306 | -0.132 | -0.359 | 0.073 | 0.764 | 79 |  | *Adarb2* | 0.081 | 0.306 | 0.132 | 0.359 | 0.073 | 0.764 | 79 |
| *Kiaa0947* | -0.172 | 0.136 | -0.215 | -0.464 | 0.133 | 0.746 | 79 |  | *Kiaa0947* | 0.172 | 0.136 | 0.215 | 0.464 | 0.133 | 0.746 | 79 |
| *Mrps25* | -0.19 | 0.216 | -0.085 | -0.279 | 0.18 | 0.761 | 79 |  | *Mrps25* | 0.19 | 0.216 | 0.085 | 0.279 | 0.18 | 0.761 | 79 |
| *Fbxo25* | -0.261 | 0.092 | -0.077 | -0.325 | 0.234 | 0.727 | 79 |  | *Fbxo25* | 0.261 | 0.092 | 0.077 | 0.325 | 0.234 | 0.727 | 79 |
| *Phtf1* | -0.174 | 0.272 | -0.066 | -0.328 | 0.205 | 0.778 | 79 |  | *Phtf1* | 0.174 | 0.272 | 0.066 | 0.328 | 0.205 | 0.778 | 79 |
| *Tbrg1* | -0.234 | 0.168 | -0.113 | -0.356 | 0.135 | 0.741 | 79 |  | *Tbrg1* | 0.234 | 0.168 | 0.113 | 0.356 | 0.135 | 0.741 | 79 |
| *St8sia3* | -0.221 | 0.301 | -0.052 | -0.3 | 0.22 | 0.73 | 79 |  | *St8sia3* | 0.221 | 0.301 | 0.052 | 0.3 | 0.22 | 0.73 | 79 |
| *Myo9a* | -0.094 | 0.076 | -0.185 | -0.37 | 0.296 | 0.749 | 79 |  | *Myo9a* | 0.094 | 0.076 | 0.185 | 0.37 | 0.296 | 0.749 | 79 |
| *Gria3* | -0.137 | 0.071 | -0.262 | -0.384 | 0.333 | 0.751 | 79 |  | *Gria3* | 0.137 | 0.071 | 0.262 | 0.384 | 0.333 | 0.751 | 79 |
| *Flnb* | -0.069 | 0.12 | -0.287 | -0.461 | 0.144 | 0.743 | 79 |  | *Flnb* | 0.069 | 0.12 | 0.287 | 0.461 | 0.144 | 0.743 | 79 |
| *Ccdc39* | -0.12 | 0.488 | -0.085 | -0.274 | 0.03 | 0.727 | 79 |  | *Ccdc39* | 0.12 | 0.488 | 0.085 | 0.274 | 0.03 | 0.727 | 79 |
| *Zfp446* | -0.26 | 0.116 | -0.219 | -0.444 | 0.151 | 0.76 | 79 |  | *Zfp446* | 0.26 | 0.116 | 0.219 | 0.444 | 0.151 | 0.76 | 79 |
| *Polr3a* | -0.241 | 0.127 | -0.202 | -0.359 | 0.185 | 0.753 | 79 |  | *Polr3a* | 0.241 | 0.127 | 0.202 | 0.359 | 0.185 | 0.753 | 79 |
| *Trub1* | -0.238 | 0.447 | -0.121 | -0.33 | -0.125 | 0.732 | 79 |  | *Trub1* | 0.238 | 0.447 | 0.121 | 0.33 | 0.125 | 0.732 | 79 |
| *Kiaa1191* | -0.303 | 0.189 | -0.041 | -0.421 | 0.096 | 0.709 | 79 |  | *Kiaa1191* | 0.303 | 0.189 | 0.041 | 0.421 | 0.096 | 0.709 | 79 |
| *B230216G23Rik* | -0.197 | 0.189 | -0.19 | -0.335 | 0.222 | 0.719 | 79 |  | *B230216G23Rik* | 0.197 | 0.189 | 0.19 | 0.335 | 0.222 | 0.719 | 79 |
| *Tuba1a* | -0.204 | 0.005 | -0.165 | -0.352 | 0.298 | 0.681 | 79 |  | *Tuba1a* | 0.204 | 0.005 | 0.165 | 0.352 | 0.298 | 0.681 | 79 |
| *Amigo1* | -0.088 | 0.028 | -0.244 | -0.325 | 0.132 | 0.696 | 79 |  | *Amigo1* | 0.088 | 0.028 | 0.244 | 0.325 | 0.132 | 0.696 | 79 |
| *Trim33* | -0.25 | 0.077 | -0.209 | -0.429 | 0.229 | 0.734 | 79 |  | *Trim33* | 0.25 | 0.077 | 0.209 | 0.429 | 0.229 | 0.734 | 79 |
| *Cacnb4* | -0.178 | 0.073 | -0.176 | -0.302 | 0.362 | 0.739 | 79 |  | *Cacnb4* | 0.178 | 0.073 | 0.176 | 0.302 | 0.362 | 0.739 | 79 |
| *Dzip1* | -0.188 | 0.207 | -0.228 | -0.42 | 0.247 | 0.75 | 79 |  | *Dzip1* | 0.188 | 0.207 | 0.228 | 0.42 | 0.247 | 0.75 | 79 |
| *Trpc4* | -0.25 | 0.079 | -0.207 | -0.444 | 0.211 | 0.697 | 79 |  | *Trpc4* | 0.25 | 0.079 | 0.207 | 0.444 | 0.211 | 0.697 | 79 |
| *Phtf2* | -0.243 | 0.101 | -0.152 | -0.419 | 0.182 | 0.714 | 79 |  | *Phtf2* | 0.243 | 0.101 | 0.152 | 0.419 | 0.182 | 0.714 | 79 |
| *Slc35e2* | -0.201 | 0.058 | -0.183 | -0.336 | 0.277 | 0.714 | 79 |  | *Slc35e2* | 0.201 | 0.058 | 0.183 | 0.336 | 0.277 | 0.714 | 79 |
| *B3galnt2* | -0.229 | 0.078 | -0.252 | -0.452 | 0.128 | 0.732 | 79 |  | *B3galnt2* | 0.229 | 0.078 | 0.252 | 0.452 | 0.128 | 0.732 | 79 |
| *Nktr* | -0.231 | 0.147 | -0.173 | -0.409 | 0.02 | 0.739 | 79 |  | *Nktr* | 0.231 | 0.147 | 0.173 | 0.409 | 0.02 | 0.739 | 79 |
| *Tcf4* | -0.222 | 0.109 | -0.254 | -0.509 | -0.024 | 0.705 | 79 |  | *Tcf4* | 0.222 | 0.109 | 0.254 | 0.509 | 0.024 | 0.705 | 79 |
| *Kpnb1* | -0.199 | 0.004 | -0.175 | -0.367 | 0.256 | 0.71 | 79 |  | *Kpnb1* | 0.199 | 0.004 | 0.175 | 0.367 | 0.256 | 0.71 | 79 |
| *Dhx15* | -0.277 | 0.047 | -0.11 | -0.327 | 0.276 | 0.704 | 79 |  | *Dhx15* | 0.277 | 0.047 | 0.11 | 0.327 | 0.276 | 0.704 | 79 |
| *Uck1* | -0.286 | 0.137 | -0.202 | -0.405 | 0.087 | 0.695 | 79 |  | *Uck1* | 0.286 | 0.137 | 0.202 | 0.405 | 0.087 | 0.695 | 79 |
| *Taf6* | -0.263 | 0.039 | -0.273 | -0.4 | 0.079 | 0.699 | 79 |  | *Taf6* | 0.263 | 0.039 | 0.273 | 0.4 | 0.079 | 0.699 | 79 |
| *Vps29* | -0.225 | 0.091 | -0.087 | -0.282 | 0.337 | 0.729 | 79 |  | *Vps29* | 0.225 | 0.091 | 0.087 | 0.282 | 0.337 | 0.729 | 79 |
| **Rs** | **-9.945** | **8.401** | **-7.811** | **-18.172** | **8.53** | **37.228** | **3950** |  | **Rn** | **9.945** | **8.401** | **7.881** | **18.172** | **9.08** | **37.228** | **3950** |
| **Re** | **-0.1989** | **0.16802** | **-0.15622** | **-0.36344** | **0.1706** | **0.74456** | **79** |  | **Ra** | **0.1989** | **0.16802** | **0.15762** | **0.36344** | **0.1816** | **0.74456** | **79** |

Table 2. Top 50 genes that their expression levels are most correlated to that of VegfB

| Index | Record ID | Symbol | Description | Location Chr and Mb | Mean Expr | Max LRS | Max LRS Location Chr and Mb | [Sample r](http://www.genenetwork.org/correlationAnnotation.html#genetic_r) | N Cases | [Sample p(r)](http://www.genenetwork.org/correlationAnnotation.html#genetic_p_r) | [Lit Corr](http://www.genenetwork.org/correlationAnnotation.html#literatureCorr) | [Tissue r](http://www.genenetwork.org/correlationAnnotation.html#tissue_r) | [Tissue p(r)](http://www.genenetwork.org/correlationAnnotation.html#tissue_p_r) |  |
| --- | --- | --- | --- | --- | --- | --- | --- | --- | --- | --- | --- | --- | --- | --- |
| 1  | [**ILMN_2450384**](javascript:showTrait('fm_2FVNdAFa',%20'ILMN_2450384')) | [***Vegfb***](javascript:opennewwindow('http://www.ncbi.nlm.nih.gov/entrez/query.fcgi?db=gene&cmd=Retrieve&dopt=Graphics&list_uids=22340');) | vascular endothelial growth factor B; 3' UTR | Chr19: 7.057173 | 12.614 | 13.7 | Chr9: 10.573030 | [**1.000**](javascript:showCorrPlot('fm_2FVNdAFa',%20'ILMN_2450384')) | 79 | 0.00e+00 | 1.000 | [**1.000**](javascript:showTissueCorrPlot('fm_2FVNdAFa','Vegfb','Vegfb',0)) | -- |  |
| 2  | [**ILMN_3068754**](javascript:showTrait('fm_2FVNdAFa',%20'ILMN_3068754')) | [***Akr1a4***](javascript:opennewwindow('http://www.ncbi.nlm.nih.gov/entrez/query.fcgi?db=gene&cmd=Retrieve&dopt=Graphics&list_uids=58810');) | aldo-keto reductase family 1, member A4 (aldehyde reductase, homolog of human AKR1A1); exon 7 | Chr4: 116.310599 | 14.285 | 11.2 | Chr8: 75.068926 | [**0.784**](javascript:showCorrPlot('fm_2FVNdAFa',%20'ILMN_3068754')) | 79 | 0.00e+00 | 0.422 | [**0.193**](javascript:showTissueCorrPlot('fm_2FVNdAFa','Vegfb','Akr1a4',0)) | 0.345 |  |
| 3  | [**ILMN_2677092**](javascript:showTrait('fm_2FVNdAFa',%20'ILMN_2677092')) | [***Nutf2***](javascript:opennewwindow('http://www.ncbi.nlm.nih.gov/entrez/query.fcgi?db=gene&cmd=Retrieve&dopt=Graphics&list_uids=68051');) | nuclear transport factor 2; 3' UTR | Chr8: 108.404089 | 11.095 | 17.6 | Chr3: 109.232254 | [**0.749**](javascript:showCorrPlot('fm_2FVNdAFa',%20'ILMN_2677092')) | 79 | 0.00e+00 | 0.386 | [**0.583**](javascript:showTissueCorrPlot('fm_2FVNdAFa','Vegfb','Nutf2',0)) | 0.002 |  |
| 4  | [**ILMN_2624938**](javascript:showTrait('fm_2FVNdAFa',%20'ILMN_2624938')) | [***Pea15a***](javascript:opennewwindow('http://www.ncbi.nlm.nih.gov/entrez/query.fcgi?db=gene&cmd=Retrieve&dopt=Graphics&list_uids=18611');) | phosphoprotein enriched in astrocytes 15A; 3' UTR | Chr1: 174.127046 | 14.270 | 12.0 | Chr15: 87.307011 | [**0.727**](javascript:showCorrPlot('fm_2FVNdAFa',%20'ILMN_2624938')) | 79 | 8.88e-16 | 0.520 | [**0.318**](javascript:showTissueCorrPlot('fm_2FVNdAFa','Vegfb','Pea15a',0)) | 0.113 |  |
| 5  | [**ILMN_2847269**](javascript:showTrait('fm_2FVNdAFa',%20'ILMN_2847269')) | [***Tprgl***](javascript:opennewwindow('http://www.ncbi.nlm.nih.gov/entrez/query.fcgi?db=gene&cmd=Retrieve&dopt=Graphics&list_uids=67808');) | transformation related protein 63 regulated like; 3' UTR | Chr4: 153.531766 | 12.726 | 15.4 | Chr15: 87.307011 | [**0.722**](javascript:showCorrPlot('fm_2FVNdAFa',%20'ILMN_2847269')) | 79 | 2.00e-15 | 0.332 | [**0.144**](javascript:showTissueCorrPlot('fm_2FVNdAFa','Vegfb','Tprgl',0)) | 0.484 |  |
| 6  | [**ILMN_2839682**](javascript:showTrait('fm_2FVNdAFa',%20'ILMN_2839682')) | [***Btbd6***](javascript:opennewwindow('http://www.ncbi.nlm.nih.gov/entrez/query.fcgi?db=gene&cmd=Retrieve&dopt=Graphics&list_uids=399566');) | BTB (POZ) domain containing 6; 3' UTR | Chr12: 114.216891 | 10.260 | 15.1 | Chr4: 122.536808 | [**0.698**](javascript:showCorrPlot('fm_2FVNdAFa',%20'ILMN_2839682')) | 79 | 5.11e-14 | -- | [**0.603**](javascript:showTissueCorrPlot('fm_2FVNdAFa','Vegfb','Btbd6',0)) | 0.001 |  |
| 7  | [**ILMN_3147135**](javascript:showTrait('fm_2FVNdAFa',%20'ILMN_3147135')) | [***Akr1a4***](javascript:opennewwindow('http://www.ncbi.nlm.nih.gov/entrez/query.fcgi?db=gene&cmd=Retrieve&dopt=Graphics&list_uids=58810');) | aldo-keto reductase family 1, member A4 (aldehyde reductase, homolog of human AKR1A1); exon 6 | Chr4: 116.311608 | 14.891 | 20.7 | Chr15: 87.788313 | [**0.697**](javascript:showCorrPlot('fm_2FVNdAFa',%20'ILMN_3147135')) | 79 | 6.15e-14 | 0.422 | [**0.193**](javascript:showTissueCorrPlot('fm_2FVNdAFa','Vegfb','Akr1a4',0)) | 0.345 |  |
| 8  | [**ILMN_1255823**](javascript:showTrait('fm_2FVNdAFa',%20'ILMN_1255823')) | [***Higd2a***](javascript:opennewwindow('http://www.ncbi.nlm.nih.gov/entrez/query.fcgi?db=gene&cmd=Retrieve&dopt=Graphics&list_uids=67044');) | HIG1 domain family, member 2A; mid 3' UTR | Chr13: 54.692327 | 15.229 | 18.3 | Chr15: 87.307011 | [**0.695**](javascript:showCorrPlot('fm_2FVNdAFa',%20'ILMN_1255823')) | 79 | 8.26e-14 | -- | [**0.553**](javascript:showTissueCorrPlot('fm_2FVNdAFa','Vegfb','Higd2a',0)) | 0.003 |  |
| 9  | [**ILMN_2626143**](javascript:showTrait('fm_2FVNdAFa',%20'ILMN_2626143')) | [***Phpt1***](javascript:opennewwindow('http://www.ncbi.nlm.nih.gov/entrez/query.fcgi?db=gene&cmd=Retrieve&dopt=Graphics&list_uids=75454');) | phosphohistidine phosphatase 1; exon 3 | Chr2: 25.429128 | 13.211 | 12.6 | Chr2: 69.773819 | [**0.694**](javascript:showCorrPlot('fm_2FVNdAFa',%20'ILMN_2626143')) | 79 | 9.24e-14 | 0.476 | [**0.431**](javascript:showTissueCorrPlot('fm_2FVNdAFa','Vegfb','Phpt1',0)) | 0.028 |  |
| 10  | [**ILMN_1234930**](javascript:showTrait('fm_2FVNdAFa',%20'ILMN_1234930')) | [***Vps37d***](javascript:opennewwindow('http://www.ncbi.nlm.nih.gov/entrez/query.fcgi?db=gene&cmd=Retrieve&dopt=Graphics&list_uids=194309');) | vacuolar protein sorting 37D (yeast); 3' UTR | Chr5: 135.548894 | 8.950 | 9.2 | Chr10: 35.758851 | [**0.693**](javascript:showCorrPlot('fm_2FVNdAFa',%20'ILMN_1234930')) | 79 | 1.03e-13 | 0.167 | [**-0.005**](javascript:showTissueCorrPlot('fm_2FVNdAFa','Vegfb','Vps37d',0)) | 0.982 |  |
| 11  | [**ILMN_1249408**](javascript:showTrait('fm_2FVNdAFa',%20'ILMN_1249408')) | [***Cox14***](javascript:opennewwindow('http://www.ncbi.nlm.nih.gov/entrez/query.fcgi?db=gene&cmd=Retrieve&dopt=Graphics&list_uids=66379');) | cytochrome c oxidase assembly protein 14; 3' UTR | Chr15: 99.558291 | 13.256 | 16.0 | Chr15: 87.929487 | [**0.692**](javascript:showCorrPlot('fm_2FVNdAFa',%20'ILMN_1249408')) | 79 | 1.15e-13 | -- | -- | -- |  |
| 12  | [**ILMN_2971559**](javascript:showTrait('fm_2FVNdAFa',%20'ILMN_2971559')) | [***Eef1a2***](javascript:opennewwindow('http://www.ncbi.nlm.nih.gov/entrez/query.fcgi?db=gene&cmd=Retrieve&dopt=Graphics&list_uids=13628');) | eukaryotic translation elongation factor 1 alpha 2; 3' UTR | Chr2: 180.882513 | 13.835 | 12.5 | Chr1: 80.322429 | [**0.677**](javascript:showCorrPlot('fm_2FVNdAFa',%20'ILMN_2971559')) | 79 | 6.87e-13 | 0.598 | [**0.630**](javascript:showTissueCorrPlot('fm_2FVNdAFa','Vegfb','Eef1a2',0)) | 0.001 |  |
| 13  | [**ILMN_1258455**](javascript:showTrait('fm_2FVNdAFa',%20'ILMN_1258455')) | [***C030014I23Rik***](javascript:opennewwindow('http://www.ncbi.nlm.nih.gov/entrez/query.fcgi?db=gene&cmd=Retrieve&dopt=Graphics&list_uids=77381');) | RIKEN cDNA C030014I23; putative intergenic | Chr9: 44.290004 | 9.095 | 10.3 | Chr12: 105.632190 | [**0.676**](javascript:showCorrPlot('fm_2FVNdAFa',%20'ILMN_1258455')) | 79 | 7.93e-13 | -- | [**-0.350**](javascript:showTissueCorrPlot('fm_2FVNdAFa','Vegfb','C030014I23Rik',0)) | 0.080 |  |
| 14  | [**ILMN_2691613**](javascript:showTrait('fm_2FVNdAFa',%20'ILMN_2691613')) | [***Ppp1ca***](javascript:opennewwindow('http://www.ncbi.nlm.nih.gov/entrez/query.fcgi?db=gene&cmd=Retrieve&dopt=Graphics&list_uids=19045');) | protein phosphatase 1, catalytic subunit, alpha isoform; 3' UTR | Chr19: 4.195127 | 13.161 | 25.2 | Chr15: 87.788313 | [**0.675**](javascript:showCorrPlot('fm_2FVNdAFa',%20'ILMN_2691613')) | 79 | 8.60e-13 | 0.363 | [**0.262**](javascript:showTissueCorrPlot('fm_2FVNdAFa','Vegfb','Ppp1ca',0)) | 0.196 |  |
| 15  | [**ILMN_1253600**](javascript:showTrait('fm_2FVNdAFa',%20'ILMN_1253600')) | [***Trnp1***](javascript:opennewwindow('http://www.ncbi.nlm.nih.gov/entrez/query.fcgi?db=gene&cmd=Retrieve&dopt=Graphics&list_uids=69539');) | TMF1-regulated nuclear protein 1; distal 3' UTR | Chr4: 133.047239 | 12.661 | 15.9 | Chr5: 85.065017 | [**0.675**](javascript:showCorrPlot('fm_2FVNdAFa',%20'ILMN_1253600')) | 79 | 9.12e-13 | 0.346 | -- | -- |  |
| 16  | [**ILMN_2991545**](javascript:showTrait('fm_2FVNdAFa',%20'ILMN_2991545')) | [***D10Jhu81e***](javascript:opennewwindow('http://www.ncbi.nlm.nih.gov/entrez/query.fcgi?db=gene&cmd=Retrieve&dopt=Graphics&list_uids=28295');) | DNA segment, Chr 10, Johns Hopkins University 81 expressed; 3' UTR | Chr10: 77.625049 | 11.393 | 17.5 | Chr4: 122.536808 | [**0.674**](javascript:showCorrPlot('fm_2FVNdAFa',%20'ILMN_2991545')) | 79 | 1.07e-12 | 0.484 | [**0.850**](javascript:showTissueCorrPlot('fm_2FVNdAFa','Vegfb','D10Jhu81e',0)) | 0.000 |  |
| 17  | [**ILMN_2623591**](javascript:showTrait('fm_2FVNdAFa',%20'ILMN_2623591')) | [***Apbb1***](javascript:opennewwindow('http://www.ncbi.nlm.nih.gov/entrez/query.fcgi?db=gene&cmd=Retrieve&dopt=Graphics&list_uids=11785');) | amyloid beta (A4) precursor protein-binding, family B, member 1; 3' UTR | Chr7: 112.707106 | 13.198 | 16.0 | Chr5: 85.065017 | [**0.666**](javascript:showCorrPlot('fm_2FVNdAFa',%20'ILMN_2623591')) | 79 | 2.38e-12 | 0.408 | [**0.397**](javascript:showTissueCorrPlot('fm_2FVNdAFa','Vegfb','Apbb1',0)) | 0.045 |  |
| 18  | [**ILMN_2603568**](javascript:showTrait('fm_2FVNdAFa',%20'ILMN_2603568')) | [***Leprot***](javascript:opennewwindow('http://www.ncbi.nlm.nih.gov/entrez/query.fcgi?db=gene&cmd=Retrieve&dopt=Graphics&list_uids=230514');) | leptin receptor overlapping transcript; 3' UTR | Chr4: 101.331670 | 10.845 | 13.3 | Chr1: 135.891043 | [**0.665**](javascript:showCorrPlot('fm_2FVNdAFa',%20'ILMN_2603568')) | 79 | 2.88e-12 | 0.349 | [**-0.170**](javascript:showTissueCorrPlot('fm_2FVNdAFa','Vegfb','Leprot',0)) | 0.407 |  |
| 19  | [**ILMN_2992541**](javascript:showTrait('fm_2FVNdAFa',%20'ILMN_2992541')) | [***Ergic3***](javascript:opennewwindow('http://www.ncbi.nlm.nih.gov/entrez/query.fcgi?db=gene&cmd=Retrieve&dopt=Graphics&list_uids=66366');) | ERGIC and golgi 3; exon 13 | Chr2: 155.843811 | 13.398 | 15.2 | Chr5: 33.660747 | [**0.664**](javascript:showCorrPlot('fm_2FVNdAFa',%20'ILMN_2992541')) | 79 | 3.30e-12 | 0.286 | [**-0.457**](javascript:showTissueCorrPlot('fm_2FVNdAFa','Vegfb','Ergic3',0)) | 0.019 |  |
| 20  | [**ILMN_2792868**](javascript:showTrait('fm_2FVNdAFa',%20'ILMN_2792868')) | [***Blvrb***](javascript:opennewwindow('http://www.ncbi.nlm.nih.gov/entrez/query.fcgi?db=gene&cmd=Retrieve&dopt=Graphics&list_uids=233016');) | biliverdin reductase B (flavin reductase (NADPH)); exon 5 | Chr7: 28.250812 | 9.432 | 12.1 | Chr1: 32.555264 | [**0.663**](javascript:showCorrPlot('fm_2FVNdAFa',%20'ILMN_2792868')) | 79 | 3.59e-12 | 0.301 | [**-0.130**](javascript:showTissueCorrPlot('fm_2FVNdAFa','Vegfb','Blvrb',0)) | 0.527 |  |
| 21  | [**ILMN_3129497**](javascript:showTrait('fm_2FVNdAFa',%20'ILMN_3129497')) | [***Nsmf***](javascript:opennewwindow('http://www.ncbi.nlm.nih.gov/entrez/query.fcgi?db=gene&cmd=Retrieve&dopt=Graphics&list_uids=56876');) | NMDA receptor synaptonuclear signaling and neuronal migration factor; 3' UTR | Chr2: 24.918186 | 10.607 | 14.8 | Chr1: 80.322429 | [**0.663**](javascript:showCorrPlot('fm_2FVNdAFa',%20'ILMN_3129497')) | 79 | 3.66e-12 | 0.399 | -- | -- |  |
| 22  | [**ILMN_1224034**](javascript:showTrait('fm_2FVNdAFa',%20'ILMN_1224034')) | [***Pde1b***](javascript:opennewwindow('http://www.ncbi.nlm.nih.gov/entrez/query.fcgi?db=gene&cmd=Retrieve&dopt=Graphics&list_uids=18574');) | phosphodiesterase 1B, Ca2+-calmodulin dependent; 3' UTR | Chr15: 103.360348 | 12.983 | 9.1 | Chr10: 84.376303 | [**0.662**](javascript:showCorrPlot('fm_2FVNdAFa',%20'ILMN_1224034')) | 79 | 3.95e-12 | 0.457 | [**-0.163**](javascript:showTissueCorrPlot('fm_2FVNdAFa','Vegfb','Pde1b',0)) | 0.426 |  |
| 23  | [**ILMN_2639819**](javascript:showTrait('fm_2FVNdAFa',%20'ILMN_2639819')) | [***Bet1l***](javascript:opennewwindow('http://www.ncbi.nlm.nih.gov/entrez/query.fcgi?db=gene&cmd=Retrieve&dopt=Graphics&list_uids=54399');) | blocked early in transport 1 homolog (S. cerevisiae)-like; 3' UTR | Chr7: 148.039495 | 11.169 | 12.1 | Chr5: 44.449244 | [**0.661**](javascript:showCorrPlot('fm_2FVNdAFa',%20'ILMN_2639819')) | 79 | 4.25e-12 | 0.281 | [**-0.288**](javascript:showTissueCorrPlot('fm_2FVNdAFa','Vegfb','Bet1l',0)) | 0.154 |  |
| 24  | [**ILMN_2994779**](javascript:showTrait('fm_2FVNdAFa',%20'ILMN_2994779')) | [***Tecr***](javascript:opennewwindow('http://www.ncbi.nlm.nih.gov/entrez/query.fcgi?db=gene&cmd=Retrieve&dopt=Graphics&list_uids=106529');) | trans-2,3-enoyl-CoA reductase (glycoprotein, synaptic 2); exon 13 | Chr8: 86.095731 | 14.092 | 14.0 | Chr5: 33.930826 | [**0.660**](javascript:showCorrPlot('fm_2FVNdAFa',%20'ILMN_2994779')) | 79 | 4.69e-12 | 0.368 | -- | -- |  |
| 25  | [**ILMN_2625047**](javascript:showTrait('fm_2FVNdAFa',%20'ILMN_2625047')) | [***C430004E15Rik***](javascript:opennewwindow('http://www.ncbi.nlm.nih.gov/entrez/query.fcgi?db=gene&cmd=Retrieve&dopt=Graphics&list_uids=97031');) | RIKEN cDNA C430004E15 gene; 3' UTR | Chr2: 25.125227 | 9.337 | 12.2 | Chr4: 124.048406 | [**0.660**](javascript:showCorrPlot('fm_2FVNdAFa',%20'ILMN_2625047')) | 79 | 5.12e-12 | -- | [**-0.563**](javascript:showTissueCorrPlot('fm_2FVNdAFa','Vegfb','C430004E15Rik',0)) | 0.003 |  |
| 26  | [**ILMN_2808186**](javascript:showTrait('fm_2FVNdAFa',%20'ILMN_2808186')) | [***Gtf2ird2***](javascript:opennewwindow('http://www.ncbi.nlm.nih.gov/entrez/query.fcgi?db=gene&cmd=Retrieve&dopt=Graphics&list_uids=114674');) | GTF2I repeat domain containing 2; 3' UTR | Chr5: 134.693726 | 10.551 | 12.5 | Chr3: 112.036748 | [**0.653**](javascript:showCorrPlot('fm_2FVNdAFa',%20'ILMN_2808186')) | 79 | 1.02e-11 | 0.369 | [**-0.194**](javascript:showTissueCorrPlot('fm_2FVNdAFa','Vegfb','Gtf2ird2',0)) | 0.342 |  |
| 27  | [**ILMN_2689056**](javascript:showTrait('fm_2FVNdAFa',%20'ILMN_2689056')) | [***Cd2bp2***](javascript:opennewwindow('http://www.ncbi.nlm.nih.gov/entrez/query.fcgi?db=gene&cmd=Retrieve&dopt=Graphics&list_uids=70233');) | CD2 antigen (cytoplasmic tail) binding protein 2; 3' UTR | Chr7: 134.335721 | 12.457 | 11.4 | Chr13: 71.140973 | [**0.652**](javascript:showCorrPlot('fm_2FVNdAFa',%20'ILMN_2689056')) | 79 | 1.19e-11 | 0.352 | [**0.032**](javascript:showTissueCorrPlot('fm_2FVNdAFa','Vegfb','Cd2bp2',0)) | 0.876 |  |
| 28  | [**ILMN_2648386**](javascript:showTrait('fm_2FVNdAFa',%20'ILMN_2648386')) | [***5730427N09Rik***](javascript:opennewwindow('http://www.ncbi.nlm.nih.gov/entrez/query.fcgi?db=gene&cmd=Retrieve&dopt=Graphics&list_uids=59050');) | RIKEN cDNA 5730427N09 gene; 3' UTR | Chr13: 97.901076 | 12.354 | 15.3 | Chr4: 122.536808 | [**0.648**](javascript:showCorrPlot('fm_2FVNdAFa',%20'ILMN_2648386')) | 79 | 1.68e-11 | 0.395 | [**-0.333**](javascript:showTissueCorrPlot('fm_2FVNdAFa','Vegfb','5730427N09Rik',0)) | 0.097 |  |
| 29  | [**ILMN_2869225**](javascript:showTrait('fm_2FVNdAFa',%20'ILMN_2869225')) | [***Rpl19***](javascript:opennewwindow('http://www.ncbi.nlm.nih.gov/entrez/query.fcgi?db=gene&cmd=Retrieve&dopt=Graphics&list_uids=19921');) | ribosomal protein L19; exon 5 | Chr11: 97.891623 | 12.590 | 10.9 | Chr2: 62.000421 | [**0.645**](javascript:showCorrPlot('fm_2FVNdAFa',%20'ILMN_2869225')) | 79 | 2.27e-11 | 0.537 | [**0.433**](javascript:showTissueCorrPlot('fm_2FVNdAFa','Vegfb','Rpl19',0)) | 0.027 |  |
| 30  | [**ILMN_2728431**](javascript:showTrait('fm_2FVNdAFa',%20'ILMN_2728431')) | [***Cdc42ep4***](javascript:opennewwindow('http://www.ncbi.nlm.nih.gov/entrez/query.fcgi?db=gene&cmd=Retrieve&dopt=Graphics&list_uids=56699');) | CDC42 effector protein (Rho GTPase binding) 4; 3' UTR | Chr11: 113.588336 | 11.703 | 13.8 | Chr15: 87.929487 | [**0.645**](javascript:showCorrPlot('fm_2FVNdAFa',%20'ILMN_2728431')) | 79 | 2.38e-11 | 0.280 | [**-0.490**](javascript:showTissueCorrPlot('fm_2FVNdAFa','Vegfb','Cdc42ep4',0)) | 0.011 |  |
| 31  | [**ILMN_3094608**](javascript:showTrait('fm_2FVNdAFa',%20'ILMN_3094608')) | [***Rdbp***](javascript:opennewwindow('http://www.ncbi.nlm.nih.gov/entrez/query.fcgi?db=gene&cmd=Retrieve&dopt=Graphics&list_uids=27632');) | RD RNA-binding protein; putative intergenic | Chr10: 84.653178 | 9.540 | 13.6 | Chr8: 75.068926 | [**0.644**](javascript:showCorrPlot('fm_2FVNdAFa',%20'ILMN_3094608')) | 79 | 2.73e-11 | 0.372 | [**-0.761**](javascript:showTissueCorrPlot('fm_2FVNdAFa','Vegfb','Rdbp',0)) | 0.000 |  |
| 32  | [**ILMN_2896528**](javascript:showTrait('fm_2FVNdAFa',%20'ILMN_2896528')) | [***Mre11a***](javascript:opennewwindow('http://www.ncbi.nlm.nih.gov/entrez/query.fcgi?db=gene&cmd=Retrieve&dopt=Graphics&list_uids=17535');) | meiotic recombination 11 homolog A (S. cerevisiae); intron 9 | Chr9: 14.612260 | 13.263 | 16.6 | Chr4: 122.536808 | [**0.642**](javascript:showCorrPlot('fm_2FVNdAFa',%20'ILMN_2896528')) | 79 | 3.08e-11 | 0.287 | [**0.688**](javascript:showTissueCorrPlot('fm_2FVNdAFa','Vegfb','Mre11a',0)) | 0.000 |  |
| 33  | [**ILMN_2701750**](javascript:showTrait('fm_2FVNdAFa',%20'ILMN_2701750')) | [***2310061J03Rik***](javascript:opennewwindow('http://www.ncbi.nlm.nih.gov/entrez/query.fcgi?db=gene&cmd=Retrieve&dopt=Graphics&list_uids=66391');) | RIKEN cDNA 2310061J03 gene; putative intergenic | Chr16: 55.973518 | 8.838 | 10.4 | Chr1: 29.231425 | [**0.640**](javascript:showCorrPlot('fm_2FVNdAFa',%20'ILMN_2701750')) | 79 | 3.85e-11 | -- | [**0.065**](javascript:showTissueCorrPlot('fm_2FVNdAFa','Vegfb','2310061J03Rik',0)) | 0.751 |  |
| 34  | [**ILMN_1239448**](javascript:showTrait('fm_2FVNdAFa',%20'ILMN_1239448')) | [***Manbal***](javascript:opennewwindow('http://www.ncbi.nlm.nih.gov/entrez/query.fcgi?db=gene&cmd=Retrieve&dopt=Graphics&list_uids=69161');) | mannosidase, beta A, lysosomal-like; 3' UTR | Chr2: 157.222398 | 12.243 | 13.0 | Chr2: 77.938377 | [**0.640**](javascript:showCorrPlot('fm_2FVNdAFa',%20'ILMN_1239448')) | 79 | 3.94e-11 | -- | [**0.191**](javascript:showTissueCorrPlot('fm_2FVNdAFa','Vegfb','Manbal',0)) | 0.350 |  |
| 35  | [**ILMN_2583882**](javascript:showTrait('fm_2FVNdAFa',%20'ILMN_2583882')) | [***D630021C08Rik***](javascript:opennewwindow('http://www.ncbi.nlm.nih.gov/entrez/query.fcgi?CMD=search&DB=gene&term=D630021C08Rik');) | D630021C08Rik; putative intergenic | Chr7: 96.536895 | 7.223 | 24.7 | Chr15: 87.929487 | [**-0.640**](javascript:showCorrPlot('fm_2FVNdAFa',%20'ILMN_2583882')) | 79 | 3.94e-11 | -- | [**0.397**](javascript:showTissueCorrPlot('fm_2FVNdAFa','Vegfb','D630021C08Rik',0)) | 0.045 |  |
| 36  | [**ILMN_2915166**](javascript:showTrait('fm_2FVNdAFa',%20'ILMN_2915166')) | [***Znhit4***](javascript:opennewwindow('http://www.ncbi.nlm.nih.gov/entrez/query.fcgi?db=gene&cmd=Retrieve&dopt=Graphics&list_uids=70020');) | zinc finger, HIT type 4; exon 5 | Chr6: 83.071864 | 8.750 | 12.9 | Chr3: 115.644636 | [**0.639**](javascript:showCorrPlot('fm_2FVNdAFa',%20'ILMN_2915166')) | 79 | 4.19e-11 | 0.387 | [**-0.629**](javascript:showTissueCorrPlot('fm_2FVNdAFa','Vegfb','Znhit4',0)) | 0.001 |  |
| 37  | [**ILMN_2702997**](javascript:showTrait('fm_2FVNdAFa',%20'ILMN_2702997')) | [***Thap7***](javascript:opennewwindow('http://www.ncbi.nlm.nih.gov/entrez/query.fcgi?db=gene&cmd=Retrieve&dopt=Graphics&list_uids=69009');) | THAP domain containing 7; exon 5 | Chr16: 17.528314 | 10.099 | 11.1 | Chr18: 50.847059 | [**0.638**](javascript:showCorrPlot('fm_2FVNdAFa',%20'ILMN_2702997')) | 79 | 4.82e-11 | 0.300 | [**-0.343**](javascript:showTissueCorrPlot('fm_2FVNdAFa','Vegfb','Thap7',0)) | 0.086 |  |
| 38  | [**ILMN_2435206**](javascript:showTrait('fm_2FVNdAFa',%20'ILMN_2435206')) | [***Tmem14c***](javascript:opennewwindow('http://www.ncbi.nlm.nih.gov/entrez/query.fcgi?db=gene&cmd=Retrieve&dopt=Graphics&list_uids=66154');) | transmembrane protein 14C; 3' UTR | Chr13: 41.117735 | 12.047 | 18.8 | Chr8: 75.068926 | [**0.638**](javascript:showCorrPlot('fm_2FVNdAFa',%20'ILMN_2435206')) | 79 | 4.97e-11 | 0.176 | [**0.134**](javascript:showTissueCorrPlot('fm_2FVNdAFa','Vegfb','Tmem14c',0)) | 0.513 |  |
| 39  | [**ILMN_2789888**](javascript:showTrait('fm_2FVNdAFa',%20'ILMN_2789888')) | [***Rnf10***](javascript:opennewwindow('http://www.ncbi.nlm.nih.gov/entrez/query.fcgi?db=gene&cmd=Retrieve&dopt=Graphics&list_uids=50849');) | ring finger protein 10; exon 17 | Chr5: 115.691977 | 14.623 | 11.9 | Chr3: 109.232254 | [**0.638**](javascript:showCorrPlot('fm_2FVNdAFa',%20'ILMN_2789888')) | 79 | 5.03e-11 | 0.391 | [**-0.065**](javascript:showTissueCorrPlot('fm_2FVNdAFa','Vegfb','Rnf10',0)) | 0.751 |  |
| 40  | [**ILMN_1218901**](javascript:showTrait('fm_2FVNdAFa',%20'ILMN_1218901')) | [***Tbc1d13***](javascript:opennewwindow('http://www.ncbi.nlm.nih.gov/entrez/query.fcgi?db=gene&cmd=Retrieve&dopt=Graphics&list_uids=70296');) | TBC1 domain family, member 13; 3' UTR | Chr2: 30.007287 | 10.789 | 7.6 | Chr9: 10.573030 | [**0.637**](javascript:showCorrPlot('fm_2FVNdAFa',%20'ILMN_1218901')) | 79 | 5.18e-11 | 0.228 | [**-0.178**](javascript:showTissueCorrPlot('fm_2FVNdAFa','Vegfb','Tbc1d13',0)) | 0.384 |  |
| 41  | [**ILMN_1254734**](javascript:showTrait('fm_2FVNdAFa',%20'ILMN_1254734')) | [***Mrps12***](javascript:opennewwindow('http://www.ncbi.nlm.nih.gov/entrez/query.fcgi?db=gene&cmd=Retrieve&dopt=Graphics&list_uids=24030');) | mitochondrial ribosomal protein S12; 3' UTR | Chr7: 29.524829 | 12.527 | 12.0 | Chr15: 87.307011 | [**0.637**](javascript:showCorrPlot('fm_2FVNdAFa',%20'ILMN_1254734')) | 79 | 5.23e-11 | 0.440 | [**0.493**](javascript:showTissueCorrPlot('fm_2FVNdAFa','Vegfb','Mrps12',0)) | 0.011 |  |
| 42  | [**ILMN_3008924**](javascript:showTrait('fm_2FVNdAFa',%20'ILMN_3008924')) | [***Mobkl2a***](javascript:opennewwindow('http://www.ncbi.nlm.nih.gov/entrez/query.fcgi?db=gene&cmd=Retrieve&dopt=Graphics&list_uids=208228');) | MOB1, Mps One Binder kinase activator-like 2A (yeast); 3' UTR | Chr10: 80.148136 | 9.177 | 15.3 | Chr5: 85.890222 | [**0.636**](javascript:showCorrPlot('fm_2FVNdAFa',%20'ILMN_3008924')) | 79 | 6.12e-11 | -- | [**-0.383**](javascript:showTissueCorrPlot('fm_2FVNdAFa','Vegfb','Mobkl2a',0)) | 0.053 |  |
| 43  | [**ILMN_2628175**](javascript:showTrait('fm_2FVNdAFa',%20'ILMN_2628175')) | [***Tsen34***](javascript:opennewwindow('http://www.ncbi.nlm.nih.gov/entrez/query.fcgi?db=gene&cmd=Retrieve&dopt=Graphics&list_uids=66078');) | tRNA splicing endonuclease 34 homolog (SEN34, S. cerevisiae); 3' UTR | Chr7: 3.652343 | 11.984 | 14.9 | Chr15: 87.307011 | [**0.635**](javascript:showCorrPlot('fm_2FVNdAFa',%20'ILMN_2628175')) | 79 | 6.16e-11 | 0.332 | [**0.176**](javascript:showTissueCorrPlot('fm_2FVNdAFa','Vegfb','Tsen34',0)) | 0.388 |  |
| 44  | [**ILMN_2891573**](javascript:showTrait('fm_2FVNdAFa',%20'ILMN_2891573')) | [***Rai12***](javascript:opennewwindow('http://www.ncbi.nlm.nih.gov/entrez/query.fcgi?db=gene&cmd=Retrieve&dopt=Graphics&list_uids=54351');) | retinoic acid induced 12; exon 8 | Chr11: 69.781999 | 11.417 | 17.3 | Chr15: 87.788313 | [**0.635**](javascript:showCorrPlot('fm_2FVNdAFa',%20'ILMN_2891573')) | 79 | 6.73e-11 | 0.411 | [**-0.098**](javascript:showTissueCorrPlot('fm_2FVNdAFa','Vegfb','Rai12',0)) | 0.633 |  |
| 45  | [**ILMN_1220846**](javascript:showTrait('fm_2FVNdAFa',%20'ILMN_1220846')) | [***Ccdc85b***](javascript:opennewwindow('http://www.ncbi.nlm.nih.gov/entrez/query.fcgi?db=gene&cmd=Retrieve&dopt=Graphics&list_uids=240514');) | coiled-coil domain containing 85B; 3' UTR | Chr19: 5.456711 | 11.232 | 12.5 | Chr19: 10.826463 | [**0.633**](javascript:showCorrPlot('fm_2FVNdAFa',%20'ILMN_1220846')) | 79 | 7.91e-11 | 0.342 | [**0.402**](javascript:showTissueCorrPlot('fm_2FVNdAFa','Vegfb','Ccdc85b',0)) | 0.042 |  |
| 46  | [**ILMN_1241962**](javascript:showTrait('fm_2FVNdAFa',%20'ILMN_1241962')) | [***Cox5b***](javascript:opennewwindow('http://www.ncbi.nlm.nih.gov/entrez/query.fcgi?db=gene&cmd=Retrieve&dopt=Graphics&list_uids=12859');) | cytochrome c oxidase, subunit Vb; exon 3 | Chr1: 36.749313 | 15.559 | 27.3 | Chr1: 36.052583 | [**0.633**](javascript:showCorrPlot('fm_2FVNdAFa',%20'ILMN_1241962')) | 79 | 7.94e-11 | 0.502 | [**0.694**](javascript:showTissueCorrPlot('fm_2FVNdAFa','Vegfb','Cox5b',0)) | 0.000 |  |
| 47  | [**ILMN_3163027**](javascript:showTrait('fm_2FVNdAFa',%20'ILMN_3163027')) | [***Fam108a***](javascript:opennewwindow('http://www.ncbi.nlm.nih.gov/entrez/query.fcgi?db=gene&cmd=Retrieve&dopt=Graphics&list_uids=216169');) | abhydrolase domain-containing protein FAM108A; exon 5 | Chr10: 80.046756 | 13.276 | 14.6 | Chr1: 80.322429 | [**0.633**](javascript:showCorrPlot('fm_2FVNdAFa',%20'ILMN_3163027')) | 79 | 8.19e-11 | -- | [**-0.500**](javascript:showTissueCorrPlot('fm_2FVNdAFa','Vegfb','Fam108a',0)) | 0.009 |  |
| 48  | [**ILMN_2775098**](javascript:showTrait('fm_2FVNdAFa',%20'ILMN_2775098')) | [***Cyb5***](javascript:opennewwindow('http://www.ncbi.nlm.nih.gov/entrez/query.fcgi?db=gene&cmd=Retrieve&dopt=Graphics&list_uids=109672');) | cytochrome b-5; exon 6 | Chr18: 85.048950 | 12.386 | 11.3 | Chr1: 135.891043 | [**0.632**](javascript:showCorrPlot('fm_2FVNdAFa',%20'ILMN_2775098')) | 79 | 8.82e-11 | 0.374 | [**0.086**](javascript:showTissueCorrPlot('fm_2FVNdAFa','Vegfb','Cyb5',0)) | 0.675 |  |
| 49  | [**ILMN_2752552**](javascript:showTrait('fm_2FVNdAFa',%20'ILMN_2752552')) | [***Sod1***](javascript:opennewwindow('http://www.ncbi.nlm.nih.gov/entrez/query.fcgi?db=gene&cmd=Retrieve&dopt=Graphics&list_uids=20655');) | superoxide dismutase 1, soluble; exon 5 | Chr16: 90.225428 | 14.272 | 10.4 | Chr9: 10.573030 | [**0.632**](javascript:showCorrPlot('fm_2FVNdAFa',%20'ILMN_2752552')) | 79 | 8.83e-11 | 0.358 | [**0.220**](javascript:showTissueCorrPlot('fm_2FVNdAFa','Vegfb','Sod1',0)) | 0.280 |  |
| 50  | [**ILMN_2730293**](javascript:showTrait('fm_2FVNdAFa',%20'ILMN_2730293')) | [***Pde1b***](javascript:opennewwindow('http://www.ncbi.nlm.nih.gov/entrez/query.fcgi?db=gene&cmd=Retrieve&dopt=Graphics&list_uids=18574');) | phosphodiesterase 1B, Ca2+-calmodulin dependent; 3' UTR | Chr15: 103.360350 | 13.045 | 10.8 | Chr5: 42.600454 | [**0.631**](javascript:showCorrPlot('fm_2FVNdAFa',%20'ILMN_2730293')) | 79 | 9.16e-11 | 0.457 | [**-0.163**](javascript:showTissueCorrPlot('fm_2FVNdAFa','Vegfb','Pde1b',0)) | 0.426 |  |
| 51  | [**ILMN_2680872**](javascript:showTrait('fm_2FVNdAFa',%20'ILMN_2680872')) | [***Mtvr2***](javascript:opennewwindow('http://www.ncbi.nlm.nih.gov/entrez/query.fcgi?db=gene&cmd=Retrieve&dopt=Graphics&list_uids=17826');) | mammary tumor virus receptor 2; intron 28 | Chr12: 45.686075 | 9.855 | 19.6 | Chr19: 4.845967 | [**0.629**](javascript:showCorrPlot('fm_2FVNdAFa',%20'ILMN_2680872')) | 79 | 1.10e-10 | 0.399 | [**-0.297**](javascript:showTissueCorrPlot('fm_2FVNdAFa','Vegfb','Mtvr2',0)) | 0.140 |  |
| Rs |  |  |  |  |  |  |  | **31.817** |  |  |  |  |  |  |
| Re |  |  |  |  |  |  |  | **0.63634** |  |  |  |  |  |  |
| Rn |  |  |  |  |  |  |  | **33.097** |  |  |  |  |  |  |
| Ra |  |  |  |  |  |  |  | **0.66194** |  |  |  |  |  |  |

Table 3. Top 50 genes that their expression levels are most correlated to that of VegfC.

| **Record** | **Symbol** | **Description** | **Location**  **Chr and Mb** | **Mean**  **Expr** | **Max**  **LRS** | **Max LRS Location**  **Chr and Mb** | [**Sample**](http://www.genenetwork.org/correlationAnnotation.html#genetic_r)  **r** | **N**  **Cases** | [**Sample**](http://www.genenetwork.org/correlationAnnotation.html#genetic_p_r)  **p(r)** | [**Lit**](http://www.genenetwork.org/correlationAnnotation.html#literatureCorr)  **Corr** | [**Tissue**](http://www.genenetwork.org/correlationAnnotation.html#tissue_r)  **r** | [**Tissue**](http://www.genenetwork.org/correlationAnnotation.html#tissue_p_r)  **p(r)** |
| --- | --- | --- | --- | --- | --- | --- | --- | --- | --- | --- | --- | --- |
| **ID** |  |  |  |  |  |  |  |  |  |  |  |  |
| ILMN_2476804 | *Ube2a* | ubiquitin-conjugating enzyme E2A, RAD6 homolog (S. cerevisiae); 3' UTR | ChrX: 36.883682 | 10.531 | 19.4 | Chr18: 82.240604 | 0.701 | 68 | 2.37E-12 | 0.361 | 0.461 | 0.018 |
| ILMN_1218424 | *Ppp2r2d* | protein phosphatase 2, regulatory subunit B, delta isoform; 3' UTR, verified transQTL on Chr 9 at 27 Mb near Jam3 | Chr7: 138.882503 | 11.418 | 10.6 | Chr1: 170.949421 | 0.653 | 68 | 3.27E-10 | 0.447 | -0.379 | 0.056 |
| ILMN_1237803 | *Fryl* | FRY like transcription coactivator; 3' UTR | Chr5: 73.020212 | 12.396 | 11.7 | Chr1: 15.981339 | -0.649 | 68 | 4.64E-10 | -- | 0.051 | 0.805 |
| ILMN_1233188 | *0610007N19Rik* | RIKEN cDNA 0610007N19 gene; putative intergenic | Chr15: 32.240856 | 8.308 | 15.8 | Chr15: 28.302726 | 0.646 | 68 | 5.84E-10 | -- | 0.391 | 0.048 |
| ILMN_2579546 | *D130027H15Rik* | RIKEN D130027H15Rik; putative intergenic | Chr6: 31.568742 | 7.214 | 9.6 | Chr8: 128.365196 | -0.643 | 68 | 7.85E-10 | -- | 0.158 | 0.441 |
| ILMN_1243908 | *Sh3gl1* | SH3-domain GRB2-like 1; 3' UTR | Chr17: 56.016831 | 9.163 | 13.7 | Chr1: 15.981339 | -0.641 | 68 | 9.24E-10 | 0.431 | -0.33 | 0.1 |
| ILMN_2711651 | *Tmco1* | transmembrane and coiled-coil domains 1; 3' UTR | Chr1: 167.330386 | 11.204 | 23 | Chr1: 166.983006 | 0.635 | 68 | 1.51E-09 | -- | 0.386 | 0.051 |
| ILMN_2860586 | *Tacr3* | tachykinin receptor 3 (neurokinin B); disal 3' UTR | Chr3: 134.934135 | 8.099 | 11.3 | Chr16: 32.702145 | 0.623 | 68 | 4.09E-09 | 0.326 | -0.29 | 0.151 |
| ILMN_2468617 | *Med12* | mediator of RNA polymerase II transcription, subunit 12 homolog (yeast); 3' UTR | ChrX: 101.297222 | 10.321 | 12.6 | Chr5: 146.682242 | -0.613 | 68 | 8.76E-09 | 0.419 | 0.182 | 0.373 |
| ILMN_1260536 | *Tmem1* | transmembrane protein 1; 3' UTR | Chr10: 77.590044 | 12.238 | 18.8 | Chr3: 109.429336 | -0.612 | 68 | 9.82E-09 | -- | 0.138 | 0.501 |
| ILMN_2684279 | *Edem2* | ER degradation enhancer, mannosidase alpha-like 2; exon 11 | Chr2: 155.702084 | 12.522 | 12.7 | Chr1: 15.981339 | -0.611 | 68 | 1.04E-08 | 0.129 | 0.029 | 0.887 |
| ILMN_2637624 | *1810055E12Rik* | RIKEN cDNA 1810055E12 gene; 3' UTR | Chr19: 60.835871 | 11.925 | 11.5 | Chr18: 82.240604 | 0.61 | 68 | 1.13E-08 | -- | -0.245 | 0.227 |
| ILMN_2720356 | *Arhgef11* | Rho guanine nucleotide exchange factor (GEF) 11; 3' UTR | Chr3: 87.737841 | 12.298 | 11.9 | Chr18: 82.377768 | -0.608 | 68 | 1.34E-08 | 0.33 | 0.311 | 0.122 |
| ILMN_2501605 | *Prkcb1* | protein kinase C, beta 1; 3' UTR | Chr7: 122.633869 | 10.974 | 8.2 | Chr4: 156.100964 | 0.603 | 68 | 1.79E-08 | 0.437 | -0.035 | 0.864 |
| ILMN_2717447 | *Gpr103* | G protein-coupled receptor 103; exon 6 | Chr3: 36.180111 | 8.58 | 11.9 | Chr17: 7.705398 | 0.603 | 68 | 1.89E-08 | 0.376 | -0.025 | 0.904 |
| ILMN_2492491 | *C030026E19Rik* | C030026E19Rik; putative intergenic | Chr9: 53.437425 | 11.422 | 11.5 | Chr17: 43.098144 | -0.603 | 68 | 1.89E-08 | -- | 0.11 | 0.592 |
| ILMN_1248555 | *Gns* | glucosamine (N-acetyl)-6-sulfatase; 3' UTR | Chr10: 121.396995 | 12.918 | 16.5 | Chr18: 84.126263 | 0.602 | 68 | 2.05E-08 | -- | 0.464 | 0.017 |
| ILMN_3131197 | *Repin1* | replication initiator 1; putative intergenic | Chr7: 83.467989 | 11.849 | 15 | Chr16: 32.702145 | -0.6 | 68 | 2.37E-08 | 0.266 | -0.059 | 0.775 |
| ILMN_2610204 | *Mtch2* | mitochondrial carrier homolog 2; 3' UTR | Chr2: 90.866138 | 10.125 | 9.2 | Chr12: 36.075170 | 0.597 | 68 | 2.91E-08 | 0.188 | -0.214 | 0.293 |
| ILMN_2608073 | *Nap1l5* | nucleosome assembly protein 1-like 5; intron 23 | Chr6: 58.905649 | 12.707 | 10.8 | Chr1: 4.878037 | 0.594 | 68 | 3.61E-08 | 0.212 | -0.029 | 0.889 |
| ILMN_3051252 | *BC026590* | cDNA sequence BC026590; 3' UTR | Chr4: 56.809413 | 10.276 | 14.5 | Chr1: 14.576853 | -0.593 | 68 | 3.87E-08 | 0.293 | -0.386 | 0.051 |
| ILMN_2660328 | *Ubxd8* | UBX domain containing 8; 3' UTR | Chr13: 54.663757 | 10.748 | 11.2 | Chr17: 29.874146 | 0.589 | 68 | 5.04E-08 | 0.114 | -0.258 | 0.203 |
| ILMN_2793503 | *Uchl5* | ubiquitin carboxyl-terminal esterase L5; exon 9 | Chr1: 143.799918 | 10.174 | 11.1 | Chr1: 4.878037 | 0.589 | 68 | 5.06E-08 | 0.266 | -0.416 | 0.034 |
| ILMN_1236312 | *Snx14* | sorting nexin 14; exon 26 | Chr9: 88.380192 | 10.132 | 8.7 | Chr2: 181.014276 | 0.587 | 68 | 5.86E-08 | 0.279 | -0.054 | 0.793 |
| ILMN_2755483 | *Kctd1* | potassium channel tetramerisation domain containing 1; 3' UTR | Chr18: 14.969434 | 10.012 | 16.7 | Chr18: 73.475369 | 0.586 | 68 | 6.25E-08 | -- | -0.086 | 0.675 |
| ILMN_2780759 | *Pknox1* | Pbx/knotted 1 homeobox; putative intergenic | Chr10: 103.237199 | 9.106 | 11 | Chr1: 166.983006 | -0.585 | 68 | 6.41E-08 | 0.371 | -0.359 | 0.072 |
| ILMN_1256680 | *4930402I24Rik* | 4930402I24Rik; putative intergenic | Chr13: 54.875458 | 10.746 | 17.3 | Chr17: 27.451463 | 0.585 | 68 | 6.5E-08 | -- | -0.436 | 0.026 |
| ILMN_1230060 | *Rbm17* | RNA binding motif protein 17; intron 3 | Chr6: 92.564740 | 11.262 | 12.4 | Chr1: 4.878037 | 0.585 | 68 | 6.6E-08 | 0.293 | 0 | 0.998 |
| ILMN_2856144 | *Mtmr2* | myotubularin related protein 2; 3' UTR | Chr9: 13.805745 | 8.791 | 8.2 | Chr1: 166.393012 | 0.585 | 68 | 6.66E-08 | 0.311 | -0.164 | 0.424 |
| ILMN_2751247 | *Urm1* | ubiquitin related modifier 1 homolog (S. cerevisiae); 3' UTR | Chr2: 29.844925 | 9.6 | 14.6 | Chr17: 10.720847 | -0.584 | 68 | 6.94E-08 | 0.222 | 0.209 | 0.304 |
| ILMN_1250036 | *D4Ertd429e* | DNA segment, Chr 4, ERATO Doi 429, expressed; 3' UTR | Chr4: 149.715438 | 9.363 | 14.8 | Chr18: 82.240604 | -0.584 | 68 | 7.14E-08 | 0.268 | -0.082 | 0.691 |
| ILMN_1240153 | *Uchl5* | ubiquitin carboxyl-terminal esterase L5; exon 10 | Chr1: 143.799918 | 10.24 | 12.3 | Chr1: 4.878037 | 0.584 | 68 | 7.24E-08 | 0.266 | -0.416 | 0.034 |
| ILMN_1257333 | *C1orf198* | human chromosome 1 open reading frame 198; 3' UTR | Chr8: 124.635827 | 11.041 | 10.6 | Chr18: 49.946937 | -0.583 | 68 | 7.52E-08 | -- | -- | -- |
| ILMN_3139818 | *Sbf1* | SET binding factor 1; 3' UTR | Chr15: 89.288345 | 11.412 | 14.2 | Chr18: 49.946937 | -0.582 | 68 | 8.08E-08 | 0.204 | -0.296 | 0.143 |
| ILMN_2814350 | *Mkks* | McKusick-Kaufman syndrome protein; 3' UTR | Chr2: 136.874185 | 9.958 | 13.9 | Chr18: 82.240604 | 0.582 | 68 | 8.34E-08 | 0.415 | 0.196 | 0.338 |
| ILMN_1224658 | *Spast* | spastin; exon 18 | Chr17: 74.388238 | 9.113 | 11.9 | Chr1: 166.393012 | 0.579 | 68 | 9.58E-08 | 0.362 | -0.166 | 0.419 |
| ILMN_2735157 | *Eif3s10* | eukaryotic translation initiation factor 3, subunit 10 (theta); 3' UTR | Chr19: 60.761648 | 11.24 | 8.9 | Chr2: 181.014276 | 0.579 | 68 | 1.02E-07 | 0.443 | 0.288 | 0.153 |
| ILMN_2655862 | *Syngr3* | synaptogyrin 3; 3' UTR | Chr17: 24.685358 | 11.665 | 10.1 | Chr1: 171.051732 | 0.577 | 68 | 1.1E-07 | 0.25 | -0.143 | 0.485 |
| ILMN_1218597 | *Lrrc50* | leucine rich repeat containing 50; 3' UTR | Chr8: 119.598073 | 9.961 | 13.7 | Chr18: 49.946937 | -0.575 | 68 | 1.27E-07 | 0.186 | -0.022 | 0.917 |
| ILMN_3159059 | *Rheb* | RAS-homolog enriched in brain, GTP-binding protein; exon 8 | Chr5: 24.803654 | 13.725 | 11.6 | Chr18: 82.240604 | 0.575 | 68 | 1.27E-07 | 0.403 | 0.399 | 0.044 |
| ILMN_1214054 | *Zdhhc7* | zinc finger, DHHC domain containing 7; 3' UTR | Chr8: 120.081152 | 12.303 | 13.3 | Chr13: 59.704246 | -0.575 | 68 | 1.3E-07 | 0.282 | 0.341 | 0.089 |
| ILMN_1236303 | *Wdsof1* | WD repeats and SOF domain containing 1; exon 10 | Chr15: 39.143694 | 10.691 | 19.4 | Chr2: 181.014276 | 0.574 | 68 | 1.42E-07 | -- | -0.138 | 0.501 |
| ILMN_2884098 | *Gorasp2* | golgi reassembly stacking protein 2; 3' UTR | Chr2: 70.691446 | 13.266 | 13.3 | Chr18: 82.240604 | 0.573 | 68 | 1.44E-07 | 0.234 | 0.29 | 0.15 |
| ILMN_2795412 | *Tmem176a* | transmembrane protein 176A; putative intergenic | Chr6: 48.846663 | 8.378 | 16.4 | Chr15: 28.302726 | 0.571 | 68 | 1.68E-07 | 0.404 | 0.282 | 0.163 |
| ILMN_2465338 | *Pigt* | phosphatidylinositol glycan anchor biosynthesis, class T; 3' UTR | Chr2: 164.508158 | 10.679 | 18.2 | Chr4: 156.100964 | -0.565 | 68 | 2.5E-07 | 0.319 | -0.006 | 0.975 |
| ILMN_3162174 | *Immt* | inner membrane protein, mitochondrial; 3' UTR | Chr6: 149.320261 | 13.898 | 13.8 | Chr18: 82.377768 | 0.563 | 68 | 2.75E-07 | 0.307 | 0.112 | 0.587 |
| ILMN_1245979 | *LOC381891* | similar to ubiquitin-conjugating enzyme E2N (homologous to yeast UBC13), bendless protein; putative intergenic | Chr7: 61.549395 | 10.399 | 11.5 | Chr18: 82.240604 | 0.56 | 68 | 3.29E-07 | -- | -0.43 | 0.028 |
| ILMN_2451036 | *Ube2h* | ubiquitin-conjugating enzyme E2H; 3' UTR | Chr6: 30.213127 | 10.771 | 20.1 | Chr18: 49.946937 | -0.559 | 68 | 3.58E-07 | 0.322 | 0.278 | 0.17 |
| ILMN_2511953 | *Tnfaip8* | tumor necrosis factor, alpha-induced protein 8; 3' UTR | Chr18: 50.091383 | 9.203 | 13 | Chr17: 6.039016 | 0.558 | 68 | 3.7E-07 | 0.363 | 0.285 | 0.159 |
| ILMN_2880346 | *Rrp1b* | ribosomal RNA processing 1 homolog B (S. cerevisiae); putative intergenic | Chr10: 72.783483 | 10.088 | 11.3 | Chr17: 33.110219 | -0.557 | 68 | 3.95E-07 | 0.476 | 0 | 0.999 |
| **Rs** |  |  |  |  |  |  | \| **5.926** \| \| --- \| \|  \| |  |  |  |  |  |
| **Re** |  |  |  |  |  |  | **0.1185** |  |  |  |  |  |
| **Rn** |  |  |  |  |  |  | **29.77** |  |  |  |  |  |
| **Ra** |  |  |  |  |  |  | **0.5954** |  |  |  |  |  |

Table 4. Top 50 genes that their expression levels are most correlated to that of Pgf.

| **Record** | **Symbol** | **Description** | **Location (Chr: Mb)** | **Mean Expr** | **Max LRS** | **Max LRS Location (Chr: Mb)** | **Sample r** | **N Cases** | **Sample**  **R**  **(absolute)** |
| --- | --- | --- | --- | --- | --- | --- | --- | --- | --- |
| ILMN_1254107 | *Trappc11* | trafficking protein particle complex 11 | Chr8: 47.413048 | 6.5067297 | 10.5 | Chr18: 60.000000 | 0.680683 | 68 | 0.680683 |
| ILMN_2680076 | *Ifi205* | interferon activated gene 205 | Chr1: 174.015071 | 6.6718243 | 12.1 | Chr2: 109.782694 | 0.6377297 | 68 | 0.63773 |
| ILMN_2671214 | *4921524J06Rik* | RIKEN cDNA 4921524J06 gene | Chr17: 88.645468 | 6.6248513 | 8.3 | Chr11: 103.177520 | 0.6283048 | 68 | 0.628305 |
| ILMN_2704335 | *Ccdc135* | coiled-coil domain containing 135 | Chr8: 95.059107 | 7.2201486 | 11.3 | Chr5: 24.371012 | -0.623526 | 68 | 0.623526 |
| ILMN_2724985 | *1700041C02Rik* | RIKEN cDNA 1700041C02 gene | Chr4: 119.359581 | 6.8882027 | 13.7 | Chr7: 87.221331 | 0.6222857 | 68 | 0.622286 |
| ILMN_2892518 | *Sp140* | Sp140 nuclear body protein | Chr8: 0.128238 | 6.5207433 | 17.2 | Chr19: 11.923268 | 0.6196034 | 68 | 0.619603 |
| ILMN_2701920 | *4930433N12Rik* | RIKEN cDNA 4930433N12 gene | Chr9: 3.134929 | 6.5662027 | 11.5 | Chr7: 87.733869 | 0.6151125 | 68 | 0.615112 |
| ILMN_2772130 | *1700080E11Rik* | RIKEN cDNA 1700080E11 gene | Chr9: 105.143418 | 6.4762568 | 13.3 | Chr9: 29.939029 | 0.6113324 | 68 | 0.611332 |
| ILMN_2594919 | *1500026H17Rik* | RIKEN cDNA 1500026H17 gene | Chr10: 89.700709 | 6.6082838 | 12.5 | Chr7: 87.733869 | 0.6100323 | 68 | 0.610032 |
| ILMN_2551824 | *Cd44* | CD44 antigen (Indian blood group) | Chr2: 102.744459 | 6.5545676 | 18.6 | Chr7: 87.733869 | 0.6089935 | 68 | 0.608994 |
| ILMN_2529177 | *Agxt2* | alanine-glyoxylate aminotransferase 2 | Chr15: 10.399081 | 6.6179595 | 8.4 | Chr7: 87.733869 | 0.6085223 | 68 | 0.608522 |
| ILMN_3108021 | *Efcab4b* | EF-hand calcium binding domain 4B | Chr6: 127.611476 | 7.0016621 | 10.8 | Chr7: 87.733869 | -0.603837 | 68 | 0.603837 |
| ILMN_1245690 | *LOC237060* | similar to hypothetical protein D12Ertd771e | ChrX: 137.203578 | 6.4872432 | 15.4 | Chr7: 87.733869 | 0.6030606 | 68 | 0.603061 |
| ILMN_2665975 | *Olfr1254* | olfactory receptor 1254 | Chr2: 89.788540 | 6.5073243 | 12.6 | Chr16: 61.486102 | 0.6029473 | 68 | 0.602947 |
| ILMN_1228313 | *LOC231959* |  | Chr6: 50.636596 | 6.451527 | 12.7 | Chr7: 139.468135 | 0.599631 | 68 | 0.599631 |
| ILMN_2751822 | *Dsg1b* | desmoglein 1 beta | Chr18: 20.409631 | 6.6641216 | 12.8 | Chr4: 127.422213 | 0.5991795 | 68 | 0.599179 |
| ILMN_1225841 | *Shc4* | SHC (Src homology 2 domain containing) family, member 4 | Chr2: 125.629733 | 6.4732838 | 8.4 | Chr15: 83.096920 | 0.5989463 | 68 | 0.598946 |
| ILMN_2507540 | *Edaradd* | EDAR (ectodysplasin-A receptor)-associated death domain | Chr13: 12.477520 | 6.6491622 | 12.7 | Chr7: 87.221331 | 0.5969094 | 68 | 0.596909 |
| ILMN_2668488 | *Dars* | aspartyl-tRNA synthetase | Chr1: 128.364017 | 6.4993919 | 16.1 | Chr7: 87.733869 | 0.5962008 | 68 | 0.596201 |
| ILMN_2656200 | *Fam133b* | Family with Sequence similarity 133, mamber B | Chr5: 3.569480 | 6.5426622 | 11.6 | Chr2: 163.806751 | 0.5959249 | 68 | 0.595925 |
| ILMN_2460292 | *8430427H17Rik* | RIKEN cDNA 8430427H17 gene | Chr2: 153.459756 | 6.432027 | 11.9 | Chr7: 87.733869 | 0.5948212 | 68 | 0.594821 |
| ILMN_1232317 | *Sstr5* | somatostatin receptor 5 | Chr17: 25.491244 | 6.4720406 | 9.4 | Chr4: 65.944234 | 0.593561 | 68 | 0.593561 |
| ILMN_1217423 | *BC037112* | cDNA sequence BC037112 | Chr5: 34.463995 | 6.4691216 | 7.4 | Chr18: 70.159002 | 0.5935491 | 68 | 0.593549 |
| ILMN_2427420 | *2610039C10Rik* | RIKEN cDNA 2610039C10 gene | Chr16: 90.719543 | 6.4359459 | 14.2 | Chr6: 37.854985 | 0.5931919 | 68 | 0.593192 |
| ILMN_2627400 | *Lrrc2* | leucine rich repeat containing 2 | Chr9: 110.983956 | 6.670473 | 10.1 | Chr7: 107.369034 | 0.5922667 | 68 | 0.592267 |
| ILMN_2559943 | *A230055O06Rik* |  | Chr13: 84.387417 | 6.5943108 | 11 | Chr10: 45.611814 | 0.5919928 | 68 | 0.591993 |
| ILMN_1228185 | *Ubxd4* | UBX domain protein 2A (p97 adaptor protein) | Chr12: 4.879027 | 6.4745676 | 13.8 | Chr9: 29.939029 | 0.591323 | 68 | 0.591323 |
| ILMN_2606345 | *Tmtc3* | transmembrane and tetratricopeptide repeat containing 3 | Chr10: 100.444298 | 6.4623108 | 10.7 | Chr4: 127.422213 | 0.5912402 | 68 | 0.59124 |
| ILMN_2534628 | *Gm397* | gene model 397, (NCBI) | Chr7: 11.065414 | 6.4122568 | 15 | Chr16: 44.143640 | 0.5912117 | 68 | 0.591212 |
| ILMN_2510641 | *4930487D11Rik* | RIKEN cDNA 4930487D11 gene | Chr5: 38.349068 | 6.6178784 | 11.7 | Chr7: 139.468135 | 0.5888692 | 68 | 0.588869 |
| ILMN_2434621 | *0610039K10Rik* | RIKEN cDNA 0610039K10 gene | Chr2: 163.645416 | 6.4691486 | 9.4 | Chr7: 87.733869 | 0.5882896 | 68 | 0.58829 |
| ILMN_2628764 | *1700108F19Rik* | RIKEN cDNA 1700108F19 gene | Chr14: 76.686871 | 6.5944324 | 13.3 | Chr7: 87.733869 | 0.5866096 | 68 | 0.58661 |
| ILMN_2520582 | *Plxna4* | plexin A4 | Chr6: 32.145937 | 7.2863649 | 18.5 | Chr7: 87.733869 | -0.585801 | 68 | 0.585801 |
| ILMN_2492956 | *Csn1s1* | casein alpha s1 | Chr5: 87.681969 | 6.4479189 | 15.8 | Chr7: 132.257029 | 0.5848225 | 68 | 0.584822 |
| ILMN_2437207 | *scl000242.1_32* |  | Chr7: 122.997639 | 6.412 | 12.1 | Chr7: 87.733869 | 0.5840568 | 68 | 0.584057 |
| ILMN_1233362 | *Map7d1* | microtubule-associated protein 7 domain containing 1 | Chr4: 126.238563 | 6.8730811 | 7.9 | Chr19: 58.686202 | -0.583515 | 68 | 0.583515 |
| ILMN_1240551 | *Psg19* | pregnancy specific glycoprotein 19 | Chr7: 18.796991 | 6.5718378 | 12.7 | Chr7: 87.733869 | 0.583355 | 68 | 0.583355 |
| ILMN_1240613 | *Pdzd4* | PDZ domain containing 4 | ChrX: 73.798781 | 6.5518108 | 9.9 | Chr2: 144.092011 | 0.5828758 | 68 | 0.582876 |
| ILMN_1232210 | *D030034I04Rik* | weakly similar to AMINO ACID TRANSPORTER NAT-2 | Chr15: 97.050248 | 6.6106216 | 10.5 | Chr6: 95.010274 | 0.582813 | 68 | 0.582813 |
| ILMN_3160486 | *Tmem158* | transmembrane protein 158 | Chr9: 123.259976 | 6.8013919 | 12 | Chr19: 6.516097 | 0.5822526 | 68 | 0.582253 |
| ILMN_2995190 | *Olfr899* | olfactory receptor 899 | Chr9: 38.368416 | 6.4333919 | 11.9 | Chr4: 127.422213 | 0.5822053 | 68 | 0.582205 |
| ILMN_1247393 | *Muc10* | mucin 10, submandibular gland salivary mucin | Chr5: 88.327956 | 6.441473 | 10.2 | Chr7: 137.867812 | 0.5821748 | 68 | 0.582175 |
| ILMN_1236143 | *Tcrg-V2* | T-cell receptor gamma, variable 2 | Chr13: 19.336623 | 6.3150136 | 12.6 | Chr7: 87.221331 | 0.5820077 | 68 | 0.582008 |
| ILMN_2704979 | *B3gnt4* | UDP-GlcNAc:betaGal beta-1,3-N-acetylglucosaminyltransferase 4 | Chr5: 123.511787 | 6.6603784 | 13.5 | Chr7: 143.090958 | 0.5815716 | 68 | 0.581572 |
| ILMN_2504796 | *Tnrc6b* | trinucleotide repeat containing 6b | Chr15: 80.892376 | 6.818527 | 16 | Chr18: 78.806816 | -0.580535 | 68 | 0.580535 |
| ILMN_2711910 | *Ifnb1* | interferon beta 1, fibroblast | Chr4: 88.522138 | 6.4933108 | 8.9 | Chr10: 45.611814 | 0.5805003 | 68 | 0.5805 |
| ILMN_2883277 | *EG209380* | predicted gene, EG209380 | Chr7: 106.423831 | 6.5942432 | 14 | Chr7: 26.451667 | 0.5793498 | 68 | 0.57935 |
| ILMN_2637059 | *Tcstv3* | 2-cell-stage, variable group, member 3 | ChrZ: 1.000000 | 6.6295676 | 8.2 | Chr12: 9.742519 | 0.5780315 | 68 | 0.578031 |
| ILMN_2931277 | *Abca13* | ATP-binding cassette, sub-family A (ABC1), member 13 | Chr11: 9.451365 | 6.5808919 | 8.1 | Chr3: 128.292533 | 0.5773831 | 68 | 0.577383 |
| ILMN_1215523 | *Zc3h12c* | zinc finger CCCH type containing 12C; putative intergenic | Chr9: 51.913573 | 6.409 | 15.5 | Chr7: 87.733869 | 0.576 | 68 | 0.576 |
|  |  |  |  |  |  |  | **Rs: 23.874511** | **3400** | **Rn: 29.82894** |
|  |  |  |  |  |  |  | **Re:**  **0.4774902** | **68** | **Ra: 0.596579** |
